# Incorporation and properties of the *tris*-fluoromethylated motif into heterocycles, amino acids and analogues of elexacaftor and tezacaftor

**DOI:** 10.1039/d6sc04239b

**Published:** 2026-07-03

**Authors:** Josephine M. Stewart, Bruno A. Piscelli, David B. Cordes, Rodrigo A. Cormanich, David O'Hagan

**Affiliations:** a EaStChem School of Chemistry, University of St Andrews North Haugh, St Andrews KY16 9ST UK do1@st-andrews.ac.uk; b Universidade Estadual de Campinas (UNICAMP), Instituto de Química Monteiro Lobato Street, Campinas Sao Paulo 13083-862 Brazil cormanich@unicamp.br

## Abstract

The synthesis and properties of the *tris*-fluoromethylated motif, (FCH_2_)_3_C-, a *tert*-butyl analogue with a fluorine atom on each methyl group, is explored in a bioactives discovery context. Aldehyde 9 is prepared on a gramme scale as a building block and incorporated into nitrogen heterocycles using Pictet–Spengler and oxidative cyclisations with *ortho*-aryldiamines. A Seyferth–Gilbert homologation delivered acetylene 24 which was progressed through Sonogashira and then Larock protocols to generate 2-substituted *tris*-fluoromethylated indoles. A Horner–Wadsworth–Emmons protocol with aldehyde 9 gave conjugated ketone 28 which was progressed to amino acid 32 after an asymmetric reduction to alcohol 29 and then employing an Overman rearrangement to introduce the amino group in a stereospecific manner. Comparative properties were measured on a series of pivaloyl esters (log *P*) and pivalic acids (p*K*_a_) assessing the impact of mono-, di- and tri-fluorination on the *tert*-butyl group. Progressive fluorination lowered log *P* (more hydrophilic) of the esters, and also lowered the p*K*_a_ (more acidic) of the pivalic acids. The study also contrasted the influence of a (Me)_2_(CF_3_)C- against a (FCH_2_)_3_C- substituent. The latter *tris*-fluoromethylated motif displays a greater electron withdrawing power when the fluorines are kept separate, over when they are clustered in a –CF_3_ group. The steric impact of the *tris*-fluoromethylated motifs in 28, and also the reduced alcohol 29, was apparent by comparing ^1^H-NMR chemical shifts of the olefinic hydrogens with analogues with none, one or two fluorines on the *tert*-butyl group. These trends were supported by computation which attributed changes in chemical shifts to through space interactions between –CH_2_F fluorine atoms and the distal H^α^ olefinic hydrogens. The *tris*-fluoromethylated motif was incorporated to make analogues 57 and 66 of two current high profile active pharmaceutical ingredients, elexacaftor 47 and tezacaftor 58 respectively. log *P* comparisons again indicated an increase in polarity (lower log *P*) when switching (Me)_2_(CF_3_)C- to (FCH_2_)_3_C-. In the case of tezacaftor replacing the (Me)_2_(CH_2_OH)C- substituent with (FCH_2_)_3_C- made the molecule more lipophilic indicating that the polarity induced by three fluorines is not sufficient to outcompete an OH group.

## Abstract 2

New synthesis approaches and properties of the *tris*-fluoromethylated motif are explored relative to *tert*-butyl, in the context of medicinal chemistry.

## Graphical abstract

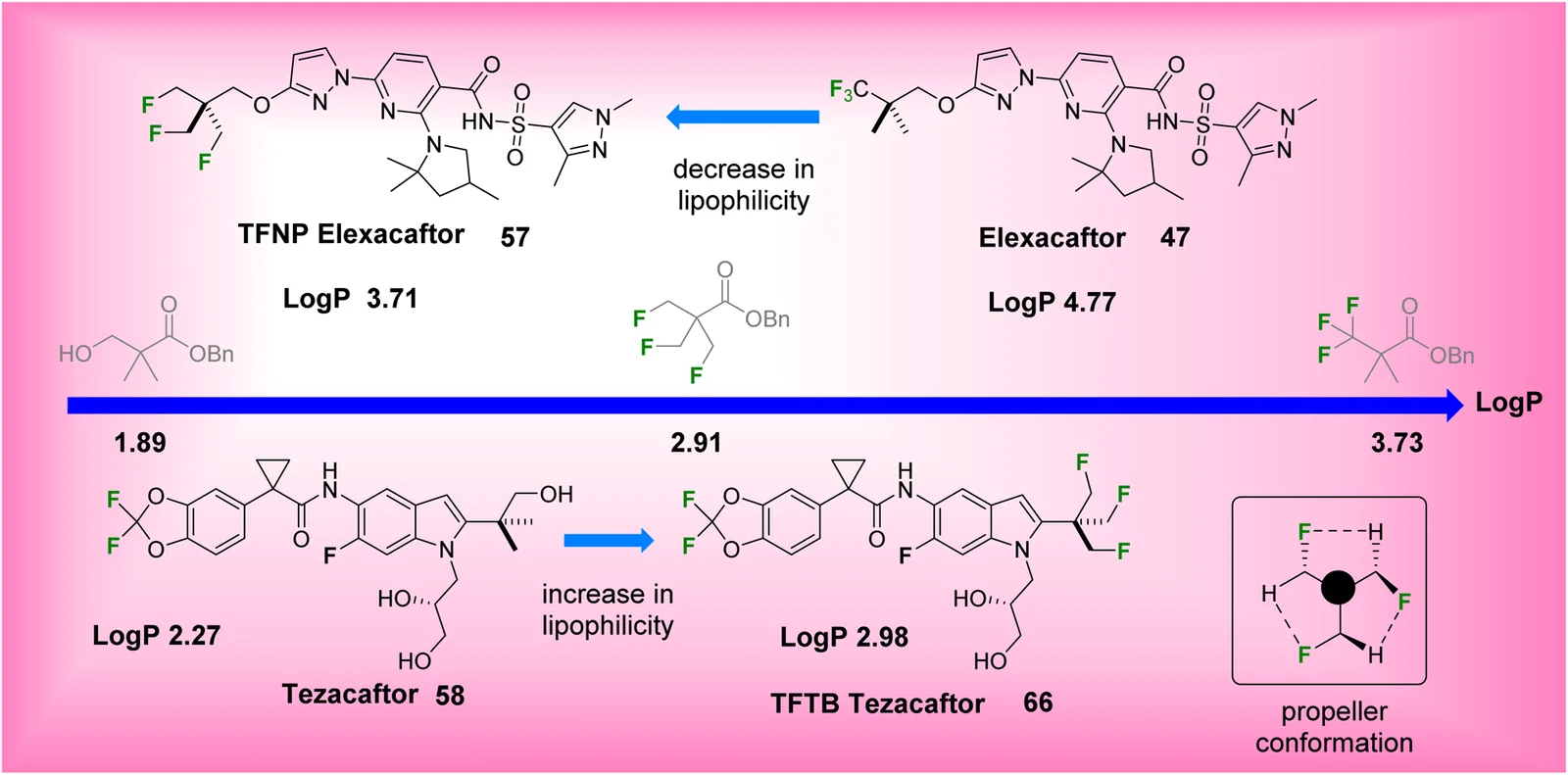

## Introduction

Selective fluorination has been of singular importance in the development of lead compounds to the clinic in the pharmaceuticals industry.^1^ The replacement of –H for –F in a compound supresses its metabolism,^2^ alters its lipophilicity^3^ and changes its electronic profile including altering p*K*_a_'s of acidic and basic functionalities.^4^ Consequently at least 20% of all FDA drugs approved in recent years contain fluorine to some extent.^5^ The –CF_3_ group has been prominent in this regard, although it is coming under some scrutiny going forward as compounds containing –CF_3_ may be affected under future PFAS regulations.^6^ Accordingly, there is a continued interest in selectively fluorinated motifs that do not contain –CF_3_ and extended perfluorocarbon substituents.^7^ In this respect we and others have been exploring the selective fluorination and properties of well-known aliphatic motifs such as cyclopropanes,^8^ cyclohexanes^3*c*,9^ and *tert*-butyl groups^10^ to assess properties relative to the parent aliphatics. In general, selective fluorination causes an increase in polarity relative to the corresponding aliphatic and therefore lowers log *P*. In this paper we continue to explore selective fluorination of the *tert*-butyl group, placing a fluorine on each of the methyl groups (*tris*-fluoromethylated). This motif was sparsely represented in the literature until recently. In two early reports^11,12^ in the 1990's a synthesis and introduction of amine 1 into an antibiotic derivative was reported.

**Fig. 1:**
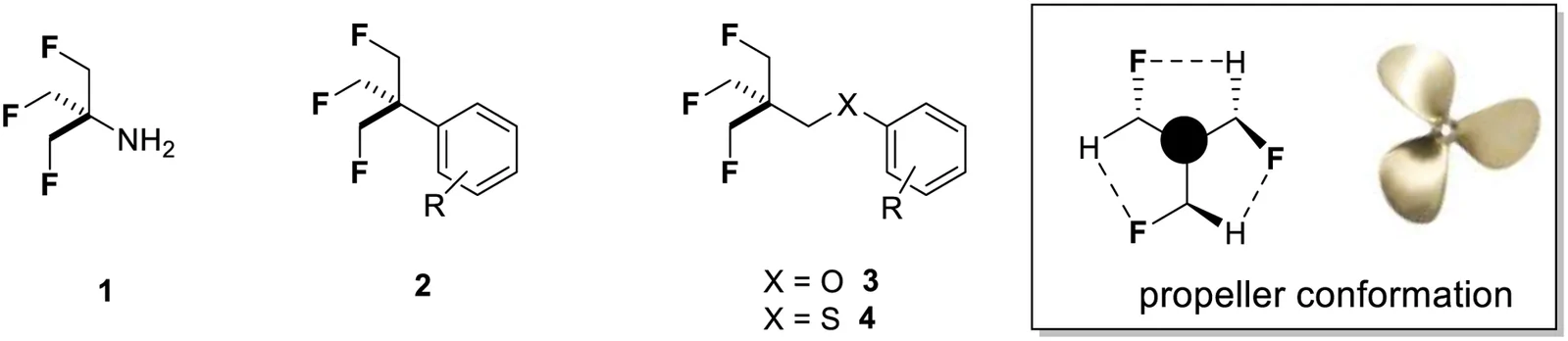
The structures of building blocks containing trifluoro *tert*-butyl groups. The motif adopts a propeller type conformation.^11,12^

**Scheme 1:**
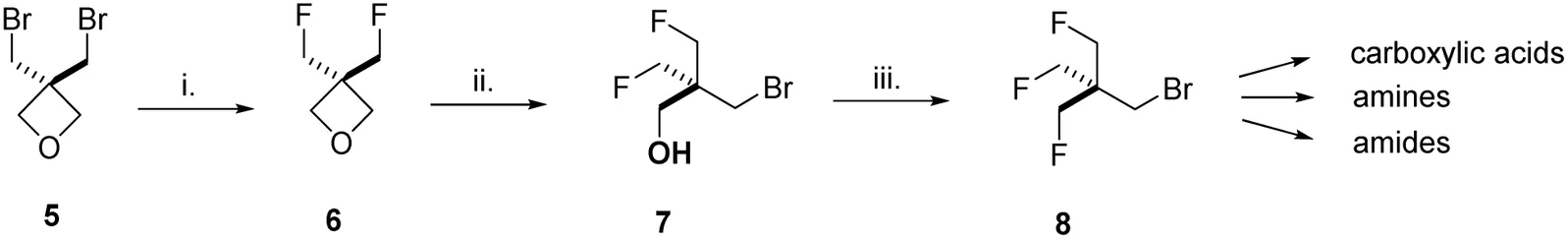
Enamine Ltd, multi-gram scale route to intermediate bromide 8. (i) CsF, diethyleneglycol, 69%. (ii) Liq. HBr, 86%. (iii) SF_4_, HF, 68%.^13^

More recently we have developed syntheses of (β,β′,β′-trifluoro-*tert*-butyl-aryl derivatives 2^10*a*^ and the (γ,γ′,γ′-trifluoro)neopentyl derivatives 3 and 4^10*b*^ and explored their conformation and some of their properties. X-Ray structures and computational analysis indicate that this motif has a preferred propeller type conformation where the fluorines of the C–F bonds repel each other and there are weak interactions to co-aligned C–H bonds as illustrated in Fig. 1. Most recently a paper from Enamine Ltd^13^ has reported a scaled-up route to bromide 8 (>100 g) from oxetane 5, as illustrated in Scheme 1, and demonstrated the chemical elaboration of bromide 8 to a range of carboxylic acids, amines and amide building blocks. So there is recent momentum around the synthesis and diversification of molecules containing this *tris*-fluoromethylated motif.

**Scheme 2:**
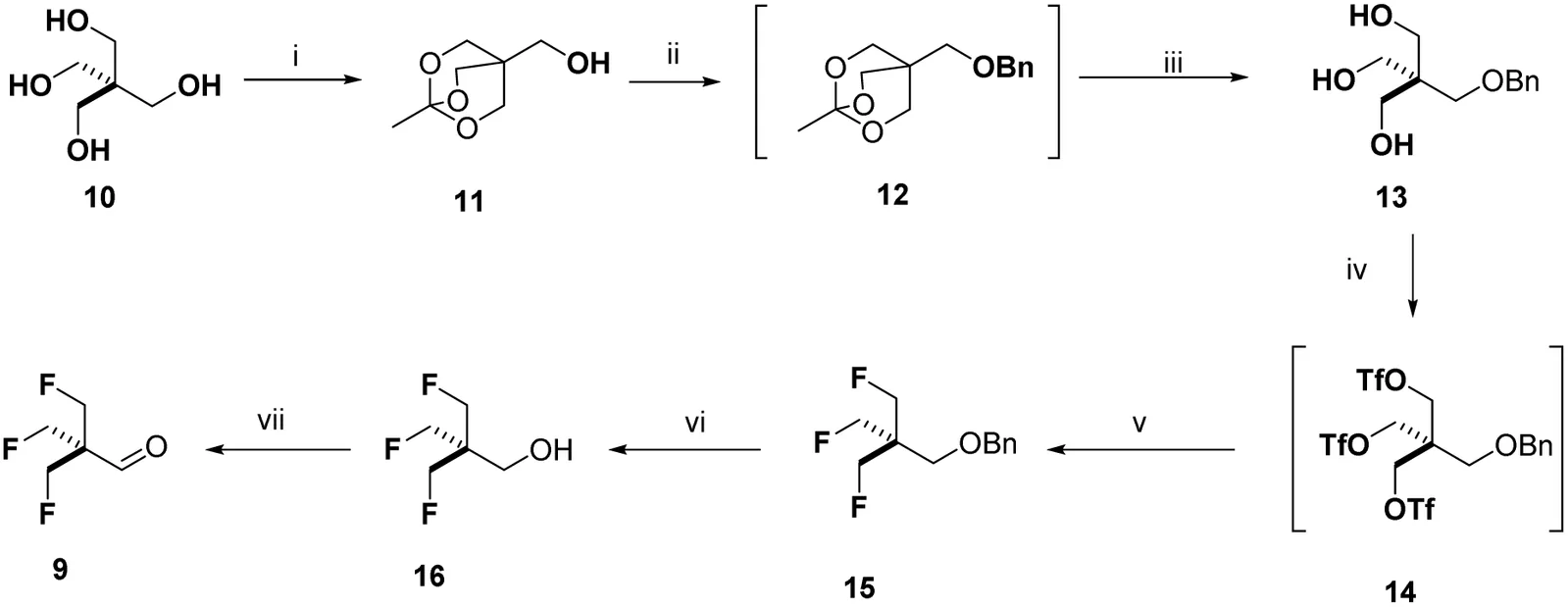
Gramme scale route to aldehyde 9. Reagents and conditions; (i) CH_3_C(OEt)_3_ (1.1 eq.), *p*-TsOH·H_2_O (10 mol%), toluene, *Δ*, 48 h, 80%. (ii) BnBr (1.2 eq.), NaH (60% dispersion in mineral oil, 1.5 eq.), toluene, *Δ*, 16 h. (iii) HCl (cat), MeOH, 40 °C, 2 h, 67% over two steps. (iv) Tf_2_O (4.5 eq.), pyridine (6 eq.), DCM, −40 °C – r.t., 2 h. (v) TBAF (4 eq.), THF, 0 °C – r.t., 2 h. (vi) H_2_ (10–15 bar), Pd(OH)_2_/C (20% loading on wet support, cat), EtOAc, r.t., 16 h, 90%. (vii) RuCl_3_, NaIO_4_, AcCN/H_2_O/EtOAc.

In this paper we have placed an emphasis on the (β,β′,β′-trifluoro)pivaldehyde 9 (Scheme 2) as a building block, and progress it synthetically towards heterocyclic systems and amino acids, and we also introduce it into scaffolds to prepare analogues of pharmaceuticals of current clinical and commercial relevance.

## Results and discussion

At the outset a multi-gram scale synthesis of alcohol 16 was developed as summarised in Scheme 2, which improved our previous route.^10*b*^

**Scheme 3:**
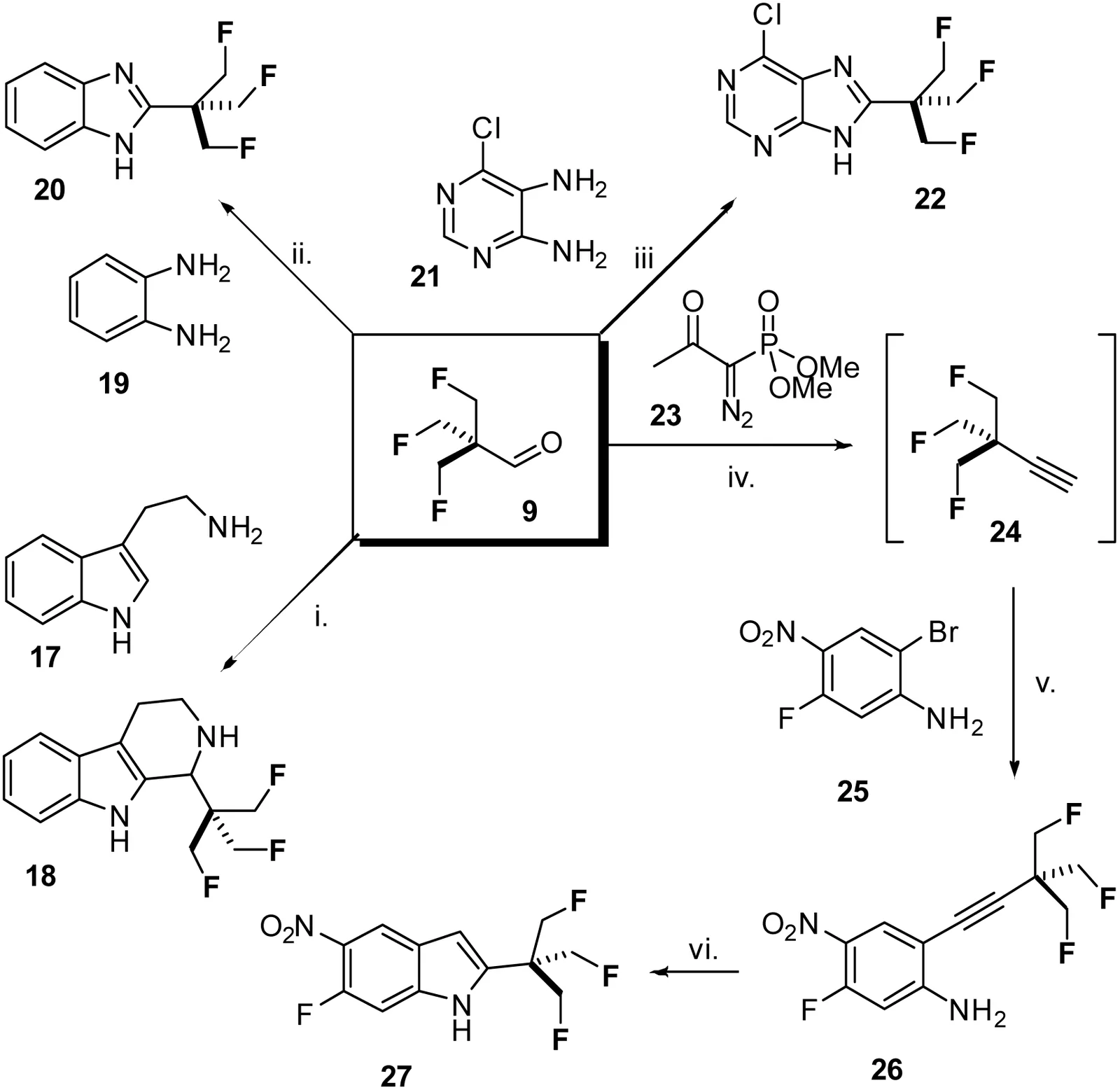
Incorporation of the *tris*-fluoromethylated motif into heterocyclic scaffolds using aldehyde 9. Reagents and conditions (i) 17 (1 eq.), T3P® (2 eq.), EtOAc, 120 °C, MW, 30 min, 34% (ii) 19 (1 eq.), NaHSO_3_ (1 eq.), DMA, 100 °C, 2 h, 35%. (iii) 21 (1 eq.) NaHSO_3_ (1 eq.), DMA, 100 °C, 1 h, 20%. (iv) 23 (1.2 eq.) Cs_2_CO_3_ (2.0 eq.), MeOH, 0 °C – r.t., 1 h. (v) 24 (2 eq.), 25 (1 eq.), Cs_2_CO_3_ (2 eq.), Pd(OAc)_2_ (4 mol%), XPhos (8 mol%), MeOH, *Δ*, 16 h, 21% over two steps. (vi) Pd(MeCN)_2_Cl_2_ (10 mol%), MeCN, *Δ*, 16 h, 29%.

This route to alcohol 16 started with an efficient orthoformate protection of tetrol 10 to generate alcohol 11. Benzylation followed by *in situ* deprotection gave benzyl ether 13 in good yield over these two steps.^14^ Fluorination was achieved by tri-triflation and then treatment of 14 without work up, with tetrabutylammonium fluoride (TBAF). This proved to be a satisfactory fluorination protocol progressing in 90% yield from 13 to benzyl ether 15. Benzyl ether cleavage by hydrogenation then gave alcohol 16. Finally, treatment of alcohol 16 with pyridinium chlorochromate (PCC) generated aldehyde 9 in 70% yield. The work-up was relatively straight forward. After removal of chromium waste by filtering through a silica plug, the solvent was distilled off to afford aldehyde 9 as a yellow oil. It was either used immediately or it could be stored at −20 °C for several weeks. Aldehyde 9 proved to be a relatively versatile intermediate for incorporations into heterocyclic scaffolds as illustrated in Scheme 3. For example, 9 was combined with tryptamine 17 to give the Pictet-Spengler^15^ product 18. It could also be progressed to benzimidazoles 20 and purines 22 by condensation/dehydrogenation reactions using NaHSO_3_ in hot dimethylacetamide (DMA)^16^ with *ortho*-aryldiamines 19 and 21 respectively. In another elaboration aldehyde 9 was treated with the Ohira–Bestmann reagent^17^23 to achieve a Seyferth–Gilbert^18^ homologation and generate acetylene 24. The resultant acetylene was too volatile to be conveniently isolated however after distillation with the reaction solvent, methanol, the solution was used directly in a Sonogashira protocol^19^ with the functionalised aryl bromide 25. This generated acetylene 26, which was a substrate for a Larock indole Pd-mediated cyclisation,^20^ to generate indole 27.

**Scheme 4:**
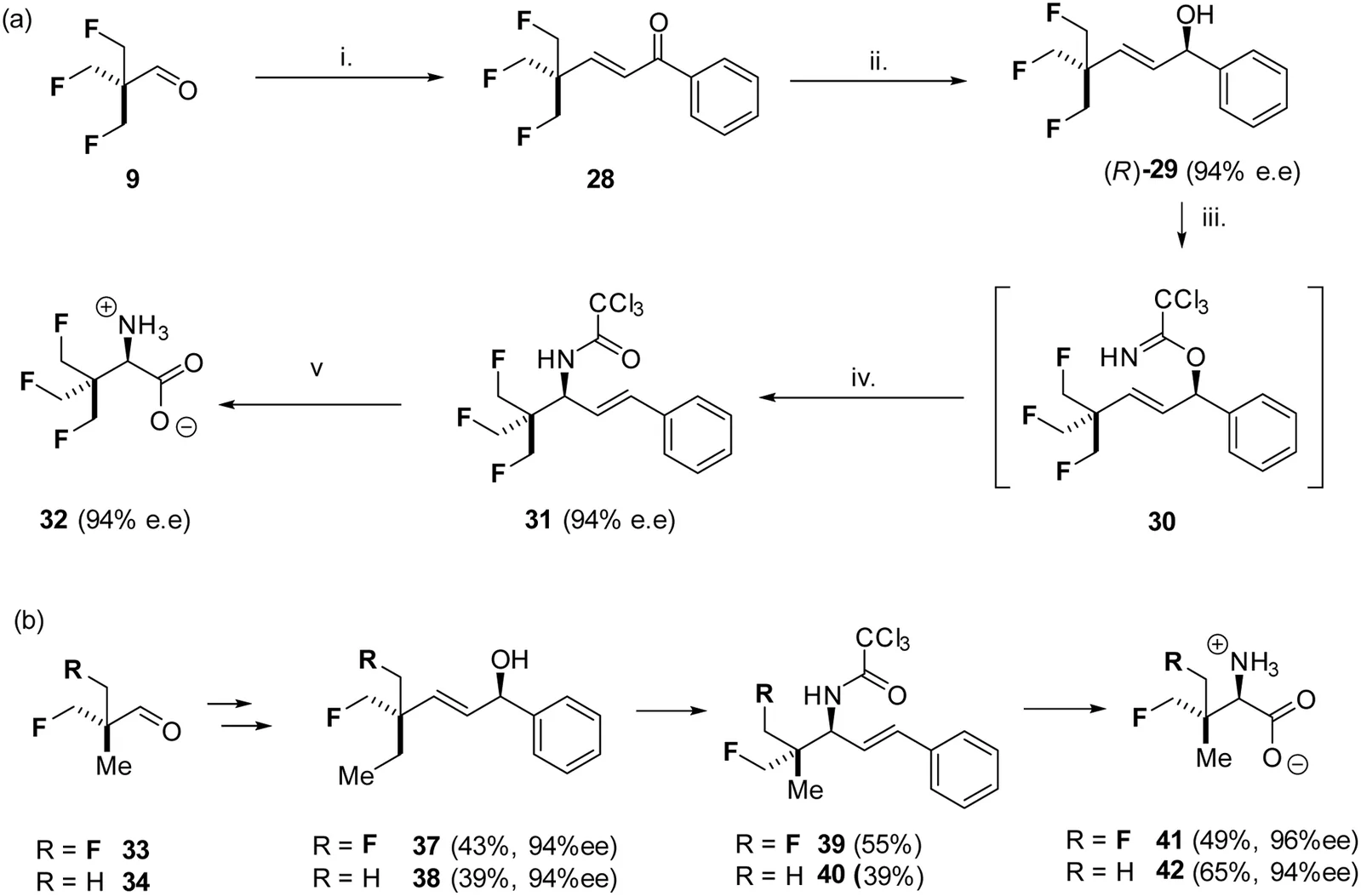
Overman rearrangement approach to amino acids 32–34. Reagents and conditions. (i) Diethyl (2-oxo-2-phenylethyl)phosphonate (1.1 eq.), NaH (60% dispersion in mineral oil, 1.3 eq.), THF, 0 °C – r.t., 6 h, 30% (ii) H_2_ (10 bar), [RuCl_2_{(*R*)-tol-BINAP}{(*S*)-dmapen}] (0.1 mol%), ^*t*^BuOK (0.5 mol%), ^*i*^PrOH, 0 °C, 16 h, 66%. (iii) DBU (0.5 eq.), trichloroacetonitrile (3 eq.), Et_2_O, r.t., 16 h, 20. (iv) Toluene, *Δ*, 16 h, 28% over two steps. (v) RuCl_3_ (1 mol%), NaIO_4_ (4 eq.), MeCN/H_2_O/EtOAc (1 : 1.5 : 1), 3 h, then 5 M aq NaOH (30 eq.), EtOH, r.t., 3 h, 70%.

Aldehyde 9 was also progressed to amino acid 32 in enantiomerically enriched (94% ee) form as illustrated in Scheme 4. This was accomplished by a Horner–Wadsworth–Emmons protocol^21^ to generate α,β-unsaturated ketone 28. In order to introduce asymmetry, the ketone was subject to an enantioselective reduction with the chiral ruthenium catalyst [RuCl_2_{(*S*)-tol-binap}{(*R*)-dmapen}], following a protocol described by Ohkuma *et al*.^22^ This proved to be satisfactory and generated allylic alcohol (*R*)-29 in a 94% ee. The absolute stereochemistry is assumed here following the literature expectation.^22*b*^ The α-amino nitrogen of the target amino acid was introduced *via* an Overman rearrangement^23^ which allowed positioning of the nitrogen immediately adjacent to the sterically intimidating quaternary centre. Accordingly, treatment of alcohol 29 with trichloroacetonitrile and DBU generated the trichloroacetimidate intermediate 30, which was not isolated but rearranged on heating to give allylic amide 31. Oxidative cleavage of the olefin in 31 with RuO_4_ under phase transfer conditions,^24^ followed by hydrolysis, gave the free amino acid 32. The enantiopurity of the amino acid was determined by Marfey's method,^25^ reacting the amino acid with 1-fluoro-2,4-dinitrophenyl-5-l-alanine-amide (FDAA) in a nucleophilic aromatic substitution reaction. In these analyses the d amino acid generally elutes after the l in a reverse phase C_18_ HPLC column, as was observed here and therefore we deduce the d-(*R*) stereochemistry for the amino acid. The stereo-integrity of the sigmatropic rearrangement is therefore consistent with an origin from (*R*)-29 and the enantiomeric excess of the amino acid which was also measured as 94% ee.

The Overman protocol^23^ was then used to prepare the difluoro- and mono-fluoro substituted amino acids 41 and 42 respectively, also in enantiomeric purities (∼94–96% ee) consistent with the asymmetric reduction of their corresponding ketones using the Ohkuma^22^ protocol.

**Fig. 2:**
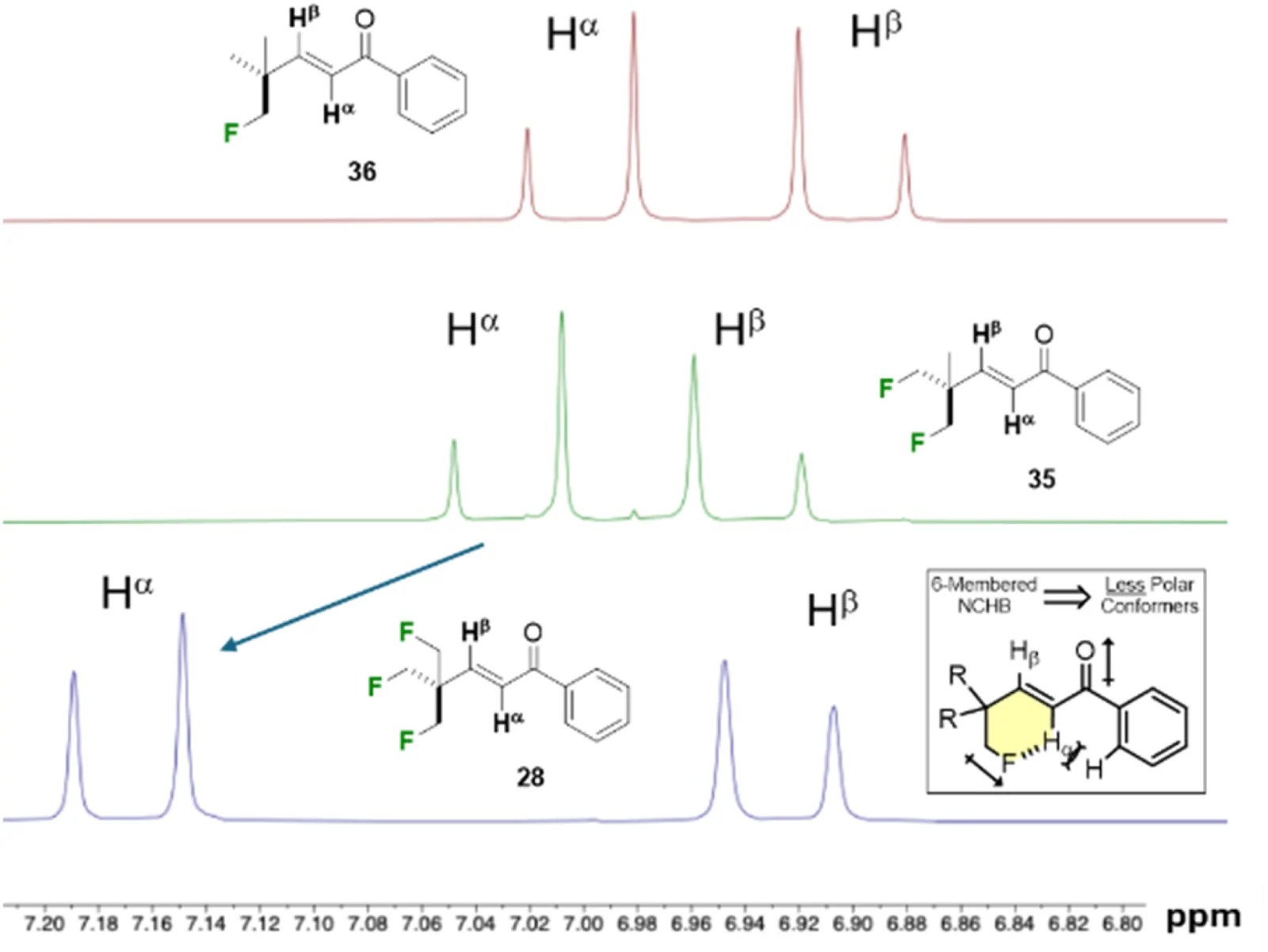
^1^H-NMR spectra of the olefinic region of conjugated ketones 36, 35, 28, showing progressive downfield shift of distal H^α^ proton with increasing fluorination.

The synthesis of the three amino acids 32, 41 and 42 progressed through conjugated ketones 28, 35 and 36 and allylic alcohols 29, 37 and 38 respectively. ^1^H-NMR of these intermediates showed a downfield chemical shift progression for the H^α^ protons of the olefins in each case with the level of fluorination of the *tert*-butyl group, indicative of the increasing influence of through space non classical CF⋯H^α^C hydrogen bonding. The H^β^ chemical shift remains much more constant. The relevant sections of the ^1^H-NMR spectra of the three conjugated ketones 36, 35 and 28 are shown in Fig. 2.^26^

The experimental and calculated Boltzmann-averaged chemical shifts of the olefinic protons (H^α^ and H^β^) in ketones 36, 35 and 28 and the corresponding alcohols 38, 37 and 29 are compared in Table 1. Chemical shifts were calculated at the SMD(CHCl_3_)-r^2^SCANh/pcSseg-2//r^2^SCANh/pc-2 level of theory^27^ as this approach provided the most accurate relative conformational population distribution among 30 tested theory levels when benchmarked against higher-level SMD(CHCl_3_)-DLPNO-CCSD(T)/CBS single point energies.^28^ Although the absolute values of the calculated chemical shifts are systematically overestimated by 0.5–1.0 ppm relative to experiment, the calculations successfully reproduce the progressive downfield shift observed experimentally for H^α^ in each case (ketones and alcohols) with increasing fluorination, while *δ*_H_^β^ remains largely unchanged. DFT-calculated absolute ^1^H chemical shifts are known to carry systematic errors that are sensitive to the level of theory, conformational sampling, and solvation model employed, with system-dependent deviations from experiment that are well documented in the literature.^29^ Nevertheless, since the conclusions drawn here rest entirely on the direction and magnitude of the changes in *δ*_H_ upon fluorination, rather than on the absolute values, this systematic offset does not affect the interpretation.

**Table 1:** Experimental and Boltzmann-averaged calculated ^1^H-NMR chemical shifts in ppm, for the proximal H^β^ and distal H^α^ hydrogens in the ketones and allylic alcohols

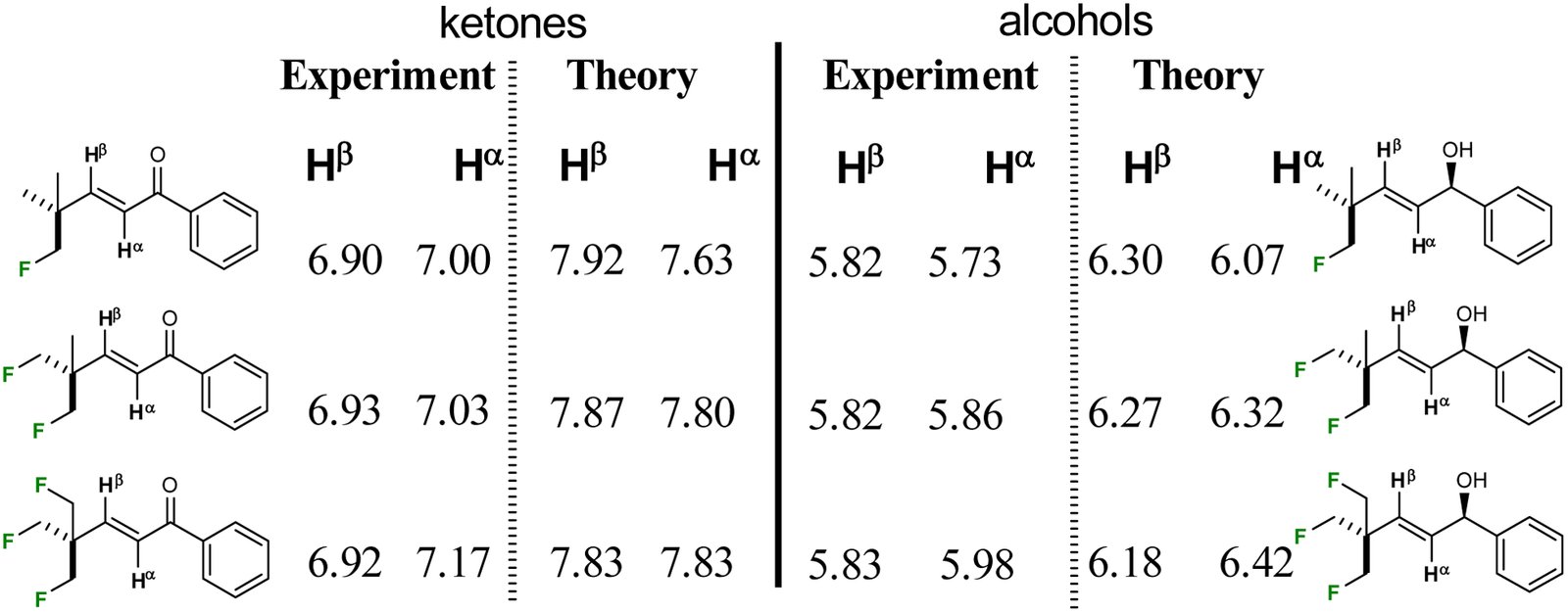

**Fig. 3:**
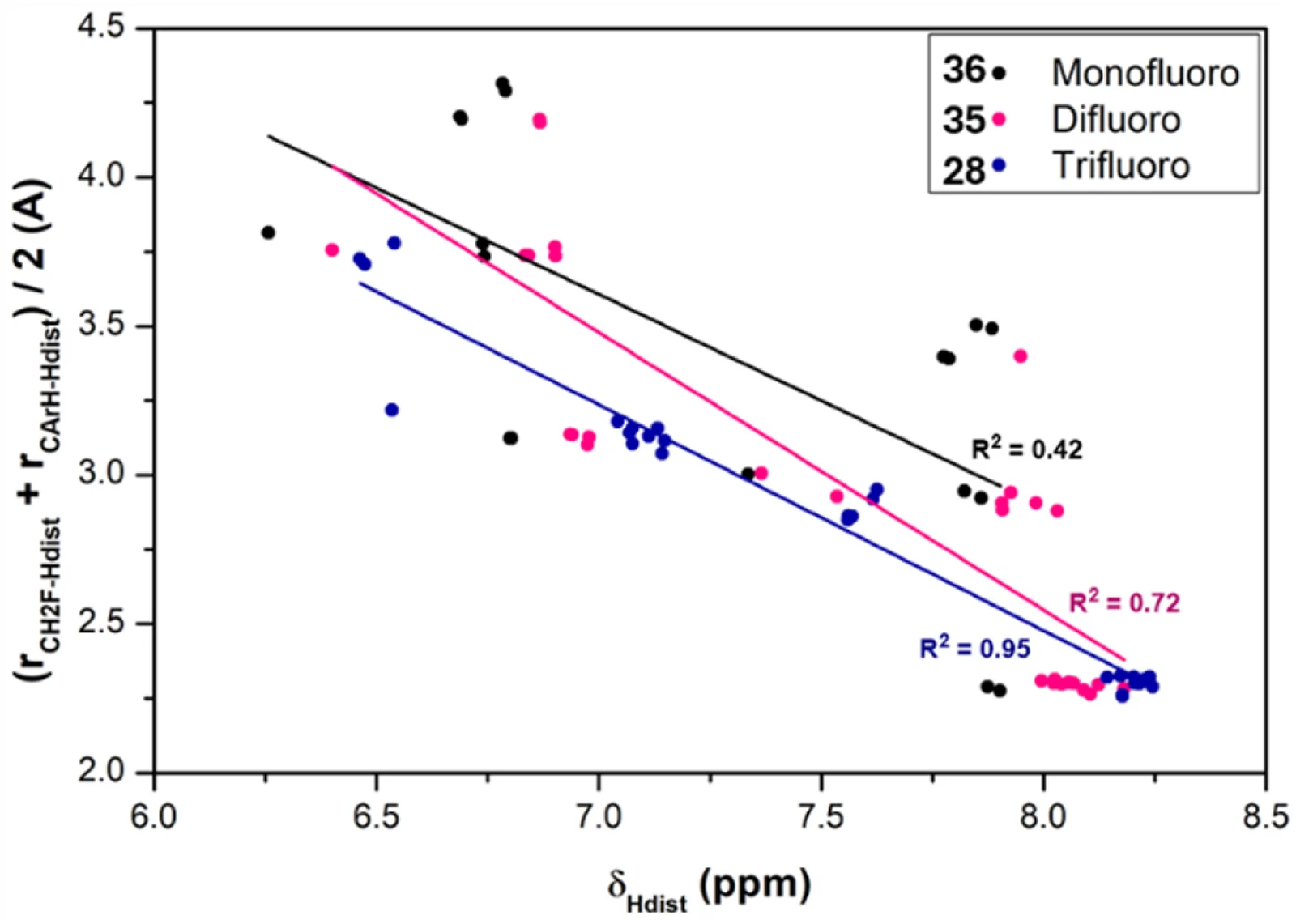
Plot of *δ*_Hdist_ against the average CF_2_H⋯H_dist_ + C_Ar_H⋯H_dist_ for ketones 28, 35 and 36.

The formation of an intramolecular 6-membered non-conventional hydrogen bond CH_2_F⋯H^α^ from the *t*Bu group to the distal distal olefinic proton, as illustrated in the inset in Fig. 2, could clearly influence the observed de-shielding, although a plot of the CH_2_F⋯H^α^ distance (*r*_CH_2_F–H_^α^) did not show a significant correlation with chemical shift (see SI Fig 1). By contrast, a plot of the C_Ar_H⋯H^α^ distance (*r*_CArH–Hα_) did reveal an associated downfield shift with distance as short as ∼2.05 Å, albeit in a non-linear manner (see SI Fig 2), indicative of de-shielding of H^α^ due to electronic depletion through C_Ar_H/H^α^C steric contacts. However a strong linear correlation emerged when the *δ*_H_^α^ is plotted against the average of the CH_2_F⋯H^α^ and C_Ar_H⋯H^α^ distances, for the ketones 28, 35 and 36 as illustrated in Fig. 3. For the monofluorinated ketone 36, the correlation is modest (*R*^2^ = 0.42), however, with increasing fluorination and the consequent increase of NCHB contacts the linearity improves substantially, reaching *R*^2^ = 0.72 for the difluoro derivative 35 and *R*^2^ = 0.95 for the trifluoro analogue 28. These results suggest that steric effects and NCHBs act cooperatively in governing the de-shielding of H^α^. Since the aromatic motif remains unchanged across the series, this analysis provides evidence that weak CH_2_F⋯H^α^ interactions contribute meaningfully to the progressive downfield shifts of H^α^ observed upon fluorination of both the ketone and alcohol derivatives (Table 2).

**Table 2:** Calculated Natural Chemical Shielding (NCS) components for the shielding tensors (*s*) for H^α^ of the ketones 28, 35 and 36 and allylic alcohols 29, 37 and 38

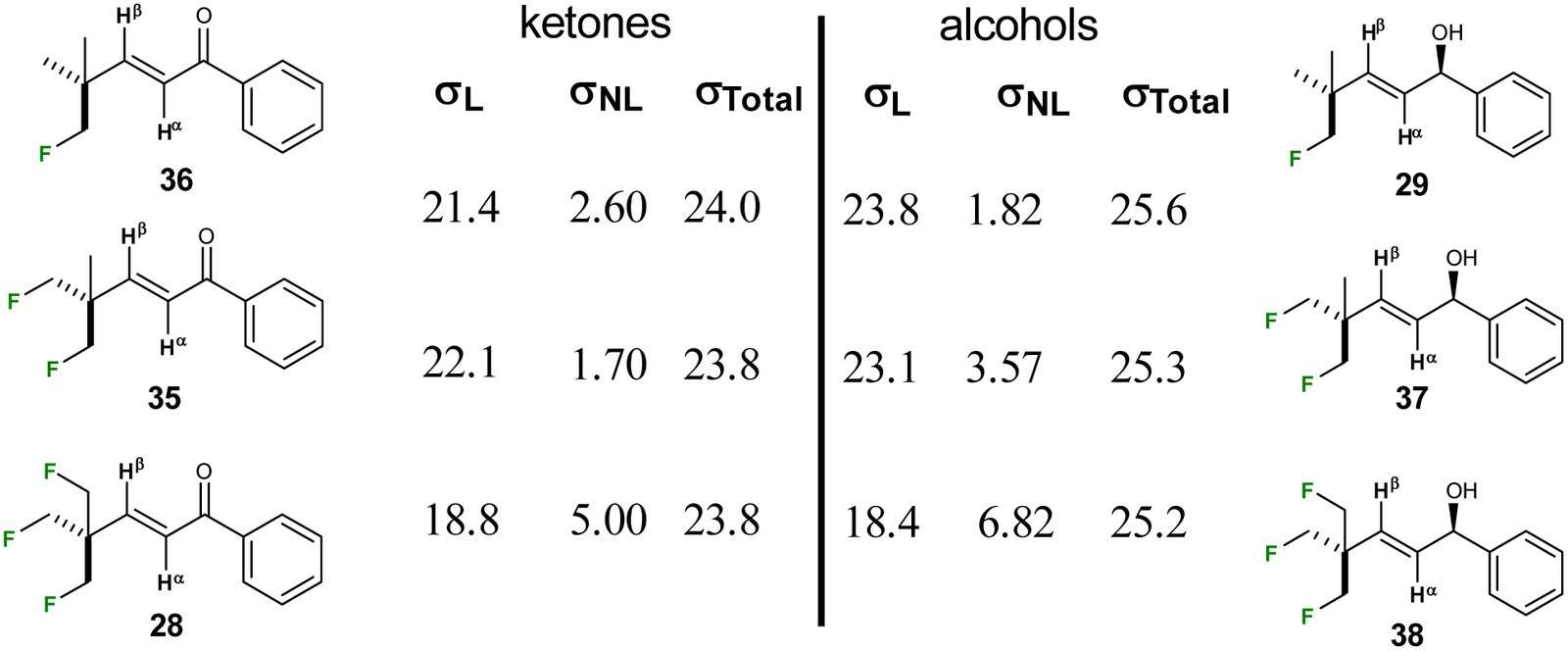

To gain further insight into the origin of the progressive de-shielding of H^α^, the total ^1^H-NMR shielding was analyzed using the Natural Chemical Shielding (NCS) framework, in which the shielding is partitioned into Lewis (L) and non-Lewis (NL) contributions.^30^ The shielding tensor (*σ*) is inversely proportional to the chemical shift (*δ*), thus, higher values of *σ* are translated into lower values of *δ*. The Lewis term reflects the static local electron density around the proton, describing how effectively the C–H bond itself and its immediate bonding environment screen the nucleus from the external magnetic field and reflects impacts of electrostatic/steric effects to shielding. In contrast, the non-Lewis term captures the magnetic response of the surrounding electronic environment, including contributions from low-lying antibonding orbitals accessed through hyperconjugation, such as 
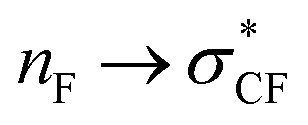
 in the context of NCHBs. Only the sum of these two terms corresponds to the experimentally observable chemical shift, while the individual L and NL contributions are not directly measurable and often compensate for one another. Although the individual L and NL contributions do not vary monotonically across the series, with *σ*_NL_ following a non-linear trend (2.63 → 1.70 → 5.00) and *σ*_L_ showing a net decrease across the series (21.4 → 22.1 → 18.8), with the difluorinated derivative displaying intermediate and less predictable conformational averaging, their sum (*σ*_Total_) consistently reproduces the overall experimental de-shielding trend. Notably, the computed Δ*σ*_Total_ of −0.20 ppm from the mono-to the trifluorinated compound is in excellent quantitative agreement with the experimental (+0.17 ppm) and Boltzmann-averaged computational (+0.20 ppm) trends, lending confidence to the NCS partitioning as a reliable framework for interpreting the origin of the effect. The net decrease in *σ*_L_ is consistent with stronger electrostatic de-shielding as the population of CH_2_F⋯H^α^ contacts increases, while the increase in *σ*_NL_ from 2.63 to 5.00 between the mono- and trifluorinated analogues is consistent with an enhanced through-space magnetic response, most likely arising from 
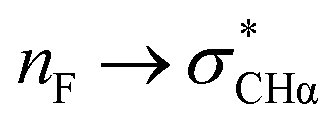
 delocalization. Conformational analysis further reveals a linear increase in the population of conformers forming short CH_2_F⋯H^α^ contacts, from 19.5% in the monofluorinated derivative to 43.2% and 60.0% in the di- and tri-fluorinated systems, respectively, indicating that fluorination progressively increases the likelihood of a stabilizing CH_2_F⋯H_α_ interaction. Taken together, these results show that fluorination enhances both the occurrence and the electronic effectiveness of the CH_2_F⋯H^α^ interaction. At higher degrees of fluorination, the experimentally observed de-shielding of H^α^ therefore cannot be accounted for by changes in the local C–H^α^ bonding environment alone, such as those expected from the inductive effect, but requires the additional contribution of non-local, field-responsive electronic effects associated with the intramolecular CH_2_F⋯H^α^ interactions.

The energetics associated with the NCHBs were also evaluated. NBO analyses^31^ suggest that the CH_2_F⋯H^α^ contacts are predominantly electrostatic in nature, exhibiting coulombic stabilization energies of approximately 11 kcal mol^−1^ in all cases, with little to no electron sharing through 
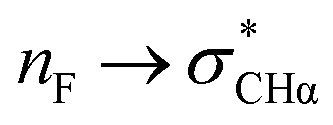
 delocalization. Indeed, this interaction contributes less than 0.1 kcal mol^−1^ in all systems except ketone 36, where it is weakly stabilizing at 0.2 kcal mol^−1^ (see Fig. S11 and S12 for further details). NCI analyses^32^ corroborate the NBO results, displaying blue-green isosurfaces between CH_2_F and H^α^, consistent with weakly stabilizing NCHBs. Likewise, QTAIM analyses^33^ do not reveal bond critical points (BCPs) connecting CH_2_F and H^α^, except in ketone 36, further supporting the weak electron-sharing and predominantly electrostatic character of these NCHBs.

**Scheme 5:**
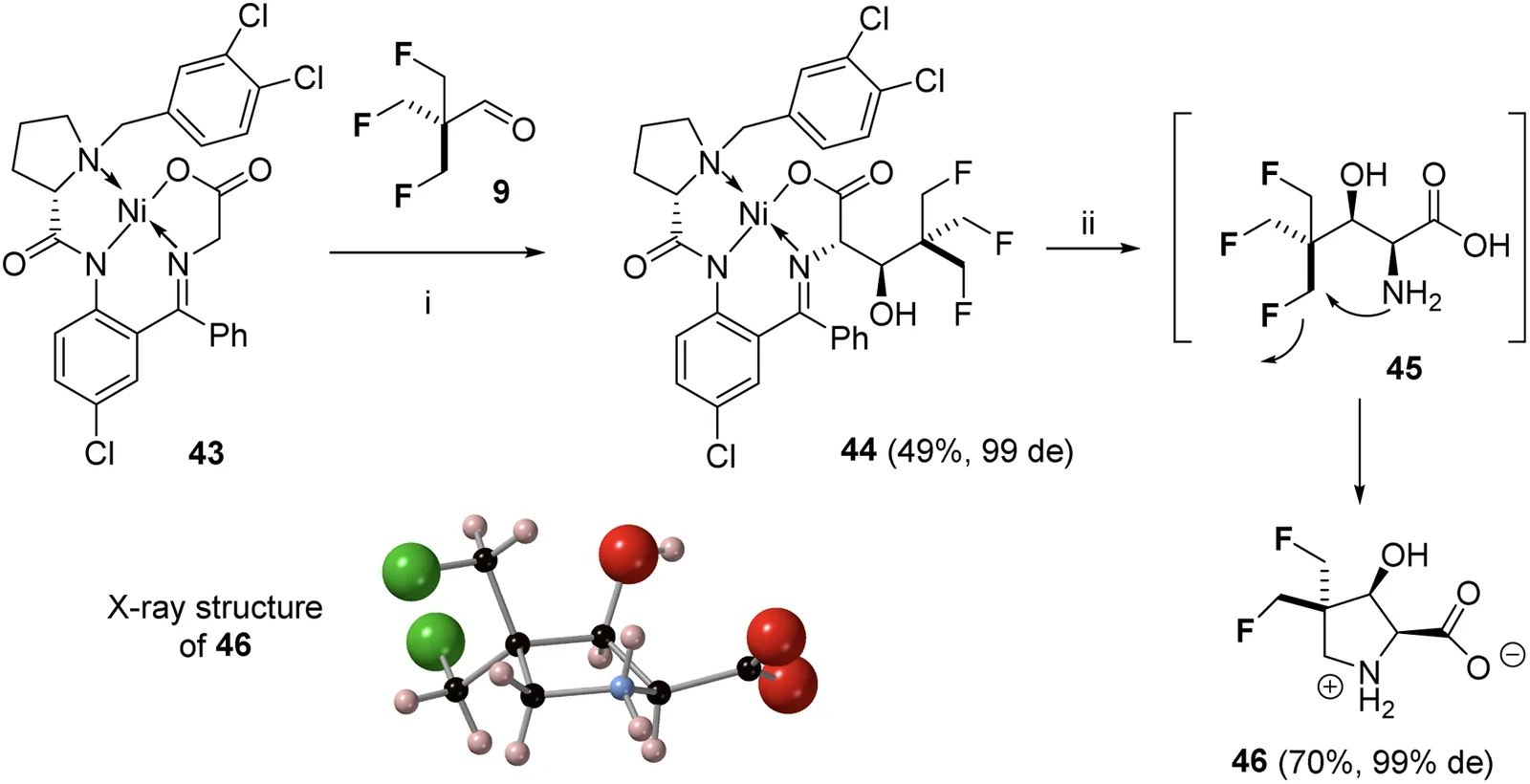
Reagents and conditions: (i) MeONa (3 eq.), 9 (1.5 eq.), MeOH, r.t., 10 min. (ii) HCl (6 M), MeOH, *Δ*, 20 min, followed by NH_3_ and aq. workup. X-ray structure of 46.

In another approach to an amino acid synthesis, an asymmetric aldol reaction was explored using the Belokon–Soloshonok^34^ chiral glycine auxiliary as illustrated in Scheme 5. Accordingly, aldehyde 9 was condensed with Ni-complex 43. This generated the aldol product 44 as a single diastereoisomer. Standard acidic methanol work-up to release the amino acid from the auxiliary did not furnish the expected amino acid 45, but instead resulted in the product from an efficient intramolecular cyclisation, involving fluoride displacement from one of the fluoromethyl groups, to generate hydroxyproline 46 as the sole product (70%, 99% de).

The structure and stereochemistry of 46 was confirmed by single crystal X-ray structure analysis as illustrated in Scheme 5. Fluoride is not a common leaving group; thus this outcome is indicative of the steric pressure that the trifluoro-*tert*-butyl group is placing on the amine functionality.

The electronic and lipophilic effects on fluorination of the *tert*-butyl groups were explored by measuring the p*K*_a_'s of the pivalic acids PA's 1–5 and the log *P*_s_ of the corresponding pivaloyl benzyl esters PE's 1–6, as determined using reverse phase HPLC.^35^

**Fig. 4:**
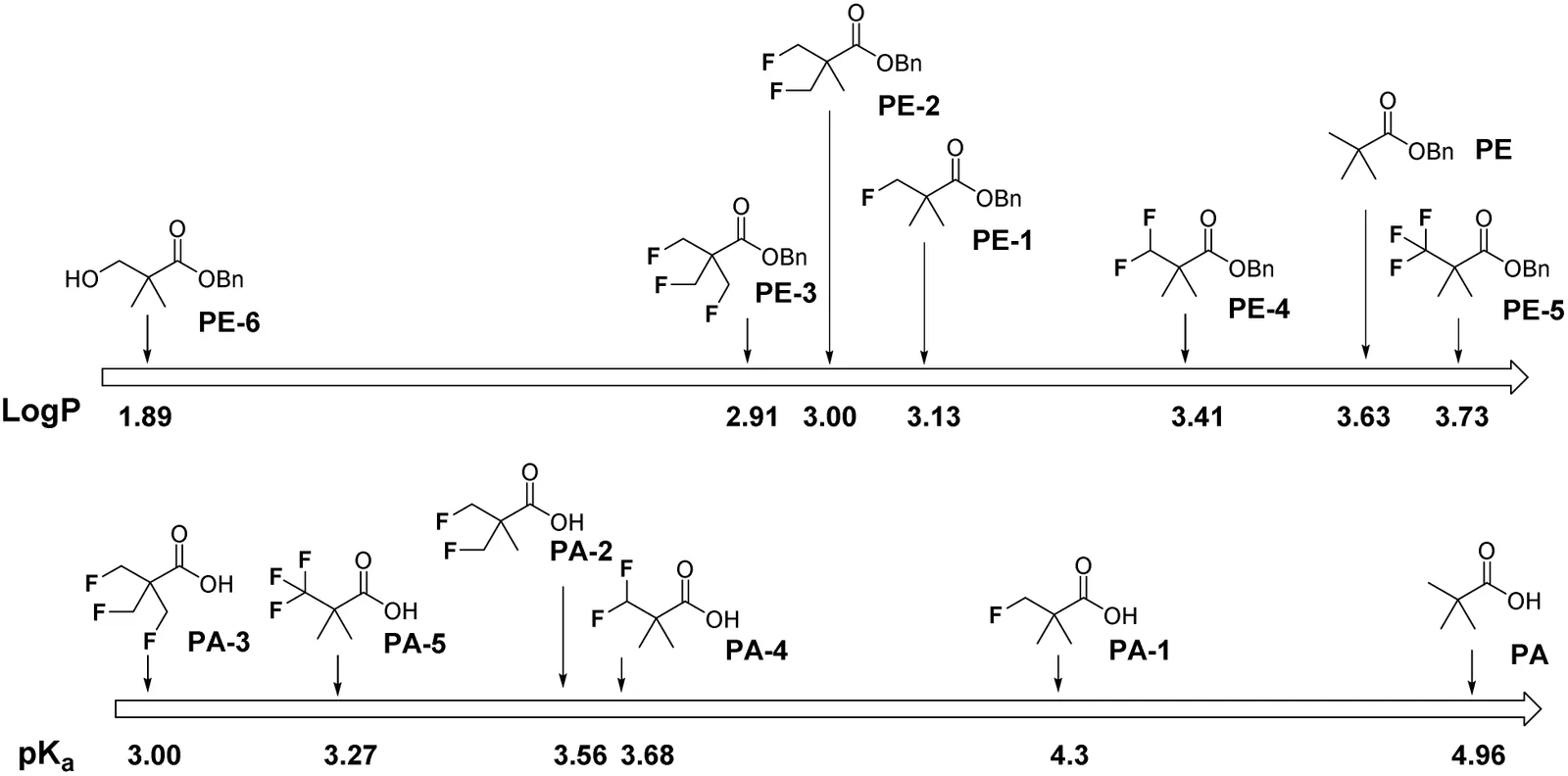
log *P* and p*K*_a_ comparisons of the fluorinated pivaloyl benzyl esters (PEs) and pivalic acids (PAs). The p*K*_a_'s were measured by titration and log *P*_s_ were determined by reverse phase HPLC, as previously described.^35^

The log *P* profile illustrated in Fig. 4 is notable in that the most polar fluorinated pivaloyl ester of the series is the γ,γ′,γ′-trifluoro ester PE-3, which is ∼*Δ*1.4 log *P* units more polar than benzyl pivalate PE. Polarity is non-linear with progressive fluorination, with mono-fluorination having the largest impact and then progressively less so with each subsequent fluorination. The difluoromethyl ester PE-4 is less polar than the γ,γ′-difluoro ester PE-2, showing a polarity increase by separating the fluorines. For the trifluoromethyl pivalate ester PE-5 lipophilicity increases consistent with expectation for a –CF_3_ group particularly without any β-hydrogens. Notably the hydroxypivaloyl ester PE-6 is the most hydrophilic of the series, and even up to three fluorines does not outcompete a hydroxyl group.

In terms of p*K*_a_, all of the fluorinated pivalic acids (PAs) have an increased acidity as expected due to the electronegative fluorines, relative to pivalic acid (PA), however perhaps surprisingly the most acidic is the β,β′,β′-trifluoropivalic acid PA-3, which is more electron withdrawing than that with the –CF_3_ group in PA-5. Presumably the electron withdrawing power of three separated fluorines is stronger than the inductive effect of a –CF_3_ group. Similarly, the β,β′,-difluoropivalic acid PA-2 is more acidic than PA-4 with geminal fluorines in the –CF_2_H. Some similar relationships regarding p*K*_a_'s were also reported recently by Enamine Ltd.^13^

In order to explore the impact of the *tris*-fluoromethylated group relative to other – *tert*-butyl groups in a medicinal chemistry context, we selected to prepare analogues of the drugs elexacaftor 47 and tezacaftor 58.^36^ Both elexacaftor 47 and tezacaftor 58 are components of the combination therapy Trikafta®, used to treat cystic fibrosis.^37^ Trikafta® is a current blockbuster pharmaceutical, and is among the highest grossing pharmaceuticals by sales on the market presently. Elexacaftor 47 has a trifluoromethyl-containing *tert*-butyl group and thus a comparison with the target analogue 57 compares the effect of spreading these fluorines across the methyls of the *tert*-butyl group.

**Scheme 6:**
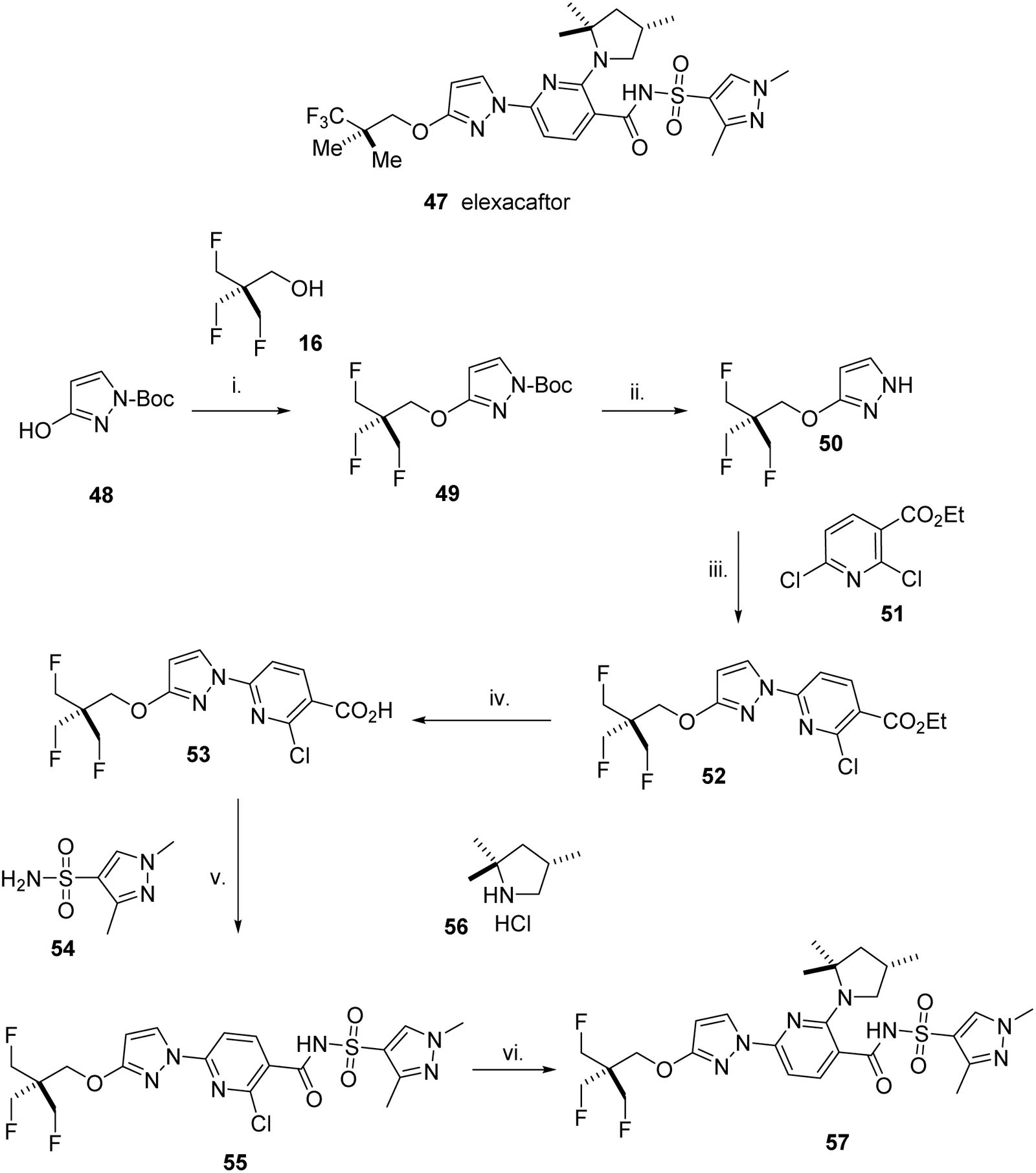
Synthetic route to elexacaftor analogue 57. Reagents and conditions: (i) 16 (1 eq.), DIAD (1.1 eq.), PPh_3_ (1.1 eq.), toluene, *Δ*, 16 h, 72%. (ii) HCl in dioxane (4 M), 45 °C, 30 min, quant. (iii) 51 (1 eq.), K_2_CO_3_ (1.5 eq.), DABCO (15 mol%), DMF, r.t., 24 h, 71%. (iv) NaOH (1 eq.), EtOH/THF/H_2_O (1 : 1 : 1), 40 °C, 2 h, 94%. (v) CDI (3 eq.), THF, 60 °C, 30 min, then 54 (1.5 eq.), DBU (3 eq.), THF, 10 min., quant. (vi) 56 (2.5 eq.), K_2_CO_3_ (5 eq.), DMSO/1,2-diethoxyethane (4 : 1), 120 °C, 16 h, 35%.

**Fig. 5:**
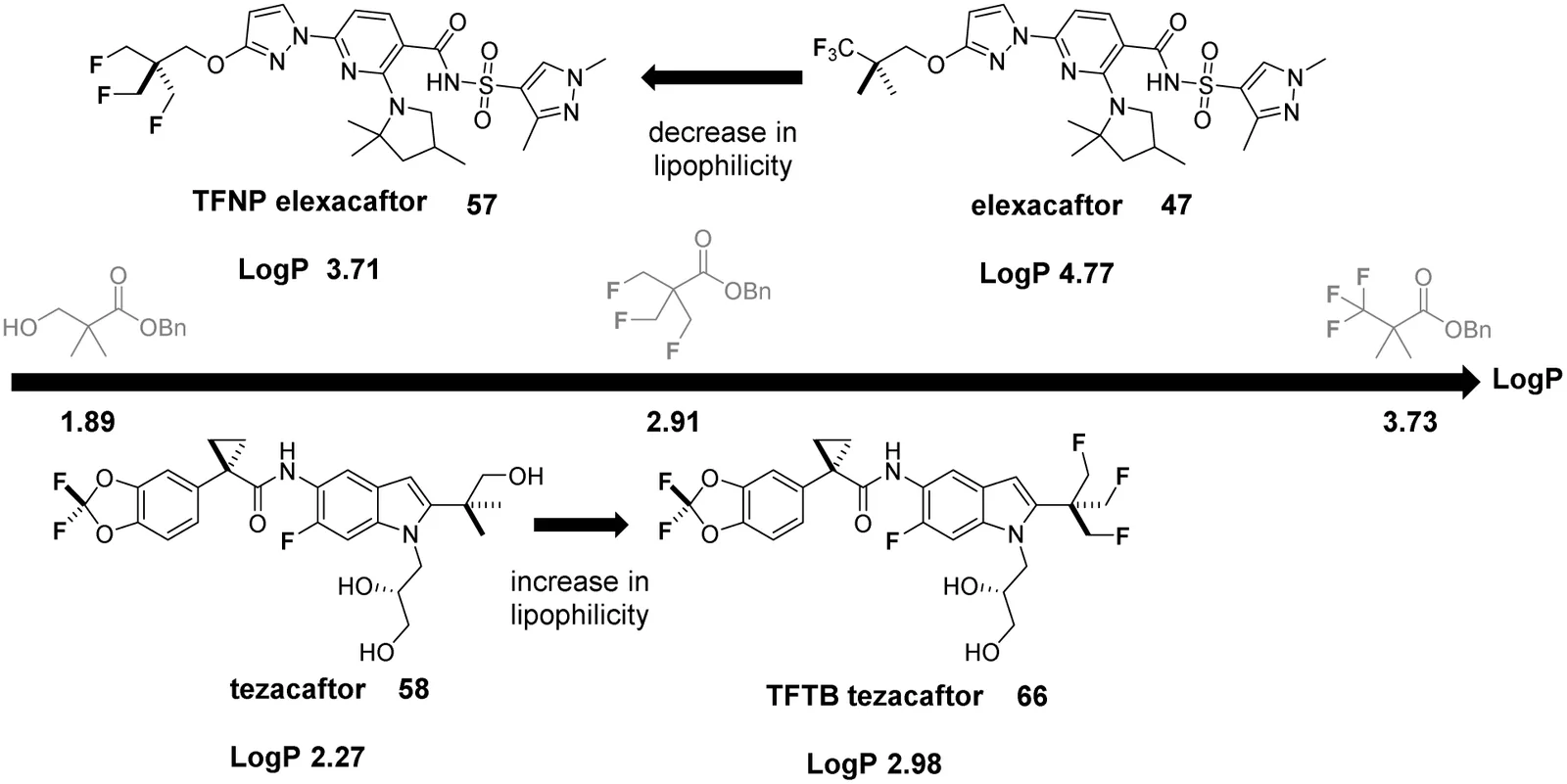
Log *P* comparisons of elexacaftor 47 and tezacaftor 66, with their *tris*-fluoromethylated *tert*-butyl analogues 57 and 68 respectively.

The synthesis of analogue 57 is illustrated in Scheme 6 and adapts previous protocols.^36^ The *tert*-butyl group was introduced through a Mitsunobu reaction^38^ between alcohol 16 and the Boc protected pyrazol-5-ol 48 to generate ether 49. NBoc deprotection followed by condensation of the resultant pyrazole 50 with pyridine dichloride 51 gave adduct 52, which was progressed to sulfonamide 55 after hydrolysis and condensation with sulfonamide 54. Finally, displacement of the chlorine of pyridylchloride 55 with pyrrolidine 56 generated target drug analogue 57. Elexacaftor 47 was similarly prepared as a reference compound starting from the appropriate trifluoromethyl containing alcohol. A comparison of the log *P*_s_,^35^ summarised in Fig. 5, indicated a reduction in log *P* (log *P* = 3.71) for 57 relative to that for elexacaftor 47 (log *P* = 4.77), showing an order of magnitude polarity increase by replacing the *tert*-butyl group containing CF_3_ with the (γ,γ′,γ′-trifluoro)neopentyl group, in this system.

**Scheme 7:**
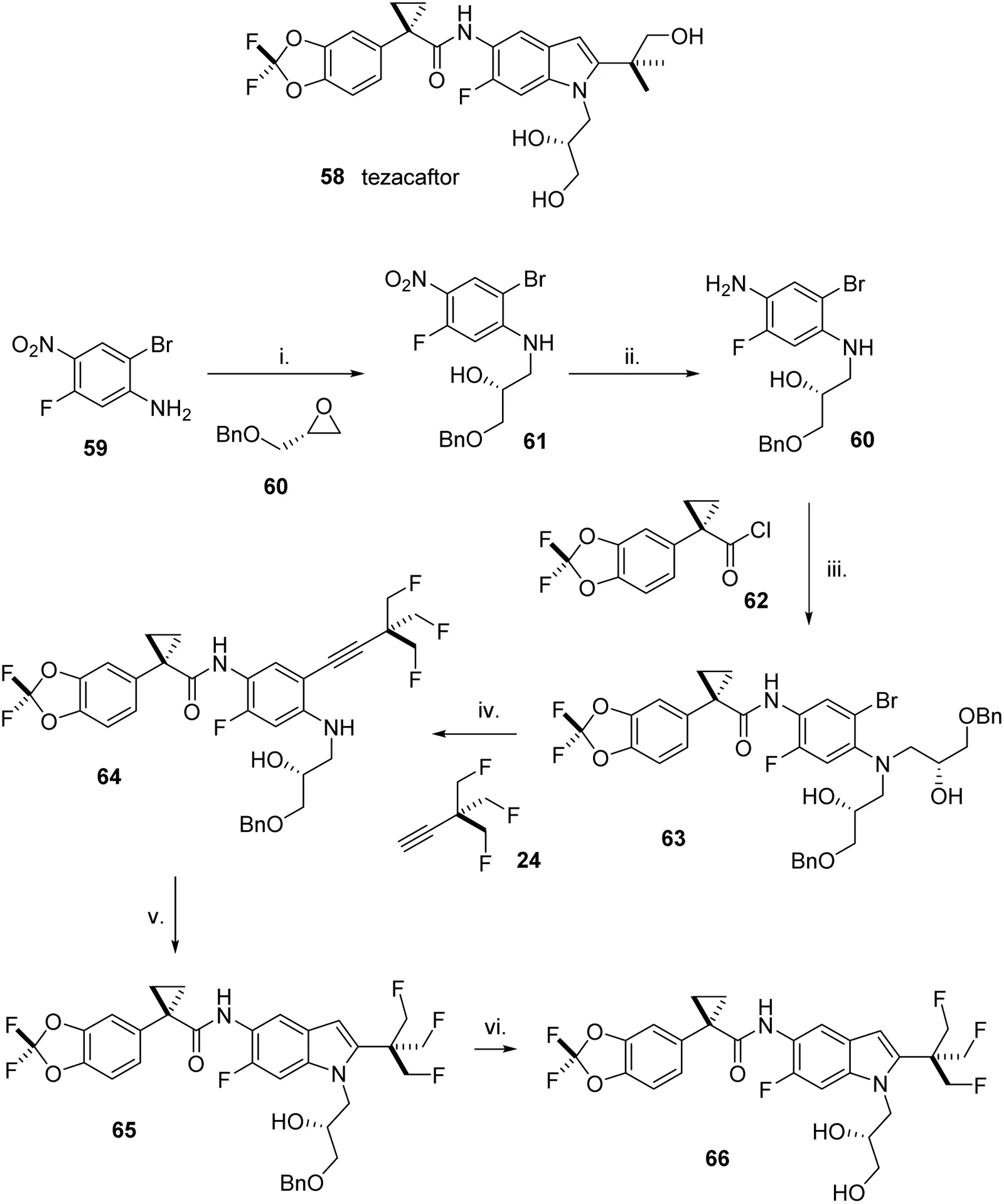
Synthetic route to tezacaftor analogue 66. Reagents and conditions: (i) (*R*)-epoxide 60 (2 eq.), Zn(ClO_4_)·6H_2_O (20 mol%), 4 Å MS, toluene, 80 °C, 16 h, 63%. (ii) H_2_ (10–15 bar), Pt/C (10% loading, 5% by wt), ^*i*^PrOAc, 16 h, 79%. (iii) Acid chloride 62 (1.1 eq), NEt_3_ (3.3 eq.), DCM, r.t., 16 h, 66%. (iv) Alkyne 24 (2 eq.), Pd(OAc)_2_ (4 mol%), XPhos (8 mol%), Cs_2_CO_3_ (2 eq.), MeOH, *Δ*, 16 h, 38%. (v) Pd(MeCN)_2_Cl_2_ (10 mol%), MeCN, *Δ*, 16 h, 30%. (vi) H_2_ (20 bar) Pd(OH)_2_/C (20% loading, cat), EtOAc, r.t.,16 h, 67%.

The analogue 66 replaces a hydroxylated *tert*-butyl group in tezacaftor 58 with the *tris*-fluoromethylated motif. The synthesis route is illustrated in Scheme 7 and is also adapted from published routes.^29^ A key reaction involved the Sonogashira cross coupling^19^ of alkyne 24 with aryl bromide 63 to generate acetylene 64, which was then a substrate for a Larock type protocol^19^ to generate indole 65. Hydrogenolysis of the benzyl ether then gave analogue 66. The log *P* comparison between tezacaftor 58 (log *P* = 2.3) and analogue 66 (log *P* = 3.00) illustrated in Fig. 5, indicated that the alcohol moiety retains a higher polarity over incorporation of three fluorines on the *tert*-butyl group. This trend is also consistent with that observed in Fig. 4 for PE-3*versus*PE-6.

## Conclusion

In summary we have prepared various new intermediates such as aldehyde 9, acetylene 24 and amino acid 32 which should enable the *tris*-fluoromethylated motif to be introduced into a greater variety of molecular structures through a diversity of chemical methodologies. This motif lowers log *P* relative to a *tert*-butyl group. We also demonstrate that the electron withdrawing power of three fluoromethyl groups is stronger than that of three fluorines in a –CF_3,_ all other structural aspects being equivalent. In order to demonstrate target accessibility we prepared analogues of two currently important active pharmaceutical ingredients replacing *tert*-butyl substituents with the *tris*-fluoromethylated motif to exemplify a potential utility in drug discovery programmes. During the study we were able to illustrate the steric impact of the *tris*-fluoromethylated motif by its influence on ^1^H-NMR chemical shifts progressing from mono- and di-fluoro analogues. We hope that this study will encourage the exploration of this motif into drug candidates as it introduces unique physiochemical properties which could play to advantage.

## Author contributions

JMS carried out the synthetic work and compiled the SI data, BAP carried out the theory work and processed and interpreted the data, DBC carried out the crystallography and processed the data, RAC led the theory work, interpreted and guided processing of the data and DOH led the overall project and the writing. All authors contributed to the writing of the manuscript.

## Conflicts of interest

There are no conflicts to declare.

## Supplementary Material

SC-017-D6SC04239B-s001

SC-017-D6SC04239B-s002

## Data Availability

The data supporting the computational research are available from the corresponding author (RAC) upon request. CCDC 2553089 contains the supplementary crystallographic data for this paper.^39^ Supplementary information (SI) is available. See DOI: https://doi.org/10.1039/d6sc04239b.
