# Supplementary material for: Incorporation and properties of the tris-fluoromethylated motif into heterocycles, amino acids and analogues of elexacaftor and tezacaftor
Source: Chem Sci. 2026 Jul 3. Online ahead of print. doi: 10.1039/d6sc04239b (PMC13348278; doi:10.1039/d6sc04239b)
Supplement: SC-OLF-D6SC04239B-s001 [file SC-OLF-D6SC04239B-s001.pdf]

Supplementary Information (SI) for Chemical Science.
This journal is © The Royal Society of Chemistry 2026

SUPPLEMENTARY INFORMATION

Incorporation and properties of the *tris*-fluoromethylated motif into heterocycles, amino acids and analogues of Elexacaftor and Tezacaftor

Josephine M. Stewart,[a] Bruno A. Piscelli,[b] David B. Cordes,[a] Rodrigo A. Cormanich*[b] and David O'Hagan*[a]

[a]EaStChem School of Chemistry, University of St Andrews, North Haugh, St Andrews, KY16 9ST, UK.

Corresponding author e.mail: do1@st-andrews.ac.uk

[b]Universidade Estadual de Campinas (UNICAMP), Instituto de Química, Monteiro Lobato Street, Campinas, Sao Paulo 13083-862, Brazil. Corresponding author e.mail: cormanich@unicamp.br

Table of Contents

General experimental 2

Synthesis 4

General procedures 4

Synthesis and characterisation 7

Chiral HPLC 44

LogP measurements 48

pK$_a$ measurements 152

Crystallography 173

General experimental

All reactions carried out under air in standard laboratory glassware unless otherwise stated. Commercially available solvents were purchased from Arcos, Alfa Aesar, Fisher Scientific, Fluorochem, Sigma Aldrich and TCI and used as received unless otherwise stated. Dry solvents (toluene, THF, hexane, diethyl ether and DCM) were dispensed from an MBraun SPS-800 solvent system. Commercially available reagents purchased from Sigma Aldrich, BLD Pharm, Fluorochem, Fisher Scientific and Apollo Scientific. Room temperature refers to the temperature range of 15-25 °C. Under reduced pressure refers to use of a rotary evaporator with membrane pump at 30-50 mbar. Microwave reactions were performed in a Biotage Initiator + microwave reactor.

Flash column chromatography was performed with Sigma-Aldrich silica gel, 60 Å pore size and 230-400 mesh, 40-63 μm particle size under 5 psi compressed air. Columns performed both manually and on a Biotage Selekt 2 system with a UV/vis detector (200-400 nm), cartridge sizes from 10 g to 100 g. Analytical TLC was performed on pre-coated aluminium plates (Kieselgel 60 F254 silica) and visualisation achieved with UV light (254 nm) and/or staining with aqueous $KMnO_4$ solution and heating.

Semi-prep and analytical HPLC purifications and analyses were performed using a Shimadzu Prominence (SIL-20A HT autosampler, CL-20AT ternary pump, DGU-20A3R solvent degasser, SPD 20A UV detector and CBM-20A controller module) equipped with a Synergi 4 μM Polar-RP 80 Å LC column (250 x 10 mm) or Kinetix 2.6 μM C18 100 Å LC column (150 x 4.6 mm). Prep-HPLC purifications were performed using a Shimadzu Prominence (SIL-20A HT autosampler, two LC-20AR pumps and an SPD-M20A diode array detector) equipped with a Synergi 4 μm Polar-RP 80 Å LC column (250 x 21.2 mm). Chiral HPLC analysis was obtained on a Shimadzu HPLC comprising of a DGU-20A5R degassing unit, LC-20AD liquid chromatography pump, SIL-20AHT autosampler, SPD-20A UV/Vis detector and a CTO-20A column oven. Separation was achieved using a Daicel Chiralcel OD-H column.

Samples were freeze dried from frozen solutions in water in a Christ Alpha 1-2 LO Plus freeze drier.

NMR spectra were recorded on Bruker AVIII 500, AVIII-HD 500, AVII 400, AV 400 and Neo 400 spectrometers. NMR analyses were carried out at room temperature in indicated deuterated solvents. Chemical shift data are reported as δ in units of ppm relative to the respective deuterated solvent. Coupling constants *J* are reported in Hz. ^{1}H spectra recorded at 400 and

500 MHz, $^{13}C\{^{1}H\}$ recorded at 101, 126 and 176 MHz and $^{19}F\{^{1}H\}$ spectra were recorded at 377 and 470 MHz. Structural assignments made with additional information from COSY, ^{1}H-^{13}C HSQC, ^{1}H-^{13}C and ^{1}H-^{19}F HMBC spectra, reported when relevant. Multiplicities are denoted as s (singlet), br s (broad singlet), d (doublet), t (triplet), q (quartet) and m (multiplet). Ar denotes an aromatic, 4ry denotes quaternary in ^{13}C NMR.

High resolution mass spectra were recorded on a Thermo Scientific Exactive orbitrap mass spectrometer owned by the University of St Andrews, UK. Spectra acquired by electrospray ionisation (ESI).

Optical rotation was performed using a Perkin Elmer Model 341 polarimeter, and values were recorded at 20 °C and 589 nm using a 1 dm cell, *c* in g/100 mL; $[\alpha]^{20}_{D}$ values are reported in units of 10^{-1} deg.cm^{2}.g^{-1}.

X-ray analysis of single crystals was conducted by Dr David Cordes at the University of St Andrews.

Synthesis

General procedures

Two-step triflation/fluorination (general procedure A)

To a solution of alcohol (1 eq) in dry DCM under N_2 atmosphere was added dry pyridine (2 eq per alcohol) and cooled to -40 °C. Triflic anhydride (1.5 eq per alcohol) was added dropwise over 30 min, once addition was complete solution was stirred at -40 °C for 30 min before being warmed to room temperature and allowed to stir for 2h. On completion of the reaction, mixture was diluted with DCM and washed with 2M HCl and brine (2 x), dried over $MgSO_4$ and concentrated to afford the crude triflate. This was then immediately taken up in dry THF, placed under N_2 atmosphere and cooled to 0 °C. TBAF (1M in THF, 1.1 eq per alcohol) was added *via* dropping funnel over 1h. The resulting solution was warmed to room temperature and stirred for 2h, monitoring by ^{19}F NMR. On completion of the reaction, the mixture was concentrated and partitioned between DCM and brine. Organic layer was washed with brine, dried over $MgSO_4$ and concentrated. Products were purified by silica gel column chromatography.

PCC oxidation of alcohols to aldehydes (general procedure B)

To a solution of alcohol (1 eq) in dry DCM was added 4Å molecular sieves (1 w/w) and PCC (1.5 eq). Mixture was allowed to stir for 3h, monitoring by ^{19}F NMR. On completion, the mixture was filtered through a silica plug washing with DCM. DCM was removed *via* distillation (short path, 55 °C, atmospheric pressure), to furnish the aldehyde product.

Cyclisation of diamines with aldehydes (general procedure C)

To a solution of diamine and $NaHSO_3$ in DMA at 100 °C, was added a solution of aldehyde in DMA (1 mL) dropwise over 10 min. After 2h the mixture was quenched and further purified.

Horner-Wadsworth Emmons reaction (general procedure D)

To a suspension of NaH (60% dispersion in mineral oil, 1.3 eq) in THF (10 mL) at 0 °C under N_2 atmosphere was added diethyl (2-oxo-2-phenylethyl)phosphonate (1.1 eq) and allowed to stir for 30 min. To this was added a solution of aldehyde (1 eq) in THF (1 mL) dropwise. On complete of addition, mixture was allowed to warm to room temperature and stirred until complete consumption of starting material (3h-6h), monitored by ^{19}F NMR. Mixture was quenched by addition of sat. NH_4Cl solution and extracted with EtOAc (3 x). Combined

organic layers were washed with brine, dried over $MgSO_4$ and concentrated to afford the crude product. Purified by silica gel column chromatography.

Asymmetric hydrogenation of α,β-unsaturated ketones (general procedure E)

Following a literature procedure,[1] [$RuCl_2${(*R*)-tol-BINAP}{(*S*)-dmapen}] (0.1 mol%) and ketone **24** (1 eq) were placed in a stainless-steel autoclave and air present was replaced with nitrogen. A solution of ^{t}BuOK (10 mmoldm^{-3} in ^{i}PrOH, 0.5 mol%) in ^{i}PrOH (5 v) which had been degassed by three freeze-pump-thaw cycles was added to the precooled autoclave under a stream of nitrogen. The nitrogen inside the autoclave was replaced with hydrogen and pressurised to 8-15 bar. The mixture was stirred vigorously at 0 °C overnight. H_2 gas was evacuated and solvent was removed under reduced pressure to afford the crude product. Purified by silica gel column chromatography.

Overman rearrangement (general procedure F)

Following a modified literature procedure,[2] to a solution of allylic alcohol (1 eq) in dry Et_2O under nitrogen atmosphere was added DBU (0.5 eq) and cooled to 0 °C. To this was added trichloroacetonitrile (3 eq) dropwise, and solution was allowed to warm to room temperature and stir overnight, monitored by ^{19}F NMR. On completion, mixture was filtered through a short silica plug, washing with Et_2O (3 x). The filtrate was concentrated and immediately taken up in dry toluene and heated to reflux under nitrogen atmosphere for 24h. Solvent was removed and residue purified by silica gel column chromatography.

Two-step ruthenium oxidation and deprotection (general procedure G)

To a solution of allylic amide (1 eq) in 1:1.5:1 MeCN:H_2O:EtOAc was added $NaIO_4$ (4 eq) and stirred until completely dissolved. $RuCl_3$ (~1 mg, cat.) was added, and the mixture was stirred vigorously at room temperature for 3h. Mixture was diluted with EtOAc and washed with 2M HCl. Aqueous layer was extracted with EtOAc (3 x), combined organics were washed with $NaHSO_3$ and brine before drying over $MgSO_4$ and concentrating. Residue was taken up in EtOH, to this was added 5M NaOH solution (30 eq) and stirred at room temperature for 3h. EtOH was removed under reduced pressure and pH adjusted to 1 with 6M HCl. Aqueous phase was washed 3 x with EtOAc then concentrated under reduced pressure to afford the amino acid as an HCl salt. Purified by ion-exchange chromatography (DOWEX 50WX8 hydrogen form, eluting with 0.5M NH_4OH) and lyophilised to give the ammonium salt of the product.

Benzylation of carboxylic acids (general procedure H)

To a solution of carboxylic acid (1 eq) in DMF (80 mL) was added K_2CO_3 (1.2 eq) and allowed to stir for 30 min at room temperature. Benzyl bromide (1 eq) was added, and solution was heated to 70 °C overnight. Mixture was quenched by addition of water and extracted with Et_2O (3 x). Organics were washed with brine, dried over $MgSO_4$ and concentrated. Purified by silica gel column chromatography (20-40% EtOAc in hexane) to afford the product.

Hydrogenation of benzyl esters (general procedure I)

To a solution of benzyl ester (1 eq) in EtOAc was added Pd/C (5-10% by wt) portion-wise. The mixture was sealed in a stainless-steel autoclave, pressurised to 15 bar and stirred vigorously overnight. Autoclave was depressurised and mixture was filtered through celite washing with EtOAc (3 x 20 mL). Solvent was carefully removed to afford the product.

Reduction of carboxylic acids to alcohols with $LiAlH_4$ (general procedure J)

To a stirring solution of $LiAlH_4$ (4 eq) in dry Et_2O at 0 °C under a blanket of nitrogen was added the carboxylic acid (1 eq) in Et_2O dropwise over 30 min. On complete addition mixture was allowed to warm to room temperature and stir for 4h. Mixture was cooled to 0 °C and slowly quenched by addition of water, followed by saturated KOH solution and water, this mixture was stirred for a further 1h before being filtered through celite, washing with Et_2O (3 x), dried over $MgSO_4$ and **carefully** concentrated to afford the product.

Synthesis and characterisation

4-(Hydroxymethyl)-1-methyl-2,6,7-trioxabicyclo[2.2.2]octane (11)

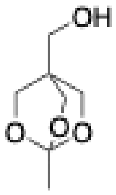

Following a literature procedure,[3] to a suspension of pentaerythritol **10** (12 g, 88.2 mmol) and *p*-TsOH (168 mg, 0.88 mmol) in hot dry toluene (150 mL) was added triethyl orthoacetate (16.3 mL, 88.2 mmol). The resulting mixture was allowed to stir at reflux until solution appeared clear (48-72h). A few drops of triethylamine were added, and the solution was hot filtered. Filtrate was concentrated under reduced pressure to afford **11** as white crystals (11.3 g, 80%).

^{1}H NMR (400 MHz, DMSO) δ 4.79 (t, J = 5.3 Hz, 1H, O**H**), 3.85 (s, 6H, 3 x C**H**$_2$O), 3.22 (d, J = 5.3 Hz, 2H, C**H**$_2$OH), 1.28 (s, 3H, C**H**$_3$). Data in accordance with literature.[4]

2-((Benzyloxy)methyl)-2-(hydroxymethyl)propane-1,3-diol (13)

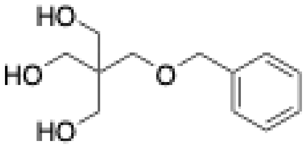

Following a literature procedure,[3] to a solution of **11** (8.8 g, 55 mmol) in refluxing dry toluene (150 mL) was added sodium hydride (3.3 g, 82.5 mmol) portion-wise and allowed to stir for 30 min. To this mixture was added benzyl bromide (6.5 mL, 60.5 mmol) and allowed to stir at reflux for 16h. Reaction mixture was cooled to room temperature before being filtered. The filtrate was concentrated to afford crude **12** which was taken up in methanol (50 mL). To this was added a few drops of HCl (conc.) and heated to 40 °C for 2h. The pH was made basic with NaOH before solution was filtered and concentrated. Crude residue subjected to column chromatography (50-100% EtOAc in hexane) to afford to **13** as a white crystalline solid (8.3 g, 67% over two steps).

^{1}H NMR (500 MHz, DMSO) δ 7.39 – 7.17 (m, 5H, Ar**H**), 4.44 (s, 2H, OC**H**$_2$Ar), 4.22 (t, J = 5.3 Hz, 3H, 3 x O**H**), 3.40 (d, J = 5.3 Hz, 6H, 3 x C**H**$_2$OH), 3.37 (s, 2H, C**H**$_2$O). Data in accordance with literature.[5]

TFNP benzyl ether (15)

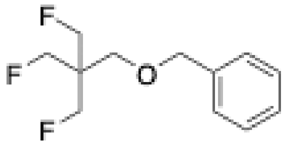

Prepared according to general procedure A, from **13** (5.2 g, 23 mmol), dry pyridine (11.1 mL, 138 mmol), triflic anhydride (17.2 mL, 103.5 mmol). Then TBAF (1M in THF, 76 mL). Purified *via* silica gel column chromatography (0-5% EtOAc in hexane), to afford **15** as a yellow oil (4.8 g, 90% over two steps).

^{1}H NMR (400 MHz, $CDCl_3$) δ 7.42 – 7.27 (m, 5H, Ar**H**), 4.56 (dt, *J* = 47.0, 1.4 Hz, 3H, 3 x C**H**$_2$F), 4.53 (s, 1H, OC**H**$_2$Ar), 3.56 (q, *J* = 1.6 Hz, 1H, C**H**$_2$O).

^{19}F{^{1}H} NMR (470 MHz, $CDCl_3$) δ -238.7.

^{13}C{^{1}H} NMR (126 MHz, $CDCl_3$) δ 137.9 (4ry Ar**C**), 128.6 (Ar**C**H), 127.9 (Ar**C**H), 127.6 (Ar**C**H), 81.4 (dt, *J* = 171.7, 5.7 Hz, 3 x **C**H$_2$F), 73.7 (O**C**H$_2$Ar), 66.5 (q, *J* = 5.0 Hz, **C**H$_2$O), 46.3 (q, *J* = 16.6 Hz, 4ry **C**(CH$_2$F)$_3$.

HRMS (ESI) m/z $[C_{12}H_{15}OF_3Na]^+$ requires [M] 255.0967, found 255.0967.

TFNP alcohol (16)

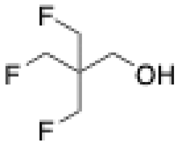

To a solution of **15** (4.5 g, 19.3 mmol) in EtOAc (20 mL) was added $Pd(OH)_2/C$ (50 mg) and sealed in a stainless-steel autoclave. This was filled and purged thrice with H_2 before being pressurised to 10 bar and stirred vigorously at room temperature overnight. The autoclave was depressurised and the resulting mixture was filtered through celite washing with EtOAc (3 x 10 mL) and **carefully** concentrated to afford the product **16** as a volatile white crystalline solid (2.48 g, 90%), Mp 103-104°C.

^{1}H NMR (400 MHz, $CDCl_3$) δ 4.55 (dt, *J* = 46.9, 1.3 Hz, 6H, 3 x C**H**$_2$F), 3.81 – 3.76 (m, 2H, C**H**$_2$OH).

^{19}F{^{1}H} NMR (377 MHz, $CDCl_3$) δ -239.1.

^{13}C{^{1}H} NMR (176 MHz, $CDCl_3$) δ 81.4 (dt, *J* = 171.0, 5.7 Hz, **C**H$_2$F), 60.3 (q, *J* = 5.7 Hz, **C**H$_2$OH), 46.7 (q, *J* = 16.0 Hz, 4ry **C**).

HRMS (ESI) m/z $[C_5H_8F_4O]^-$ requires [M] 161.0595, found 161.0596.

TFNP aldehyde (9)

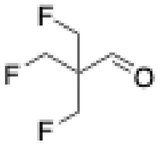

Prepared according to general procedure B, from **71** (2.0 g, 14.1 mmol) and PCC (4.6 g, 21.2 mmol) with 4Å molecular sieves (500 mg). Gave aldehyde **216** as a volatile yellow oil (1.38 g, 70%).

^{1}H NMR (400 MHz, $CDCl_3$) δ 9.84 (q, *J* = 2.1 Hz, 1H, C**H**O), 4.76 (dt, *J* = 46.5, 1.3 Hz, 6H, 3 x C**H**$_2$F).

^{19}F{^{1}H} NMR (470 MHz, $CDCl_3$) δ -238.3.

^{13}C{^{1}H} NMR (126 MHz, $CDCl_3$) δ 198.9 (q, *J* = 4.5 Hz, **C**HO), 79.6 (dt, *J* = 173.9, 6.1 Hz, **C**H$_2$F), 62.3 (q, *J* = 5.4 Hz, 4ry **C**).

HRMS (ESI) m/z $[(C_5H_7OF_3)Na]^+$ requires [M] 303.0790, found 303.0791.*

*Mass of dimer + Na^+

1-(1,3-Difluoro-2-(fluoromethyl)propan-2-yl)-2,3,4,9-tetrahydro-1*H*-pyrido[3,4-*b*]indole (18)

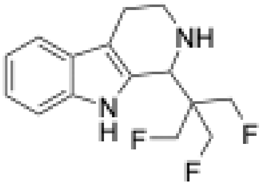

Following a literature procedure,[6] tryptamine **17** (100 mg, 0.62 mmol), aldehyde **9** (87 mg, 0.62 mmol) and T3P® (50% w/v in EtOAc, 1 mL) were combined in a microwave vial, sealed and heated to 120 °C in a microwave reactor for 30 min. Residue was diluted with EtOAc (5 mL), washed with $NaHCO_3$ (2 mL x 2) and brine (2 mL). The organic layer was dried and concentrated, before purifying by silica gel column chromatography (20-30% EtOAc in hexane) to afford **18** as a brown crystalline solid (60 mg, 34%).

^{1}H NMR (500 MHz, $CDCl_3$) δ 8.34 (br s, 1H, indole N**H**), 7.51 (d, *J* = 7.8 Hz, 1H, Ar**H**), 7.34 (dt, *J* = 8.1, 1.0 Hz, 1H, Ar**H**), 7.19 (tt, *J* = 8.2, 1.2 Hz, 1H, Ar**H**), 7.11 (ddd, *J* = 8.0, 7.0, 1.1 Hz, 1H, Ar**H**), 4.79 (ddt, *J* = 47.1, 9.8, 1.4 Hz, 3H, 3 x diastereotopic CH**H**F), 4.73 (ddt, *J* = 47.5, 9.8, 1.4 Hz, 3H, 3 x diastereotopic C**H**HF), 4.48 (s, 1H, C**H**NH) 3.19 (dt, *J* = 12.6, 5.1

Hz, 1H, diastereotopic C**H**HNH), 3.01 (ddd, *J* = 12.4, 7.3, 4.9 Hz, 1H, diastereotopic CH**H**NH), 2.73 (m, 2H, ArCH_2CH$_2$), 1.86 (br s, N**H**).

^{19}F{^{1}H} NMR (470 MHz, CDCl$_3$) δ -233.4.

^{13}C{^{1}H} NMR (126 MHz, CDCl$_3$) δ 136.0 (4ry Ar**C**NH), 129.9 (4ry Ar**C**NH), 127.2 (4ry Ar**C**), 122.3 (Ar**C**H), 119.6 (Ar**C**H), 118.3 (Ar**C**H), 112.4 (4ry Ar**C**) 111.1 (Ar**C**H), 83.9 (dt, *J* = 170.8, 6.1 Hz, 3 x **C**H$_2$F), 53.7 (q, *J* = 3.6 Hz, **C**HNH), 48.7 (q, *J* = 15.7 Hz, 4ry **C**(CH$_2$F)$_3$), 42.7 (**C**H$_2$), 22.5 (**C**H$_2$).

HRMS (ESI) m/z [$C_{15}H_{18}N_2F_3$]$^+$ requires [M] 283.1417, found 283.1415.

2-(1,3-Difluoro-2-(fluoromethyl)propan-2-yl)-1*H*-benzo[*d*]imidazole (20)

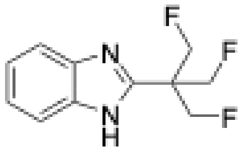

Prepared according to general procedure C, from *o*-phenylenediamine **19** (108 mg, 1.0 mmol), NaHSO$_3$ (104 mg, 1.0 mmol) and aldehyde **9** (140 mg, 1.0 mmol). After 2h the mixture was diluted with water (10 mL), and filtered, washing with water (50 mL). Solid was dissolved in EtOAc, dried over MgSO$_4$ and concentrated to afford **20** as a white solid (56 mg, 35%).

^{1}H NMR (500 MHz, DMSO) δ 12.62 (br s, 1H, N**H**), 7.57 (br s, 2H, Ar**H**), 7.20 (br s, 2H, Ar**H**), 4.96 (d, *J* = 46.8 Hz, 6H, 3 x C$\mathbf{H}_2$F).

^{19}F{^{1}H} NMR (470 MHz, DMSO) δ -232.2.

^{13}C{^{1}H} NMR (126 MHz, DMSO) δ 149.9 (4ry Ar**C**), 142.4 (4ry Ar**C**-N), 134.3 (4ry Ar**C**-N), 122.7 (Ar**C**H), 121.7 (Ar**C**H), 118.8 (Ar**C**H), 111.5 (Ar**C**H), 81.7 (dt, *J* = 172.6, 5.7 Hz, 3 x **C**H$_2$F), 47.7 (q, *J* = 17.7 Hz, 4ry **C**(CH$_2$F)$_3$).

HRMS (ESI) m/z [$C_{11}H_{11}N_2F_3Na$]$^+$ requires [M] 251.0767, found 251.0764.

6-Chloro-8-(1,3-difluoro-2-(fluoromethyl)propan-2-yl)-9*H*-purine (22)

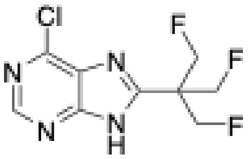

Prepared according to general procedure C, from diamine **21** (123 mg, 0.86 mmol), $NaHSO_3$ (89 mg, 0.86 mmol) and aldehyde **9** (120 mg, 0.86 mmol). Product purified by silica gel column chromatography (20-30% EtOAc in hexane) to give **22** as a white solid (46 mg, 20%).

^{1}H NMR (400 MHz, $CDCl_3$) δ 11.92 (br s, 1H, N**H**), 8.81 (s, 1H, Ar**H**), 5.06 (dt, *J* = 46.7, 1.6 Hz, 6H, 3 x C**H**$_2$F).

^{19}F{^{1}H} NMR (377 MHz, $CDCl_3$) δ -231.7.

^{13}C{^{1}H} NMR (126 MHz, DMSO) δ 154.3 (4ry Ar**C**),* 154.0 (4ry Ar**C**),* 152.0 (Ar**C**H), 150.0 (Ar**C**),* 81.1 (dt, *J* = 173.0, 5.7 Hz, 3 x **C**H$_2$F), 48.5 (q, *J* = 18.2 Hz, 4ry **C**(CH$_2$F)$_3$).†

*Determined from ^{1}H-^{13}C HMBC

† Not all carbon environments visible

HRMS (ESI) m/z [$C_9H_7N_4ClF_3$]$^-$ requires [M] 263.0317, found 263.0316.

4-Fluoro-2-(4-fluoro-3,3-bis(fluoromethyl)but-1-yn-1-yl)-5-nitroaniline (26)

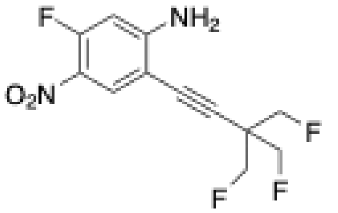

To a solution of aldehyde **9** (300 mg, 2.14 mmol) and Ohira-Bestmann reagent **23** (360 µL, 2.4 mmol) in MeOH (4 mL) at 0 °C was added Cs_2CO_3 (1.3 g, 4.0 mmol) portion-wise. The solution was allowed to warm to room temperature and stirred for 1h, monitored by ^{19}F NMR. On completion, the MeOH and resulting alkyne **24** were distilled off (100 °C, long path, atmospheric pressure) into a Schlenk flask. The resulting methanol solution was diluted with methanol (15 mL) and degassed *via* three freeze-pump-thaw cycles before placing it under a N_2 atmosphere. To this was added aryl bromide **25** (235 mg, 1 mmol) and caesium carbonate (650 mg, 2 mmol) and allowed to stir before adding Pd(OAc)$_2$ (9 mg, 0.04 mmol) and XPhos (38 mg, 0.08 mmol). Resulting mixture was heated to 60 °C until complete conversion of the alkyl bromide (12-24h), monitored by ^{19}F NMR. On completion, mixture was concentrated and purified by silica gel column chromatography (20-40% EtOAc in hexane) to afford **26** as a yellow solid (60 mg, 21%).*†

^{1}H NMR (500 MHz, $CDCl_3$) δ 8.14 (d, *J* = 8.2 Hz, 1H, Ar**H**), 6.44 (d, *J* = 12.6 Hz, 1H, Ar**H**), 5.03 (br s, 2H, N**H**$_2$), 4.63 (dt, *J* = 46.7, 1.4 Hz, 6H, 3 x C**H**$_2$F).

$^{19}F\{^1H\}$ NMR (470 MHz, $CDCl_3$) δ -111.8 (s, 1F, Ar**F**), -229.1 (s, 3F, 3 x CH_2**F**).

$^{13}C\{^1H\}$ NMR (126 MHz, $CDCl_3$) δ 158.1 (d, J = 265.7 Hz, 4ry Ar**C**F), 154.7 (d, J = 12.7 Hz, 4ry Ar**C**NH_2), 131.5 (Ar**C**H), 127.4 (Ar**C**NO_2),‡ 102.2 (d, J = 2.2 Hz, 4ry Ar**C**), 101.3 (d, J = 25.4 Hz, Ar**C**H), 90.1 (d, J = 8.2 Hz, alkyne **C**), 81.4 (dt, J = 179.8, 4.5 Hz, 3 x **C**H_2F), 80.1 (s, alkyne **C**), 44.7 (q, J = 18.5 Hz, 4ry **C**(CH_2F)$_3$).

HRMS (ESI) m/z $[C_{12}H_{10}O_2N_2F_4Na]^+$ requires [M] 313.0571, found 313.0569.

*NB S_NAr of methanol to yield methoxy derivative took up a significant amount of the yield, Sonogashira conversion is high.

† Inseparable XPhos impurity coeluted through column chromatography.

‡ Determined through ^1H-^{13}C HMBC

2-(1,3-Difluoro-2-(fluoromethyl)propan-2-yl)-6-fluoro-5-nitro-1*H*-indole (27)

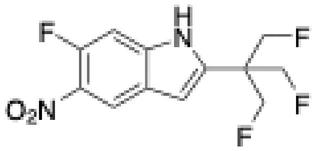

To a solution of aniline **26** (35 mg, 0.12 mmol) in MeCN (200 μL) was added Pd(MeCN)$_2$Cl$_2$ (13 mg, 0.05 mmol) and heated to reflux overnight. Solvent was removed under reduced pressure, and crude mixture purified by silica gel column chromatography (20-40% EtOAc in hexane) to give **27** as a pale-yellow solid (10 mg, 29%).

^1H NMR (400 MHz, $CDCl_3$) δ 8.85 (br s, 1H, N**H**), 8.39 (d, J = 7.1 Hz, 1H, Ar**H**), 7.21 (dd, J = 11.3, 0.7 Hz, 1H, Ar**H**), 6.62 (d, J = 1.9 Hz, 1H, Ar**H**), 4.83 (dt, J = 46.6, 1.5 Hz, 6H, 3 x C**H**$_2$F).

$^{19}F\{^1H\}$ NMR (377 MHz, $CDCl_3$) δ -124.4 (s, 1F, Ar**F**), -230.1 (s, 3F, CH_2**F**).

$^{13}C\{^1H\}$ NMR (126 MHz, $CDCl_3$) δ 153.1 (d, J = 256.1 Hz, Ar**C**F), 138.4 (d, J = 11.8 Hz, 4ry Ar**C**NH), 137.7 (br s, 4ry Ar**C**NH), 132.5 (4ry Ar**C**NO_2),* 123.0 (4ry Ar**C**), 119.6 (Ar**C**H), 103.4 (Ar**C**H), 99.6 (d, J = 25.9 Hz, Ar**C**H), 82.4 (dt, J = 177.1, 5.9 Hz, 3 x **C**H_2F), 46.6 (q, J = 18.5 Hz, 4ry **C**(CH_2F)$_3$).

*Determined from ^1H-^{13}C HMBC

HRMS (ESI) m/z $[C_{12}H_{10}O_2N_2F_4Na]^+$ requires [M] 313.0571, found 313.0569

(*E*)-5-Fluoro-4,4-bis(fluoromethyl)-1-phenylpent-2-en-1-one (28)

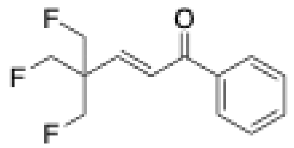

Prepared according to general procedure D, from aldehyde **9** (300 µL, 2.14 mmol), diethyl(2-oxo-2-phenylethyl)phosphonate (480 µL, 2.22 mmol) and NaH (60% dispersion in mineral oil, 110 mg, 2.85 mmol). Purified by silica gel column chromatography (0-5% EtOAc in hexane) to afford the product **28** as a yellow oil (280 mg, 58%).

^{1}H NMR (400 MHz, $CDCl_3$) δ 7.97 – 7.88 (m, 2H, Ar**H**), 7.64 – 7.55 (m, 1H, Ar**H**), 7.54 – 7.45 (m, 2H, Ar**H**), 7.17 (d, *J* = 16.2 Hz, 1H, CCH=C**H**CO), 6.93 (d, *J* = 16.2 Hz, 1H, CC**H**=CHCO), 4.63 (dt, *J* = 46.5, 1.5 Hz, 6H, 3 x C**H**$_2$F).

^{19}F{^{1}H} NMR (377 MHz, $CDCl_3$) δ -233.5.

^{13}C{^{1}H} NMR (126 MHz, $CDCl_3$) δ 189.7 (**C**=O), 141.2 (q, *J* = 3.9 Hz, **C**H=CHCO), 133.4 (4ry Ar**C**), 128.9 (2 x Ar**C**H), 128.8 (2 x Ar**C**H), 128.7 (q, *J* = 1.2 Hz, CH=**C**HCO), 81.8 (dt, *J* = 177.1, 5.4 Hz, 3 x **C**H$_2$F), 48.0 (q, *J* = 17.5 Hz, 4ry **C**).

HRMS (ESI) m/z $[C_{13}H_{13}OF_3Na]^+$ requires [M] 265.0811, found 265.0808.

[RuCl$_2${(*R*)-tol-BINAP}{(*S*)-dmapen}]

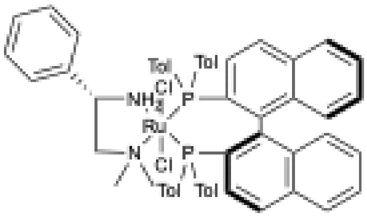

Prepared according to a literature procedure.[1]

(*R*,*E*)-5-Fluoro-4,4-bis(fluoromethyl)-1-phenylpent-2-en-1-ol (29)

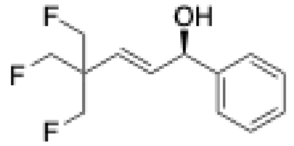

Prepared according to general procedure E, from ketone **28** (300 mg, 1.23 mmol), [RuCl$_2${(*R*)-tol-BINAP}{(*S*)-dmapen}] (~1 mg, 0.1 mol%), ^{t}BuOK (10 mmoldm^{-3} in ^{i}PrOH, 0.6 mL, 0.5 mol). Purified by silica gel column chromatography (10-20% EtOAc in hexane) to afford allylic alcohol (*R*)-**29** as a yellow oil (190 mg, 64%).

96% e.e. determined by chiral HPLC: Chiralcel OD-H, 2% ^{i}PrOH in hexane, 2 mL/min, 30 °C, detection at 211 nm (*R*)- 16.347 min, (*S*)- 23.421 min.

$[\alpha]^{20}_{D}$ +5.5° (*c* 0.55, $CHCl_3$);

^{1}H NMR (500 MHz, $CDCl_3$) δ 7.41 – 7.28 (m, 5H, Ar**H**), 5.96 (dd, *J* = 16.3, 5.7 Hz, 1H, CH=C**H**CHOH), 5.82 (d, *J* = 16.3 Hz, 1H, C**H**=CHCHOH), 5.24 (ddd, *J* = 5.4, 3.8, 1.3 Hz, 1H, C**H**OH), 4.52 (dt, *J* = 47.0, 1.4 Hz, 6H, 3 x C**H**$_2$F), 1.95 (d, *J* = 3.8 Hz, 1H, O**H**).

^{19}F{^{1}H} NMR (470 MHz, $CDCl_3$) δ -233.5.

^{13}C{^{1}H} NMR (126 MHz, $CDCl_3$) δ 142.3 (4ry Ar**C**), 136.3 (Ar**C**H), 128.9 (Ar**C**H), 128.2 (CH=**C**HCHOH), 126.5 (Ar**C**H), 125.3 (q, *J* = 3.8 Hz, **C**H=CHCHOH), 82.5 (dt, *J* = 175.7, 5.4 Hz, 3 x **C**H$_2$F), 74.9 (**C**HOH), 46.8 (q, *J* = 16.5 Hz, 4ry **C**(CH_2F)$_3$).

HRMS (ESI) m/z $[C_{13}H_{15}OF_3Na]^+$ requires [M] 267.0967, found 267.0967.

(*S*,*E*)-2,2,2-Trichloro-*N*-(5-fluoro-4,4-bis(fluoromethyl)-1-phenylpent-1-en-3-yl)acetamide (31)

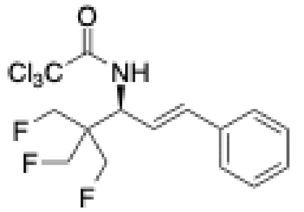

Prepared according to general procedure F, from allylic alcohol (*R*)-**29** (200 mg, 0.82 mmol) in dry Et_2O (15 mL), DBU (61 µL, 0.41 mmol), trichloroacetonitrile (247 µL, 2.46 mmol). Then heated in dry toluene (20 mL). Purified by silica gel column chromatography (0-5% EtOAc in hexane) to give (*S*)-**31** as a colourless oil (265 mg, 83%)

$[\alpha]^{20}_{D}$ -16° (*c* 0.4, $CHCl_3$).

^{1}H NMR (500 MHz, $CDCl_3$) δ 7.45 – 7.28 (m, 5H, Ar**H**), 6.75 (d, *J* = 15.6 Hz, 1H, CH=C**H**Ar), 6.18 (dd, *J* = 15.7, 8.2 Hz, 1H, C**H**=CHAr), 4.90 (ddd, *J* = 9.4, 8.2, 1.1 Hz, 1H, C**H**NH), 4.60 (d, *J* = 47.1 Hz, 6H, 3 x C**H**$_2$F).

^{19}F{^{1}H} NMR (470 MHz, $CDCl_3$) δ -235.9.

^{13}C{^{1}H} NMR (126 MHz, $CDCl_3$) δ 161.4 (**C**=O), 136.2 (CH=**C**HAr), 135.5 (4ry Ar**C**), 128.9 (Ar**C**H), 128.9 (**C**H=CHAr), 127.0 (Ar**C**H), 121.4 (Ar**C**H), 92.7 (**C**Cl$_3$), 80.9 (dt, *J* = 173.9, 6.4 Hz, 3 x **C**H$_2$F), 53.5 (q, *J* = 3.4 Hz, **C**HNH), 48.2 (q, *J* = 16.1 Hz, 4ry **C**(CH_2F)$_3$).

HRMS (ESI) m/z $[C_{15}H_{15}ON^{35}Cl_3F_3Na]^+$ requires [M] 410.0063, found 410.0058.

γ,γ',γ"-Trifluoro *tert*-leucine (32)

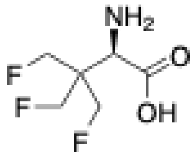

Prepared according to general procedure G, from allylic amide (*S*)-**31** (87 mg, 0.22 mmol), $NaIO_4$ (198 mg, 0.88 mmol), $RuCl_3$ (~1 mg) in MeCN:H_2O:EtOAc (1:1.5:1, 1.75 mL). Then taken up in EtOH (0.5 mL), with NaOH (5M, 1.2 mL) Purified by ion-exchange chromatography (DOWEX 50WX8 hydrogen form, eluting with 0.5M NH_4OH) and lyophilised to give (*R*)-**32** as an ammonium salt, white solid (29 mg, 72% over two steps).

$[\alpha]^{20}_D$ -2.8° (*c* 0.25, H_2O).

^{1}H NMR (500 MHz, D_2O) δ 4.74 (d, *J* = 47.5 Hz, 6H, 3 x C**H**$_2$F), 3.49 (s, 1H, C**H**NH).

^{19}F{^{1}H} NMR (470 MHz, D_2O) δ -235.1.

^{13}C{^{1}H} NMR (101 MHz, D_2O) δ 180.1 (**C**=O),† 82.0 (dt, *J* = 169.3, 6.9 Hz, **C**H$_2$F), 55.5 (q, *J* = 2.7 Hz, **C**HNH$_2$), 46.6 (m, 4ry **C**).†

HRMS (ESI) m/z $[C_6H_{11}O_2NF_3]^+$ requires 186.0536, found 186.0539.

*Coupling constant determined by ^{19}F-^{1}H HMBC due to solvent peak overlap with CH$_2$F peak.

†Determined by ^{1}H-^{13}C HMBC and HSQC

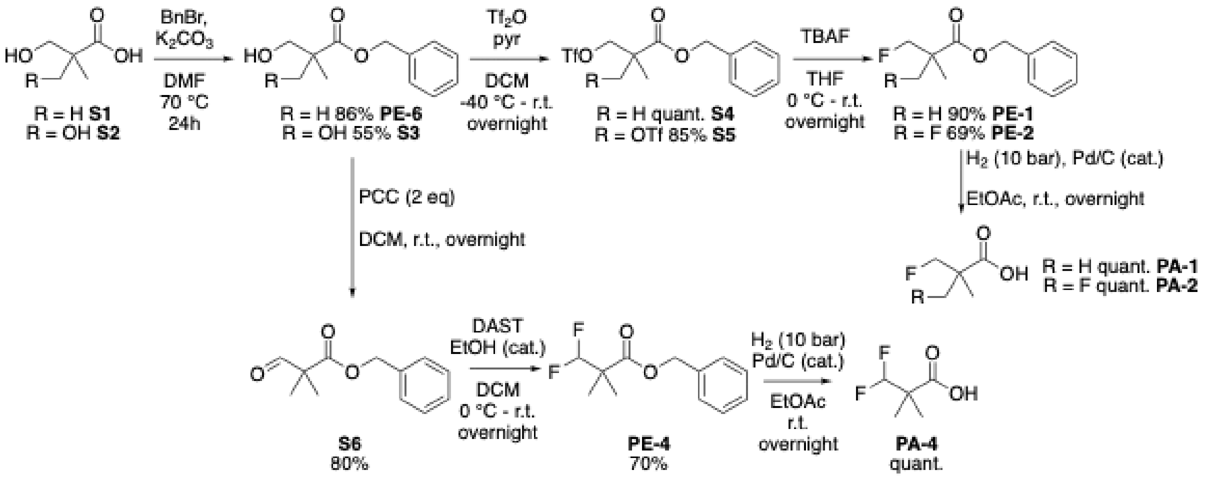

Scheme S1: Preparation of other fluorinated pivalic acid derivatives.

Benzyl hydroxypivalate (PE-6)

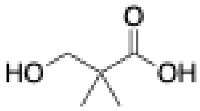

Prepared according to general procedure H, from hydroxypivalic acid **S1** (10 g, 84.5 mmol), K_2CO_3 (13 g, 101 mmol), benzyl bromide (9.5 mL, 84.5 mmol). Purified by silica gel column chromatography (20-40% EtOAc in hexane) to afford **PE-6** as a colourless oil (15.3 g, 87%).

^{1}H NMR (500 MHz, $CDCl_3$) δ 7.73 – 7.31 (m, 5H, Ar**H**), 5.15 (s, 2H, OC$\mathbf{H}_2$Ar), 3.58 (s, 2H, C$\mathbf{H}_2$OH), 1.22 (s, 6H, 2 x C$\mathbf{H}_3$).

Data in accordance with literature.[7]

Benzyl 2,2-bis(hydroxymethyl)propionate (S3)

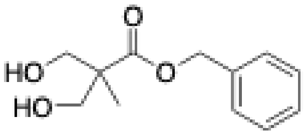

Prepared according to general procedure H, from 2,2-bis(hydroxymethyl)propionic acid **S2** (12 g, 89.6 mmol), K_2CO_3 (18. 4 g, 134 mmol), benzyl bromide (22.8 mL, 134 mmol). Purified by silica gel column chromatography (30-50% EtOAc in hexane) to give **S3** as a white crystalline solid (11 g, 55%).

^{1}H NMR (500 MHz, $CDCl_3$) δ 7.51 – 7.27 (m, 5H, Ar**H**), 5.21 (d, J = 2.0 Hz, 2H, OC$\mathbf{H}_2$Ar), 3.94 (dd, J = 11.3, 2.8 Hz, 2H, 2 x diastereotopic C**H**HOH), 3.74 (dd, J = 11.3, 2.2 Hz, 2H, 2 x diastereotopic CH**H**OH), 2.79 (br s, 2H, O**H**), 1.08 (s, 3H, C$\mathbf{H}_3$).

Data in accordance with literature.[8]

Benzyl fluoropivalate (PE-1)

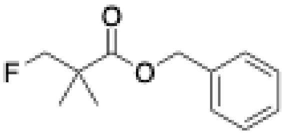

Prepared according to general procedure A, from **PE-6** (15 g, 72 mmol), pyridine (11.6 mL, 144 mmol) and triflic anhydride (18 mL, 108 mmol). Then TBAF (1M in THF, 86 mL). Purified by silica gel column chromatography (0-5% EtOAc in hexane) to give **PE-1** as a yellow oil (12.5 g, 83% over two steps).

^{1}H NMR (400 MHz, $CDCl_3$) δ 7.41 – 7.28 (m, 5H, Ar**H**), 5.16 (s, 2H, OC$\mathbf{H}_2$Ar), 4.43 (d, J = 47.1 Hz, 2H, C$\mathbf{H}_2$F), 1.26 (d, J = 1.7 Hz, 6H, 2 x C$\mathbf{H}_3$).

$^{19}F\{^1H\}$ NMR (377 MHz, $CDCl_3$) δ -222.1.

Data in accordance with literature.[9]

Benzyl 2,2-bis(fluoromethyl)propionate (PE-2)

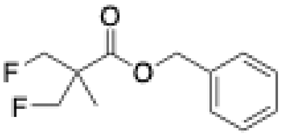

Prepared according to general procedure A, from **S3** (8.0 g, 35.5 mmol), pyridine (11.5 mL, 142 mmol), triflic anhydride (17.9 mL, 106 mmol). Then TBAF (1M in THF, 86 mL). Purified by silica gel column chromatography (0-5% EtOAc in hexane) to give **PE-2** as a yellow oil (3.6 g, 45% over two steps).

^1H NMR (400 MHz, $CDCl_3$) δ 7.57 – 7.29 (m, 5H, Ar**H**), 5.19 (s, 2H, OC**H**$_2$Ar), 4.68 (ddd, *J* = 46.8, 8.9, 1.2 Hz, 2H, 2 x diastereotopic C**H**HF), 4.53 (ddd, *J* = 46.7, 9.1, 1.2 Hz, 2H, 2 x diastereotopic CH**H**F), 1.25 (t, *J* = 1.7 Hz, 3H, C**H**$_3$).

$^{19}F\{^1H\}$ NMR (377 MHz, $CDCl_3$) δ -228.9.

$^{13}C\{^1H\}$ NMR (126 MHz, $CDCl_3$) δ 172.0 (**C**=O), 135.5 (4ry Ar**C**), 128.8 (Ar**C**H), 128.5 (Ar**C**H), 128.0 (Ar**C**H), 83.3 (dd, *J* = 173.9, 4.1 Hz, 2 x **C**H$_2$F), 67.0 (O**C**H$_2$Ar), 48.4 (t, *J* = 18.6 Hz, 4ry **C**(CH_3)(CH_2F)$_2$), 15.9 (t, *J* = 5.7 Hz, **C**H$_3$).

HRMS (ESI) m/z $[C_{12}H_{14}O_2F_2Na]^+$ requires [M] 251.0854, found 251.0851.

Fluoropivalic acid (PA-1)

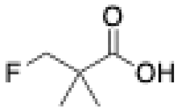

Prepared according to general procedure I, from **PE-1** (10 g, 47.6 mmol) and Pd/C (500 mg) pressurised to 15 bar H_2. Solvent was carefully removed to afford **PA-1** as a yellow solid (5.8 g, quant.).

^1H NMR (400 MHz, $CDCl_3$) δ 4.41 (d, *J* = 47.0 Hz, 2H, C**H**$_2$F), 1.27 (d, *J* = 1.6 Hz, 6H, 2 x C**H**$_3$).

$^{19}F\{^1H\}$ NMR (377 MHz, $CDCl_3$) δ -222.2.

Data in accordance with literature.[9]

2,2-Bis(fluoromethyl)propionic acid (PA-2)

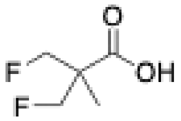

Prepared according to general procedure I, from **PE-2** (3.5 g, 15.3 mmol) and Pd/C (350 mg), pressurised to 15 bar H_2. Gave **PA-2** as a white crystalline solid (2.2 g, 15.9 mmol).

^{1}H NMR (400 MHz, $CDCl_3$) δ 4.66 (ddd, *J* = 46.8, 9.2, 1.5 Hz, 2H, 2 x diastereotopic C**H**HF), 4.52 (ddd, *J* = 46.8, 9.2, 1.1 Hz, 2H, 2 x diastereotopic CH**H**F), 1.29 (t, *J* = 1.7 Hz, 3H, C**H**$_3$).

^{19}F{^{1}H} NMR (377 MHz, $CDCl_3$) δ -229.1.

^{13}C{^{1}H} NMR (126 MHz, $CDCl_3$) δ 178.7 (t, *J* = 5.1 Hz, **C**=O), 83.0 (dd, *J* = 174.2, 4.3 Hz, 2 x **C**H_2F), 48.3 (t, *J* = 18.8 Hz, 4ry **C**), 15.8 (t, *J* = 5.7 Hz, **C**H_3).

HRMS (ESI) m/z $[C_5H_7O_2F_2]^+$ requires [M] 137.0420, found 137.0420.

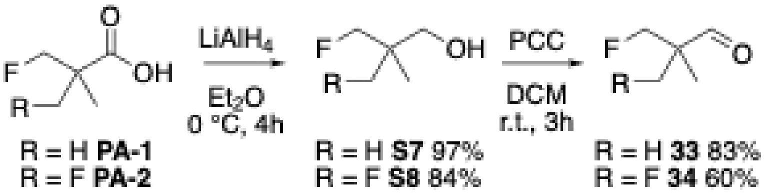

Scheme S2: Synthesis of aldehydes **33** and **34**.

Fluoroneopentyl alcohol (S7)

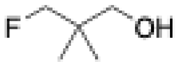

Prepared according to general procedure J, from $LiAlH_4$ (5.1 g, 134 mmol) and **PA-1** (4.0 g, 33.5 mmol). **Carefully** concentrated to afford the product **S7** as a volatile white solid (3.4 g, 97%).

^{1}H NMR (500 MHz, $CDCl_3$) δ 4.23 (d, *J* = 47.8 Hz, 2H, C**H**$_2$F), 3.47 (d, *J* = 1.3 Hz, 2H, C**H**$_2$OH), 0.93 (d, *J* = 1.8 Hz, 6H, 2 x C**H**$_3$).

^{19}F{^{1}H} NMR (470 MHz, $CDCl_3$) δ -226.5.

Data in accordance with literature.[10]

2,2-Bis(fluoromethyl)propyl alcohol (S8)

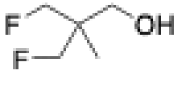

Prepared according to general procedure D, from **PA-2** (1.87 g, 13.5 mmol) and $LiAlH_4$ (2.2 g, 58 mmol). Gave **S8** as a volatile white crystalline solid (1.4 g, 84%).

^{1}H NMR (500 MHz, $CDCl_3$) δ 4.41 (ddd, *J* = 47.3, 11.3, 1.4 Hz, 2H, 2 x diastereotopic C**H**HF), 4.39 (ddd, *J* = 47.3, 11.4, 1.3 Hz, 2H, 2 x diastereotopic CH**H**F), 3.62 (t, *J* = 1.3 Hz, 2H, C**H**$_2$OH), 0.98 (t, *J* = 1.8 Hz, 3H, C**H**$_3$).

^{19}F{^{1}H} NMR (470 MHz, $CDCl_3$) δ -233.2.

^{13}C{^{1}H} NMR (126 MHz, $CDCl_3$) δ 85.3 (dd, *J* = 171.7, 5.0 Hz, **C**H$_2$F), 64.7 (t, *J* = 5.4 Hz, **C**H$_2$OH), 41.9 (t, *J* = 15.8 Hz, 4ry **C**), 14.8 (t, *J* = 5.4 Hz, **C**H$_3$).

HRMS (ESI) m/z $[C_5H_{10}OF_2Na]^+$ requires [M] 147.0592, found 147.0592.

Fluoropivaldehyde (33)

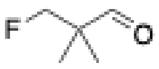

Prepared according to general procedure B, from alcohol **S7** (800 mg, 7.54 mmol) and PCC (3.2 g, 15.1 mmol) with 4Å MS (800 mg). Gave aldehyde **33** as a volatile yellow oil (652 mg, 83%).

^{1}H NMR (400 MHz, $CDCl_3$) δ 9.59 (d, *J* = 1.1 Hz, 1H, C**H**O), 4.41 (d, *J* = 47.3 Hz, 2H, C**H**$_2$F), 1.13 (d, *J* = 1.7 Hz, 6H, 2 x C**H**$_3$).

^{19}F{^{1}H} NMR (377 MHz, $CDCl_3$) δ -225.0.

^{13}C{^{1}H} NMR (126 MHz, $CDCl_3$) δ 181.8 (d, *J* = 3.2 Hz, **C**HO), 88.4 (d, *J* = 174.8 Hz, **C**H$_2$F), 43.7 (d, *J* = 18.6 Hz, 4ry **C**), 21.2 (d, *J* = 5.0 Hz, 2 x **C**H$_3$).

HRMS (ESI) $[(C_5H_9FO)_2Na]^+$ requires [M] 231.1167, found 231.1167.*

*Mass of dimer + Na^+

2,2-Bis(fluoromethyl)propanal (34)

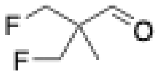

Prepared according to general procedure B, from alcohol **S8** (1.0 g, 8.2 mmol) and PCC (2.6 g, 12.2 mmol) with 4Å MS (1.0 g). Gave aldehyde **34** as a volatile yellow oil (600 mg, 60%).

^{1}H NMR (500 MHz, $CDCl_3$) δ 9.68 (t, *J* = 2.2 Hz, 1H, C**H**O), 4.64 (ddd, *J* = 47.0, 9.6, 1.5 Hz, 2H, 2 x diastereotopic C**H**HF), 4.57 (ddd, *J* = 46.9, 9.6, 0.8 Hz, 2H, 2 x diastereotopic CH**H**F), 1.16 (t, *J* = 1.7 Hz, 4H, C**H**$_3$).

$^{19}F\{^1H\}$ NMR (470 MHz, $CDCl_3$) δ -232.8.

$^{13}C\{^1H\}$ NMR (126 MHz, $CDCl_3$) δ 201.0 (t, *J* = 4.6 Hz, **C**HO), 82.7 (dd, *J* = 174.2, 4.8 Hz, **C**H_2F), 52.3 (t, *J* = 17.5 Hz, 4ry **C**), 12.9 (t, *J* = 5.7 Hz, **C**H_3).

HRMS (ESI) m/z $[(C_5H_8F_2O)Na]^+$ requires [M] 267.0979, found 267.0979.*

*Mass of dimer + Na^+

(*E*)-5-Fluoro-4,4-dimethyl-1-phenylpent-2-en-1-one (S9)

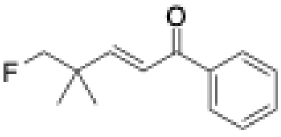

Prepared according to general procedure D, from aldehyde **33** (400 mg, 3.84 mmol), diethyl(2-oxo-2-phenylethyl)phosphonate (984 µg, 3.84 mmol) and NaH (60% dispersion in mineral oil, 192 mg, 4.99 mmol). Purified by silica gel column chromatography (0-10% EtOAc in hexane) to give **S9** as a yellow oil (350 mg, 44%).

^1H NMR (400 MHz, $CDCl_3$) δ 7.97 – 7.90 (m, 2H, Ar**H**), 7.62 – 7.53 (m, 1H, Ar**H**), 7.51 – 7.42 (m, 2H, Ar**H**), 7.00 (d, *J* = 15.9 Hz, 1H, CH=C**H**O), 6.90 (d, *J* = 15.8 Hz, 1H, C**H**=CHO), 4.26 (d, *J* = 47.6 Hz, 2H, C**H**$_2$F), 1.20 (d, *J* = 1.8 Hz, 6H, 2 x C**H**$_3$).

$^{19}F\{^1H\}$ NMR (377 MHz, $CDCl_3$) δ -221.6.

$^{13}C\{^1H\}$ NMR (126 MHz, $CDCl_3$) δ 191.2 (**C**=O), 153.4 (d, *J* = 3.6 Hz, **C**H=CHCO), 138.0 (4ry Ar**C**), 133.0 (Ar**C**H), 128.7 (Ar**C**H), 128.7 (Ar**C**H), 124.1 (CH=**C**HCO), 89.8 (d, *J* = 177.6 Hz, **C**H_2F), 38.9 (d, *J* = 17.5 Hz, 4ry **C**), 22.9 (d, *J* = 5.0 Hz, **C**H_3).

HRMS (ESI) m/z $[C_{13}H_{15}OFNa]^+$ requires [M] 229.0999, found 229.0996.

(*E*)-5-Fluoro-4-(fluoromethyl)-4-methyl-1-phenylpent-2-en-1-one (S10)

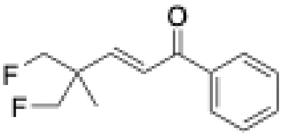

Prepared according to general procedure D, from aldehyde **34** (270 mg, 2.21 mmol), diethyl(2-oxo-2-phenylethyl)phosphonate (480 µL, 2.21 mmol) and NaH (60% dispersion in mineral oil, 111 mg, 2.87 mmol). Purified by silica gel column chromatography (0-10% EtOAc in hexane) to give **S10** as a yellow oil (120 mg, 24%).

^{1}H NMR (400 MHz, $CDCl_3$) δ 7.96 – 7.90 (m, 2H, Ar**H**), 7.61 – 7.56 (m, 1H, Ar**H**), 7.53 – 7.44 (m, 2H, Ar**H**), 7.03 (d, *J* = 16.0 Hz, 1H, CH=C**H**O), 6.94 (dt, *J* = 16.0, 0.6 Hz, 1H, C**H**=CHO), 4.46 (ddd, *J* = 47.2, 16.1, 1.5 Hz, 2H, 2 x diastereotopic C**H**HF), 4.43 (ddd, *J* = 47.2, 16.1, 1.5 Hz, 2H, 2 x diastereotopic CH**H**F), 1.23 (t, *J* = 1.9 Hz, 1H, C**H**$_3$).

$^{19}F\{^{1}H\}$ NMR (377 MHz, $CDCl_3$) δ -227.5.

$^{13}C\{^{1}H\}$ NMR (101 MHz, $CDCl_3$) δ 190.3 (**C**=O),* 147.0 (t, *J* = 4.3 Hz, **C**H=CHCO), 137.6 (4ry Ar**C**), 133.7 (Ar**C**H), 128.8 (Ar**C**H), 128.8 (Ar**C**H), 126.6 (CH=**C**HCO), 85.0 (dd, *J* = 177.5, 4.9 Hz, **C**H$_2$F), 43.7 (t, *J* = 14.7 Hz, 4ry **C**),* 17.3 (t, *J* = 4.9 Hz, **C**H$_3$).

*Determined from ^{1}H-^{13}C HMBC.

HRMS (ESI) m/z $[C_{13}H_{14}O_2F_2Na]^+$ requires [M] 247.0905, found 247.0902.

(*R*,*E*)-5-Fluoro-4,4-dimethyl-1-phenylpent-2-en-1-ol (37)

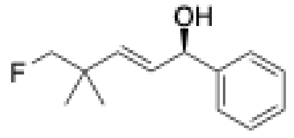

Prepared according to general procedure E, from ketone **S9** (190 mg, 0.92 mmol), [RuCl$_2${(*R*)-tol-BINAP}{(*S*)-dmapen}] (1 mg, 0.001 mmol), ^{t}BuOK in ^{i}PrOH (10 mmoldm^{-3}, 0.5 mL, 0.005 mmol). Purified by silica gel column chromatography (10-20% EtOAc in hexane) to afford allylic alcohol (*R*)-**37** as a colourless oil (164 mg, 86%).

96% e.e. determined by chiral HPLC: Chiralcel OD-H, 2% ^{i}PrOH in hexane, 2 mL/min, 30 °C, detection at 254 nm (*R*)- 7.099 min, (*S*)- 8.057 min.

$[\alpha]^{20}_{D}$ -2° (*c* 0.15, $CHCl_3$).

^{1}H NMR (500 MHz, $CDCl_3$) δ 7.40 – 7.27 (m, 5H, Ar**H**), 5.80 (dd, *J* = 15.8, 0.9 Hz, 1H, C**H**=CHOH), 5.71 (dd, *J* = 15.8, 6.3 Hz, 1H, CH=C**H**OH), 5.20 (ddd, *J* = 6.4, 3.7, 0.9 Hz, 1H, C**H**OH), 4.13 (d, *J* = 47.9 Hz, 2H, C**H**$_2$F), 1.92 (d, *J* = 3.7 Hz, 1H, O**H**), 1.08 (d, *J* = 1.7 Hz, 3H, diastereotopic C**H**$_3$), 1.07 (d, *J* = 1.7 Hz, 3H, diastereotopic C**H**$_3$).

$^{19}F\{^{1}H\}$ NMR (470 MHz, $CDCl_3$) δ -221.9.

$^{13}C\{^{1}H\}$ NMR (126 MHz, $CDCl_3$) δ 143.1 (4ry Ar**C**), 137.1 (d, *J* = 5.0 Hz, **C**H=CHCHOH), 131.0 (CH=**C**HCHOH), 128.7 (ArC**H**), 127.8 (Ar**C**H), 126.4 (Ar**C**H), 90.8 (d, *J* = 176.2 Hz, **C**H$_2$F), 37.6 (d, *J* = 17.7 Hz, **C**HOH), 23.87 – 22.84 (m, 2 x diastereotopic **C**H$_3$).

HRMS (ESI) m/z $[C_{13}H_{15}OFNa]^+$ requires [M] 229.0999, found 229.0998.

(*R*,*E*)-5-Fluoro-4-(fluoromethyl)-4-methyl-1-phenylpent-2-en-1-ol (38)

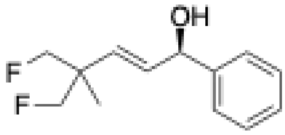

Prepared according to general procedure E, from, ketone **S10** (100 mg, 0.45 mmol), $[RuCl_2\{(R)$-tol-BINAP$\}\{(S)$-dmapen$\}]$ (~1 mg, 0.1 mol%), ^{t}BuOK in ^{i}PrOH (10 mmoldm^{-3}, 0.25 mL, 0.0025 mmol). Purified by silica gel column chromatography (10-20% EtOAc in hexane) to afford allylic alcohol (*R*)-**38** as a colourless oil (56 mg, 55%).

94% e.e. determined by chiral HPLC: Chiralcel OD-H, 2% ^{i}PrOH in hexane, 2 mL/min, 30 °C, detection at 211 nm, (*R*)- 10.929 min, (*S*)- 14.578 min.

$[\alpha]^{20}_D$ +6° (*c* 0.15, $CHCl_3$).

^{1}H NMR (500 MHz, $CDCl_3$) δ 7.40 – 7.27 (m, 5H, Ar**H**), 5.84 (dd, *J* = 16.0, 5.0 Hz, 1H, CH=C**H**CHOH), 5.80 (d, *J* = 16.1 Hz, 1H, C**H**=CHCHOH), 5.22 (dd, *J* = 4.9, 3.7 Hz, 1H, C**H**OH), 4.42 – 4.22 (m, 4H, 2 x C**H**$_2$F), 1.94 (d, *J* = 3.8 Hz, 1H, O**H**), 1.09 (t, *J* = 1.9 Hz, 3H, C**H**$_3$).

$^{19}F\{^1H\}$ NMR (470 MHz, $CDCl_3$) δ -227.2 (s, 1F, diastereotopic CH_2**F**), -227.2 (s, 1F, diastereotopic CH_2**F**).

$^{13}C\{^1H\}$ NMR (126 MHz, $CDCl_3$) δ 142.7 (4ry Ar**C**), 134.1 (CH=**C**HCHOH), 130.7 (t, *J* = 5.0 Hz, **C**H=CHCHOH), 128.8 (Ar**C**H), 128.0 (Ar**C**H), 126.4 (Ar**C**H), 85.7 (dd, *J* = 176.4, 4.3 Hz, 2 x **C**H$_2$F), 75.1 (**C**HOH), 42.1 (t, *J* = 18.3 Hz, 4ry **C**), 17.3 (t, *J* = 5.0 Hz, **C**H$_3$).

HRMS (ESI) m/z $[C_{13}H_{16}OF_2Na]^+$ requires [M] 249.1061, found 249.1060.

(*S*,*E*)-2,2,2-Trichloro-*N*-(5-fluoro-4,4-dimethyl-1-phenylpent-1-en-3-yl)acetamide (39)

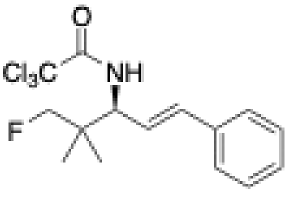

Prepared according to general procedure F, from allylic alcohol (*R*)-**37** (120 mg, 0.58 mmol), DBU (44 µL, 0.29 mmol) and trichloroacetonitrile (174 µL, 1.74 mmol). Then heated in dry toluene (10 mL). Purified by silica gel column chromatography (0-5% EtOAc in hexane) to give (*S*)-**39** as a viscous colourless oil (112 mg, 55%).

$[\alpha]^{20}_{D}$ +45° (*c* 1.05, $CHCl_3$).

^1H NMR (500 MHz, $CDCl_3$) δ 7.39 (dd, *J* = 7.0, 1.7 Hz, 2H, Ar**H**), 7.33 (t, *J* = 7.5 Hz, 2H, Ar**H**), 7.30 – 7.26 (m, 1H, Ar**H**), 6.66 (dd, *J* = 15.8, 1.2 Hz, 1H, CH=C**H**Ar), 6.19 (dd, *J* = 15.8, 7.5 Hz, 1H, C**H**=CHAr), 4.52 (t, *J* = 8.4 Hz, 1H, C**H**NH), 4.49 (dd, *J* = 48.2, 9.4 Hz, 1H, diastereotopic C**H**HF), 4.21 (dd, *J* = 47.1, 9.4 Hz, 1H, diastereotopic CH**H**F), 1.18 (s, 3H, diastereotopic C**H**$_3$), 1.00 (d, *J* = 2.5 Hz, 3H, diastereotopic C**H**$_3$).

$^{19}F\{^1H\}$ NMR (470 MHz, $CDCl_3$) δ -221.3.

$^{13}C\{^1H\}$ NMR (126 MHz, $CDCl_3$) δ 161.3 (**C**=O), 136.2 (4ry Ar**C**), 134.3 (CH=**C**HAr), 128.8 (Ar**C**H), 128.3 (Ar**C**H), 126.8 (Ar**C**H), 123.8 (**C**H=CHAr), 93.1 (**C**Cl_3), 89.9 (d, *J* = 171.2 Hz, **C**H_2F), 60.4 (**C**HNH), 39.1 (d, *J* = 15.9 Hz, 4ry **C**), 22.01 (d, *J* = 3.6 Hz, diastereotopic **C**H_3), 21.28 (d, *J* = 8.2 Hz, diastereotopic **C**H_3).

HRMS (ESI) m/z $[C_{15}H_{17}ONCl_3FNa]^+$ requires [M] 374.0250, found 374.0247.

(*S*,*E*)-2,2,2-Trichloro-*N*-(5-fluoro-4-(fluoromethyl)-4-methyl-1-phenylpent-1-en-3-yl)acetamide (40)

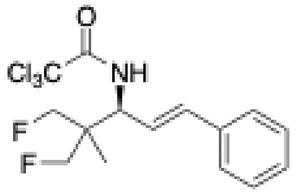

Prepared according to general procedure H, from allylic alcohol (*R*)-**38** (50 mg, 0.22 mmol), DBU (16 μL, 0.11 mmol), trichloroacetonitrile (66 μL, 0.66 mmol). Then heated in dry toluene (5 mL). Purified by silica gel column chromatography (0-5% EtOAc in hexane) to give (*S*)-**40** as a colourless oil (32 mg, 39%).

$[\alpha]^{20}_{D}$ +50° (*c* 0.05, $CHCl_3$).

^1H NMR (500 MHz, $CDCl_3$) δ 7.42 – 7.38 (m, 2H, Ar**H**), 7.37 – 7.32 (m, 2H, Ar**H**), 7.31 – 7.27 (m, 1H, Ar**H**), 6.70 (d, *J* = 15.8 Hz, 1H, CH=C**H**Ar), 6.18 (dd, *J* = 15.8, 7.8 Hz, 1H, C**H**=CHAr), 4.68 (t, *J* = 8.0 Hz, 1H, C**H**NH), 4.62 – 4.37 (m, 4H, C**H**$_2$F), 1.08 (t, *J* = 2.0 Hz, 3H, C**H**$_3$).

$^{19}F\{^1H\}$ NMR (470 MHz, $CDCl_3$) δ -226.0 (s, 1F, diastereotopic CH_2**F**), -232.1 (s, 1F, diastereotopic CH_2**F**).

$^{13}C\{^1H\}$ NMR (126 MHz, $CDCl_3$) δ 161.3 (**C**=O), 135.8 (4ry Ar**C**), 135.3 (CH=**C**HAr), 128.9 (Ar**C**H), 128.6 (Ar**C**H), 126.9 (Ar**C**H), 122.3 (**C**H=CHAr), 85.3 (dd, *J* = 171.9, 5.7 Hz, diastereotopic **C**H_2F), 85.1 (dd, *J* = 173.9, 4.1 Hz, diastereotopic **C**H_2F), 57.0 (dd, *J* = 3.5, 1.4 Hz, **C**HNH), 43.6 (t, *J* = 16.0 Hz, 4ry **C**), 15.8 (dd, *J* = 7.7, 5.0 Hz, **C**H_3).;*

*CCl_3 carbon environment not visible.

HRMS (ESI) m/z $[C_{15}H_{16}ONCl_3F_2Na]^+$ requires [M] 392.0158, found 392.0153.

*CCl_3 carbon environment not visible.

D-γ-Fluoro *tert*-leucine (41)

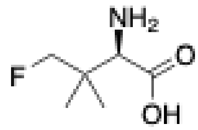

Prepared according to general procedure I, from allylic amide (*S*)-**39** (100 mg, 0.28 mmol) and $NaIO_4$ (248 mg, 1.1 mmol), $RuCl_3$ (~1 mg) in MeCN/H_2O/EtOAc (1:1.5:1, 2 mL). Then taken up in EtOH (1 mL) with NaOH (5M, 1.68 mL). Purified by ion exchange chromatography (DOWEX 50WX8 hydrogen form, eluting with 0.5M NH_4OH) and lyophilised to afford (*R*)-**41** as an ammonium salt, white solid (20 mg, 48% over two steps).

$[\alpha]^{20}_D$ +4.3° (*c* 0.07, H_2O).

^1H NMR (500 MHz, D_2O) δ 4.43 (dd, *J* = 47.0, 9.5 Hz, 1H, diastereotopic C**H**HF), 4.37 (dd, *J* = 47.4, 9.5 Hz, 1H, diastereotopic CH**H**F), 3.57 (s, 1H, C**H**NH), 1.07 (m, 6H, overlapping diastereotopic C**H**$_3$).

$^{19}F\{^1H\}$ NMR (470 MHz, D_2O) δ -221.6.

$^{13}C\{^1H\}$ NMR (176 MHz, D_2O) δ 172.6 (**C**=O), 89.6 (d, *J* = 169.8 Hz, **C**H_2F), 60.6 (C**H**NH), 36.1 (d, *J* = 16.5 Hz, 4ry **C**), 19.77 (d, *J* = 31.1 Hz, diastereotopic **C**H_3), 19.73 (d, *J* = 31.2 Hz, diastereotopic **C**H_3).

HRMS (ESI) m/z $[C_6H_{12}ONFNa]^+$ requires [M] 172.0744, found 172.0744.

D-γ,γ'-Difluoro *tert*-leucine (42)

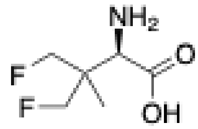

Prepared according to general procedure I, from allylic amide (*S*)-**40** (32 mg, 0.086 mmol), $NaIO_4$ (75 mg, 0.34 mmol), $RuCl_3$ (~1 mg) in MeCN/H_2O/EtOAc (1:1.5:1, 1 mL). Then taken up in EtOH (0.5 mL) with NaOH (5M, 0.5 mL). Purified by ion exchange chromatography (DOWEX 50WX8 hydrogen form, eluting with 0.5M NH_4OH) and lyophilised to afford (*R*)-**42** as an ammonium salt, white sold (8.2 mg, 57% over two steps).

$[\alpha]^{20}_D$ +4.4° (*c* 0.25, H_2O).

^1H NMR (400 MHz, D_2O) δ 4.63 (dd, *J* = 46.4, 9.8 Hz, 1H, diastereotopic C**H**HF), 4.62 (dd, *J* = 46.5, 10.2 Hz, 1H, diastereotopic C**H**HF), 4.56 (ddd, *J* = 46.6, 10.0, 1.6 Hz, 1H, diastereotopic CH**H**F), 4.55 (dd, *J* = 47.2, 9.9 Hz, 1H, diastereotopic CH**H**F), 3.79 (s, 1H α-**H**), 1.06 (t, *J* = 2.1 Hz, 3H, C**H**$_3$).

^{19}F NMR (471 MHz, D_2O) δ -227.4 (d, *J* = 46.0 Hz, 1F, diastereotopic CH_2**F**), -227.6 (d, *J* = 46.3 Hz, 1F, diastereotopic CH_2**F**).

$^{13}C\{^1H\}$ NMR (126 MHz, D_2O) δ 171.7 (**C**=O), 85.6 (dd, *J* = 170.1, 5.7 Hz, diastereotopic **C**H_2F), 85.5 (dd, *J* = 171.3, 5.1 Hz, diastereotopic **C**H_2F), 57.4 (t, *J* = 2.0 Hz, **C**HNH), 40.7 (t, *J* = 16.7 Hz, 4ry **C**), 13.5 (t, *J* = 6.0 Hz, **C**H_3).

HRMS (ESI) m/z $[C_6H_{11}O_2F_2Na]^+$ requires [M] 190.0650, found 190.648.

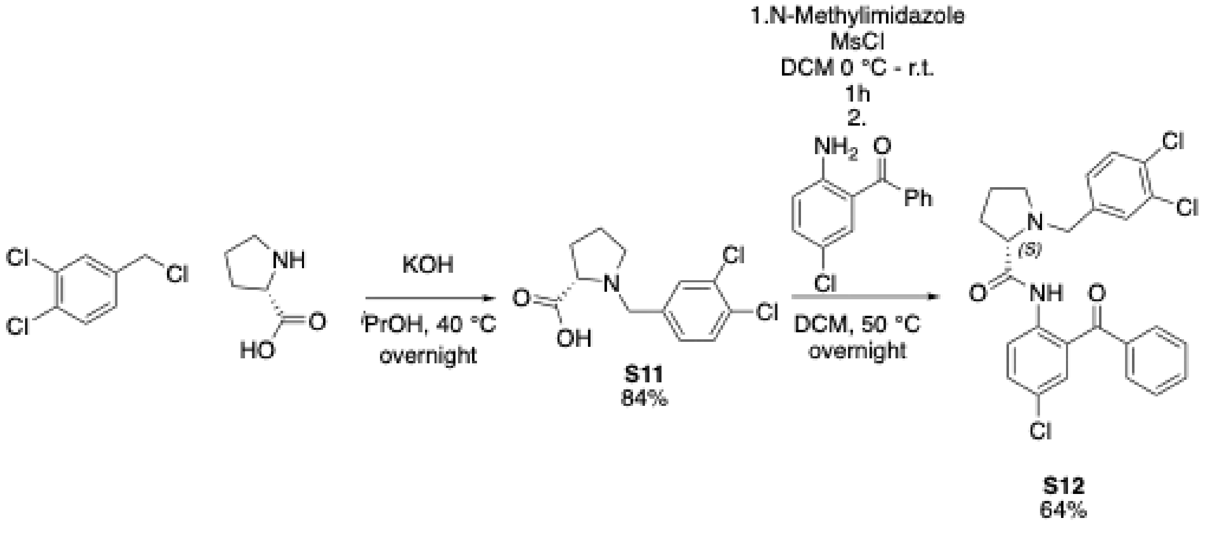

Scheme S3: Preparation of ligand **S12**.

N-(3,4-Dichlorobenzyl)-L-proline (S11)

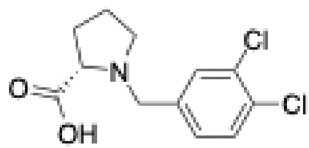

Prepared according to a literature procedure,[11] a suspension of L-proline (6.0 g, 52 mmol) and KOH (11.6 g, 208 mmol) in isopropanol (50 mL) was stirred at 40 °C until the solution became transparent. 3,4-Dichlorobenzyl chloride (8.6 mL, 62 mmol) was added and the resulting solution was stirred at 40 °C overnight. Mixture was cooled to 0 °C at pH adjusted to 5 with conc. HCl, before diluting with chloroform (50 mL) and stirring at room temperature overnight. Resulting precipitate was filtered and filtrate concentrated to afford the crude product, recrystallised from acetone to give **S11** as a white solid (12 g, 84%).

^{1}H NMR (500 MHz, DMSO) δ 7.62 (d, *J* = 1.9 Hz, 1H, Ar**H**), 7.59 (d, *J* = 8.2 Hz, 1H, Ar**H**), 7.35 (dd, *J* = 8.2, 2.0 Hz, 1H, Ar**H**), 3.96 (d, *J* = 13.5 Hz, 1H, diastereotopic NC**H**HAr), 3.63 (d, *J* = 13.6 Hz, 1H, diastereotopic NCH**H**Ar), 3.32 – 3.27 (m, 1H, proline α-**H**), 2.96 (ddd, *J* = 9.3, 7.5, 3.9 Hz, 1H, proline C**H**H), 2.45 (q, *J* = 7.9 Hz, 1H, proline C**H**H), 2.15 – 2.06 (m, 1H), 1.89 – 1.80 (m, 1H, proline C**H**H), 1.80 – 1.65 (m, 2H, proline C**H**$_2$).

Data in accordance with literature.[11]

Soloshonok ligand (S12)

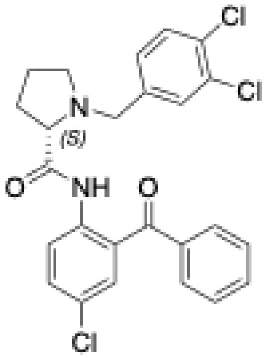

Prepared according to a literature procedure,[11] to a solution of **S11** (4.17 g, 15.2 mmol) and *N*-methylimidazole (2.66 mL, 33.4 mmol) in dry DCM (66 mL) at 0 °C under nitrogen atmosphere was added MsCl (1.2 mL, 15.2 mmol), mixture was allowed to warm to room temperature and stir for 1h. To this mixture was added 2-amino-5-chlorobenzene (4.26 g, 18.4 mmol) in dry DCM (10 mL) dropwise, on complete addition mixture was heated to 50 °C for 24h. Sat. NH_4Cl (50 mL) was added and mixture was extracted with DCM (3 x 30 mL). Combined organics were dried over $MgSO_4$ and concentrated. Residue was taken up in acetone (70 mL) and conc.

HCl (2.7 mL) was added and stirred for 3h until complete precipitation of the product. Recrystallised from acetone/Et_2O (1:1) to afford **S12** as an off-white solid (4.7 g, 64%).

^{1}H NMR (500 MHz, MeOD) δ 7.78 (d, *J* = 8.1 Hz, 1H, Ar**H**), 7.69 – 7.59 (m, 3H, Ar**H**), 7.58 – 7.49 (m, 3H, Ar**H**), 7.45 (s, 1H, Ar**H**), 7.40 – 7.32 (m, 2H, Ar**H**), 4.37 – 4.26 (m, 2H, ArC$\mathbf{H}_2$Ar), 4.26 – 4.21 (m, 1H, proline α-**H**), 3.59 – 3.50 (m, 1H, proline NC**H**H), 3.32 – 3.24 (m, 1H, proline NC**H**H), 2.37 (dq, *J* = 16.4, 8.3 Hz, 1H, proline C**H**H), 2.21 – 2.08 (m, 1H, proline C**H**H), 1.87 (dp, *J* = 16.1, 8.3 Hz, 1H, proline C**H**H), 1.60 (dq, *J* = 13.2, 6.7 Hz, 1H, proline C**H**H).

Data in accordance with literature.[11]

Soloshonok ligand/glycine nickel complex (43)

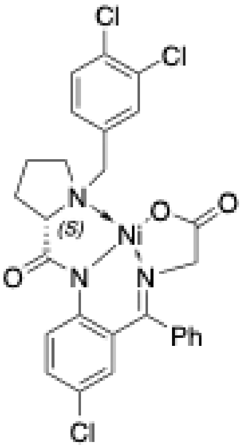

Prepared according to a modified literature procedure,[12] to a solution of **S12** (730 mg, 1.5 mmol) in MeOH (10 mL/g, 7.3 mL), was added glycine (563 mg, 7.5 mmol) and $Ni(OAc)_2.4H_2O$ (821 mg, 3.0 mmol) followed by K_2CO_3 (1.24 g, 9.0 mmol). The mixture was heated to reflux for 2h. NaH (60% dispersion in mineral oil, 120 mg, 3.0 mmol) was added and the mixture was refluxed for a further 3h before quenching by addition of 5% AcOH in ice water. Mixture was filtered, and dried under suction before taking it up in DCM and drying over $MgSO_4$ and concentrating to afford complex **43** as a red solid (887 mg, 98%);

^{1}H NMR (500 MHz, $CDCl_3$) δ 8.80 (d, *J* = 2.1 Hz, 1H, Ar**H**), 8.21 (d, *J* = 9.3 Hz, 1H, Ar**H**), 7.78 (dd, *J* = 8.2, 2.2 Hz, 1H, Ar**H**), 7.65 – 7.48 (m, 3H, Ar**H**), 7.45 (d, *J* = 8.1 Hz, 1H, Ar**H**), 7.17 (dd, *J* = 9.3, 2.7 Hz, 1H, Ar**H**), 7.14 (d, *J* = 6.8 Hz, 1H, Ar**H**), 6.94 (d, *J* = 7.4 Hz, 1H, Ar**H**), 6.75 (d, *J* = 2.7 Hz, 1H, Ar**H**), 4.38 (d, *J* = 12.6 Hz, 1H, diastereotopic NC**H**HAr), 3.78 (d, *J* = 20.2 Hz, 1H, glycine diastereotopic C**H**HN), 3.75 – 3.68 (m, 1H, proline α-proton), 3.69 (d, *J* = 20.2 Hz, 1H, glycine diastereotopic CH**H**N), 3.45 – 3.32 (m, 2H, C$\mathbf{H}_2$N), 3.36 (d, *J* =

12.8 Hz, 1H, diastereotopic NCH**H**Ar), 2.64 – 2.45 (m, 2H, proline C**H**$_2$), 2.20 – 2.04 (m, 2H, proline C**H$_2$**).

Data in accordance with literature.[12]

Soloshonok ligand/TFNP serine complex (44)

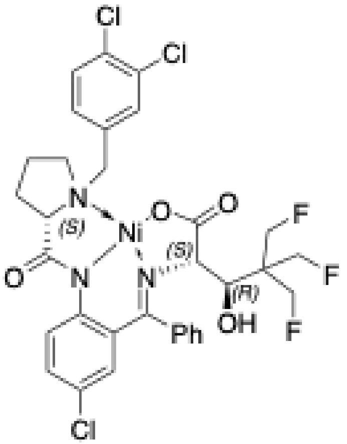

Following a modified literature procedure,[13] to a solution of complex **43** (210 mg, 0.33 mmol) in dry methanol (0.5 mL), was added MeONa (30% w/v in MeOH, 200 µL, 1 mmol) at room temperature. To this was added aldehyde **9** (70 mg, 0.5 mmol) and allowed to stir at room temperature for 10 min. Reaction was quenched by pouring onto 10% AcOH in ice water (5 mL). Solid was filtered, washed with water and air dried to afford the crude product. This was then dissolved in a minimum volume of chloroform and precipitated with hexane, stirring for a few hours before filtering off the pure product **44** as an orange solid (120 mg, 49%).

$[\alpha]^{20}_{D}$ +2197 (*c* 0.082, $CHCl_3$).

^{1}H NMR (500 MHz, $CDCl_3$) δ 8.94 (d, *J* = 2.1 Hz, 1H, Ar**H**), 8.06 (d, *J* = 9.3 Hz, 1H, Ar**H**), 7.70 (dd, *J* = 8.2, 2.2 Hz, 1H, Ar**H**), 7.59 – 7.51 (m, 2H, Ar**H**), 7.49 – 7.44 (m, 1H, Ar**H**), 7.32 (d, *J* = 8.2 Hz, 1H, Ar**H**), 7.31 – 7.28 (m, 1H, Ar**H**), 7.03 (dd, *J* = 9.3, 2.6 Hz, 1H, Ar**H**), 6.96 (d, *J* = 7.7 Hz, 1H, Ar**H**), 6.59 (d, *J* = 2.6 Hz, 1H, Ar**H**), 4.85 (m, 1H, C**H**OH), 4.70 (dd, *J* = 46.9, 9.7 Hz, 3H, diastereotopic C**H**HF), 4.66 (dd, *J* = 46.5, 9.7 Hz, 3H, diastereotopic CH**H**F), 4.25 (d, *J* = 12.5 Hz, 1H, diastereotopic C**H**HAr), 4.24 (d, *J* = 8.3 Hz, 1H, C**H**N), 3.57 – 3.43 (m, 1H, proline C**H**$_2$N), 3.41 – 3.35 (m, 2H, proline C**H**N and O**H**), 3.17 (d, *J* = 12.6 Hz, 1H, diastereotopic CH**H**Ar), 2.85 – 2.74 (m, 1H, proline C**H**), 2.63 (ddd, *J* = 19.7, 13.5, 8.7 Hz, 1H, proline C**H**), 2.28 – 2.17 (m, 1H, proline C**H**), 2.15 – 2.07 (m, 1H, proline **C**H).

^{19}F{^{1}H} NMR (470 MHz, $CDCl_3$) δ -236.1.

$^{13}C\{^1H\}$ NMR (126 MHz, $CDCl_3$) δ 179.9 (proline **C**=O), 177.7 (**C**=O), 172.8 (**C**=N), 141.0 (4ry Ar**C**-N), 135.1 (4ry Ar**C**-CH_2), 133.7 (Ar**C**H), 133.7 (4ry Ar**C**-Cl), 133.6 (4ry Ar**C**-Cl), 133.5 (4ry Ar**C**-Cl), 133.0 (Ar**C**H), 132.9 (Ar**C**H), 131.2 (Ar**C**H), 130.6 (Ar**C**H), 129.8 (Ar**C**H), 129.4 (Ar**C**H), 129.2 (2 x Ar**C**H), 127.4 (Ar**C**H), 127.4 (4ry Ar**C**), 126.1 (4ry Ar**C**), 123.6 (Ar**C**H), 80.9 (dt, *J* = 172.1, 6.8 Hz, **C**H_2F), 72.4 (q, *J* = 3.5 Hz, C**H**OH), 72.3 (**C**HN), 71.9 (proline **C**HN), 63.5 (**C**H_2Ar), 58.7 (proline **C**H_2N), 49.4 (q, *J* = 15.4 Hz, 4ry **C**), 31.1 (proline **C**H_2), 23.6 (proline **C**H_2);

HRMS (ESI) m/z $[C_{32}H_{29}O_4N_3Cl_3F_3NiNa]^+$ requires [M] 762.0426, found 762.0424.

4,4-Bis(fluoromethyl)-3-(*R*)-hydroxy-(*S*)-proline (46)

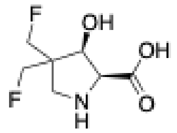

To a solution of aqueous HCl (6M, 240 μL) was added dropwise complex **44** (80 mg, 0.11 mmol) in methanol (200 μL) and heated to reflux for 20 min. On cooling to room temperature, pH was made basic using NH_3 (aq) and then extracted with DCM (2 x 1 mL). The aqueous layer pH was adjusted to 1 using HCl and subjected to ion exchange chromatography (DOWEX 50WX8 hydrogen form, eluting with 0.5M NH_4OH) before lyophilising to afford **46** as an ammonium salt, white solid (15 mg, 70%).

$[\alpha]^{20}_D$ -9.70 (*c* 0.33, H_2O).

^1H NMR (500 MHz, D_2O) δ 4.78 (ddd, *J* = 46.0, 9.7, 2.2 Hz, 1H, diastereotopic C**H**HF), 4.67 (ddd, *J* = 46.5, 9.8, 1.8 Hz, 1H, diastereotopic CH**H**F), 4.65 (ddd, *J* = 46.5, 13.6, 1.3 Hz, 1H, diastereotopic C**H**HF), 4.64 (ddd, *J* = 46.5, 13.7, 1.3 Hz, 1H, diastereotopic CH**H**F), 4.60 (d, *J* = 4.4 Hz, 1H, C**H**OH), 4.40 (d, *J* = 4.4 Hz, 1H, C**H**NH), 3.51 (dd, *J* = 12.9, 2.4 Hz, 1H, diastereotopic C**H**HNH), 3.42 (d, *J* = 12.8 Hz, 1H, diastereotopic CH**H**NH).

$^{19}F\{^1H\}$ NMR (470 MHz, D_2O) δ -229.6 (s, 1F, diastereotopic CH_2**F**), -230.9 (s, 1F, diastereotopic CH_2**F**).

$^{13}C\{^1H\}$ NMR (176 MHz, D_2O) δ 170.5 (**C**=O), 83.95 (dd, *J* = 171.0, 5.1 Hz, diastereotopic **C**H_2F), 82.24 (dd, *J* = 167.2, 5.7 Hz, diastereotopic **C**H_2F), 73.20 (t, *J* = 3.7 Hz, **C**HOH), 67.91 (d, *J* = 1.7 Hz, **C**HNH), 53.08 (t, *J* = 17.2 Hz, 4ry **C**) 46.90 (t, *J* = 7.0 Hz, **C**H_2NH).

HRMS (ESI) m/z $[C_7H_{11}O_3NF_2Na]^+$ requires [M] 218.0599, found 218.0597.

3-Fluoro-2,2-bis(fluoromethyl)propanoic acid (PA-3)

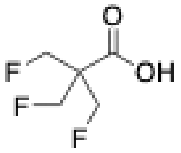

To a solution of alcohol **16** (100 mg, 0.7 mmol) in MeCN/H_2O/EtOAc (1: 1.5 :1, 4 mL) was added $NaIO_4$ (602 mg, 2.81 mmol) and stirred until completely dissolved. $RuCl_3$ (~1 mg) was added, and the mixture was stirred vigorously at room temperature for 3h. Mixture was diluted with EtOAc (10 mL) and washed with 2M HCl (10 mL). Aqueous layer was extracted with EtOAc (3 x 5 mL), combined organics were washed with $NaHSO_3$ (10 mL) and brine (10 mL) before drying over $MgSO_4$ and concentrating to afford **PA-3** as a white crystalline solid (100 mg, 92%).

^{1}H NMR (500 MHz, $CDCl_3$) δ 4.70 (dt, *J* = 46.2, 1.1 Hz, 6H, C**H**$_2$F).

^{19}F{^{1}H} NMR (470 MHz, $CDCl_3$) δ -234.9.

^{13}C{^{1}H} NMR (126 MHz, $CDCl_3$) δ 174.1 (**C**=O), 79.5 (dt, *J* = 175.3, 5.9 Hz, **C**H$_2$F), 53.9 (q, *J* = 18.4 Hz, 4ry **C**).

Data in accordance with literature.[14]

Benzyl 3-fluoro-2,2-bis(fluoromethyl)propanoate (PE-3)

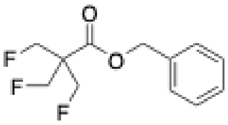

Prepared according to general procedure H, from **PA-3** (100 mg, 0.64 mmol), K_2CO_3 (177 mg, 1.28 mmol) and benzyl bromide (114 µL, 0.96 mmol). Purified by silica gel column chromatography (0-5% EtOAc in hexane) to afford **PE-3** as a colourless oil (79 mg, 50%).

^{1}H NMR (500 MHz, $CDCl_3$) δ 7.44 – 7.29 (m, 5H, Ar**H**), 5.24 (s, 2H, OC**H**$_2$Ar), 4.70 (d, *J* = 46.2 Hz, 6H, 3 x C**H**$_2$F).

^{19}F{^{1}H} NMR (470 MHz, $CDCl_3$) δ -234.8.

^{13}C{^{1}H} NMR (101 MHz, CDCl3) δ 168.9 (q, *J* = 4.6 Hz, **C**=O), 135.1 (4ry Ar**C**), 128.8 (Ar**C**H), 128.7 (Ar**C**H), 128.1 (Ar**C**H), 79.7 (dt, *J* = 174.7, 6.2 Hz, **C**H$_2$F), 67.6 (O**C**H$_2$Ar), 54.05 (q, *J* = 18.5 Hz, 4ry **C**).

HRMS (ESI) m/z $[C_{12}H_{13}O_2F_3Na]^+$ requires [M] 269.0760, found 269.0757.

Benzyl 2,2-dimethyl-3-oxopropanoate (S6)

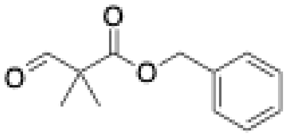

To a solution of **PE-6** (3.0 g, 15 mmol) and 4 Å MS (2.0 g) in DCM (30 mL) at room temperature was added PCC (4.8 g, 22.5 mmol) and allowed to stir overnight. Mixture was passed through a silica plug, washing with DCM (3 x 15 mL) to afford **S6** as a colourless oil (2.5 g, 80% yield).

^{1}H NMR (500 MHz, $CDCl_3$) δ 9.68 (s, 1H, C**H**O), 7.44 – 7.29 (m, 5H, Ar**H**), 5.19 (s, 2H, OC**H**$_2$Ar), 1.37 (s, 6H, 2 x C**H**$_3$).

Data in accordance with literature.[15]

Benzyl *gem*-difluoropivalate (PE-4)

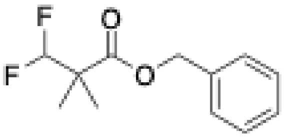

To a solution of **S6** (1.0 g, 4.8 mmol) in dry DCM (10 mL) at 0 °C was added dropwise DAST (1.9 mL, 14.5 mmol), followed by EtOH (10 µL, cat.). The resulting solution was allowed to warm to room temperature and stirred overnight until complete consumption of starting aldehyde. Mixture was quenched by addition of sat. $NaHCO_3$ solution and extracted with DCM (2 x 10 mL). Combined organics were washed with water (20 mL), brine (20 mL), dried over $MgSO_4$ and concentrated to afford crude product. Purified by silica gel column chromatography (0-5% EtOAc in hexane) to afford **PE-4** as a pale-yellow oil (774 mg, 71%).
1

H NMR (400 MHz, $CDCl_3$) δ 7.42 – 7.31 (m, 5H, Ar**H**), 6.00 (t, J = 56.4 Hz, 1H, C**H**F$_2$), 5.17 (s, 2H, OC**H**$_2$Ar), 1.30 (t, J = 1.1 Hz, 6H, 2 x C**H**$_3$).

^{19}F{^{1}H} NMR (377 MHz, $CDCl_3$) δ -128.4.

^{13}C{^{1}H} NMR (126 MHz, $CDCl_3$) δ 172.9 (t, J = 4.9 Hz, **C**=O), 135.6 (4ry Ar**C**), 128.8 (Ar**C**H), 128.5 (Ar**C**H), 128.0 (Ar**C**H), 117.5 (t, J = 245.2 Hz, **C**HF$_2$), 67.0 (O**C**H$_2$Ar), 47.1 (t, J = 21.1 Hz, 4ry **C**), 18.3 (t, J = 3.9 Hz, **C**H$_3$).

HRMS (ESI) m/z $[C_{12}H_{14}O_2F_2Na]^+$ requires [M] 251.0854, found 251.0853.

***Gem*-difluoropivalic acid (PA-4)**

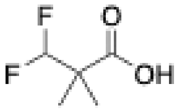

Prepared according to general procedure I, from **PE-4** (2.5 g, 11 mmol) and Pd/C (250 mg) pressurised to 15 bar H_2. Afforded **PA-4** after workup as a white crystalline solid (1.1 g, 72%).

^{1}H NMR (400 MHz, $CDCl_3$) δ 5.98 (t, *J* = 56.2 Hz, 1H, C**H**F_2), 1.31 (t, *J* = 1.1 Hz, 6H, 2 x **C**H_3).

^{19}F{^{1}H} NMR (377 MHz, $CDCl_3$) δ -128.5.

^{13}C{^{1}H} NMR (101 MHz, $CDCl_3$) δ 179.6 (t, *J* = 4.4 Hz, **C**=O), 117.2 (t, *J* = 245.4 Hz, **C**HF_2), 46.9 (t, *J* = 21.4 Hz, 4ry **C**), 18.2 (t, *J* = 3.8 Hz, 2 x **C**H_3).

HRMS (ESI) m/z $[C_5H_7O_2F_2]^-$ requires [M] 137.0420, found 137.0419.

Benzyl 3,3,3-trifluoro-2,2-dimethylpropanoate (PE-5)

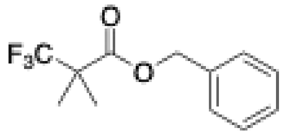

Prepared according to general procedure H, from **PA-5** (100 mg, 0.64 mmol), K_2CO_3 (177 mg, 1.28 mmol) and benzyl bromide (114 µL, 0.96 mmol) in DMF. Purified by silica gel column chromatography (0-5% EtOAc in hexane) to afford **PE-5** as a colourless oil (60 mg, 38%).

^{1}H NMR (500 MHz, $CDCl_3$) δ 7.41 – 7.30 (m, 5H, Ar**H**), 5.20 (s, 2H, OC**H**$_2$Ar), 1.44 (s, 6H, 2 x C**H**$_3$).

^{19}F{^{1}H} NMR (377 MHz, $CDCl_3$) δ -74.8.

^{13}C{^{1}H} NMR (126 MHz, $CDCl_3$) δ 170.4 (**C**=O), 135.4 (4ry Ar**C**), 128.7 (Ar**C**H), 128.5 (Ar**C**H), 127.9 (Ar**C**H), 126.5 (q, *J* = 282.9 Hz, **C**F_3), 67.5 (**C**H_2O), 48.7 (q, *J* = 26.3 Hz, 4ry **C**), 19.8 (q, *J* = 2.7 Hz, **C**H_3).

HRMS (ESI) m/z $[C_{12}H_{13}O_2F_3Na]^+$ requires [M] 269.0760, found 269.0758.

Benzyl pivalate (PE)

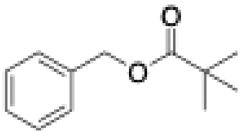

Prepared according to general procedure H, from pivalic acid **PA** (100 mg, 1 mmol), K_2CO_3 (276 mg, 2 mmol) and benzyl bromide (178 μL, 1.5 mmol). Purified by silica gel column chromatography (0-5% EtOAc in hexane) to afford **PE** as a colourless oil (60 mg, 31%).

^{1}H NMR (500 MHz, $CDCl_3$) δ 7.47 – 7.30 (m, 5H, Ar**H**), 5.11 (s, 2H, OC**H**$_2$), 1.23 (s, 9H, C**H**$_3$).

Data consistent with literature.[16]

Boc-3-hydroxy-1*H*-pyrazole (48)

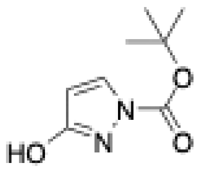

To a solution of 1*H*-pyrazol-3-ol (1.0 g, 11.9 mmol) and triethylamine (2.48 mL, 17.8 mmol) in DCM (10 mL) was added dropwise Boc anhydride (2.85 g, 13.1 mmol) and mixture was allowed to stir overnight. Mixture was diluted with DCM (10 mL) and extracted with water (10 mL) and aqueous layer was acidified using 1M citric acid solution before extracting with EtOAc (3 x 10 mL). Combined organics were dried over $MgSO_4$ and concentrated to afford **48** as a yellow solid (1.3 g, 59%).

^{1}H NMR (500 MHz, DMSO) δ 10.90 (br s, 1H, N**H**), 7.97 (d, *J* = 2.9 Hz, 1H, Ar**H**), 5.89 (d, *J* = 2.9 Hz, 1H, Ar**H**), 1.53 (s, 9H, 3 x **C**H$_3$).

^{13}C{^{1}H} NMR (126 MHz, DMSO) δ 164.0 (**C**=O), 147.3 (4ry pyrazole **C**), 132.5 (pyrazole **C**H), 98.2 (pyrazole **C**H), 83.7 (4ry O**C**), 27.6 (**C**H$_3$).

HRMS (ESI) m/z $[C_8H_{12}O_3N_2Na]^+$ requires [M] 207.0740, found 207.0740.

Boc-3-(3-fluoro-2,2-bis(fluoromethyl)propoxy)-1*H*-pyrazole (49)

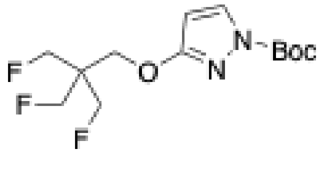

To a solution of Boc-pyrazole **48** (520 mg, 2.82 mmol), alcohol **16** (400 mg, 2.82 mmol) in dry toluene (15 mL) at room temperature was added triphenylphosphine (814 mg, 3.10 mmol), followed by DIAD (626 mg, 3.10 mmol). Mixture was heated to 110 °C under N_2 atmosphere overnight. On completion, mixture was concentrated and purified by silica gel column chromatography (5-10% EtOAc in hexane) to afford **49** as a colourless oil (630 mg, 72%).

^1H NMR (400 MHz, $CDCl_3$) δ 7.85 (d, *J* = 3.0 Hz, 1H, Ar**H**), 5.88 (d, *J* = 3.0 Hz, 1H, Ar**H**), 4.61 (dt, *J* = 46.8, 1.4 Hz, 6H, C**H**$_2$F), 4.40 (q, *J* = 1.4 Hz, 2H, C**H**$_2$O), 1.62 (s, 9H, 3 x C**H**$_3$).

$^{19}F\{^1H\}$ NMR (377 MHz, $CDCl_3$) δ -238.6.

$^{13}C\{^1H\}$ NMR (101 MHz, $CDCl_3$) δ 165.0 (**C**=O), 147.5 (4ry Ar**C**), 132.6 (Ar**C**H), 97.4 (Ar**C**H), 85.1 (4ry O**C**(CH_3)$_3$), 80.9 (dt, *J* = 173.3, 5.6 Hz, 3 x **C**H$_2$F), 65.5 (q, *J* = 5.4 Hz, **C**H$_2$O), 46.0 (q, *J* = 16.9 Hz, 4ry **C**), 31.7 (4ry **C**).

HRMS (ESI) m/z $[C_{13}H_{19}O_3N_2F_3Na]^+$ requires [M] 331.1240, found 331.1238.

3-(3-Fluoro-2,2-bis(fluoromethyl)propoxy)-1*H*-pyrazole (50)

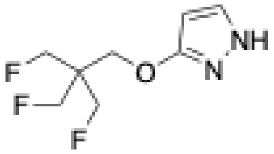

49 (630 mg, 2.04 mmol) was taken up in 4M HCl in dioxane (3 mL) and stirred at 45 °C for 1h. Mixture was evaporated to dryness and dissolved in 1M NaOH (10 mL) and extracted with EtOAc (3 x 10 mL). Combined organics were washed with brine (10 mL), dried over $MgSO_4$ and concentrated to afford **50** as a white crystalline solid (392 mg, 77%).

^1H NMR (400 MHz, $CDCl_3$) δ 7.37 (d, *J* = 2.5 Hz, 1H, Ar**H**), 5.75 (d, *J* = 2.5 Hz, 1H, Ar**H**), 4.63 (dt, *J* = 46.9, 1.4 Hz, 6H, 3 x C**H**$_2$F), 4.28 (q, *J* = 1.4 Hz, 2H, C**H**$_2$O).

$^{19}F\{^1H\}$ NMR (377 MHz, $CDCl_3$) δ -238.4.

$^{13}C\{^1H\}$ NMR (126 MHz, $CDCl_3$) δ 159.8 (4ry pyrazole **C**), 132.6 (pyrazole **C**H), 90.2 (pyrazole **C**H), 80.5 (dt, *J* = 173.0, 5.4 Hz, **C**H$_2$F), 67.7 (q, *J* = 5.4 Hz, O**C**H$_2$), 46.3 (q, *J* = 17.0 Hz, 4ry **C**).

HRMS (ESI) m/z $[C_8H_{11}ON_2F_3Na]^+$ requires [M] 231.0716, found 231.0712.

Ethyl 2-chloro-6-(3-(3-fluoro-2,2-bis(fluoromethyl)propoxy)-1*H*-pyrazol-1-yl)nicotinate (52)

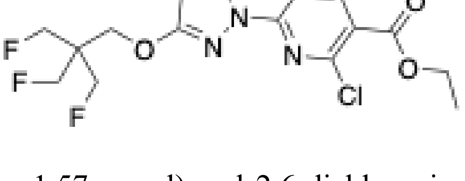

To a solution of **50** (392 mg, 1.57 mmol) and 2,6-dichloronicotinic acid **51** (345 mg, 1.57 mmol) in DMF (2 mL) was added K_2CO_3 (325 mg, 2.36 mmol) and DABCO (27 mg, 0.24

mmol) and allowed to stir at room temperature overnight. On completion solvent was removed under reduced pressure and mixture was purified by silica gel column chromatography (10-20% EtOAc in hexane) to afford **52** as a white crystalline solid (439 mg, 71%).

^{1}H NMR (400 MHz, $CDCl_3$) δ 8.39 (d, *J* = 2.9 Hz, 1H, pyrazole **H**), 8.28 (d, *J* = 8.5 Hz, 1H, pyridine **H**), 7.72 (d, *J* = 8.4 Hz, 1H, pyridine **H**), 5.98 (d, *J* = 2.9 Hz, 1H, pyrazole **H**), 4.66 (dt, *J* = 46.8, 1.4 Hz, 6H, 3 x C**H**$_2$F), 4.41 (q, *J* = 7.1 Hz, 2H, OC**H**$_2$CH$_3$), 4.41 (q, *J* = 1.4 Hz, 2H, OC**H**$_2$), 1.42 (t, *J* = 7.1 Hz, 3H, OCH$_2$C**H**$_3$).

^{19}F{^{1}H} NMR (377 MHz, $CDCl_3$) δ -238.5.

^{13}C{^{1}H} NMR (126 MHz, $CDCl_3$) δ 165.3 (4ry pyrazole **C**), 164.2 (4ry pyridine **C**), 151.9 (4ry pyridine **C**), 149.6 (4ry pyridine **C**), 143.4 (pyridine **C**H), 129.9 (pyridine **C**H), 122.3 (4ry pyridine **C**), 109.3 (pyridine **C**H), 97.0 (pyrazole **C**H), 81.0 (dt, *J* = 172.6, 5.4 Hz, **C**H$_2$F), 65.5 (q, *J* = 5.4 Hz, **C**H$_2$O), 62.0 (O**C**H$_2$CH$_3$), 46.1 (q, *J* = 16.6 Hz, 4ry **C**), 14.4 (**C**H$_3$).

HRMS (ESI) m/z $[C_{16}H_{17}O_3N_3ClF_3Na]^+$ requires [M] 414.0803, found 414.0799.

2-Chloro-6-(3-(3-fluoro-2,2-bis(fluoromethyl)propoxy)-1*H*-pyrazol-1-yl)nicotinic acid (53)

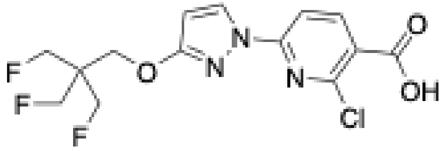

To a solution of **52** (439 mg, 1.1 mmol) in EtOH (4 mL) and THF (2 mL) was added 1M NaOH solution (1.1 mL) and heated to 40 °C for 2h. Solution was cooled to room temperature and acidified with HCl and extracted with EtOAc (3 x 10 mL). Combined organics were dried over $MgSO_4$ and concentrated to afford **53** as a white solid (400 mg, quant.).

^{1}H NMR (500 MHz, DMSO) δ 8.45 (d, *J* = 2.9 Hz, 1H, pyrazole **H**), 8.39 (d, *J* = 8.3 Hz, 1H, pyridine **H**), 7.75 (d, *J* = 8.4 Hz, 1H, pyridine **H**), 6.24 (d, *J* = 2.9 Hz, 1H, pyrazole **H**), 4.65 (d, *J* = 46.5 Hz, 6H, 3 x C**H**$_2$F), 4.35 (s, 2H, C**H**$_2$O).

^{19}F{^{1}H} NMR (470 MHz, DMSO) δ -236.9.

^{13}C{^{1}H} NMR (126 MHz, DMSO) δ 164.9 (4ry pyrazole **C**), 150.9 (4ry pyridine **C**), 147.8 (4ry pyridine **C**), 144.0 (pyridine **C**H), 130.1 (pyrazole **C**H), 123.3 (4ry pyridine **C**), 109.6 (pyridine **C**H), 97.1 (pyrazole **C**H), 81.2 (dt, *J* = 170.3, 5.4 Hz, **C**H$_2$F), 65.7 (q, *J* = 5.4 Hz, O**C**H$_2$), 45.22 (q, *J* = 16.8 Hz, 4ry **C**).

HRMS (ESI) m/z [$C_{14}H_{13}O_3N_3ClF_3Na$] requires [M] 386.0487, found 386.0486.

2-Chloro-*N*-((1,3-dimethyl-1*H*-pyrazol-4-yl)sulfonyl)-6-(3-(3-fluoro-2,2-bis(fluoromethyl)propoxy)-1*H*-pyrazol-1-yl)nicotinamide (55)

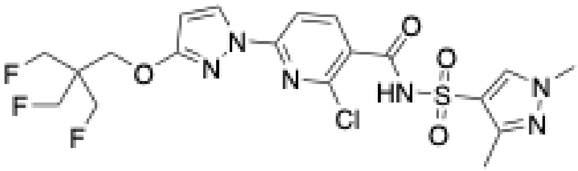

To a solution of **53** (100 mg, 0.26 mmol) in THF (1 mL) at room temperature was added CDI (120 mg, 0.74 mmol), the resulting solution was heated to 60 °C for 20 min. During this time, to a solution of sulfonamide **54** (56 mg, 0.32 mmol) in THF (100 µL) was added DBU (100 µL, 0.67 mmol). After 20 min the CDI adduct had formed, this was then added dropwise into the solution of the sulfonamide **54** with DBU, and the resulting solution was stirred for 10 min. The reaction mixture was then diluted with EtOAc (5 mL) and washed with 1M citric acid solution (5 mL). The aqueous layer was extracted with EtOAc (2 x 5 mL), combined organics were washed with brine (5 mL) and dried over $MgSO_4$ before concentrating to afford **55** as a white foam (137 mg, quant.).

^{1}H NMR (500 MHz, $CDCl_3$) δ 9.34 (br s, 1H, amide N**H**), 8.34 (d, *J* = 2.9 Hz, 1H, pyrazole **H**), 8.29 (d, *J* = 8.5 Hz, 1H, pyridine **H**), 8.08 (s, 1H, pyrazole **H**), 7.79 (d, *J* = 8.4 Hz, 1H, pyridine **H**), 6.01 (d, *J* = 2.8 Hz, 1H, pyrazole **H**), 4.64 (d, *J* = 46.8 Hz, 6H, 3 x C**H**$_2$F), 4.40 (s, 2H, C**H**$_2$O), 3.89 (s, 3H, NC**H**$_3$), 2.50 (s, 3H, C**H**$_3$).

^{19}F{^{1}H} NMR (470 MHz, $CDCl_3$) δ -238.5.

^{13}C{^{1}H} NMR (126 MHz, $CDCl_3$) δ 165.5 (4ry pyrazole **C**-O), 161.4 (**C**=O), 152.3 (4ry pyridine **C**), 149.1 (4ry pyrazole **C**), 146.1 (pyridine **C**H), 143.8 (pyrazole **C**H), 136.7 (pyridine **C**H), 130.1 (pyrazole **C**H), 123.1 (4ry pyridine **C**), 117.1 (4ry pyrazole **C**), 110.4 (pyridine **C**H), 97.8 (pyrazole **C**H), 80.9 (dt, *J* = 173.0, 5.4 Hz, **C**H$_2$F), 65.6 (q, *J* = 5.4 Hz, **C**H$_2$O), 46.1 (q, *J* = 16.9 Hz, 4ry **C**), 39.6 (N-**C**H$_3$), 12.7 (**C**H$_3$).

HRMS (ESI) m/z [$C_{19}H_{20}O_4N_6ClF_3NaS$]$^+$ requires [M] 543.0800, found 543.0797.

***Rac*-TFNP Elexacaftor (57)**

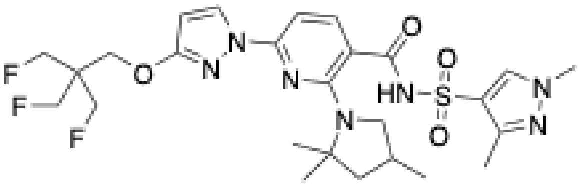

A solution of **55** (42 mg, 0.08 mmol), K_2CO_3 (55 mg, 0.4 mmol) and *rac*-2,2,4-trimethylpyrrolidine hydrochloride salt **56** (30 mg, 0.2 mmol) in DMSO (400 μL) and 1,2-diethoxyethane (100 μL) was heated to 120 °C overnight. Mixture was diluted with DCM (2 mL) and 2M HCl solution (2 mL) and layers separated. Aqueous phase was extracted with DCM (2 x 2 mL), combined organics washed with water (3 x 2 mL), dried over $MgSO_4$ and concentrate to afford the crude product. Purified by semi-prep HPLC (isocratic 60% MeCN in H_2O (0.05% TFA), Synergi C18 250x10 mm column, 2.5 mL/min, R_t 28.06 min) to afford **57** as a white foam (16.6 mg, 35%).

^1H NMR (400 MHz, DMSO) δ 12.35 (s, 1H, amide N**H**), 8.37 (s, 1H, pyrazole **H**), 8.22 (d, *J* = 2.8 Hz, 1H, pyrazole **H**), 7.74 (d, *J* = 8.2 Hz, 1H, pyridine **H**), 6.93 (d, *J* = 8.2 Hz, 1H, pyridine **H**), 6.16 (d, *J* = 2.8 Hz, 1H, pyrazole **H**), 4.65 (d, *J* = 46.6 Hz, 6H, 3 x C$\mathbf{H}_2$F), 4.31 (s, 2H, C$\mathbf{H}_2$O), 3.80 (s, 3H, NC$\mathbf{H}_3$), 2.59 – 2.50 (m, 1H, pyrrolidine **H**), 2.45 – 2.36 (m, 1H, pyrrolidine **H**), 2.32 (s, 3H, pyrazole-C$\mathbf{H}_3$) 2.24 – 2.11 (m, 1H, pyrrolidine **H**), 1.87 (dd, *J* = 11.9, 5.4 Hz, 1H, pyrrolidine **H**), 1.56 (s, 3H, diastereotopic C$\mathbf{H}_3$), 1.53 (s, 3H, diastereotopic C$\mathbf{H}_3$), 1.42 (m, 1H, pyrrolidine **H**), 0.81 (d, *J* = 6.4 Hz, 1H, C$\mathbf{H}_3$).

$^{19}F\{^1H\}$ NMR (377 MHz, DMSO) δ -236.9.

$^{13}C\{^1H\}$ NMR (126 MHz, DMSO) δ 165.2 (4ry pyrazole **C**-O), 164.0 (**C**=O), 152.3 (4ry pyridine **C**-N), 148.9 (4ry pyridine **C**-N), 146.8 (4ry pyrazole **C**-CH_3), 141.6 (pyridine **C**H), 136.5 (pyrazole **C**H), 128.5 (pyrazole **C**H), 117.3 (4ry pyrazole **C**-S), 111.2 (4ry pyridine **C**-CO), 96.2 (pyridine **C**H), 95.4 (pyrazole **C**H), 81.3 (dt, *J* = 170.8, 5.4 Hz, **C**H_2F), 65.4 (q, *J* = 5.1 Hz, **C**H_2O), 64.1 (4ry **C**$(CH_3)_2$), 57.8 (pyrrolidine **C**H_2), 50.8 (pyrrolidine **C**H_2), 45.2 (d, *J* = 16.7 Hz, 4ry **C**), 39.7 (N-**C**H_3) 29.8 (pyrrolidine **C**H), 26.5 (diastereotopic **C**H_3), 25.0 (diastereotopic **C**H_3), 16.5 (**C**H_3), 12.1 (**C**H_3).

HRMS (ESI) m/z $[C_{26}H_{34}O_4N_7F_3NaS]^+$ requires [M] 620.2237, found 620.2231.

***Rac*-Elexacaftor (*rac*-47)**

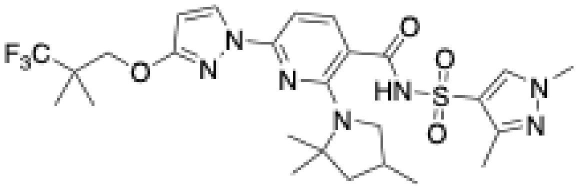

Prepared *via* an analogous route to **57**, overall yield 12%, consistent with patent data.[17]

(*R*)-1-(Benzyloxy)-3-((2-bromo-5-fluoro-4-nitrophenyl)amino)propan-2-ol (61)

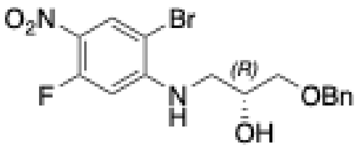

Prepared according to a literature procedure,[18] to a solution of 2-bromo-5-fluoro-4-nitro aniline **59** (400 mg, 1.73 mmol) and zinc perchlorate hexahydrate (130 mg, 0.35 mmol) in dry toluene (4 mL) was added 4 Å molecular sieves (200 mg) and stirred at room temperature for 30 min. A solution of (*R*)-2-((benzyloxy)methyl)oxirane **60** (570 mg, 3.46 mmol) in toluene (1 mL) was added dropwise to the stirring mixture before heating to 80 °C overnight. Reaction was cooled to room temperature and celite and EtOAc (4 mL) were added. Mixture was filtered and washed with NH_4Cl (10 mL) and $NaHCO_3$ (10 mL). Solvent was removed and residue was purified by silica gel column chromatography (10-20% EtOAc in hexane) to afford **61** as a yellow oil (433 mg, 63%).

^{1}H NMR (400 MHz, $CDCl_3$) δ 8.27 (d, *J* = 7.8 Hz, 1H, Ar**H**), 7.41 – 7.30 (m, 5H, 5 x Ar**H**), 6.37 (d, *J* = 13.7 Hz, 1H, Ar**H**), 5.69 (t, *J* = 4.4 Hz, 1H, N**H**), 4.59 (s, 2H, OC**H**$_2$Ar), 4.17 – 4.03 (m, 1H, C**H**OH), 3.64 (dd, *J* = 9.5, 4.3 Hz, 1H, diastereotopic C**H**HO), 3.56 (dd, *J* = 9.5, 5.4 Hz, 1H, diastereotopic CH**H**O), 3.40 (ddd, *J* = 13.1, 6.2, 4.4 Hz, 1H, diastereotopic C**H**HNH), 3.30 (ddd, *J* = 13.0, 6.7, 4.6 Hz, 1H, diastereotopic CH**H**NH), 2.53 (d, *J* = 5.6 Hz, 1H, O**H**).

^{19}F{^{1}H} NMR (377 MHz, $CDCl_3$) δ -113.3.

Data in accordance with the literature.[18,19]

***R*)-1-((4-Amino-2-bromo-5-fluorophenyl)amino)-3-(benzyloxy)propan-2-ol (60)**

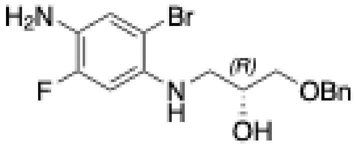

Prepared according to a literature procedure,[18] to a solution of **61** (430 mg, 1.1 mmol) in ^{i}PrOAc (4 mL) was added Pt/C (10% loading on wet support, 40 mg), pressurised to 5-10 bar and stirred overnight. Mixture was filtered through celite, washing with DCM, solvent was removed to afford **61** as a brown oil (321 mg, 77%).

^{1}H NMR (400 MHz, $CDCl_3$) δ 7.41 – 7.29 (m, 5H, 5 x Ar**H**), 6.94 (d, *J* = 9.0 Hz, 1H, Ar**H**), 6.43 (d, *J* = 12.9 Hz, 1H, Ar**H**), 4.58 (s, 2H, OC**H**$_2$Ar), 4.30 (br s, 1H, N**H**), 4.10 – 3.98 (m, 1H, C**H**OH), 3.61 (dd, *J* = 9.6, 4.0 Hz, 1H, diastereotopic C**H**HO), 3.55 (dd, *J* = 9.6, 6.0 Hz,

1H, diastereotopic CH**HO**), 3.32 (br s, 2H, N**H**$_2$), 3.28 – 3.19 (m, 1H, diastereotopic C**H**HNH), 3.19 – 3.07 (m, 1H, diastereotopic CH**H**NH), 2.53 (d, *J* = 5.1 Hz, 1H, O**H**).

^{19}F{^{1}H} NMR (470 MHz, CDCl$_3$) δ -132.9.

Data in accordance with the literature.[18,19]

(*R*)-*N*-(4-((3-(Benzyloxy)-2-hydroxypropyl)amino)-5-bromo-2-fluorophenyl)-1-(2,2-difluorobenzo[*d*][1,3]dioxol-5-yl)cyclopropane-1-carboxamide (63)

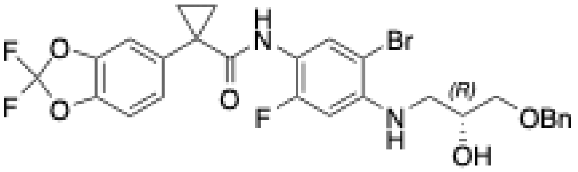

To a suspension of 1-(2,2-difluorobenzo[*d*][1,3]dioxol-5-yl)cyclopropane-1-carboxylic acid (1.2 g, 4.8 mmol) in $SOCl_2$ (1.6 mL, 22 mmol) was added a drop of DMF and allowed to stir at room temperature until a clear solution was obtained. The excess $SOCl_2$ was removed under reduced pressure and the resulting acid chloride **62** was taken up in DCM (5 mL). This was added dropwise to a stirring solution of **60** (1.3 g, 4.4 mmol) and NEt_3 (2.0 mL, 14.6 mmol) in DCM (5 mL) at room temperature and the resulting mixture was stirred overnight. Quenched with water (10 mL) and layers separated. Organic layer was dried and concentrated to afford the crude product. Purified by silica gel column chromatography (0-30% EtOAc in hexane) to give **63** as a colourless oil (1.56 g, 66%).

$[\alpha]^{20}_D$ +3.8° (*c* 1.0, CHCl$_3$).

^{1}H NMR (400 MHz, CDCl$_3$) δ 8.15 (d, *J* = 8.2 Hz, 1H, Ar**H**), 7.38 – 7.29 (m, 5H, 5x Ar**H**), 7.24 (dd, *J* = 8.2, 1.8 Hz, 1H, Ar**H**), 7.20 (d, *J* = 1.6 Hz, 1H, Ar**H**), 7.11 (d, *J* = 8.2 Hz, 1H, Ar**H**), 6.93 (br s, 1H, amide N**H**), 6.33 (d, *J* = 13.0 Hz, 1H, Ar**H**), 4.73 – 4.65 (t, *J* = 5.3 Hz, 1H, N**H**), 4.56 (s, 2H, OC**H**$_2$Ar), 4.07 – 3.97 (m, 1H, C**H**OH), 3.59 (dd, *J* = 9.5, 4.0 Hz, 1H, diastereotopic C**H**HO), 3.52 (dd, *J* = 9.5, 5.8 Hz, 1H, diastereotopic CH**HO**), 3.24 (ddd, *J* = 12.9, 6.6, 4.7 Hz, 1H, diastereotopic C**H**HNH), 3.14 (ddd, *J* = 12.9, 6.8, 5.0 Hz, 1H, diastereotopic CH**H**NH), 2.49 (d, *J* = 5.2 Hz, 1H, O**H**), 1.71 (ddd, *J* = 3.9, 3.9, 3.8 Hz, 2H, 2 x cyclopropyl C**H**H), 1.14 (ddd, *J* = 4.0, 3.9, 3.9 Hz, 2H, 2 x cyclopropyl C**H**H).

^{19}F{^{1}H} NMR (377 MHz, CDCl$_3$) δ -49.8 (OC**F**$_2$O), -128.9 (Ar**F**).

^{13}C{^{1}H} NMR (126 MHz, CDCl$_3$) δ 171.3 (**C**=O), 153.7 (d, *J* = 242.1 Hz, 4ry Ar**C**F), 144.2 (4ry Ar**C**O), 143.7 (4ry Ar**C**O), 143.1 (d, *J* = 10.4 Hz, 4ry Ar**C**NH), 137.7 (4ry Ar**C**-C), 135.3 (4ry Ar**C**-C), 131.8 (t, *J* = 256.4 Hz, O**C**F$_2$O), 128.7 (Ar**C**H), 128.1 (Ar**C**H), 128.0 (Ar**C**H),

126.7 (ArCH), 126.6 (d, J = 2.3 Hz, ArCH), 116.1 (d, J = 12.3 Hz, 4ry ArCNH), 112.4 (ArCH), 110.2 (ArCH), 103.7 (d, J = 3.2 Hz, 4ry ArCBr), 98.3 (d, J = 25.4 Hz, ArCH), 73.7 (OCH$_2$Ar), 72.2 (CH$_2$NH), 68.9 (CHOH), 46.8 (CH$_2$O), 31.1 (4ry **C**), 16.8 (cyclopropyl **C**H$_2$).

HRMS (ESI) m/z [$C_{27}H_{24}O_5N_2{}^{79}BrF_3Na]^+$ requires [M] 615.0713, found 615.0708.

(*R*)-*N*-(4-((3-(Benzyloxy)-2-hydroxypropyl)amino)-2-fluoro-5-(4-fluoro-3,3-bis(fluoromethyl)but-1-yn-1-yl)phenyl)-1-(2,2-difluorobenzo[*d*][1,3]dioxol-5-yl)cyclopropane-1-carboxamide (64)

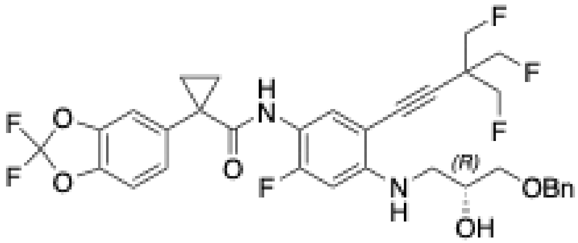

To a solution of aldehyde **9** (300 mg, 2.14 mmol) and Ohira-Bestmann reagent (360 µL, 2.4 mmol) in MeOH (4 mL) at 0 °C was added Cs_2CO_3 (1.3 g, 4.0 mmol) portion-wise. The solution was allowed to warm to room temperature and stirred for 1h, monitored by ^{19}F NMR. On completion, the MeOH and resulting alkyne **24** were distilled off (100 °C, long path, atmospheric pressure) into a Schlenk flask. The resulting methanol solution was diluted with methanol (15 mL) and degassed *via* three freeze-pump-thaw cycles before placing it under a N_2 atmosphere. To this was added aryl bromide **63** (800 mg, 1.35 mmol) and caesium carbonate (1.30 g, 4.2 mmol) and allowed to stir before adding Pd(OAc)$_2$ (12 mg, 0.05 mmol) and XPhos (52 mg, 0.11 mmol). Resulting mixture was heated to 60 °C overnight. Mixture was filtered through celite, concentrated and purified by silica gel column chromatography (20-40% EtOAc in hexane) to afford **64** as a yellow foam (244 mg, 38%).

$[\alpha]^{20}_D$ -1.4° (*c* 0.5, CHCl$_3$).

^{1}H NMR (500 MHz, CDCl$_3$) δ 7.97 (d, J = 8.5 Hz, 1H, Ar**H**), 7.38 - 7.28 (m, 5H, 5 x Ar**H**), 7.24 (dd, J = 8.2, 1.8 Hz, 1H, Ar**H**), 7.20 (d, J = 1.8 Hz, 1H, Ar**H**), 7.10 (d, J = 8.1 Hz, 1H, Ar**H**), 6.91 (br s, 1H, N**H**CO), 6.26 (d, J = 13.1 Hz, 1H, Ar**H**), 4.92 (br s, 1H, N**H**), 4.57 (d, J = 46.7 Hz, 6H, 3 x C**H**$_2$F), 4.55 (s, 2H, OC**H**$_2$Ar), 4.02 (br s, 1H, C**H**OH), 3.57 (dd, J = 9.6, 3.9 Hz, 1H, diastereotopic C**H**HO), 3.50 (dd, J = 9.6, 6.4 Hz, 1H, diastereotopic CH**H**O), 3.23 (dd, J = 12.6, 4.1 Hz, 2H, diastereotopic C**H**HNH), 3.12 (dd, J = 12.5, 6.4 Hz, 1H, diastereotopic CH**H**NH), 2.43 (br s, 1H, O**H**), 1.71 (ddd, J = 3.9, 3.9, 3.8 Hz, 2H, 2 x cyclopropyl C**H**H), 1.13 (ddd, J = 4.0, 3.9, 3.9 Hz, 2H, 2 x cyclopropyl C**H**H).

$^{19}F\{^1H\}$ NMR (470 MHz, $CDCl_3$) δ -49.9 (OC**F**$_2$O), -124.6 (Ar**F**), -228.9 (CH_2**F**).

$^{13}C\{^1H\}$ NMR (126 MHz, $CDCl_3$) δ 171.3 (**C**=O), 155.1 (d, *J* = 245.2 Hz, 4ry Ar**C**F), 147.9 (d, *J* = 11.4 Hz, 4ry Ar**C**NH), 144.2 (Ar**C**O), 143.7 (Ar**C**O), 137.7 (4ry Ar**C**-C), 135.4 (4ry Ar**C**-C), 131.8 (t, *J* = 256.6 Hz, O**C**F$_2$O), 128.7 (Ar**C**H), 128.1 (Ar**C**H), 128.0 (Ar**C**H), 126.8 (Ar**C**H), 126.7 (Ar**C**H), 115.3 (d, *J* = 12.3 Hz, 4ry Ar**C**NH), 112.4 (Ar**C**H), 110.2 (Ar**C**H), 102.4 (4ry Ar**C**Br), 97.1 (d, *J* = 25.4 Hz, Ar**C**H), 88.8 (q, *J* = 6.5 Hz, Ar-C≡**C**), 82.2 (Ar-**C**≡C), 81.7 (dt, *J* = 179.8, 4.5 Hz, **C**H$_2$F), 73.7 (O**C**H$_2$Ar), 72.1 (**C**H$_2$NH), 69.2 (**C**HOH), 46.2 (**C**H$_2$O), 44.3 (q, *J* = 17.5 Hz, 4ry **C**), 31.1 (4ry **C**), 16.8 (cyclopropyl **C**H$_2$).

HRMS (ESI) m/z $[C_{33}H_{30}O_5N_2F_6Na]^+$ requires [M] 671.1951, found 671.1953.

(*R*)-*N*-(1-(3-(Benzyloxy)-2-hydroxypropyl)-2-(1,3-difluoro-2-(fluoromethyl)propan-2-yl)-6-fluoro-1*H*-indol-5-yl)-1-(2,2-difluorobenzo[*d*][1,3]dioxol-5-yl)cyclopropane-1-carboxamide (65)

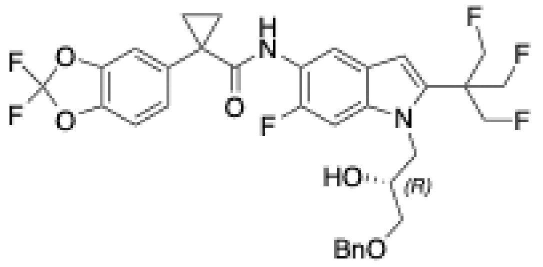

A solution of alkyne **64** (90 mg, 0.14 mmol) and $Pd(MeCN)_2Cl_2$ (6 mg, 0.024 mmol) in MeCN (500 µL) was degassed *via* a steady stream of nitrogen for 30 min before being refluxed under a blanket of nitrogen overnight. Mixture was filtered through celite and solvent was removed before purifying by silica gel column chromatography (10-30% EtOAc in hexane) to give **65** as a white foam (27 mg, 30%).

$[\alpha]^{20}_D$ +3.3° (*c* 0.15, $CHCl_3$).

^1H NMR (500 MHz, $CDCl_3$) δ 8.26 (d, *J* = 7.7 Hz, 1H, indole **H**), 7.40 – 7.31 (m, 5H, 5 x Ar**H**), 7.29 (dd, *J* = 8.2, 1.8 Hz, 1H, Ar**H**), 7.25 (d, *J* = 1.8 Hz, 1H, Ar**H**), 7.23 (d, *J* = 2.8 Hz, 1H, Ar**H**), 7.13 (d, *J* = 8.1 Hz, 1H, Ar**H**), 7.09 (d, *J* = 11.8 Hz, 1H, indole **H**), 6.40 (s, 1H, indole **H**), 4.91 (ddt, *J* = 46.7, 9.7, 1.6 Hz, 3H, diastereotopic C**H**HF), 4.86 (ddt, *J* = 47.0, 9.7, 1.6 Hz, 3H, diastereotopic CH**H**F), 4.57 (d, *J* = 11.9 Hz, diastereotopic OC**H**$_2$Ar), 4.56 (d, *J* = 11.6 Hz, diastereotopic OC**H**$_2$Ar), 4.35 (dd, *J* = 15.7, 4.4 Hz, 1H, diastereotopic C**H**HO), 4.28 (dd, *J* = 15.8, 7.7 Hz, 1H, diastereotopic CH**H**O), 4.21 – 4.15 (m, 1H, C**H**OH), 3.55 (dd, *J* = 9.6, 5.0 Hz, 1H, diastereotopic C**H**HNH), 3.49 (dd, *J* = 9.6, 5.1 Hz, 1H, diastereotopic

CH**H**NH), 2.25 (d, J = 4.9 Hz, 1H, O**H**), 1.75 (ddd, J = 3.8, 3.8, 3.7 Hz, 2H, 2 x cyclopropyl C**H**), 1.15 (ddd, J = 4.0, 3.9, 3.9 Hz, 2H, 2 x cyclopropyl C**H**).

$^{19}F\{^{1}H\}$ NMR (470 MHz, $CDCl_3$) δ -49.8 (OC**F**$_2$O), -135.4 (Ar**F**), -228.9 (CH_2**F**).

$^{13}C\{^{1}H\}$ NMR (126 MHz, $CDCl_3$) δ 171.3 (**C**=O), 151.1 (d, J = 238.4 Hz, 4ry indole **C**F), 144.2 (4ry Ar**C**O), 143.7 (4ry Ar**C**O), 137.5 (4ry Ar**C**-C), 135.6 (4ry Ar**C**-C), 135.5 (4ry indole **C**-TFTB), 134.5 (d, J = 12.1 Hz, 4ry indole **C**-NH), 131.9 (t, J = 256.5 Hz, O**C**F$_2$O), 128.7 (Ar**C**H), 128.2 (Ar**C**H), 128.0 (Ar**C**H), 126.7 (Ar**C**H), 123.3 (4ry indole **C**), 120.4 (d, J = 12.7 Hz, 4ry indole **C**), 113.4 (indole **C**H), 112.5 (Ar**C**H), 110.2 (Ar**C**H), 104.4 (indole **C**H), 97.0 (d, J = 25.4 Hz, indole **C**H), 82.4 (dt, J = 176.7, 5.7 Hz, **C**H$_2$F), 73.8 (O**C**H$_2$Ar), 71.5 (**C**H$_2$N), 69.9 (**C**HOH), 48.7 (**C**H$_2$O), 47.85 (q, J = 19.4 Hz, 4ry **C**), 31.3 (4ry **C**), 16.8 (cyclopropyl **C**H$_2$).

HRMS (ESI) m/z $[C_{33}H_{30}O_5N_2F_6Na]^+$ requires [M] 671.1951, found 671.1953.

TFTB Tezacaftor (66)

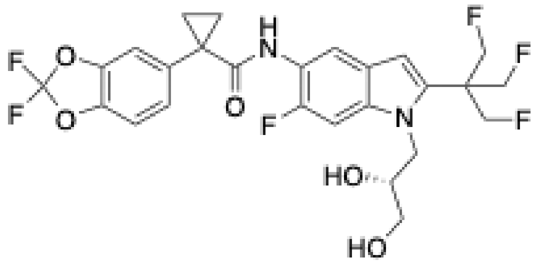

To a solution of benzyl ether protected **65** (20 mg, 0.03 mmol) in EtOAc (1 mL) was added $Pd(OH)_2/C$ (20% loading on wet support, 1 mg), sealed in a stainless-steel autoclave, pressurised to 20 bar and allowed to stir at room temperature overnight. The mixture was passed through celite, washing with DCM before solvent was removed. The crude product was purified by semi-prep HPLC (58% isocratic MeCN in H_2O with 0.1% TFA over 30 min, 2.5 mL/min, Synergi C18 250x10 mm column, detection 254 nm, R_t 16.66 min) to afford **66** as a colourless oil (12 mg, 67%).

$[\alpha]^{20}_{D}$ -10° (c 0.05, $CHCl_3$).

^{1}H NMR (500 MHz, CD_3CN) δ 7.78 (d, J = 7.7 Hz, 1H, indole **H**), 7.41 (d, J = 1.8 Hz, 1H, Ar**Hz**), 7.37 (dd, J = 8.3, 1.8 Hz, 1H, Ar**H**), 7.32 (br s, 1H, N**H**CO), 7.25 (d, J = 11.3 Hz, 1H, indole **H**), 7.25 (d, J = 8.3 Hz, 1H, Ar**H**), 6.50 (s, 1H, indole **H**), 4.97 (ddt, J = 46.5, 9.7, 1.5 Hz, 3H, diastereotopic C**H**HF), 4.91 (ddt, J = 46.3, 9.8, 1.5 Hz, 3H, diastereotopic CH**H**F), 4.33 (dd, J = 15.7, 3.0 Hz, 1H, diastereotopic C**H**HOH), 4.20 (dd, J = 15.7, 9.4 Hz, 1H,

diastereotopic C**H**HOH), 4.04 – 3.95 (m, 1H, C**H**OH), 3.57 (dd, *J* = 11.2, 5.5 Hz, 1H, diastereotopic C**H**HNH), 3.53 (dd, *J* = 11.0, 5.1 Hz, 1H, diastereotopic CH**H**NH), 1.57 (ddd, *J* = 3.8, 3.8, 3.7 Hz, 2H, 2 x cyclopropyl C**H**), 1.16 (ddd, *J* = 3.9, 3.8, 3.8 Hz, 2H, 2 x cyclopropyl C**H**).

$^{19}F\{^1H\}$ NMR (470 MHz, CD_3CN) δ -51.0 (OC**F**$_2$O), -133.6 (Ar**F**), -230.6 (CH_2**F**).

$^{13}C\{^1H\}$ NMR (126 MHz, CD_3CN) δ 172.8 (**C**=O), 153.33 (d, *J* = 237.5 Hz, indole 4ry **C**F), 144.8 (4ry Ar**C**O), 144.2 (4ry Ar**C**O), 137.1 (4ry Ar**C**-C), 136.8 (q, *J* = 3.9 Hz, indole 4ry **C**-TFTB), 136.4 (d, *J* = 11.8 Hz, indole 4ry **C**-NH), 132.7 (t, *J* = 252.7 Hz, 4ry O**C**F_2O), 128.1 (Ar**C**H), 124.1 (indole 4ry **C**), 120.5 (d, *J* = 14.5 Hz, indole 4ry **C**), 116.6 (d, *J* = 1.3 Hz, indole **C**H), 113.8 (Ar**C**H), 111.1 (Ar**C**H), 104.7 (indole **C**H), 98.6 (d, *J* = 25.9 Hz, indole **C**H), 83.3 (dt, *J* = 174.4, 5.9 Hz, **C**H_2F), 71.4 (**C**HOH), 64.8 (**C**H_2N), 49.5 (**C**H_2OH), 48.7 (q, *J* = 17.4 Hz, 4ry **C**), 31.6 (4ry **C**), 16.9 (cyclopropyl **C**H_2).

HMRS (ESI) m/z $[C_{26}H_{24}O_5N_2F_6Na]^+$ requires [M] 581.1482, found 581.1482.

Chiral HPLC analysis of allylic alcohols

Initial optimisation of the chiral HPLC protocol was performed with *rac*-**38** to generate the optimum conditions.

Chiral HPLC enantiomeric excess purity was determined using a Chiralcel OD-H column, with an injection of 5 µL of a sample at 1 mg/mL concentration (1% isopropanol in hexane). The sample was run on an isocratic 2% isopropanol in hexane method, with a flow rate of 2 mL/min and a temperature of 30 °C, with a detection at 211 nm.

Table 4.1: Chiral HPLC analysis of allylic alcohols **29**, **38** and **39**.

**Entry**	**(*R*)**		**(*S*)**		**e.e.**
	**Retention time (min)**	**Relative area**	**Retention time (min)**	**Relative area**	
**38**	7.618	98.2	10.318	1.8	96%
**39**	10.929	96.6	14.578	3.4	94%
**29**	16.347	96.9	23.421	3.1	94%

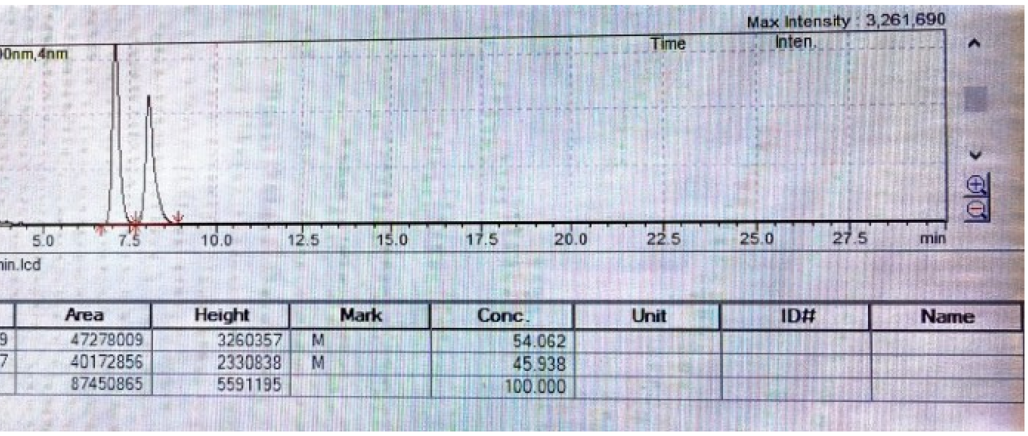

	Area	Height	Mark	Conc.	Unit	ID#	Name
9	47278009	3260357	M	54.062			
7	40172856	2330838	M	45.938			
	87450865	5591195		100.000			

Figure S1: Chiral HPLC data for rac-**38**.

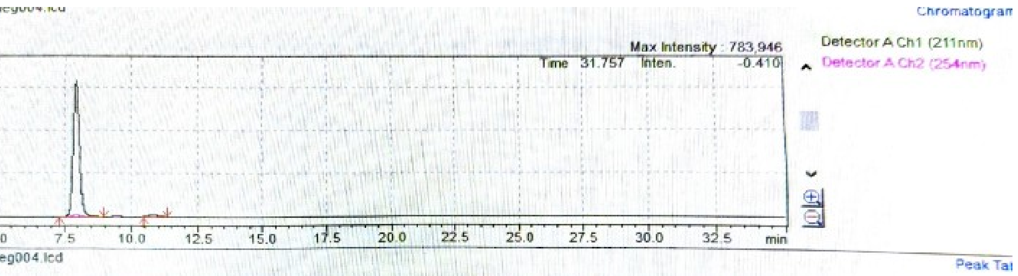

Area	Height	Mark	Conc.	Unit	ID#	Name	Area%
12923375	783814	V	97.821				97.821
287878	13511	M	2.179				2.179
13211253	797325		100.000				100.000

Figure S2: Chiral HPLC data for (R)-**38**.

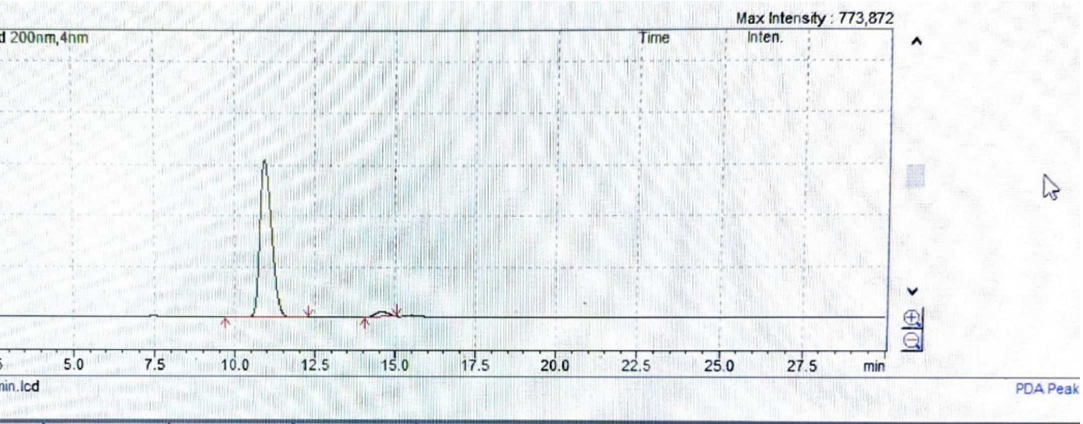

me	Area	Height	Mark	Conc.	Unit	ID#	Name	Area%
[illegible].926	19378657	773619	M	96.598				96.598
[illegible].578	682501	24014	M	3.402				3.402
	20061158	797633		100.000				100.000

Figure S3: Chiral HPLC data for (R)-**39**.

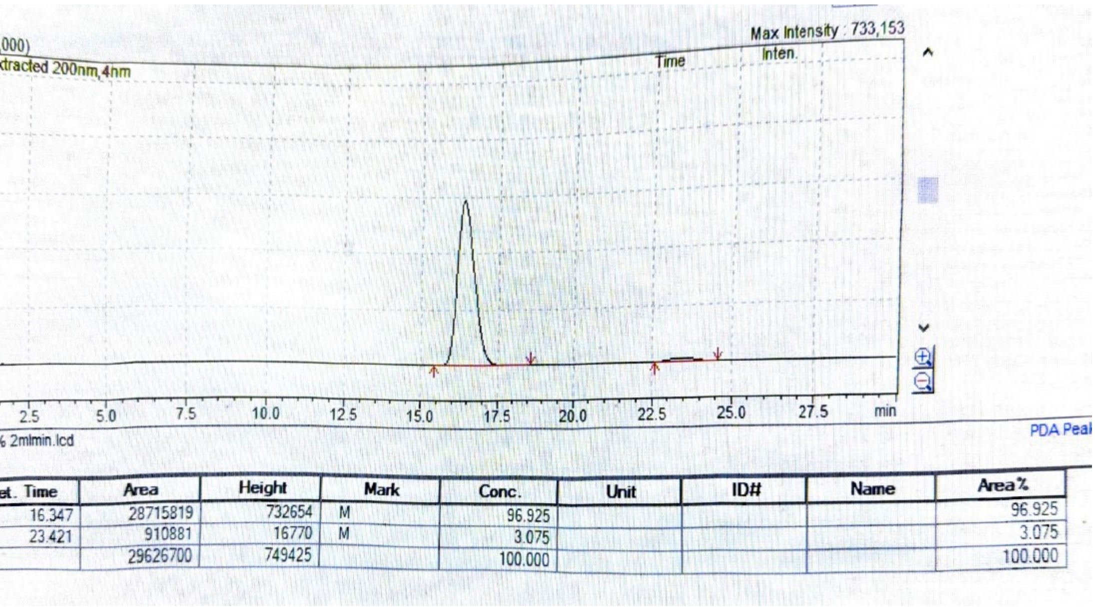

Figure S4: Chiral HPLC data for (R)-**29**.

Marfey’s reagent analysis of enantiopurity general procedure

The amino acid (~1 mg) was dissolved in 0.5 M $NaHCO_3$ solution (100 µL) and to this was added a 1% solution of Marfey’s reagent in acetone (200 µL). The mixture was warmed to 40 °C for 90 min, before being cooled and acidified with 2M HCl. Mixture was then diluted 20-fold with the eluant for the HPLC column.

Analysis by HPLC was conducted on a Kinetix C18 silica column (150 x 4.6 mm), with a sample injection of 50 µL. The sample was run on an isocratic 40% MeCN in H_2O with 0.1% TFA, with a flow rate of 1 mL/min and a detection at 254 nm.

Table 4.2*: Marfey's reagent determination of enantiopurity for amino acids* ***209-211****.*

Entry	(*R*)		(*S*)		e.e.
	**Retention time (min)**	**Relative area**	**Retention time (min)**	**Relative area**	
**41**	5.783	97.8	4.377	2.2	96%
**42**	5.358	97.1	4.186	2.9	94%
**32**	5.346	97.2	4.209	2.8	94%

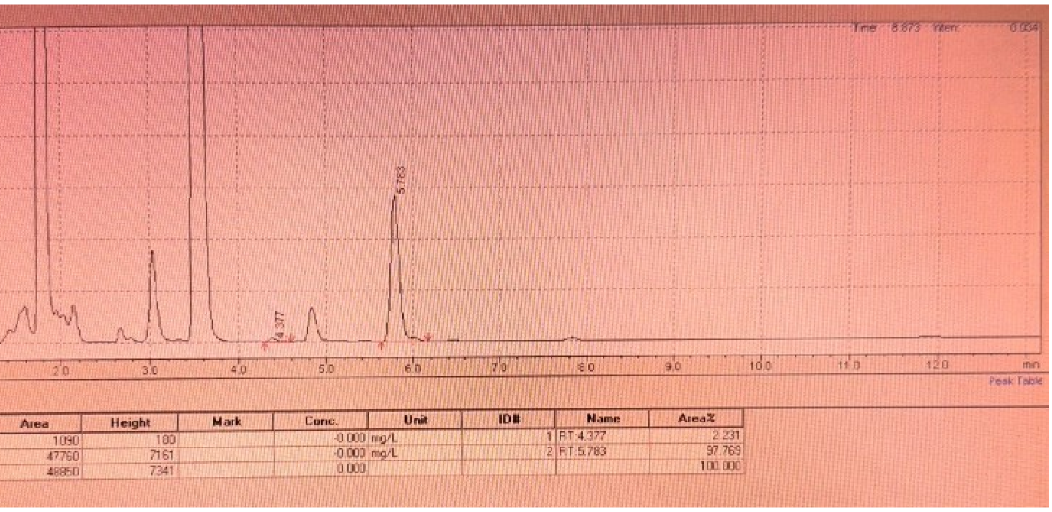

Figure S5: HPLC analysis of (R)-**41** with Marfey's reagent.

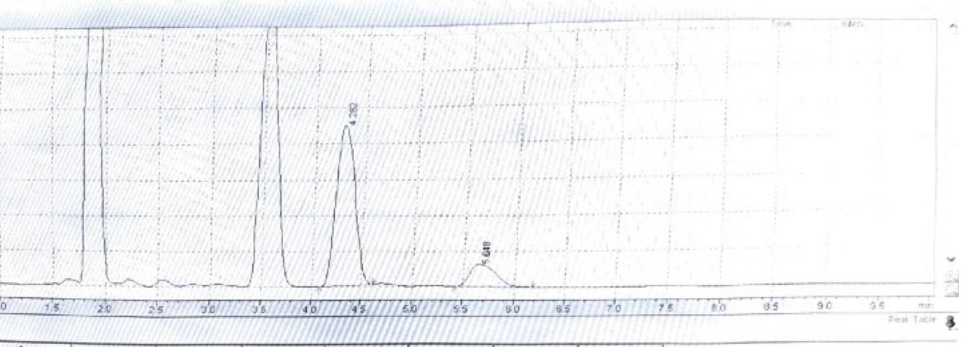

Area	Height	Mark	Conc.	Unit	ID#	Name	Area%
1451620	112047		0.000	mg/L	1	RT:4.282	84.120
274035	15874		0.000	mg/L	2	RT:5.648	15.880
1725654	127921		0.000				100.000

Figure S6: HPLC analysis of (S)-**41** with Marfey's reagent.

LogP measurements

General procedure for estimation of LogP by reverse phase HPLC

The estimation of lipophilicity values was conducted using a Kinetix® 2.6 µm C18 100 Å, 150 x 4.6 mm column on a Shimadzu Prominence HPLC system. Using an isocratic method, 60% MeCN in H_2O with 0.05% TFA, 1 mL/min, detection at 254 nm.

A series of reference compounds were injected (10 µL of 0.5 mg/mL in 60% MeCN in H_2O) and retention times (R_t) measured in triplicate. Average retention times were converted to Logk values, where k is the capacity factor of the column. Calculated as follows:

$$Capacity\ factor\ (k) = \frac{Retention\ time - dead\ time\ of\ the\ column}{Dead\ time\ of\ the\ column}$$

Wherein the dead time of the column is the time that it takes for an unretained molecule to pass through the column.

A linear graph of Logk vs literature LogP[20,21] can be plotted which allows for determination of unknown LogP values.

Reference compounds

Table S3: Retention times and Logk calculations of the reference compounds for LogP analysis.

Compound	LogP (lit)	Rt_1 (min)	Rt_2 (min)	Rt_3 (min)	Average R_t (min)	Capacity factor (k)	Logk
**Phenol**	**1.50**	2.015	2.014	2.010	2.013 ± 0.002	0.6433	**-0.1916**
**Toluene**	**2.73**	4.459	4.452	4.447	4.452 ± 0.006	2.634	**0.4207**
**Cumene**	**3.66**	7.630	7.647	7.655	7.644 ± 0.013	5.240	**0.7193**
**Pyrene**	**4.88**	14.406	14.410	14.413	14.410 ± 0.004	10.79	**1.032**

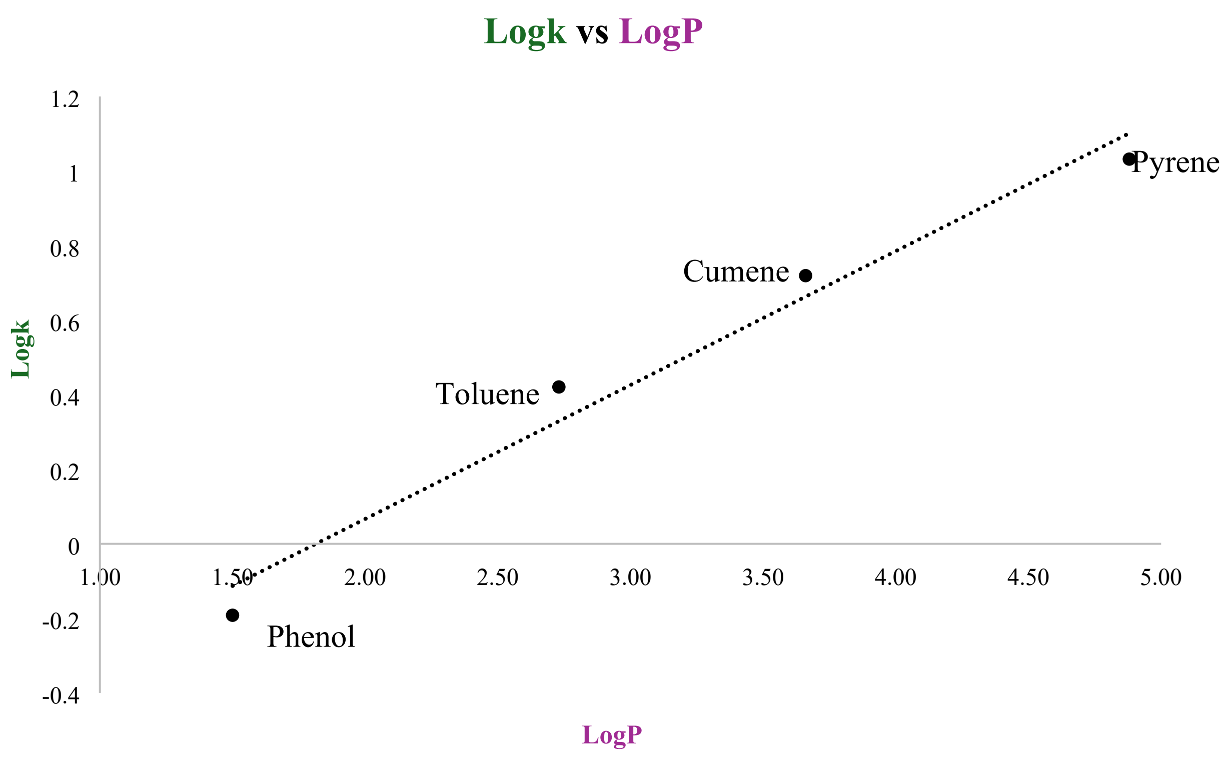

Figure S7: Linear plot of Logk vs LogP for the reference compounds

The R_t of each of the compounds was measured in triplicate, average R_t used for measurement of capacity factor. Measured Logk could then be used with the equation from the reference compounds for calculation of LogP.

LogP of benzyl pivalate ester derivatives

Table S4: Data from LogP analysis on benzyl pivalate esters**.**

Compound	LogP (measured)	Rt_1 (min)	Rt_2 (min)	Rt_3 (min)	Average R_t (min)	Capacity factor (k)	Logk
**PE**	**3.63**	6.694	6.729	6.730	6.718 ± 0.020	4.484	**0.6516**
**PE-1**	**3.13**	4.863	4.865	4.891	4.873 ± 0.016	2.978	**0.4739**
**PE-2**	**3.00**	4.476	4.477	4.490	4.481 ± 0.008	5.240	**0.7193**

**PE-3**	2.91	4.270	4.269	4.252	4.264 ± 0.010	2.481	**0.3945**
**PE-4**	3.41	5.804	5.809	5.815	5.809 ± 0.006	3.742	**0.5731**
**PE-5**	3.73	7.211	7.175	7.167	7.184 ± 0.023	4.865	**0.6514**
**PE-6**	1.89	2.533	2.528	2.527	2.529 ± 0.003	1.065	**0.0272**

LogP of elexacaftor and tezacaftor derivatives

Table S5: Data from LogP analysis of elexacaftor **40** and the TFNP derivative **50**.

Compound	LogP (measured)	Rt_1 (min)	Rt_2 (min)	Rt_3 (min)	Average R_t (min)	Capacity factor (k)	Logk
**47** (Elexacaftor)	3.72	6.892	6.895	6.900	6.896 ± 0.004	5.190	**0.7152**
**57**	4.77	14.459	14.438	14.429	14.442 ± 0.015	11.96	**1.078**
**58** (Tezacaftor)	2.27	2.935	2.942	2.941	2.939 ± 0.004	1.639	**0.2145**
**66**	2.98	4.318	4.320	4.317	4.318 ± 0.002	2.876	**0.4589**

pK_a measurements

General procedure for measurement of pK_a values

To a stirred solution of the acid to be tested (0.01 M in H_2O) was added in 50 µL portions NaOH solution (0.1 M in H_2O) and resulting pH was measured using a Thermo Scientific Orion VersaStar Pro pH meter, calibrated at 4.01, 7.00 and 10.01 using Therma Scientific Orion buffer solutions. After addition of 3.5 mL of NaOH solution, 200 µL was added to the stirred mixture until 5.1 mL total volume of NaOH had been added.

The pK_a is derived from the pH at 0.5 eq of NaOH.

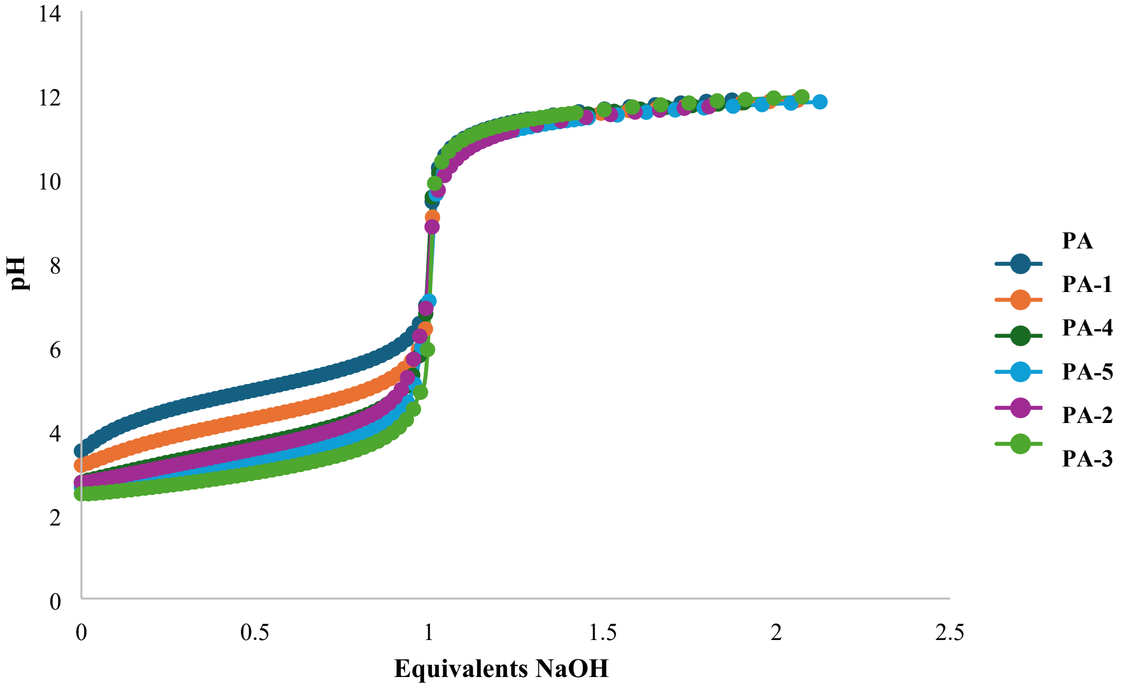

Figure S8: Titration curves of pivalic acid derivatives

NMR Spectra of synthesised compounds

11

^{1}H NMR (400 MHz, DMSO)

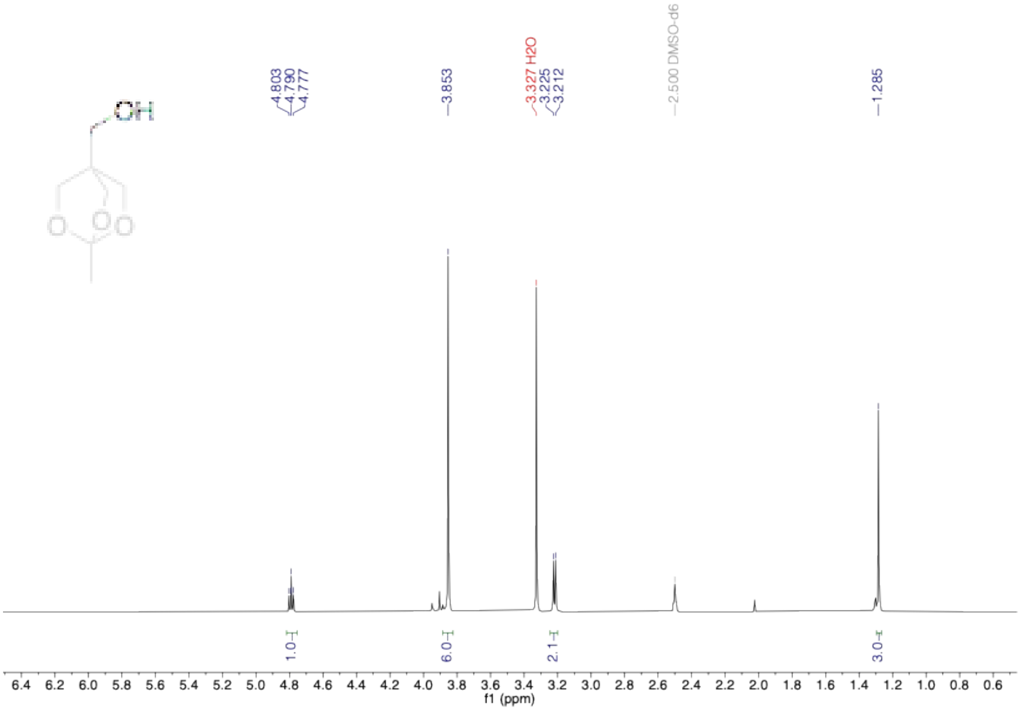

13

^{1}H NMR (500 MHz, DMSO)

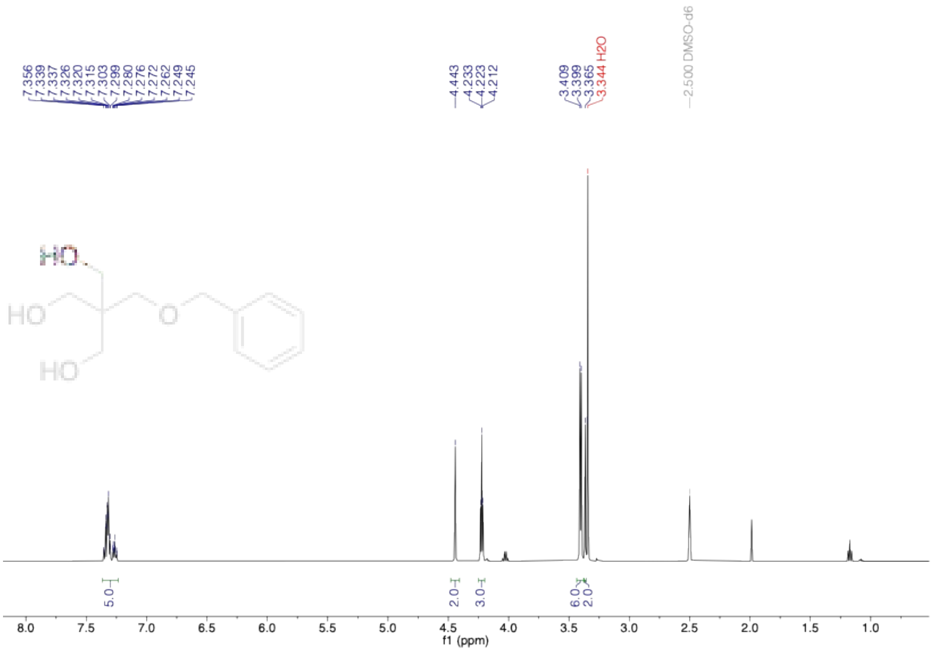

15

^{1}H NMR (400 MHz, $CDCl_3$)

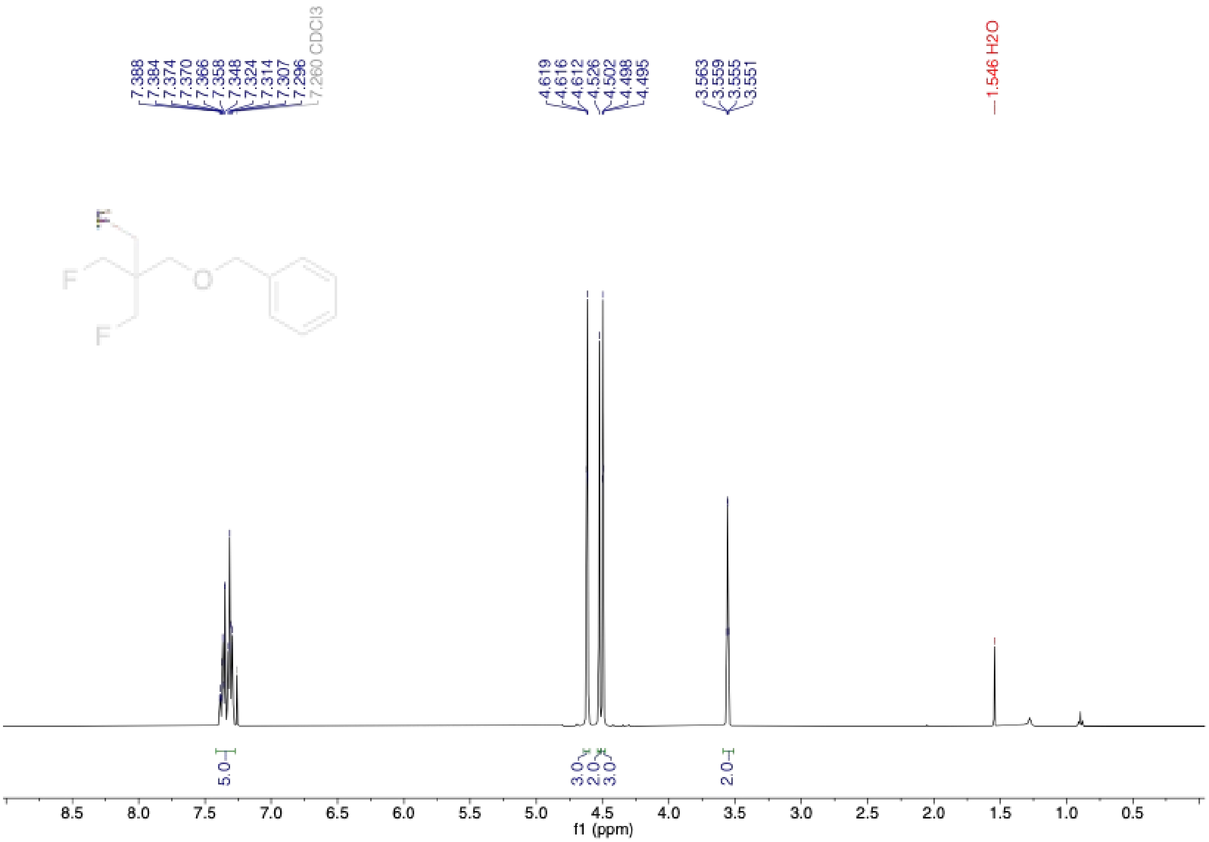

$^{19}F\{^{1}H\}$ NMR (470 MHz, $CDCl_3$)

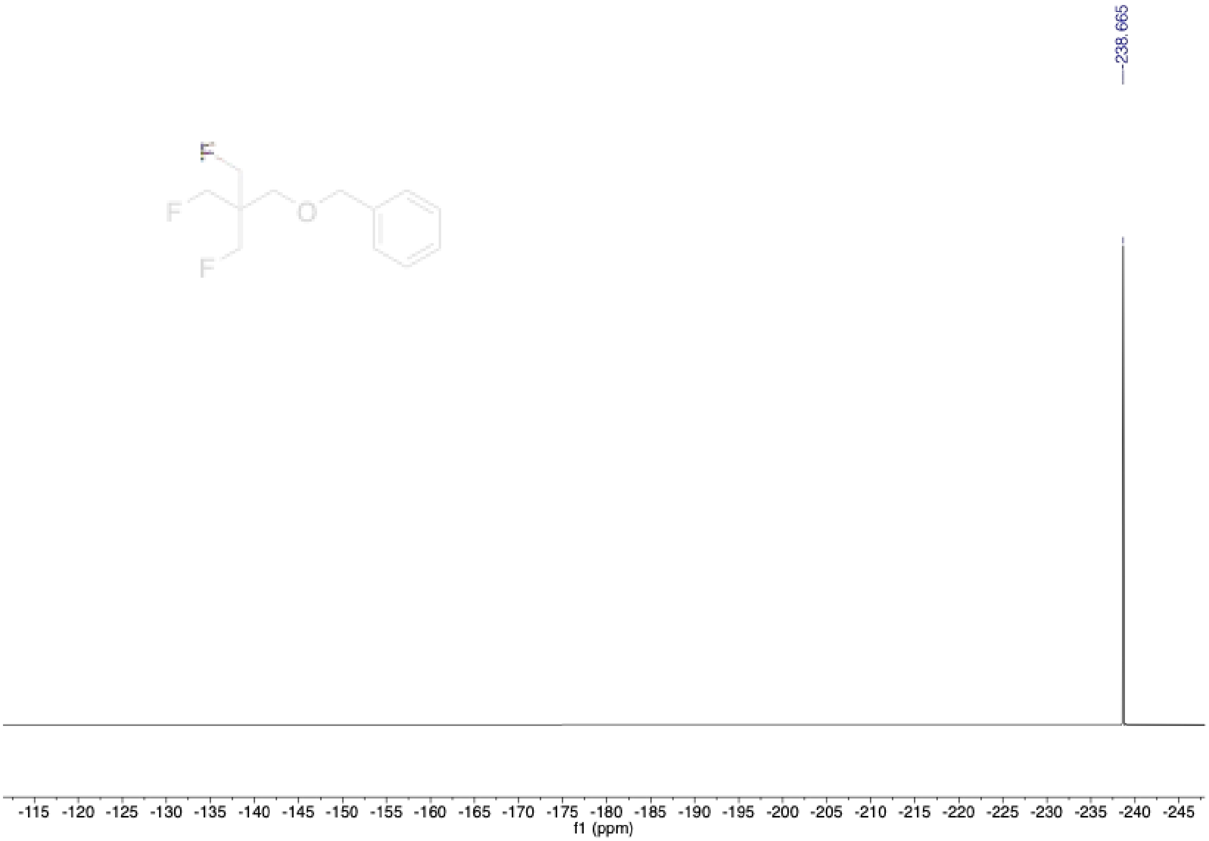

$^{13}C\{^{1}H\}$ NMR (126 MHz, $CDCl_3$)

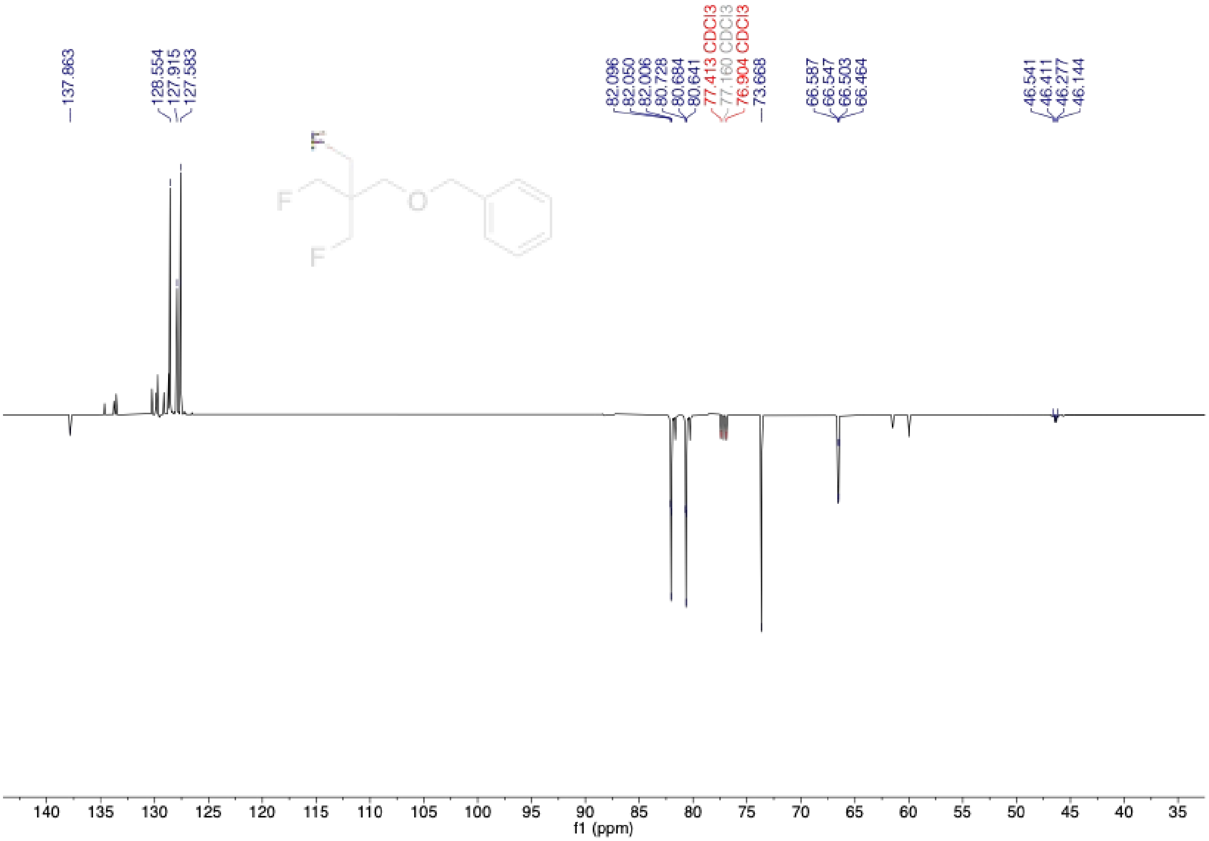

16

^{1}H NMR (400 MHz, $CDCl_3$)

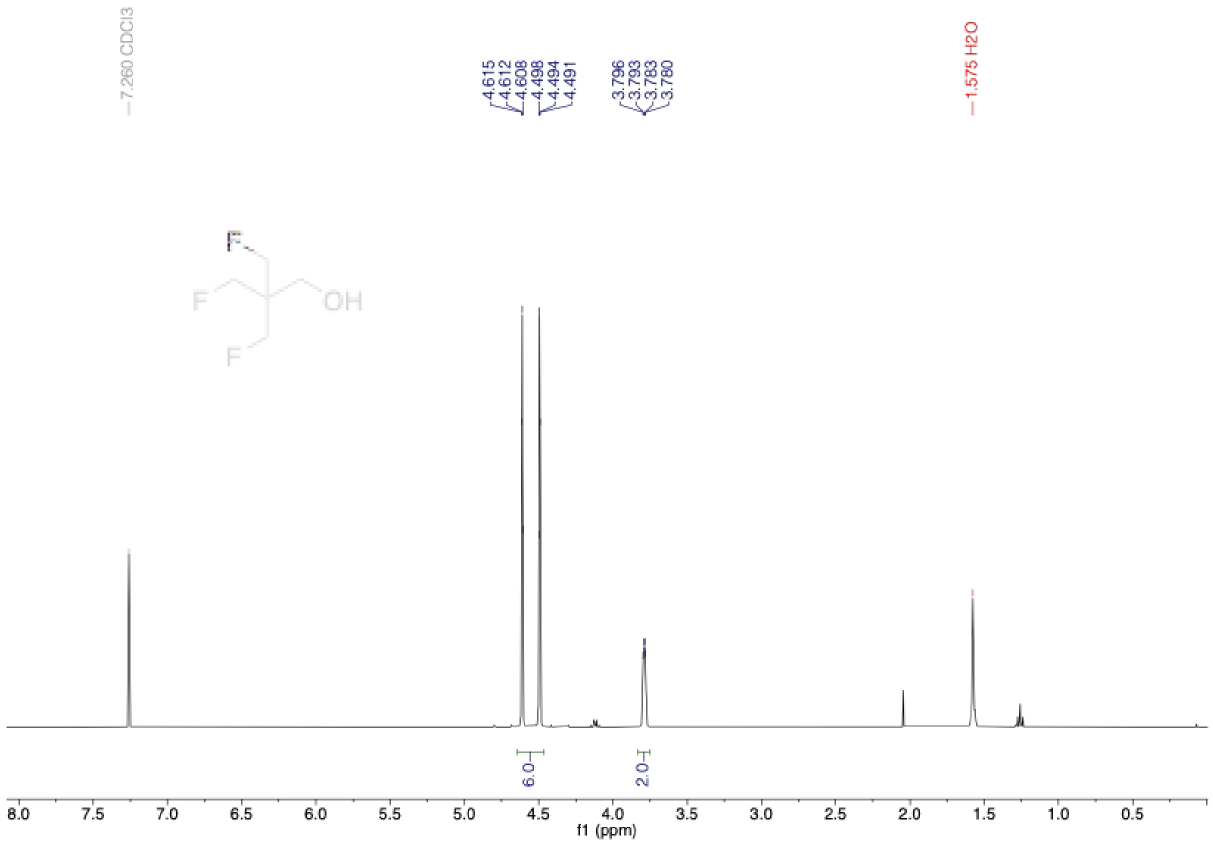

$^{19}F\{^{1}H\}$ NMR (377 MHz, $CDCl_3$)

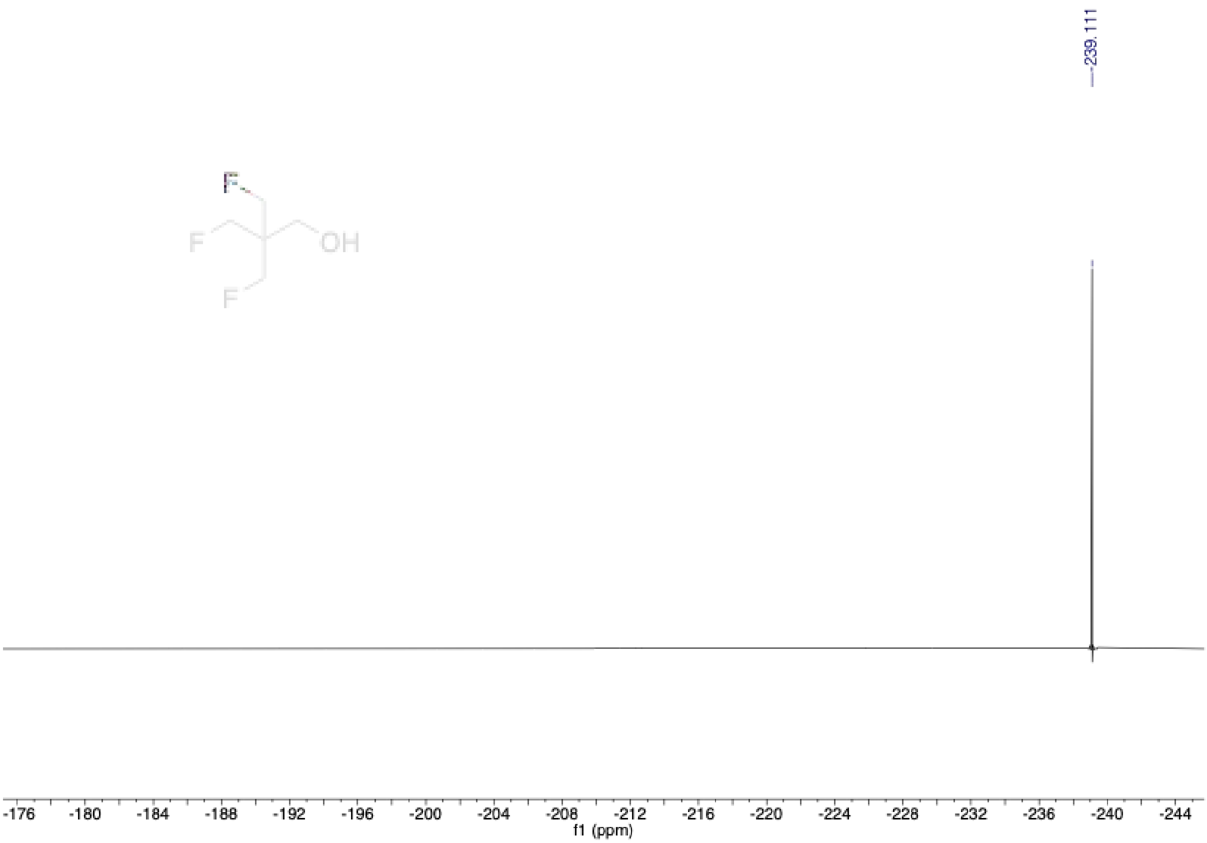

$^{13}C\{^{1}H\}$ NMR (176 MHz, $CDCl_3$)

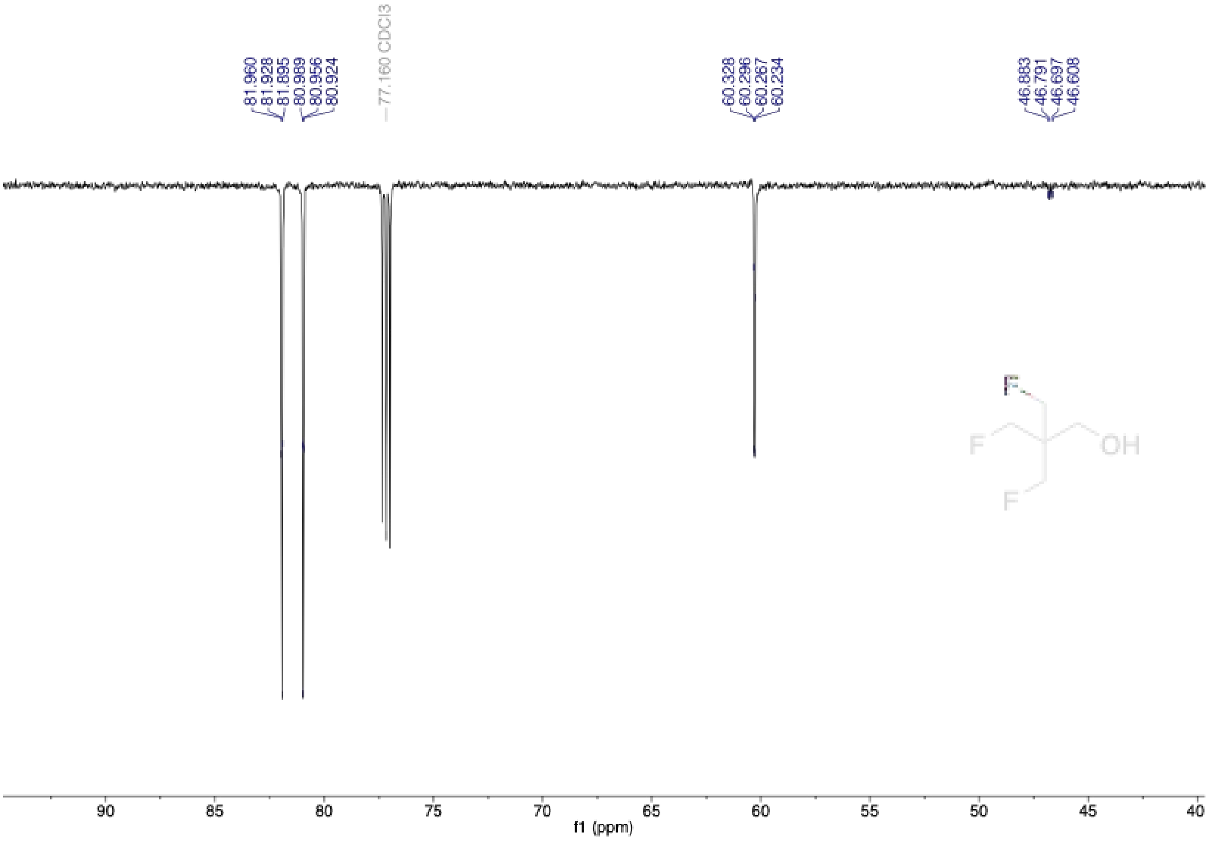

9

^{1}H NMR (400 MHz, $CDCl_3$)

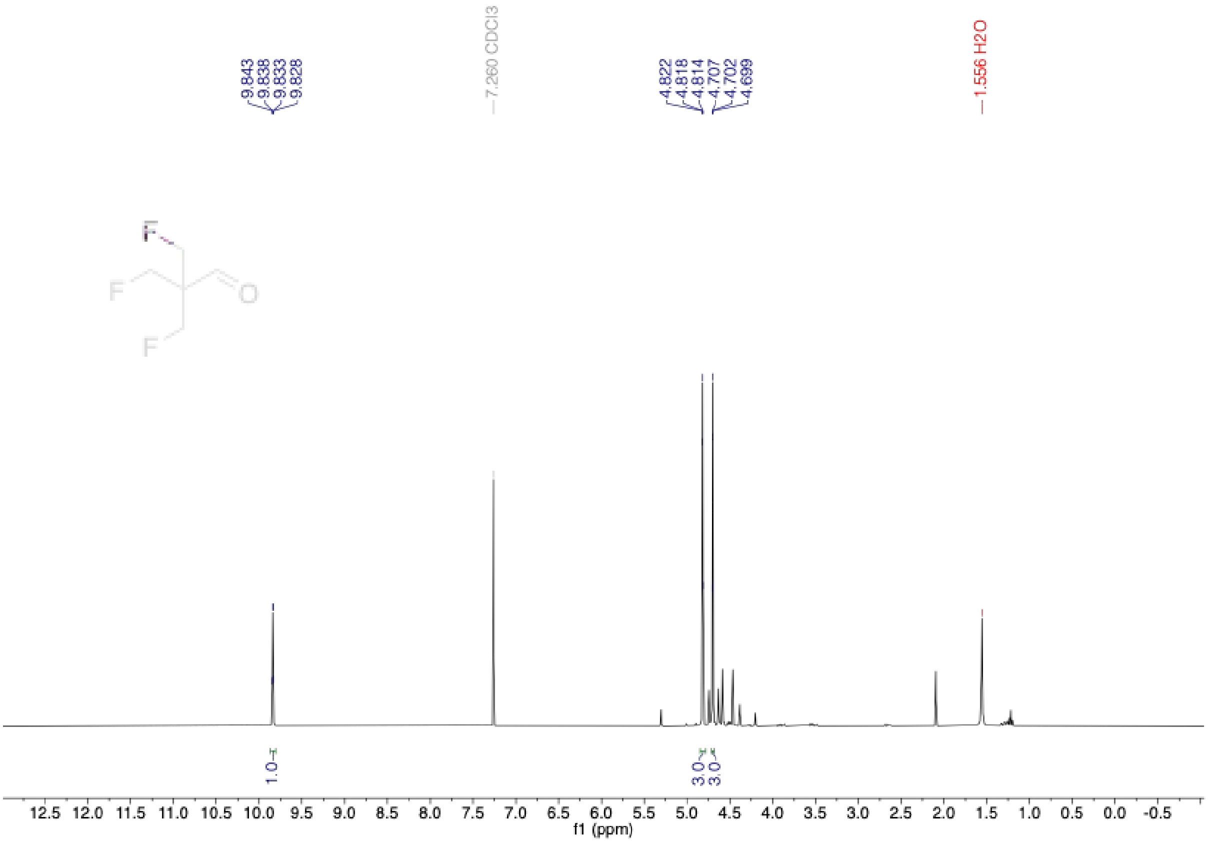

$^{19}F\{^{1}H\}$ NMR (470 MHz, $CDCl_3$)

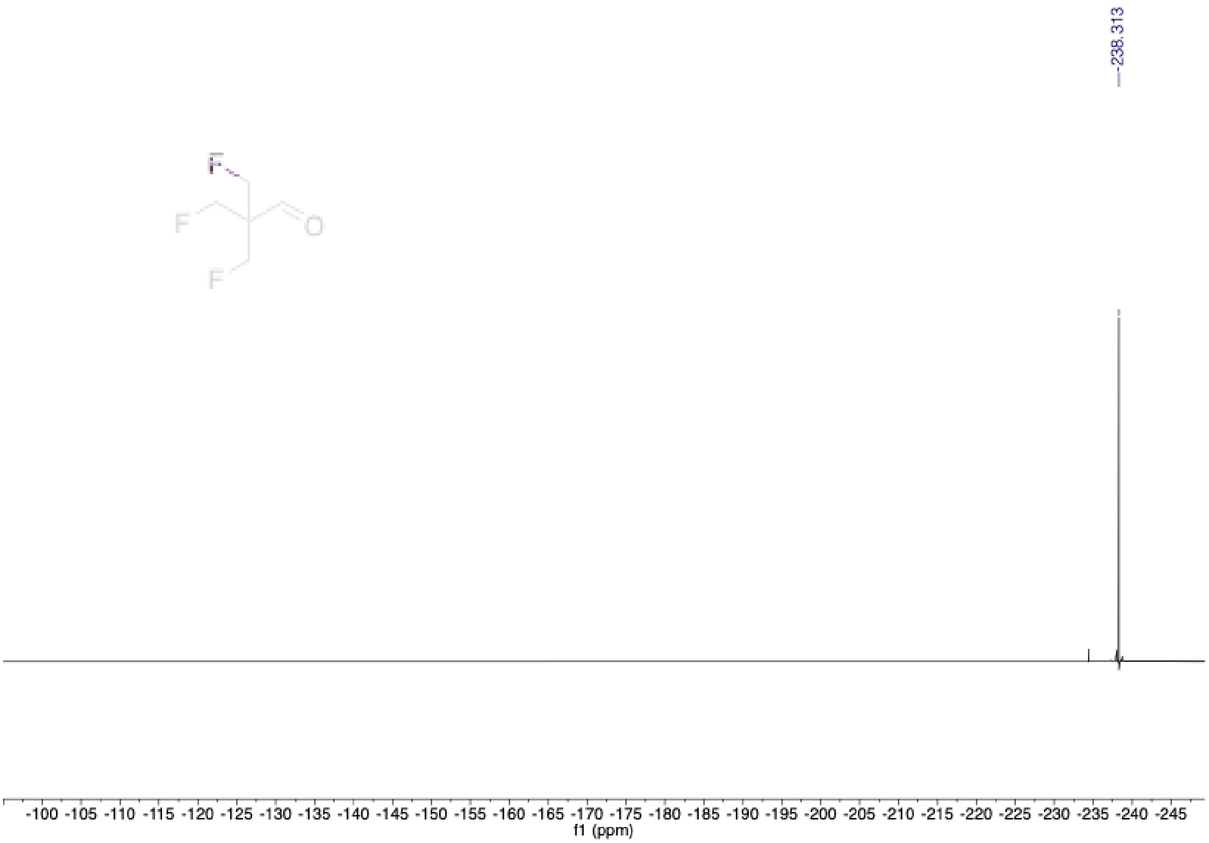

$^{13}C\{^{1}H\}$ NMR (126 MHz, $CDCl_3$)

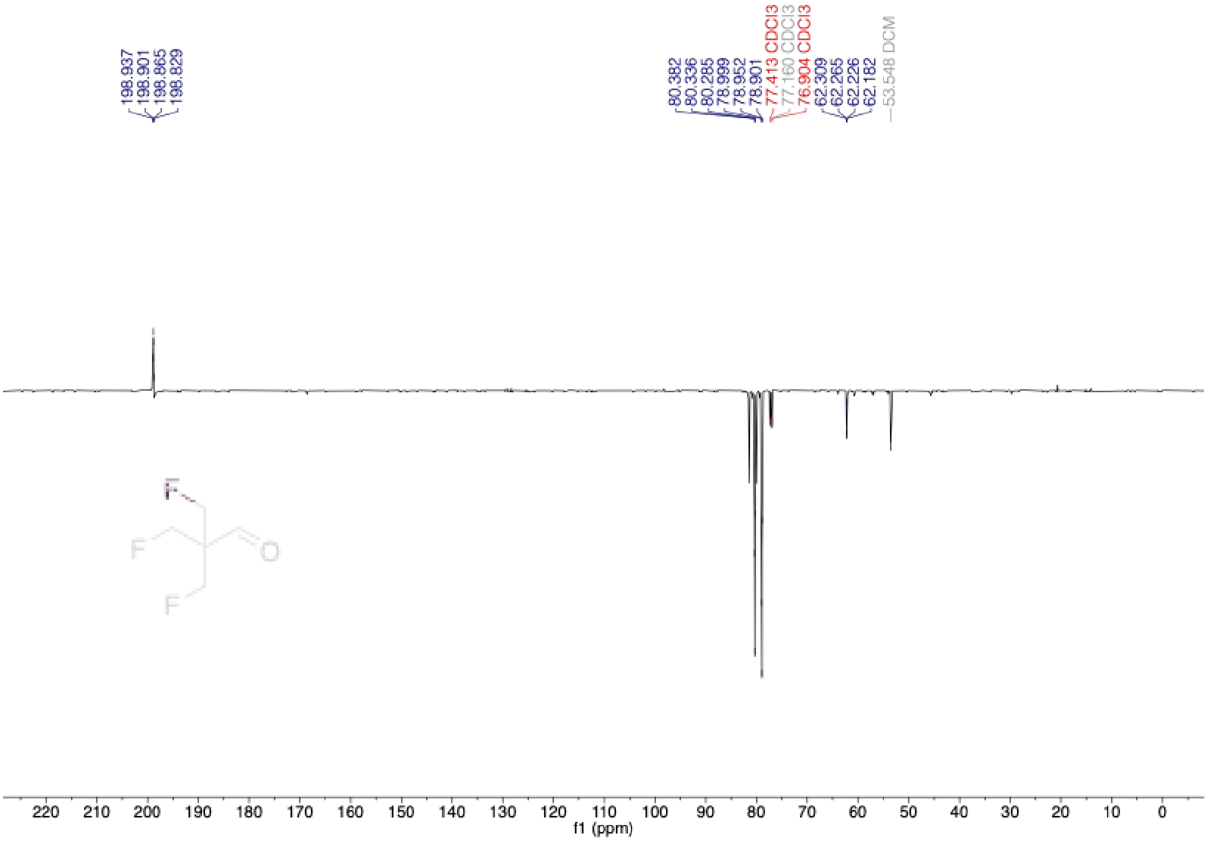

18

^{1}H NMR (500 MHz, $CDCl_3$)

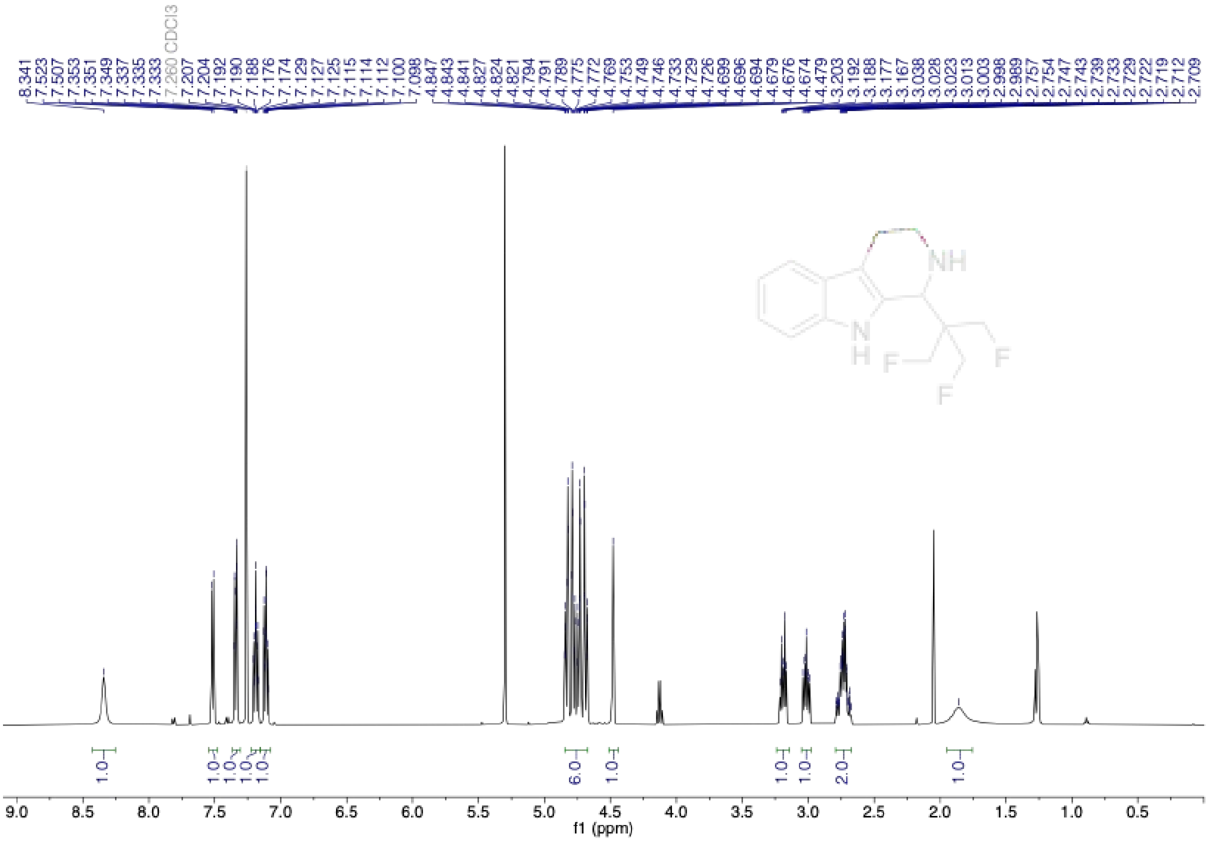

^{19}F{^{1}H} NMR (470 MHz, $CDCl_3$)

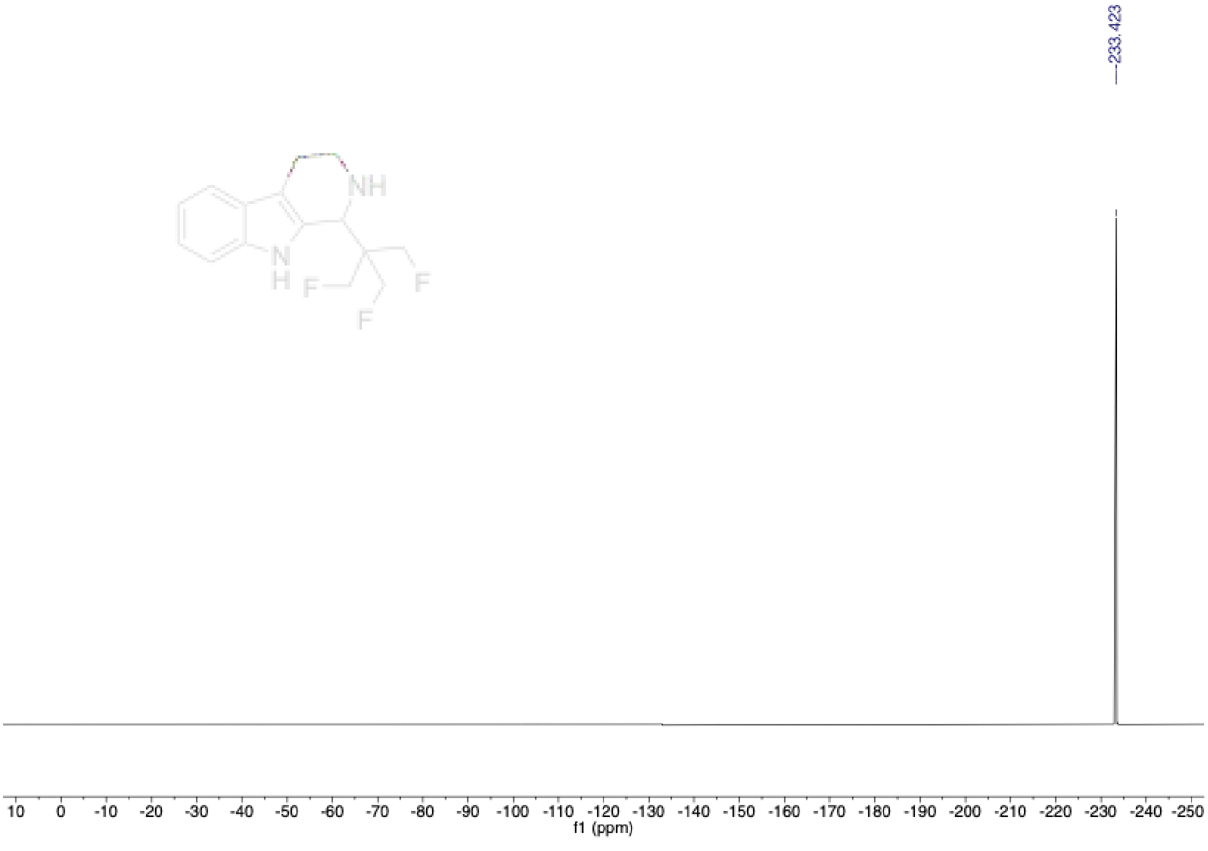

$^{13}C\{^{1}H\}$ NMR (126 MHz, $CDCl_3$)

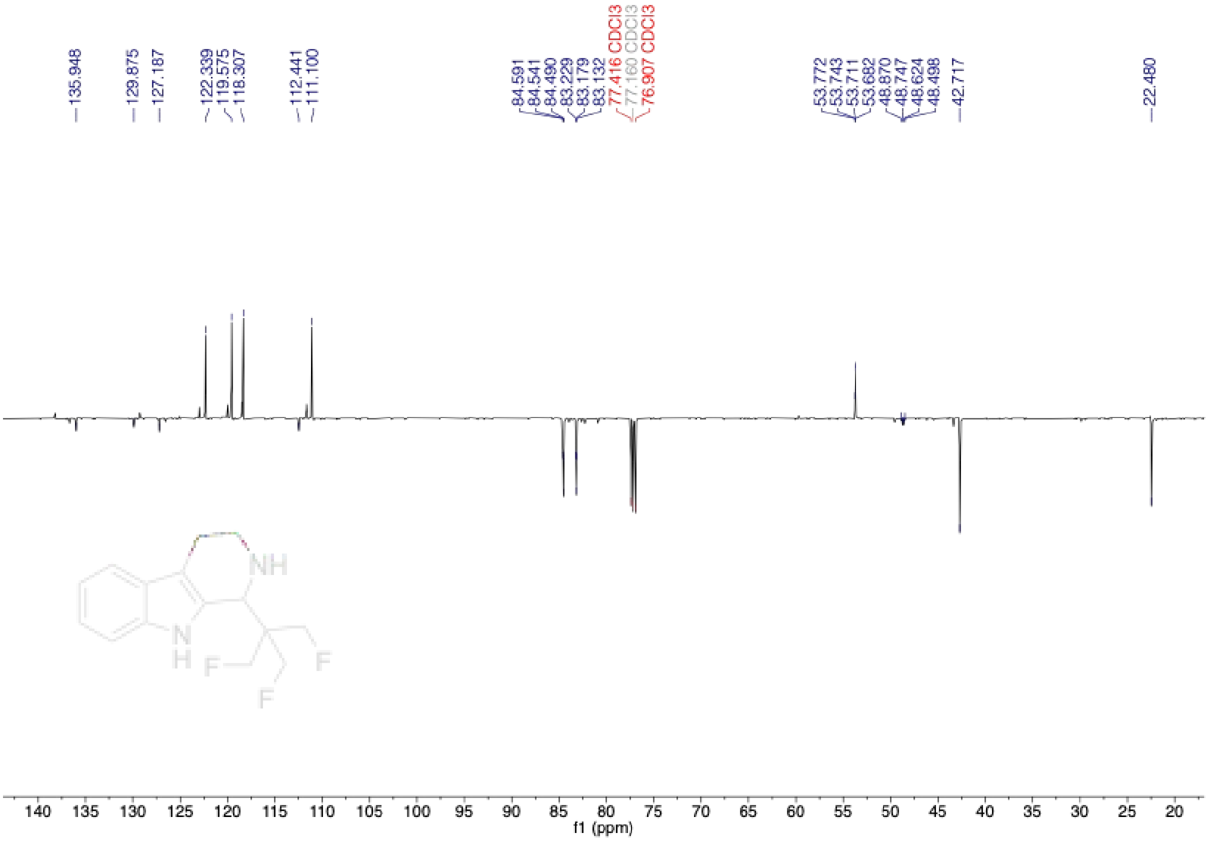

20

^{1}H NMR (500 MHz, DMSO)

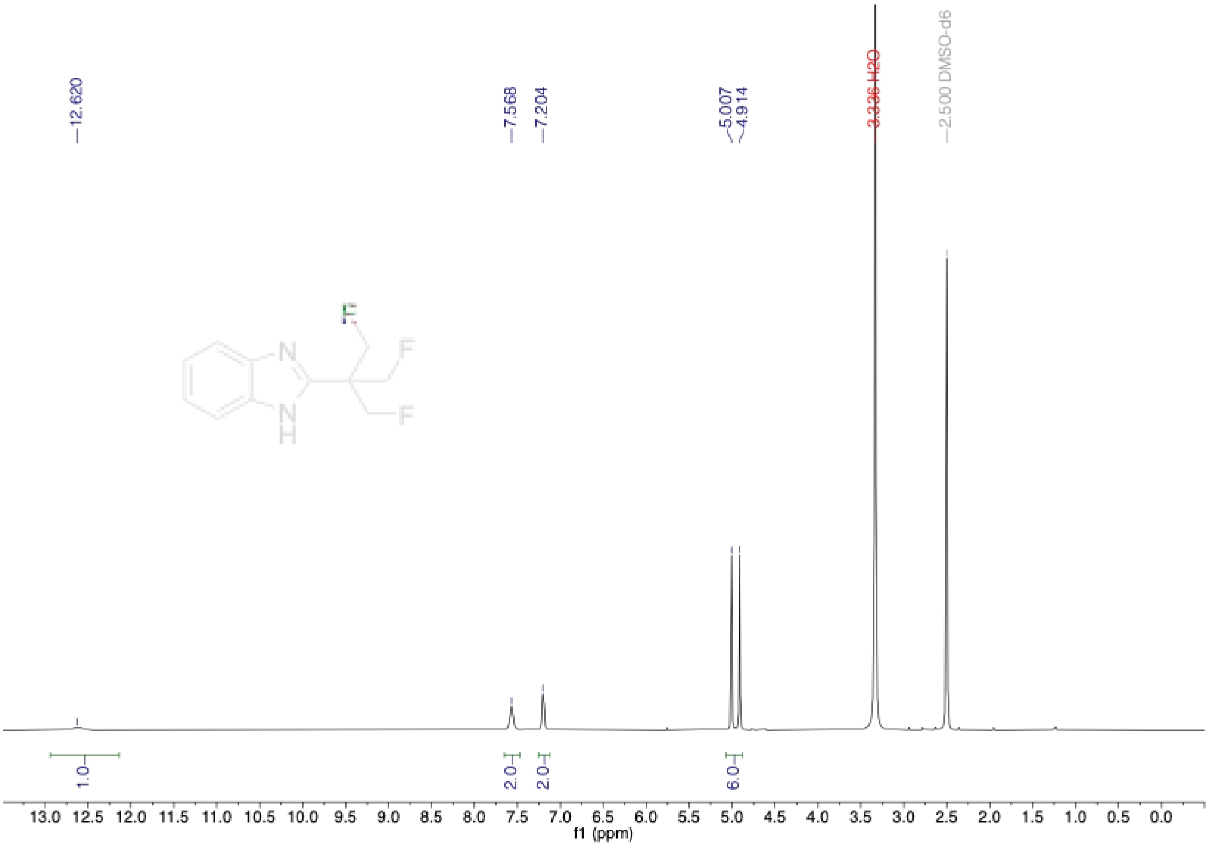

$^{19}F\{^{1}H\}$ NMR (470 MHz, DMSO)

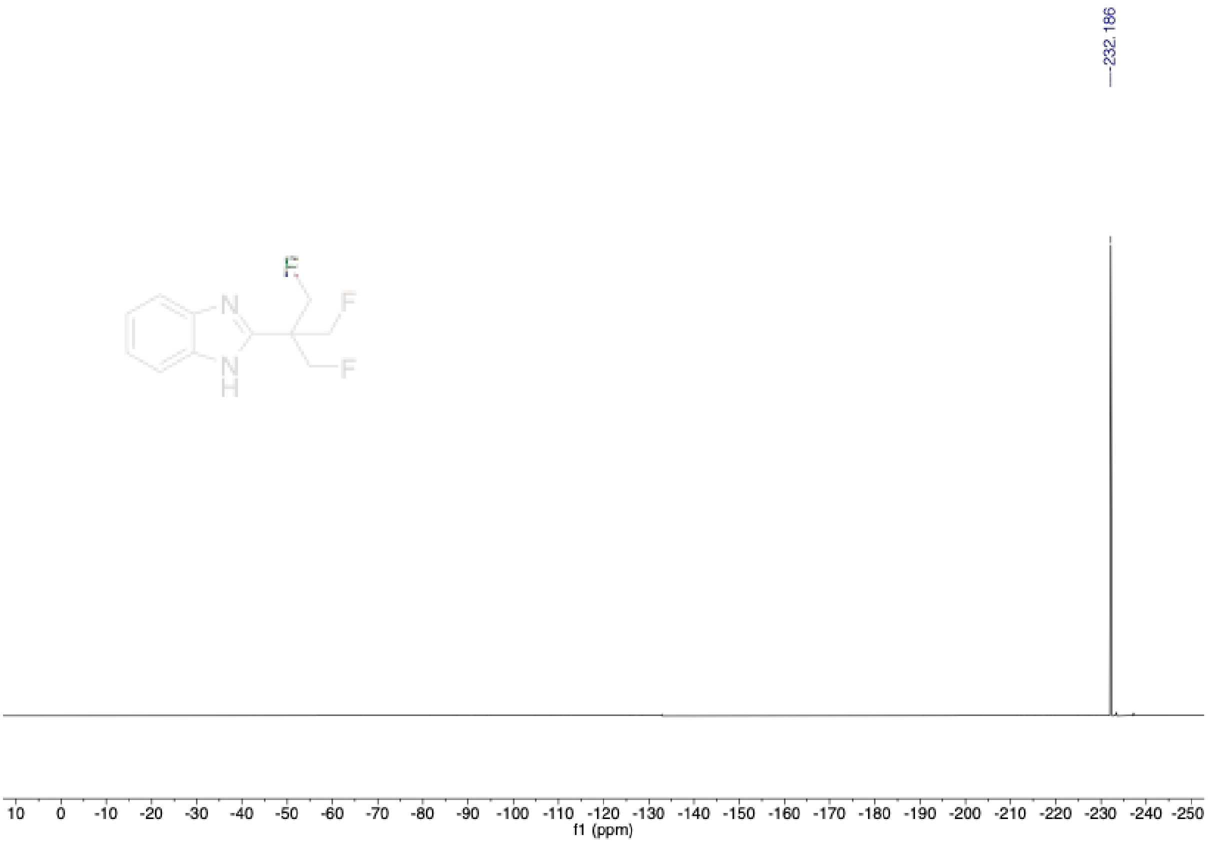

$^{13}C\{^{1}H\}$ NMR (126 MHz, DMSO)

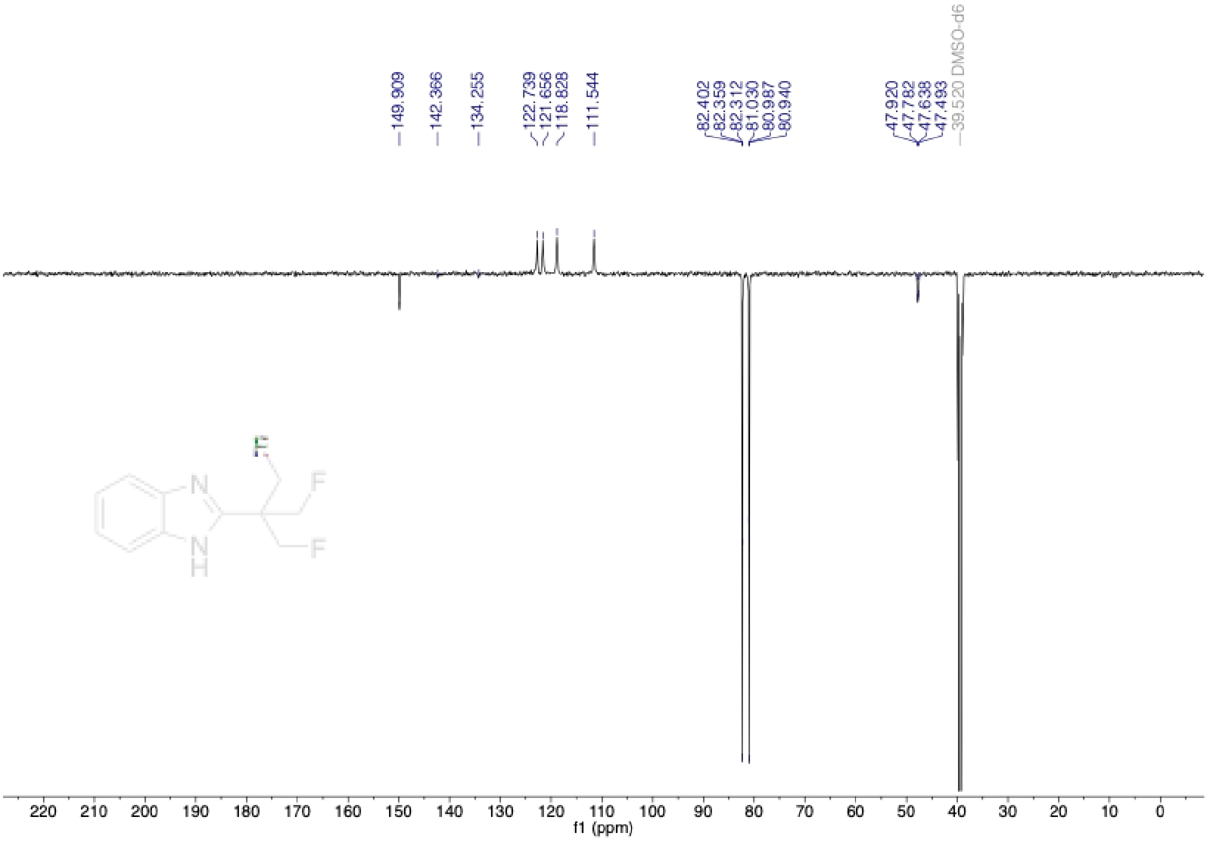

22

^{1}H NMR (400 MHz, $CDCl_3$)

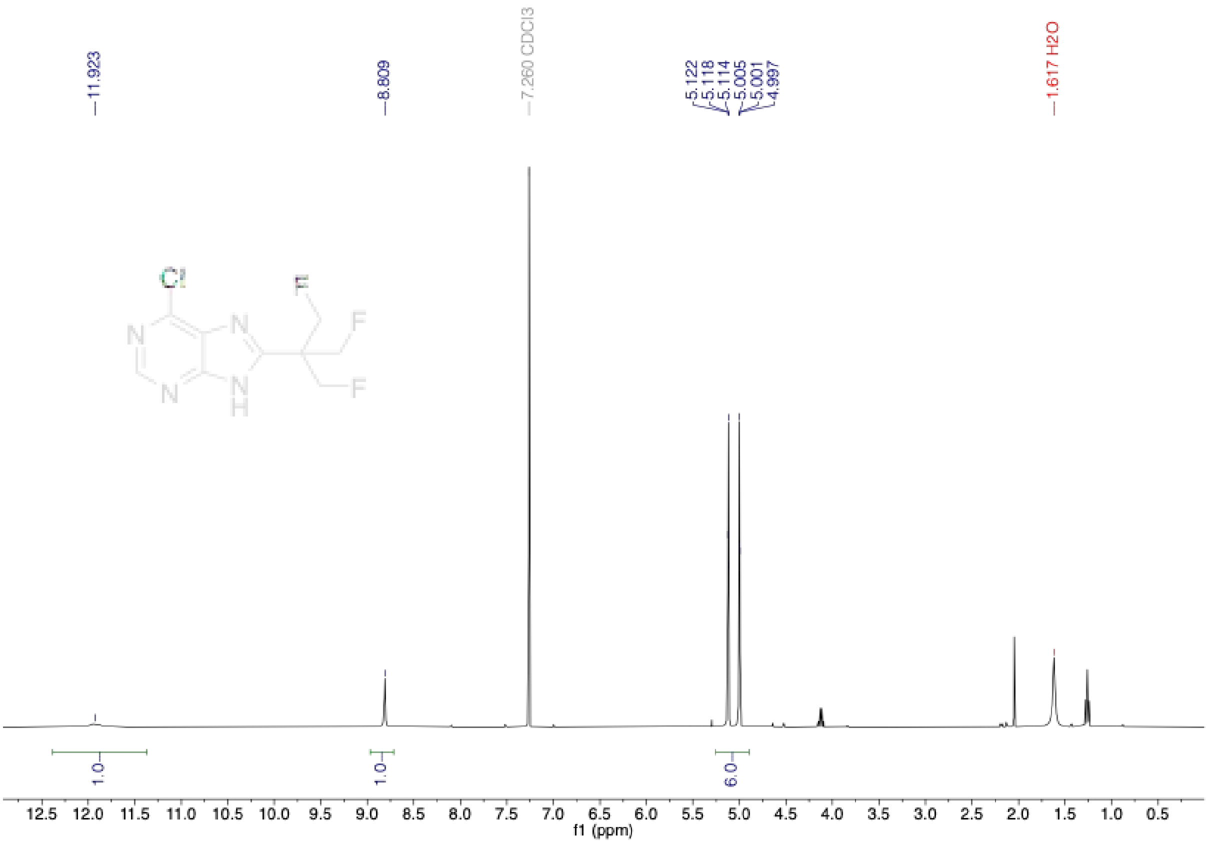

$^{19}F\{^{1}H\}$ NMR (377 MHz, $CDCl_3$)

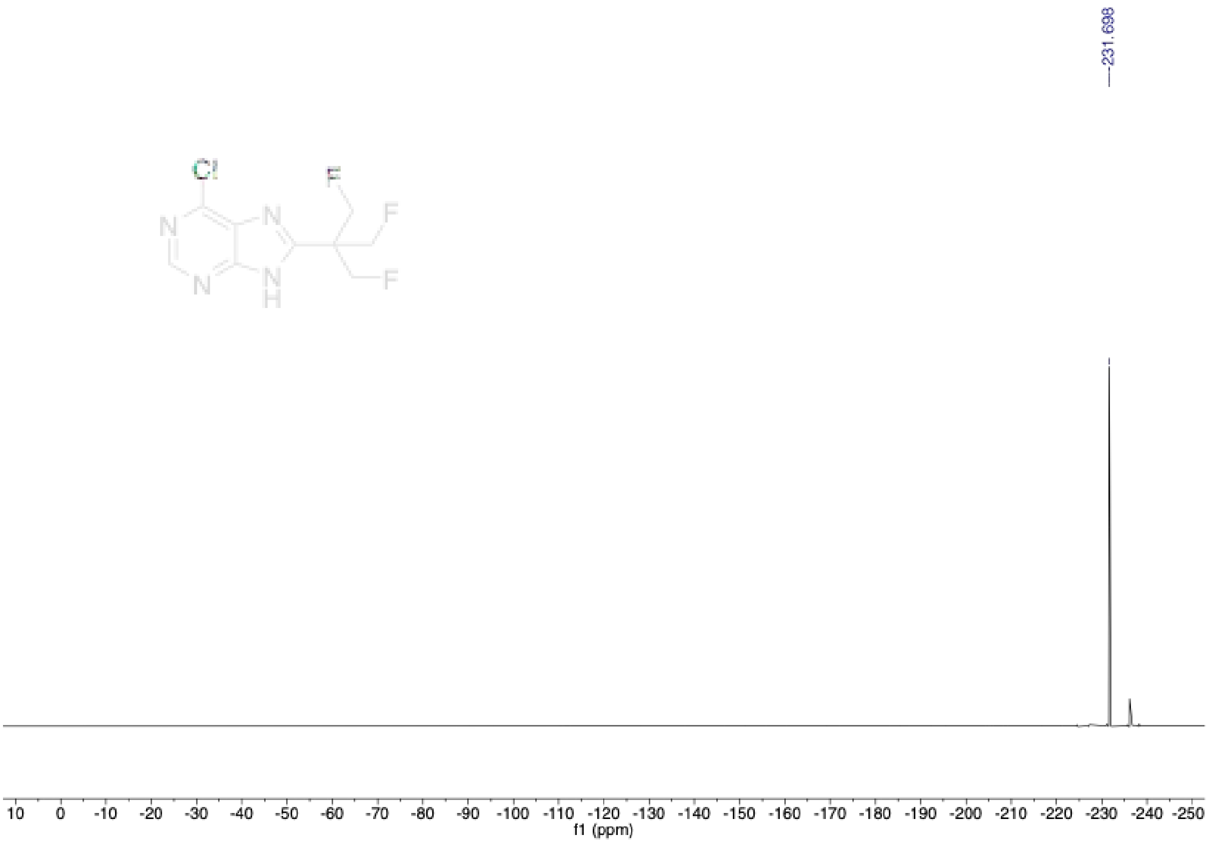

$^{13}C\{^{1}H\}$ NMR (126 MHz, DMSO)

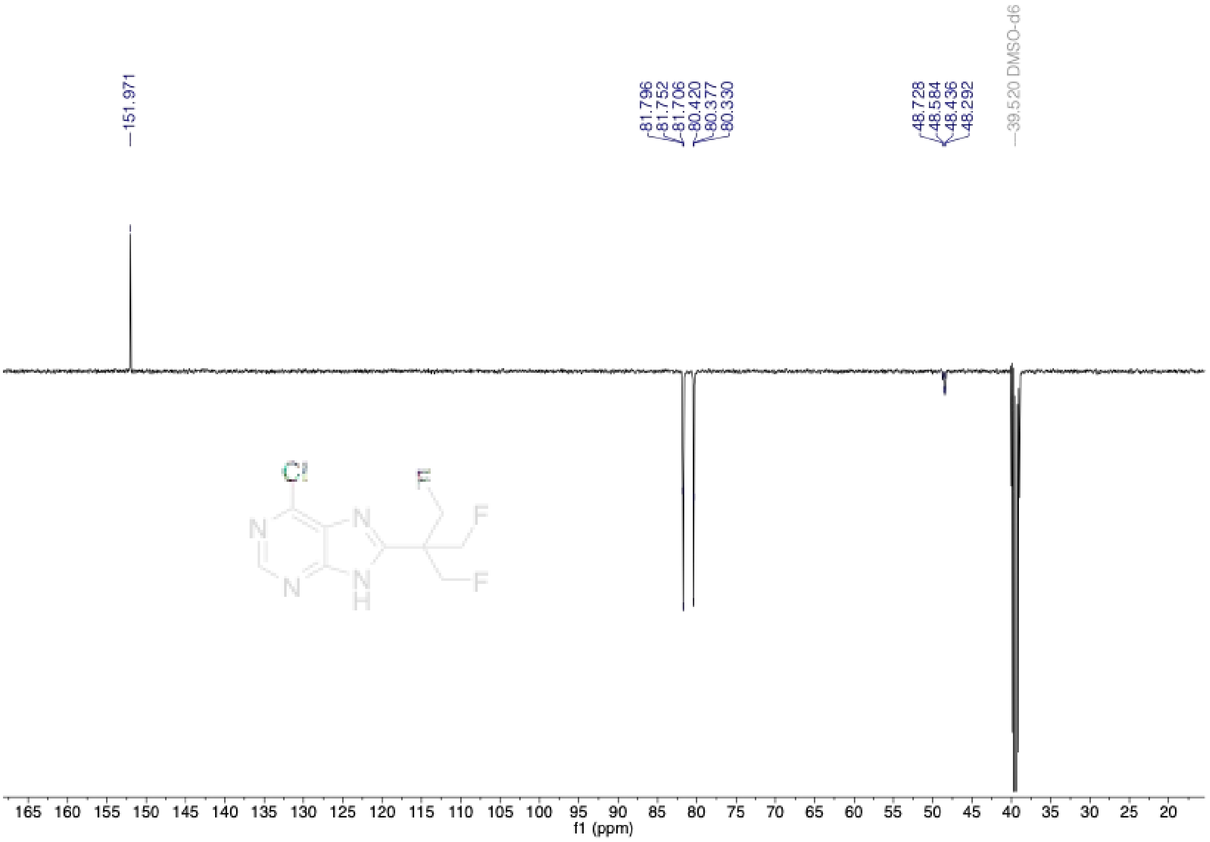

^{1}H-^{13}C HMBC NMR (DMSO)

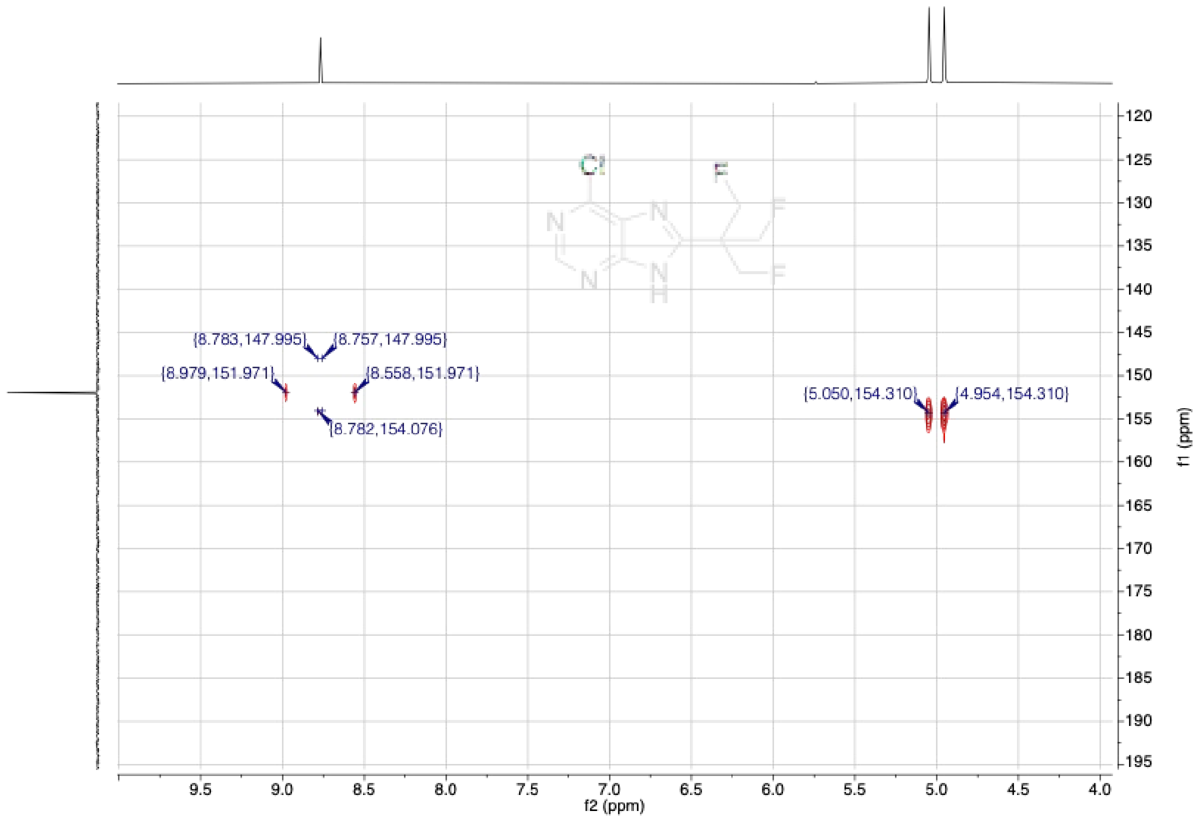

26

^{1}H NMR (500 MHz, $CDCl_3$)

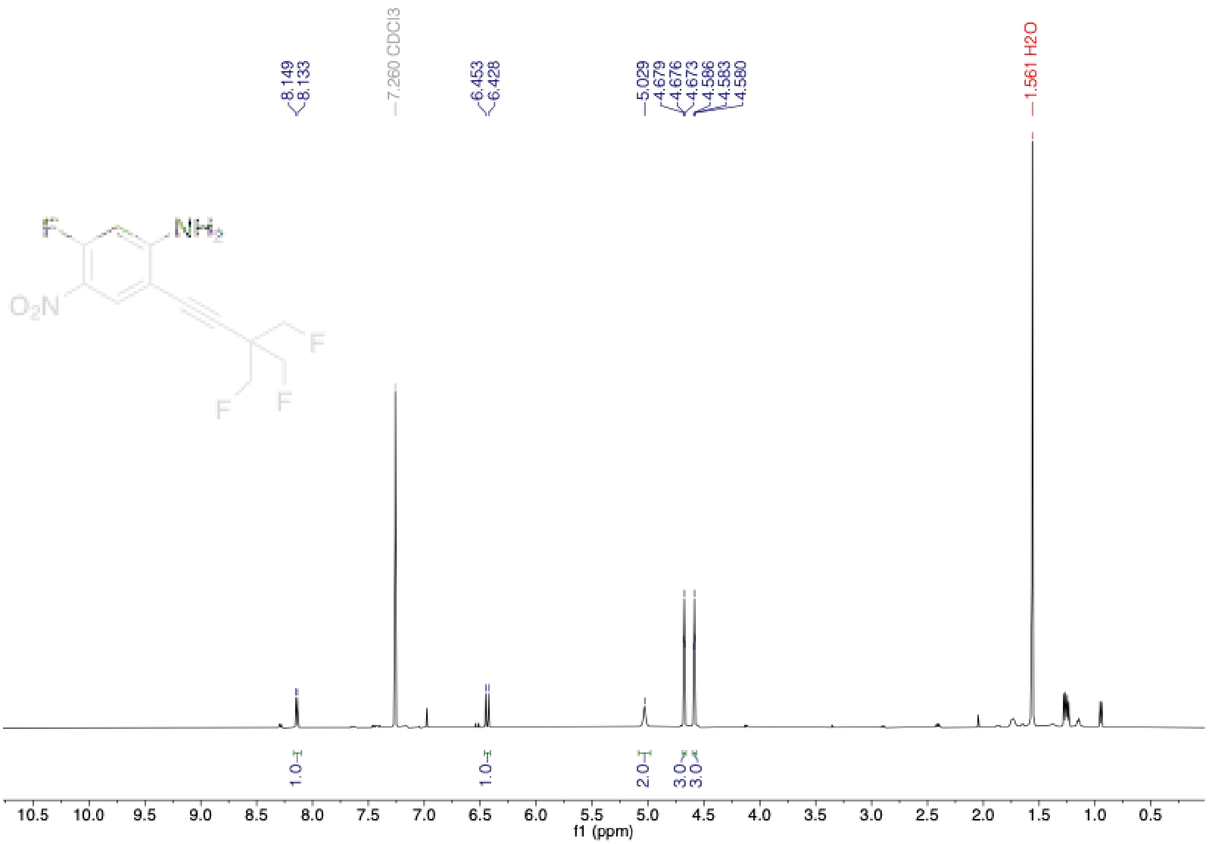

$^{19}F\{^1H\}$ NMR (470 MHz, $CDCl_3$)

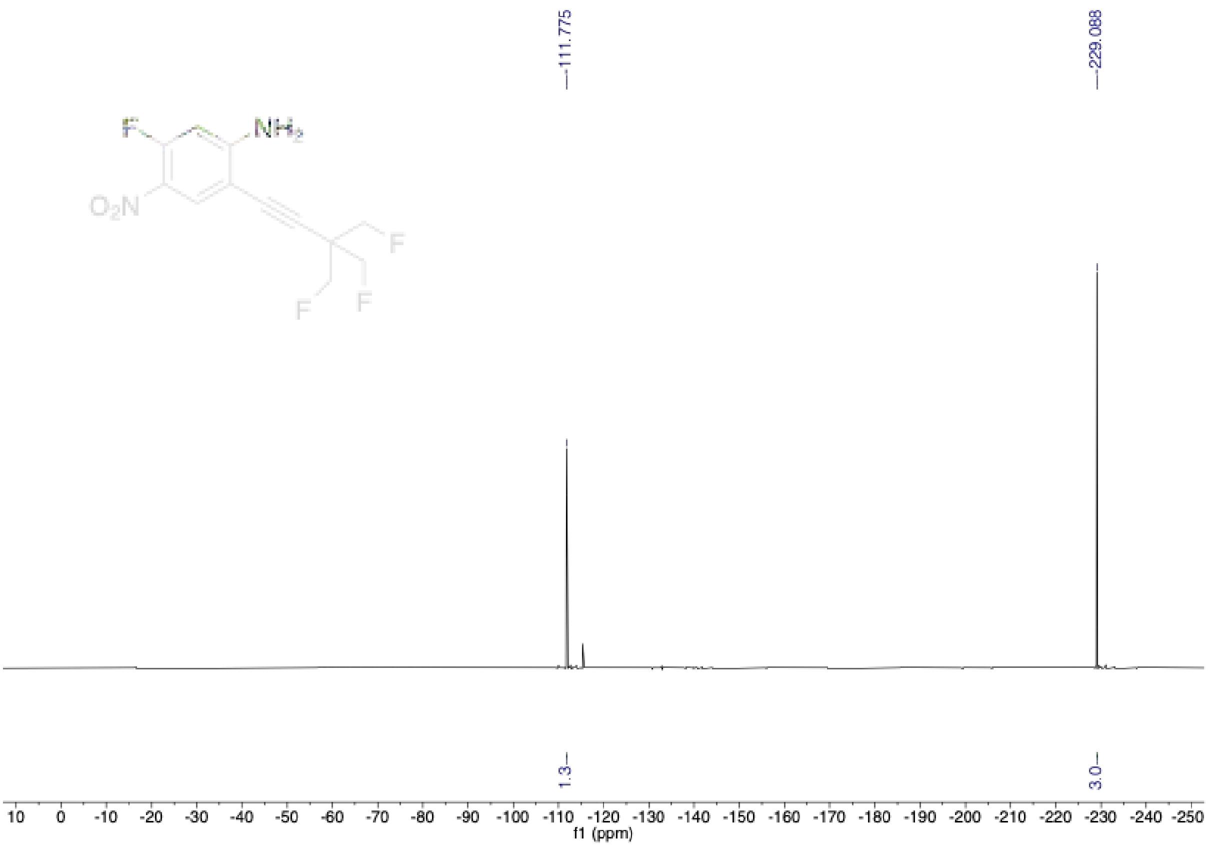

$^{13}C\{^1H\}$ NMR (126 MHz, $CDCl_3$)

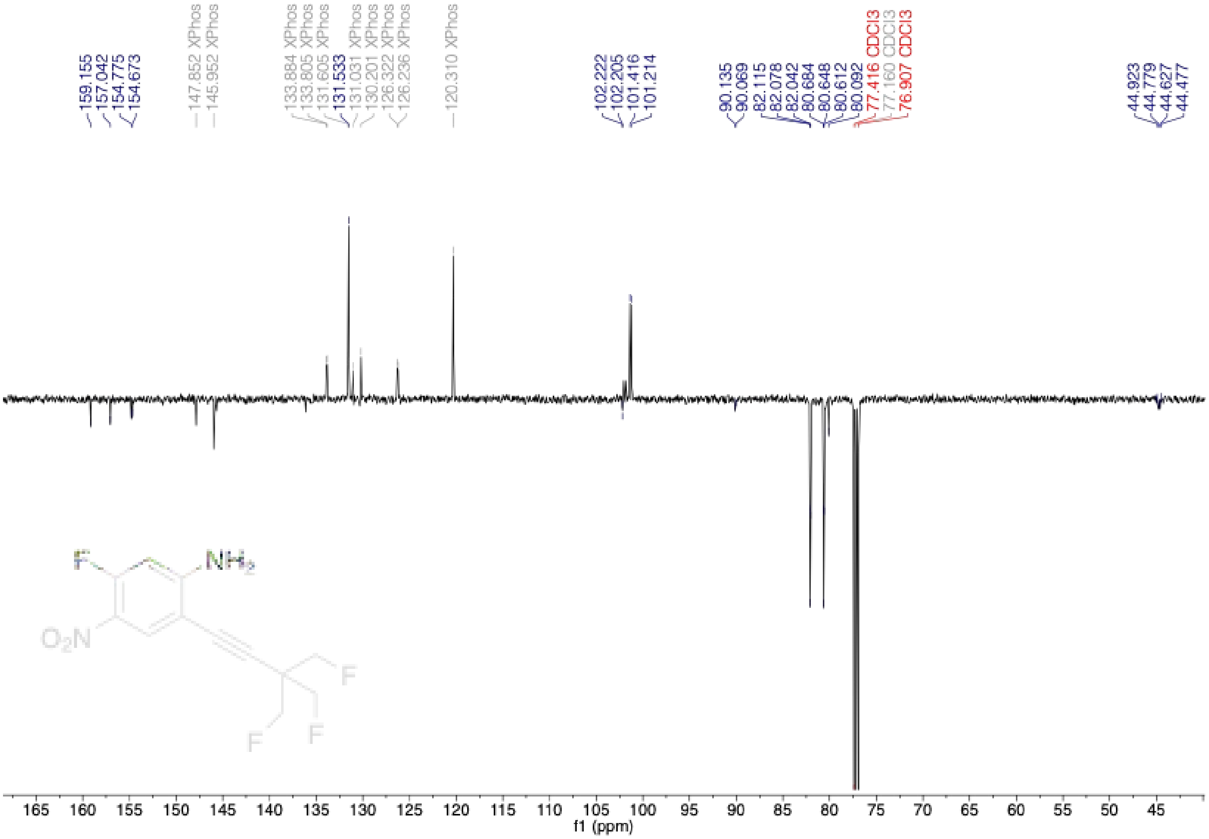

^{1}H-^{13}C HMBC NMR ($CDCl_3$)

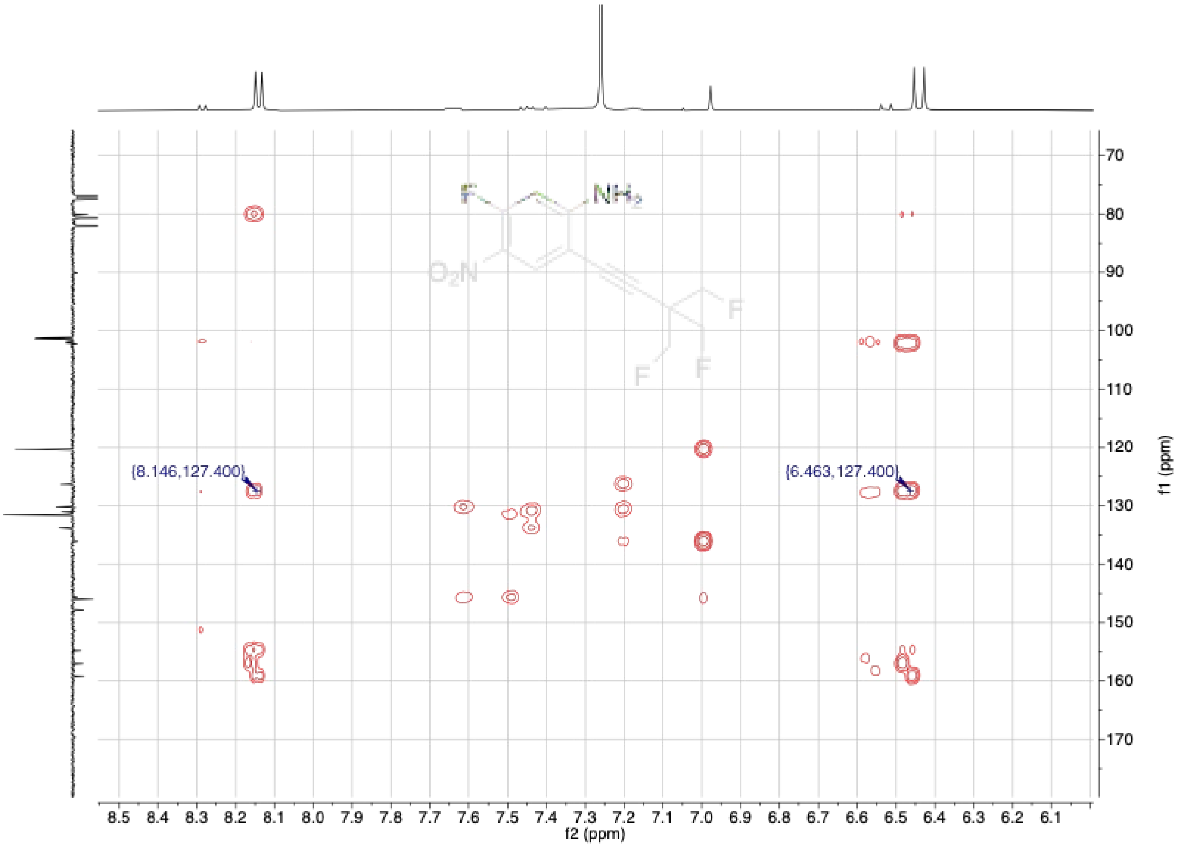

27

^{1}H NMR (400 MHz, $CDCl_3$)

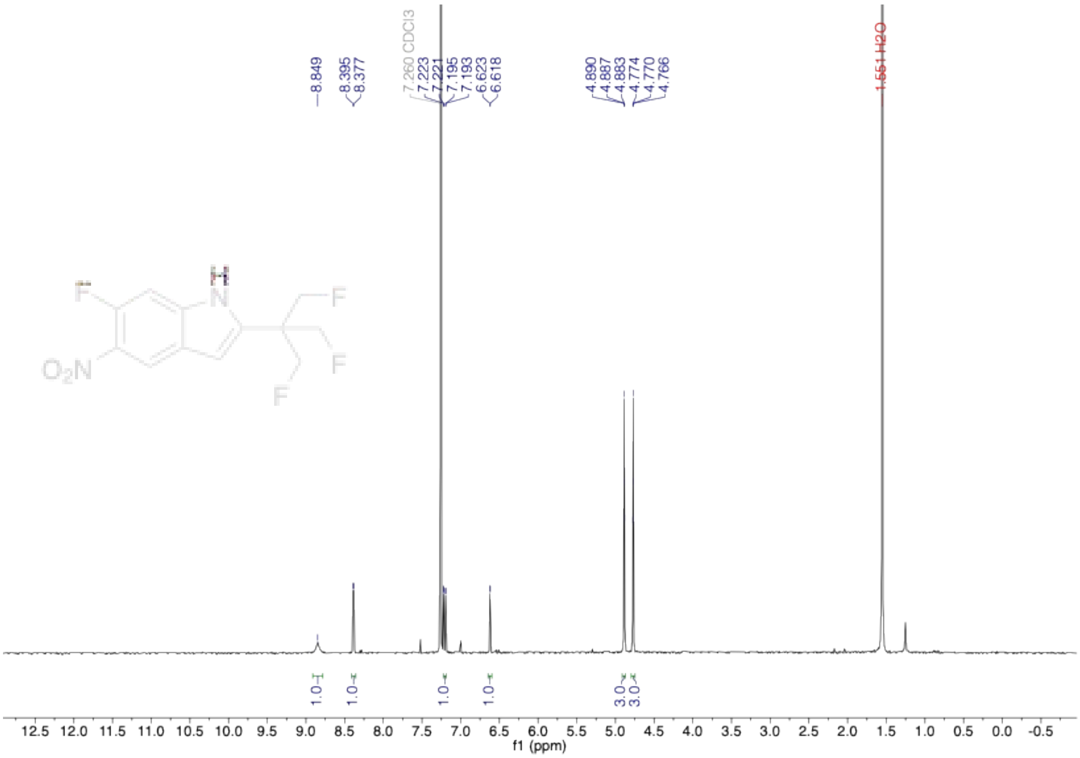

$^{19}F\{^{1}H\}$ NMR (377 MHz, $CDCl_3$)

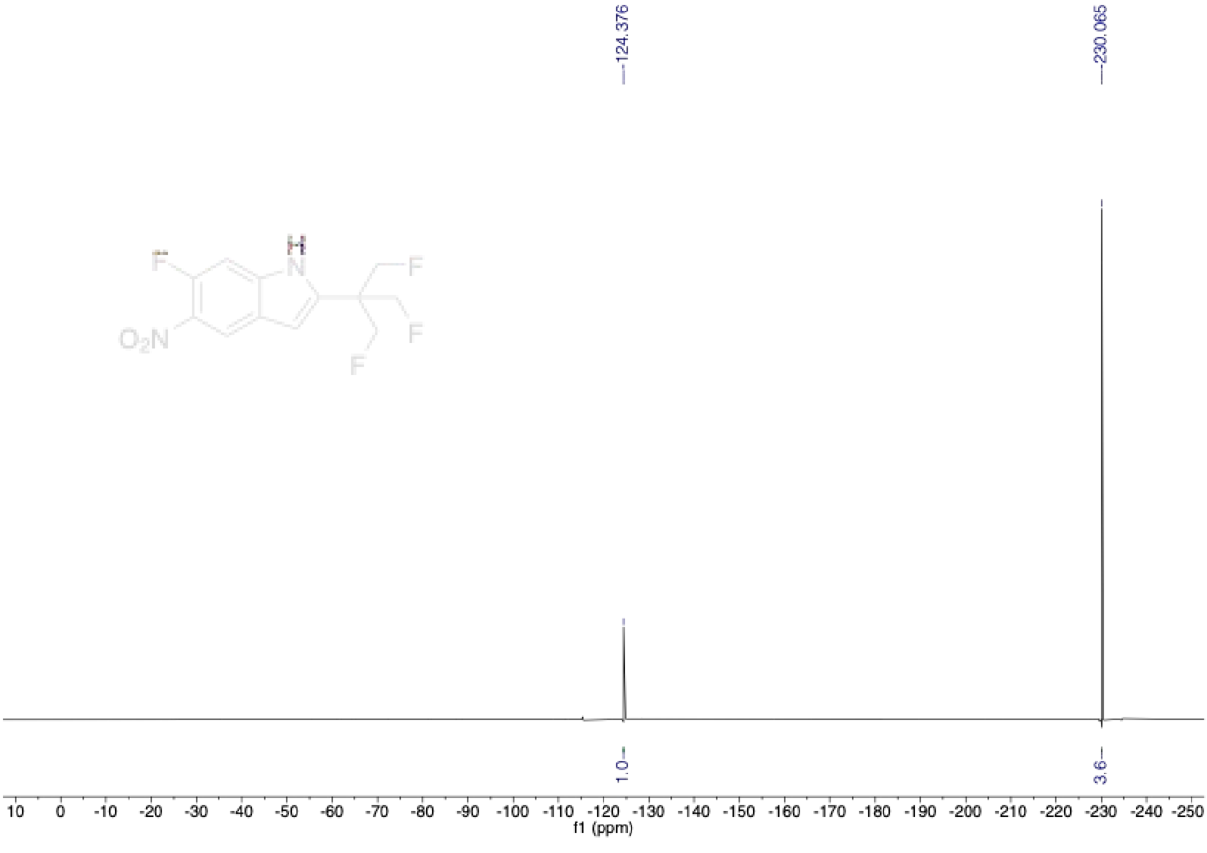

$^{13}C\{^1H\}$ NMR (126 MHz, $CDCl_3$)

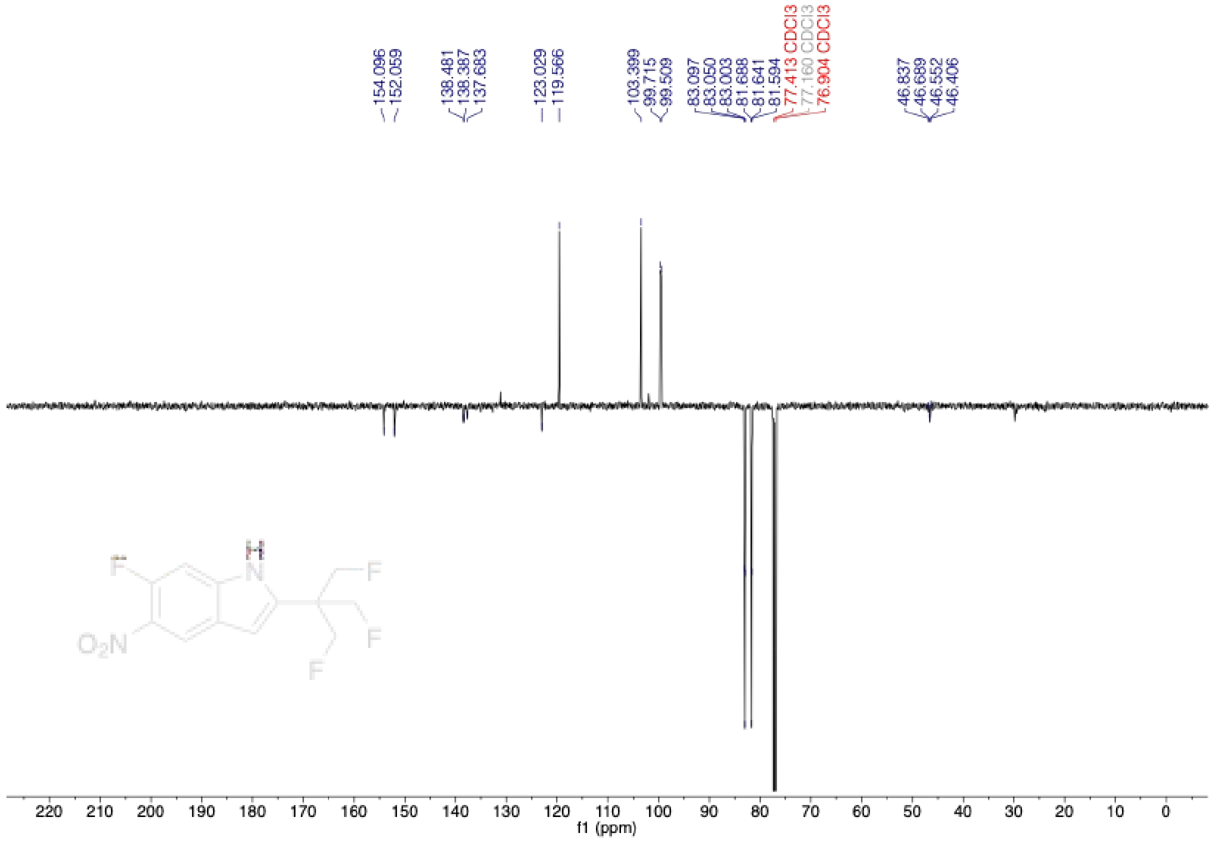

^1H-^{13}C HMBC NMR ($CDCl_3$)

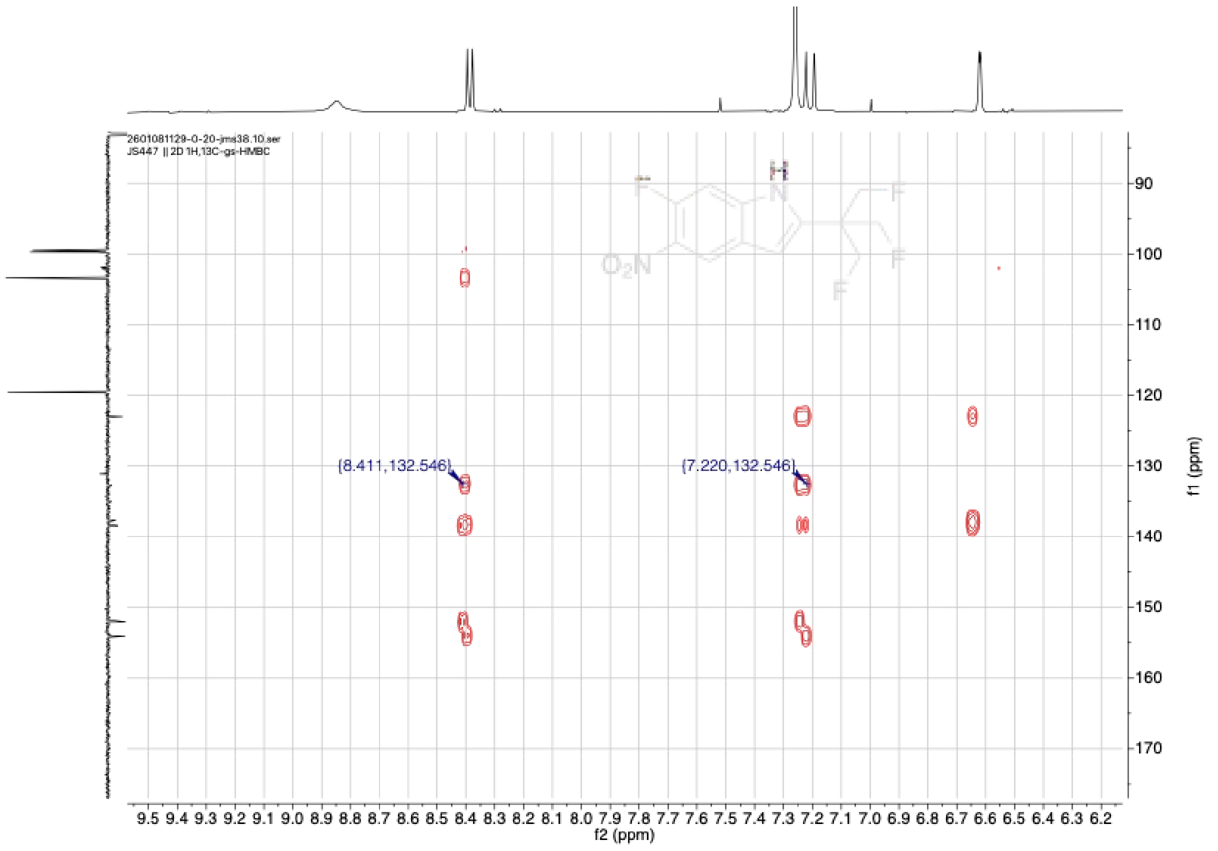

28

^1H NMR (400 MHz, $CDCl_3$)

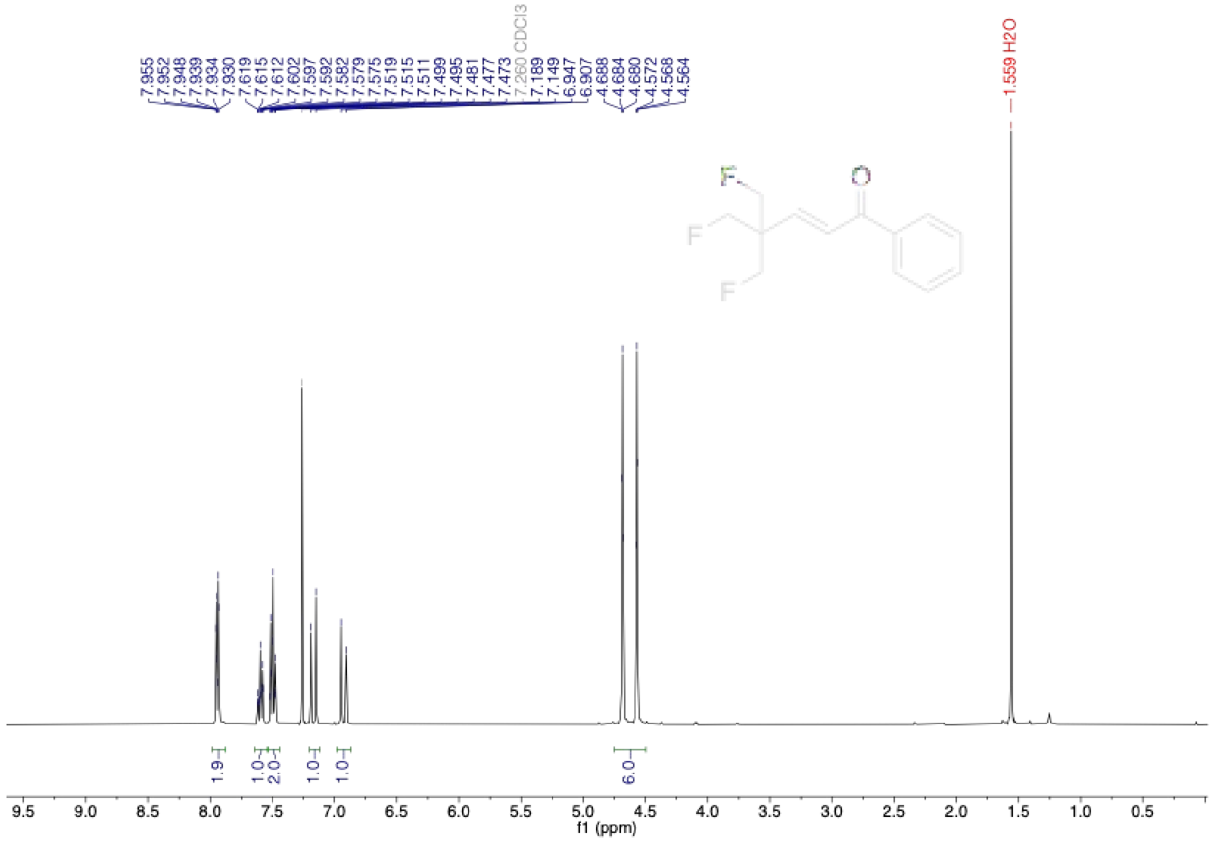

$^{19}F\{^{1}H\}$ NMR (377 MHz, $CDCl_3$)

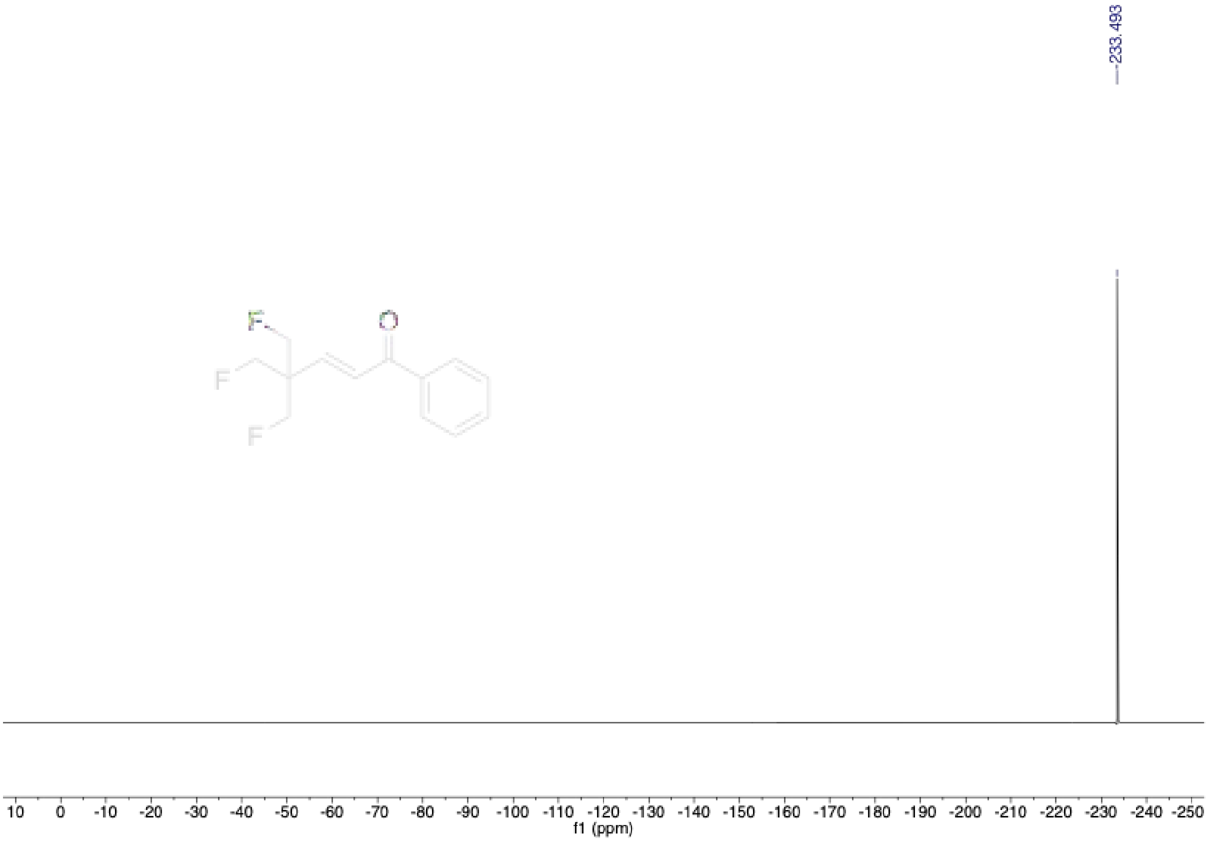

$^{13}C\{^{1}H\}$ NMR (126 MHz, $CDCl_3$)

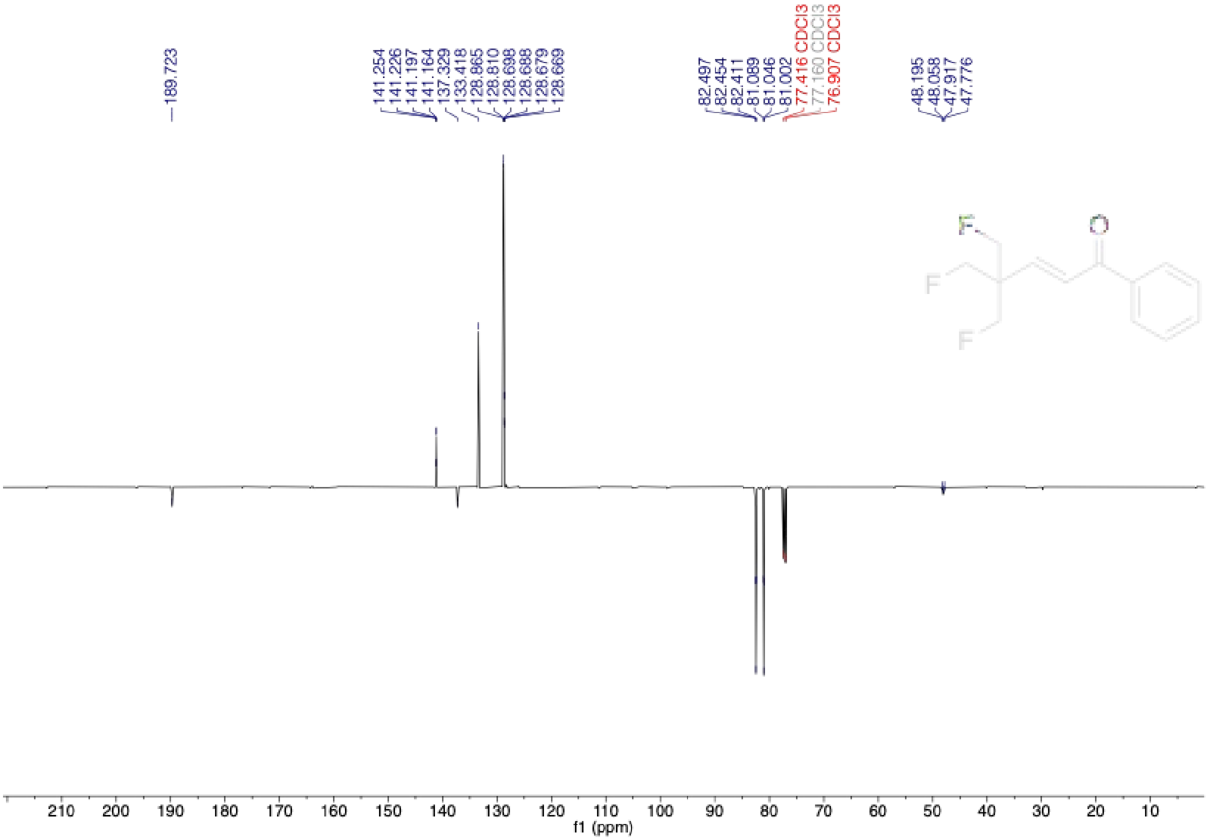

29

^{1}H NMR (500 MHz, $CDCl_3$)

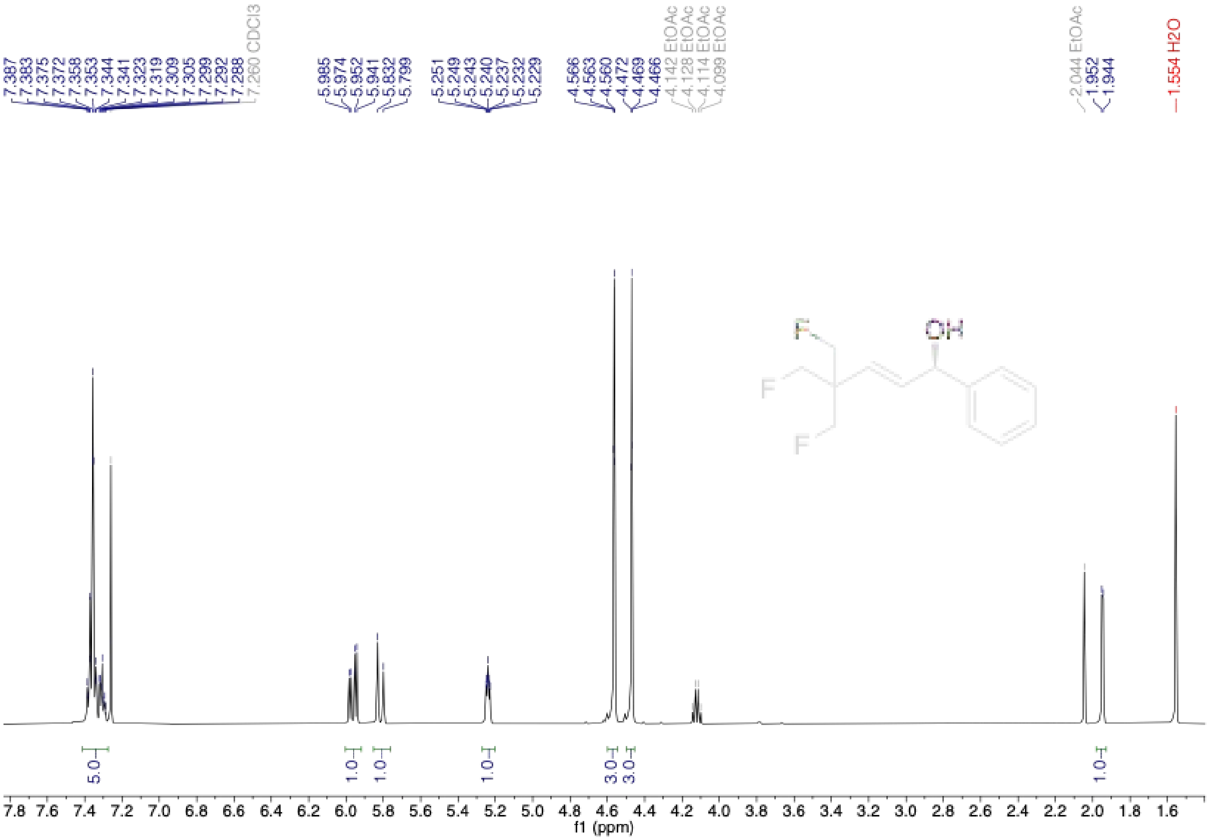

$^{19}F\{^{1}H\}$ NMR (470 MHz, $CDCl_3$)

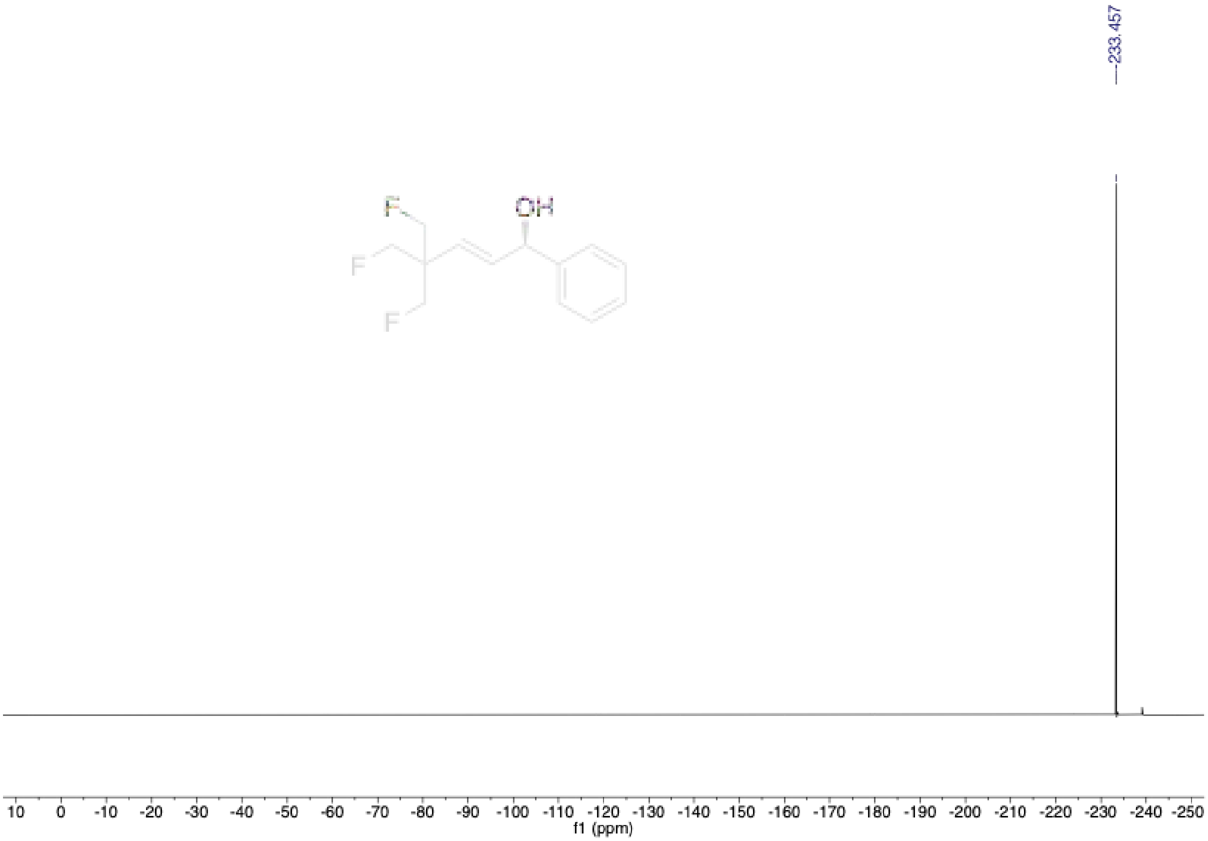

$^{13}C\{^{1}H\}$ NMR (126 MHz, $CDCl_3$)

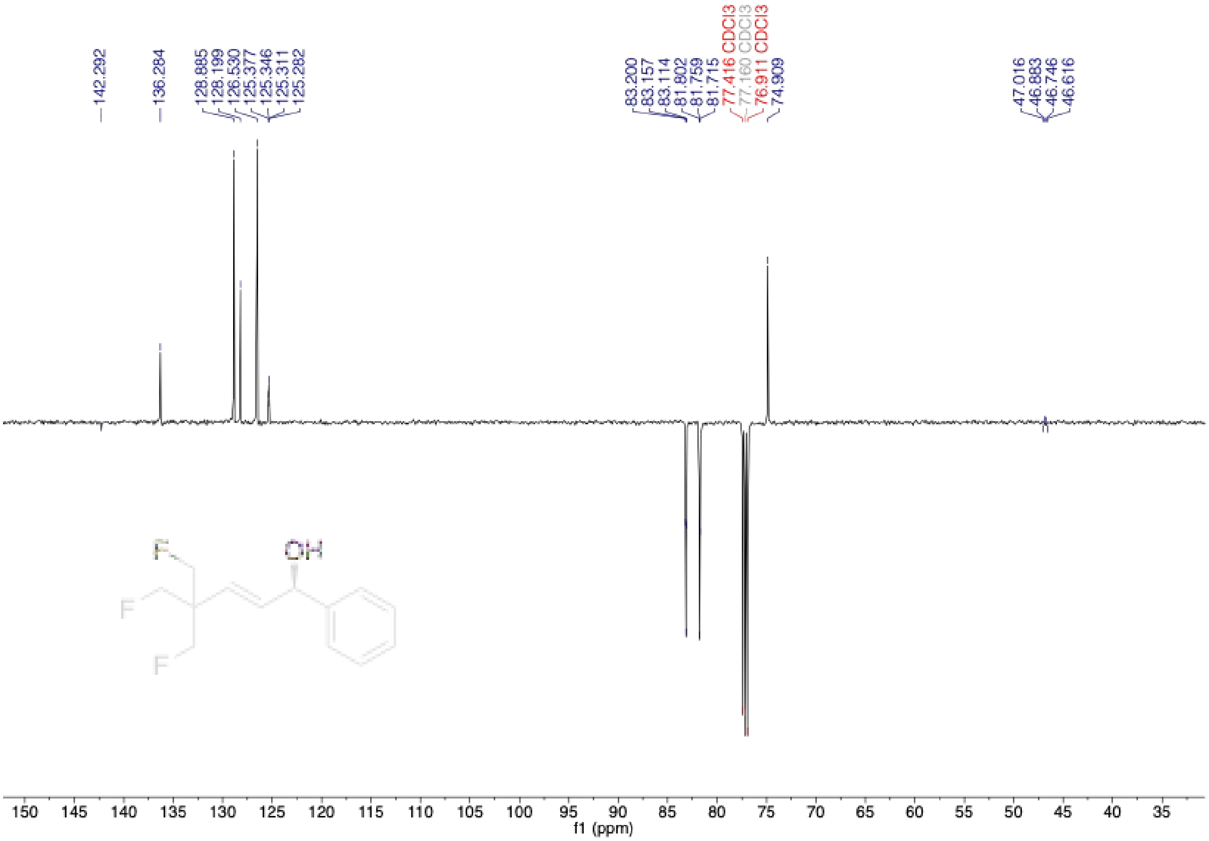

31

^{1}H NMR (500 MHz, $CDCl_3$)

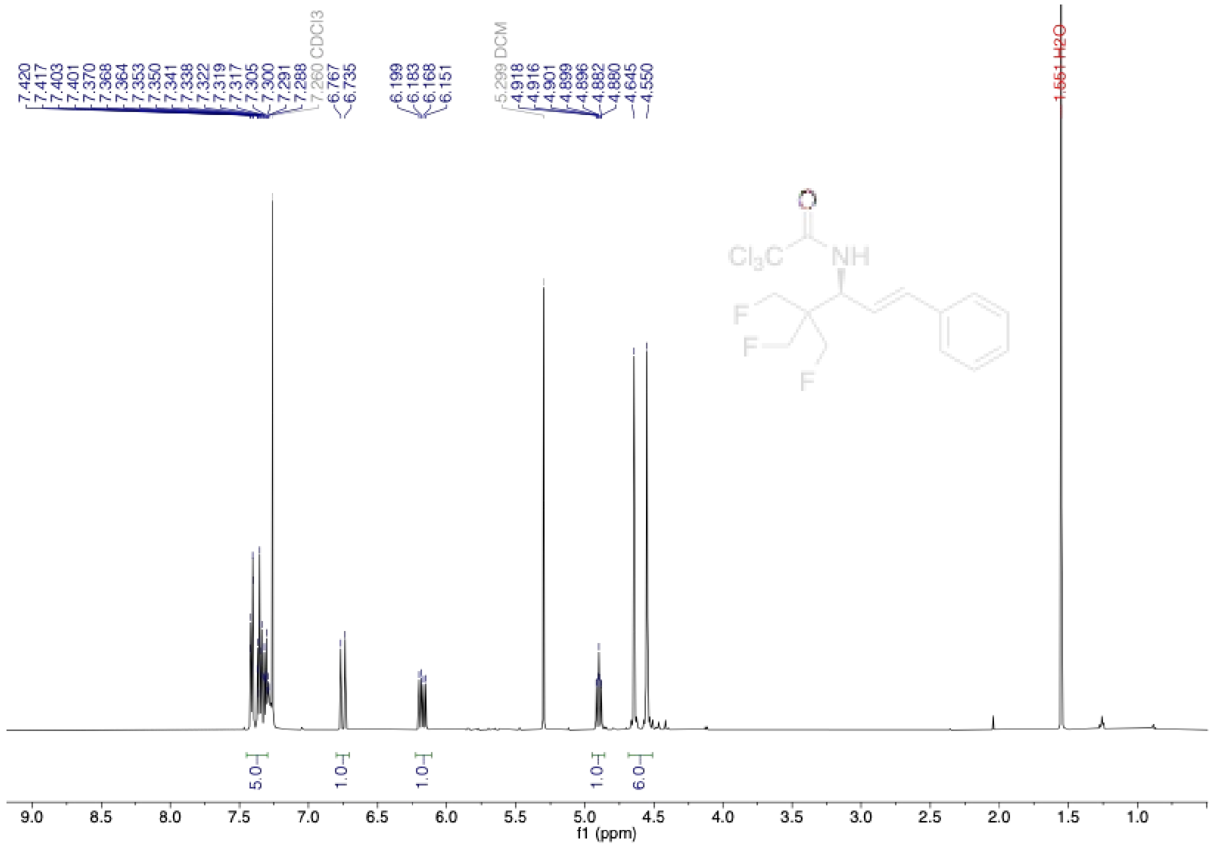

$^{19}F\{^{1}H\}$ NMR (470 MHz, $CDCl_3$)

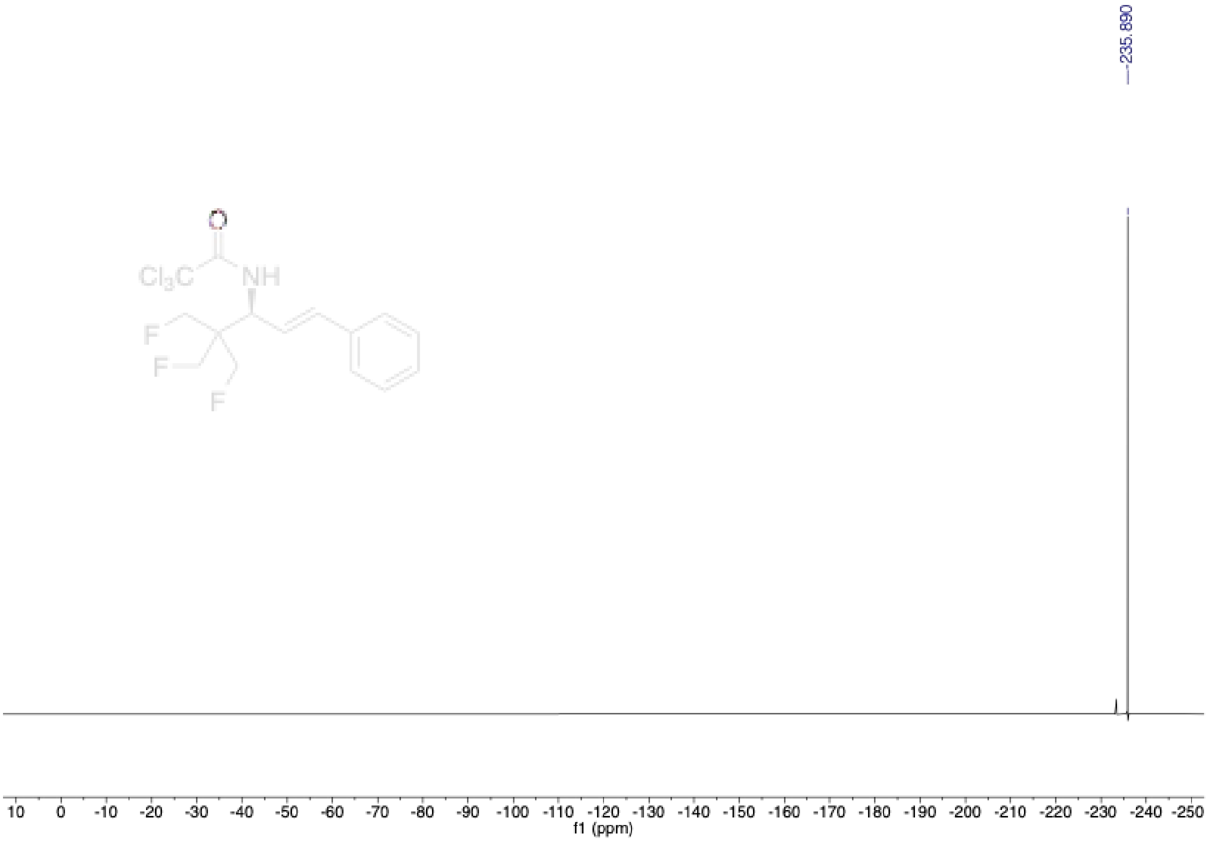

$^{13}C\{^{1}H\}$ NMR (126 MHz, $CDCl_3$

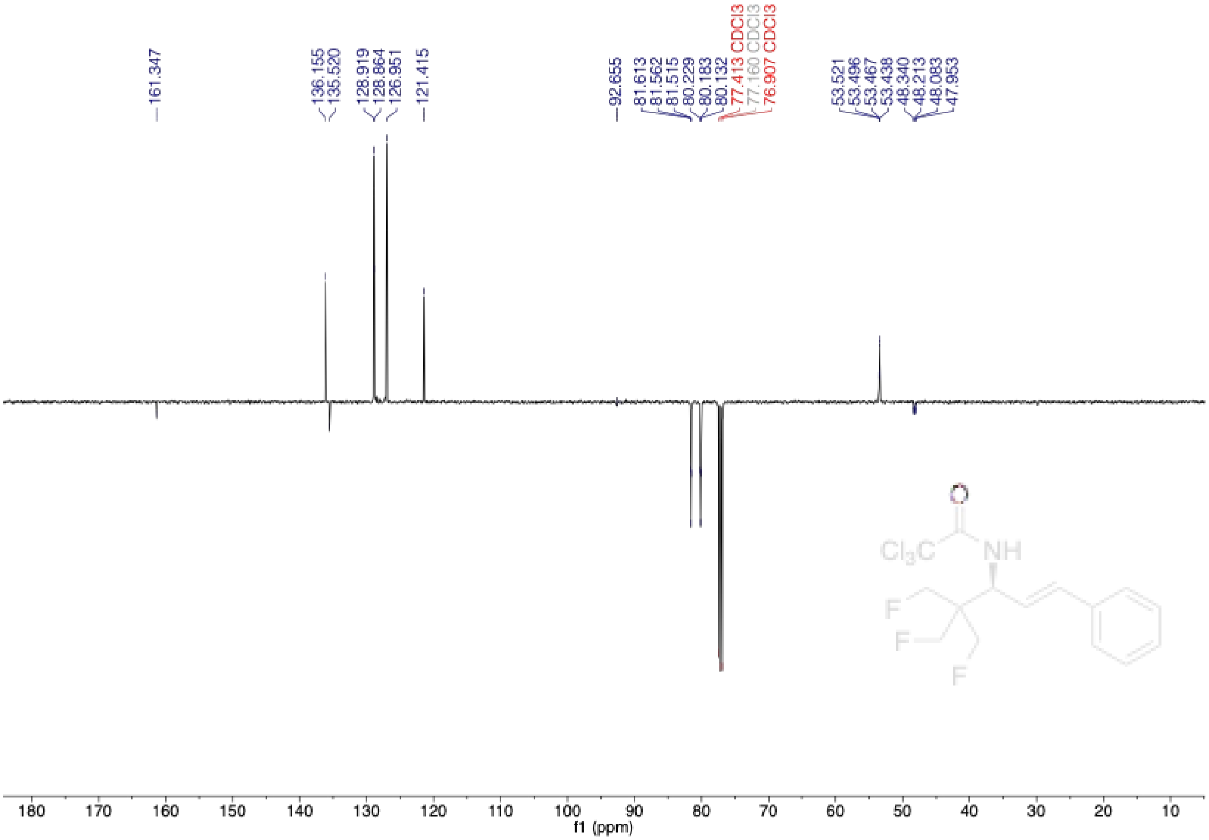

32

^1H NMR (500 MHz, D_2O)

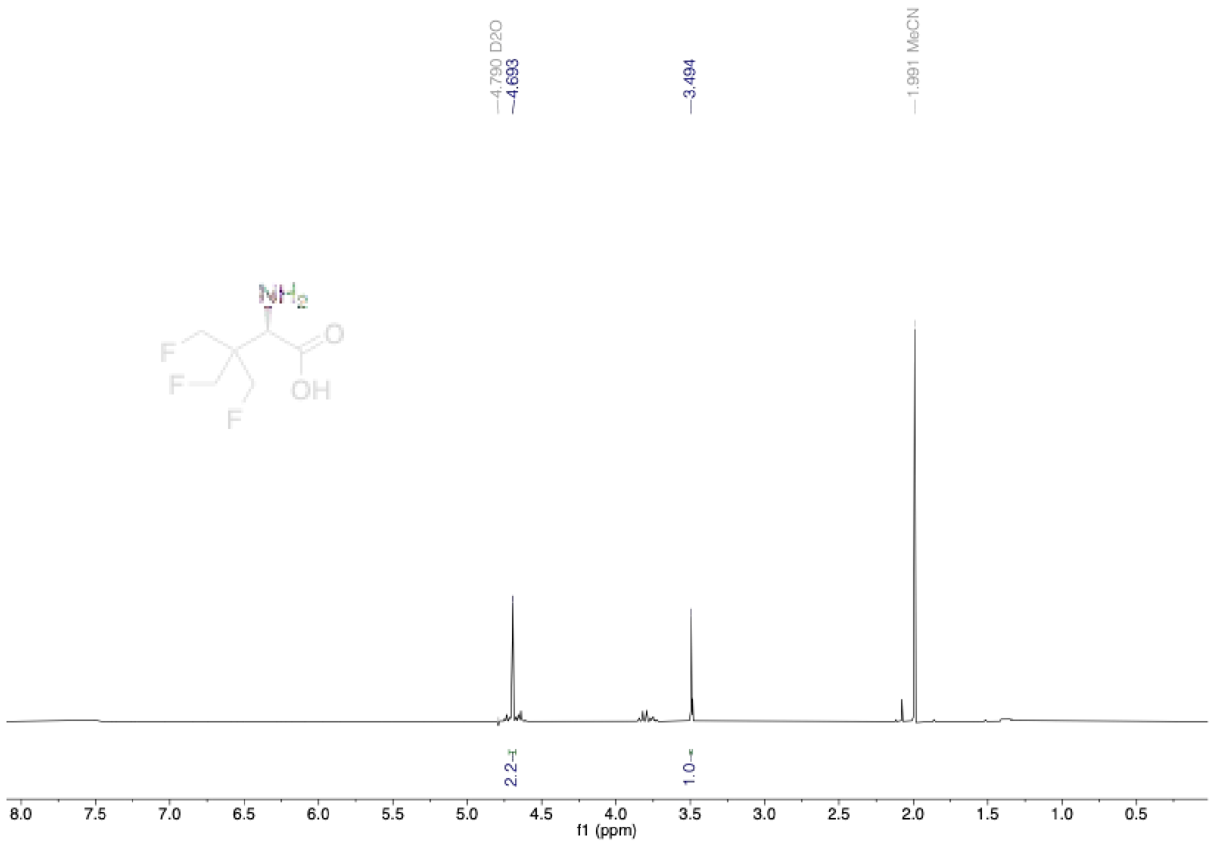

$^{19}F\{^1H\}$ NMR (470 MHz, D_2O)

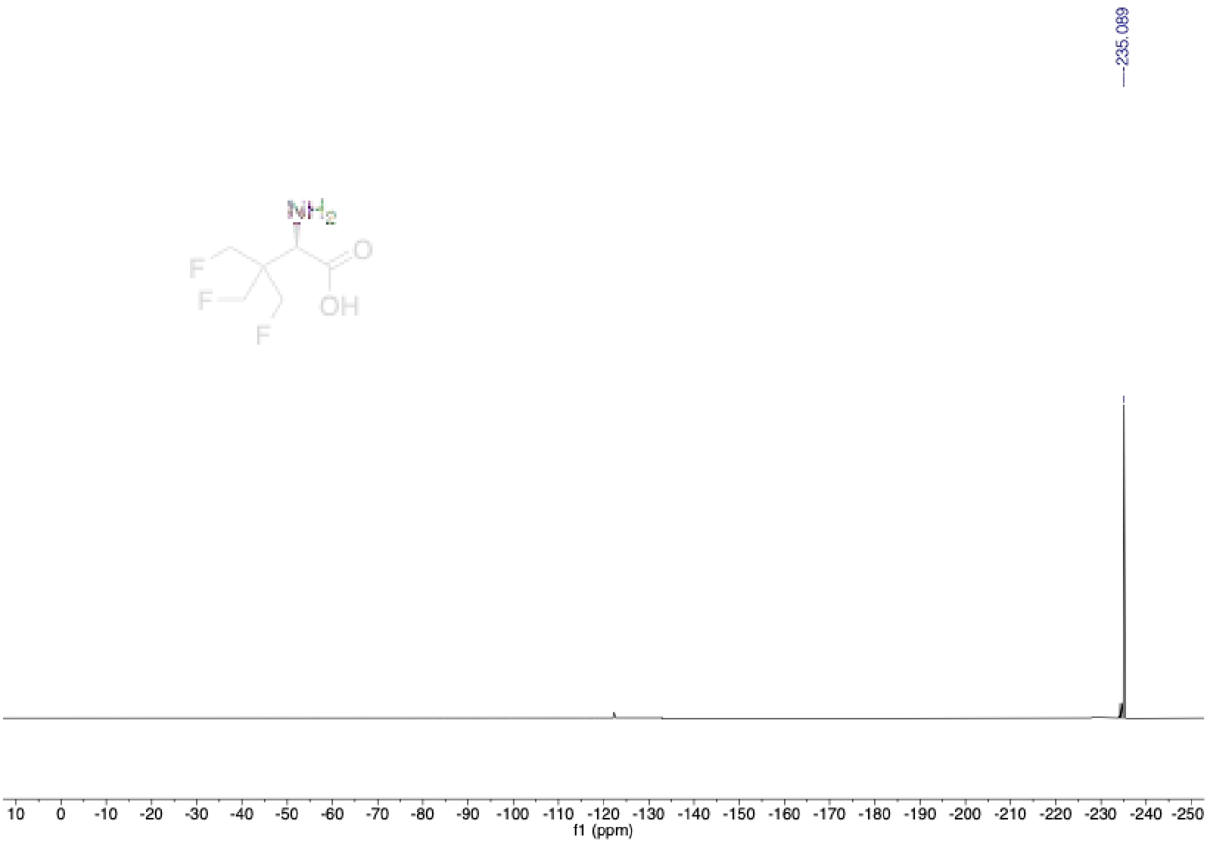

^{1}H-^{19}F HMBC (D_2O)

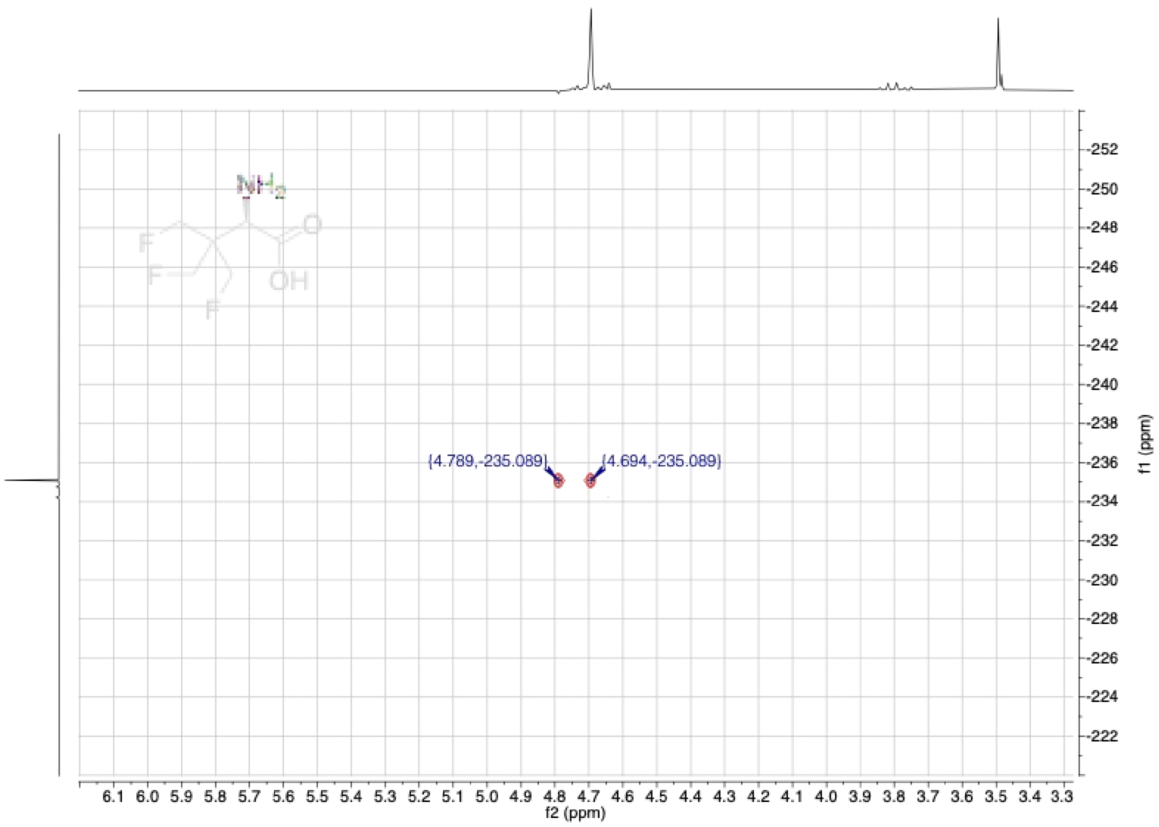

^{1}H-^{13}C HMBC (D_2O)

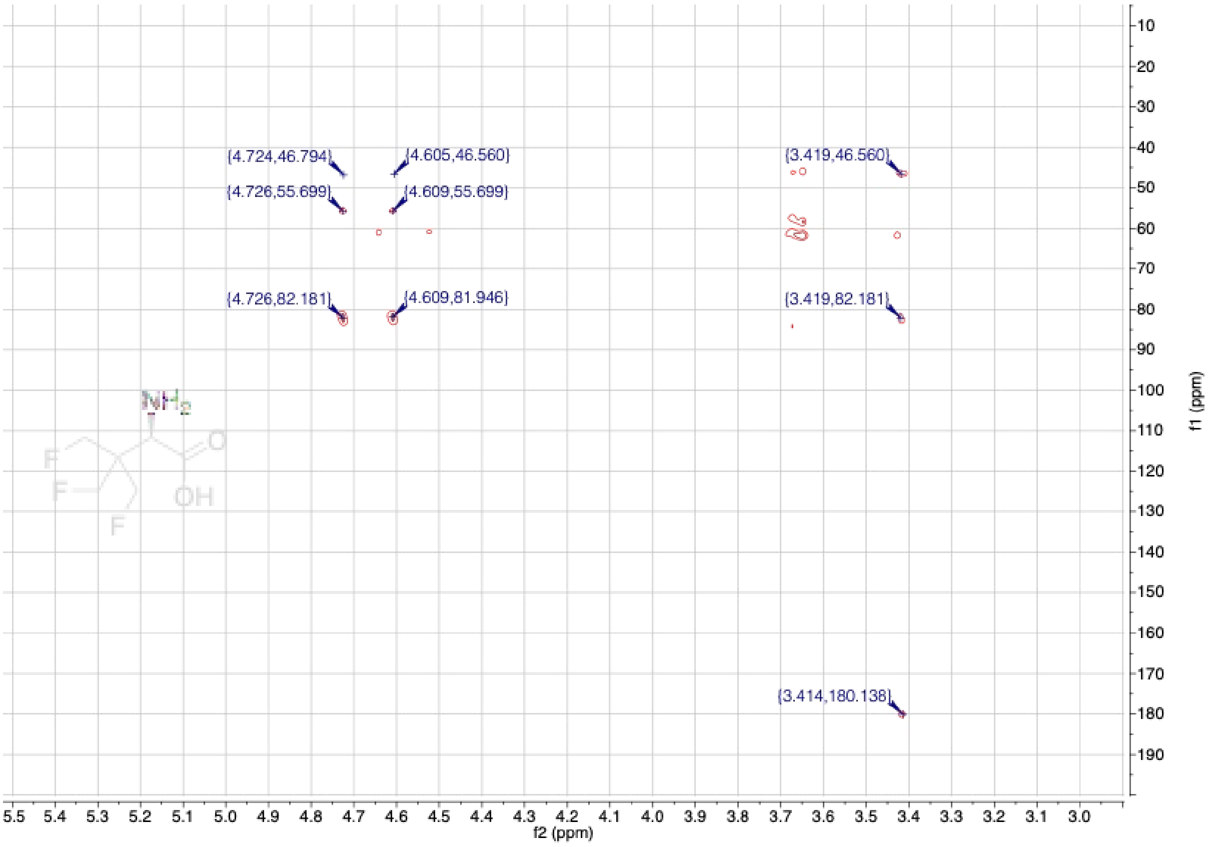

^{1}H-^{13}C HSQC (D_2O)

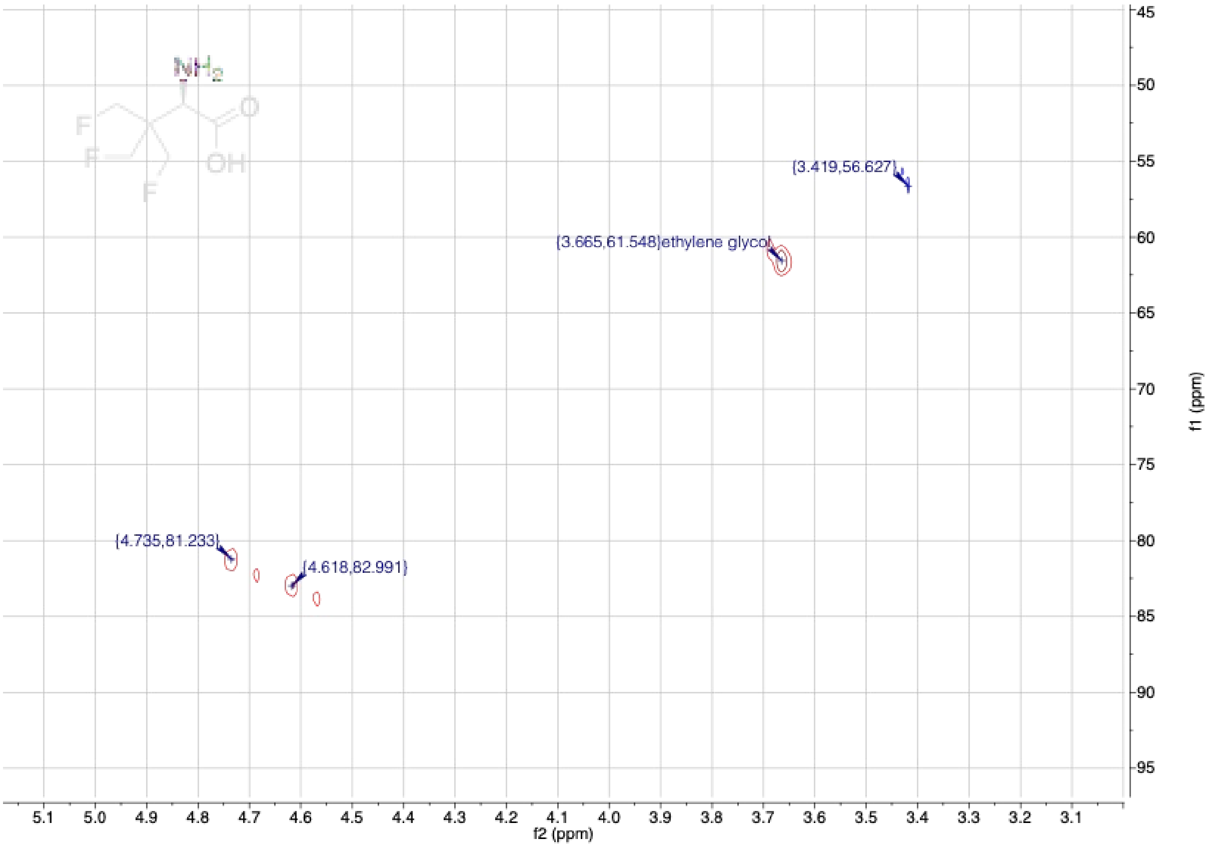

PE-6

^{1}H NMR (500 MHz, $CDCl_3$)

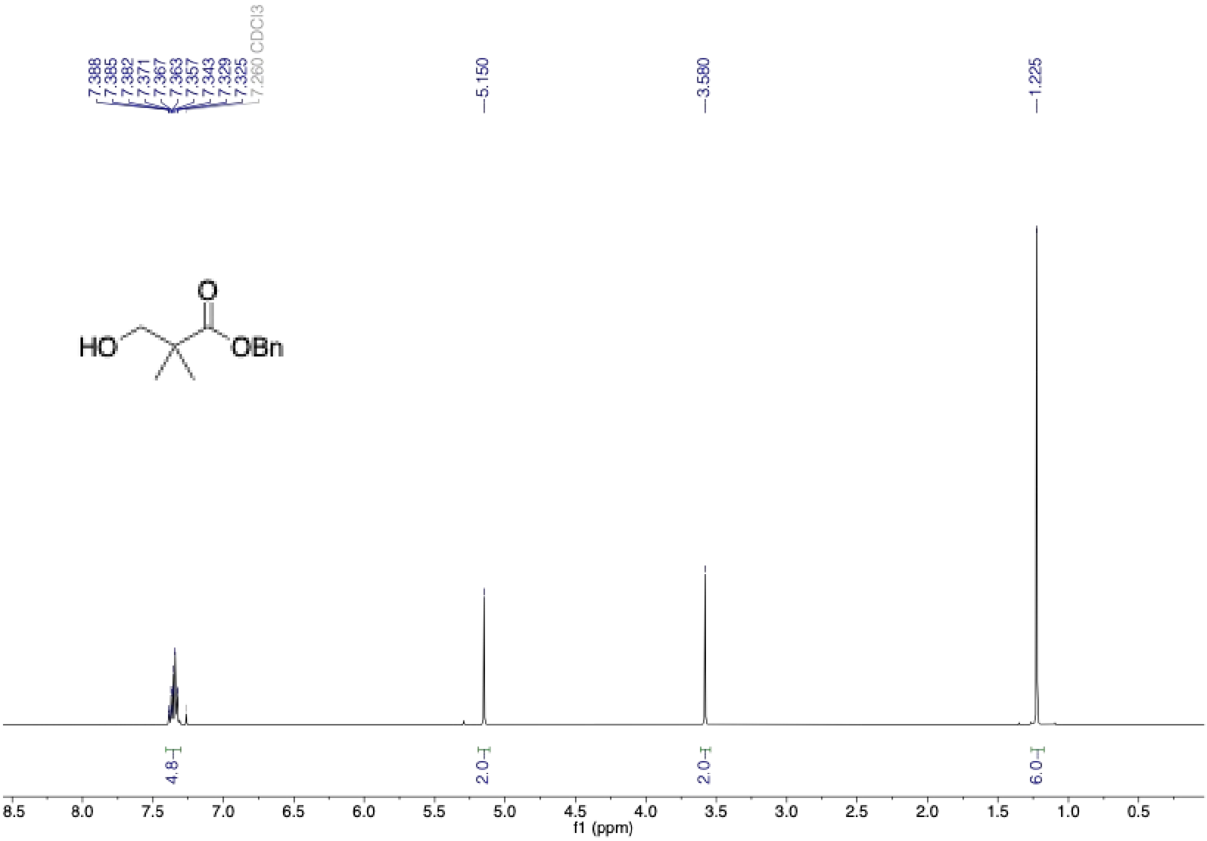

S3

^{1}H NMR (500 MHz, $CDCl_3$)

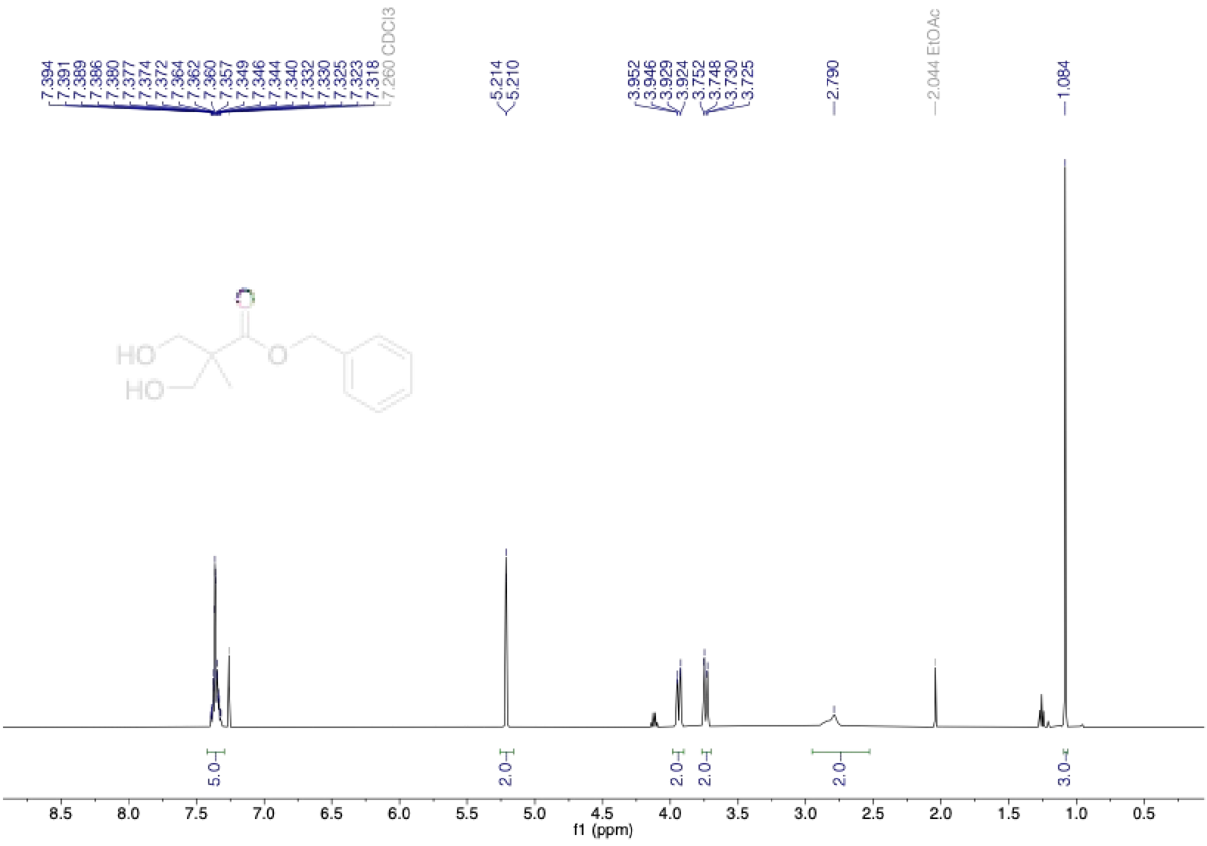
7.394
7.391
7.389
7.386
7.380
7.377
7.374
7.372
7.364
7.362
7.360
7.357
7.349
7.346
7.344
7.340
7.332
7.330
7.325
7.323
7.318
7.260 CDCl3
5.214
5.210
3.952
3.946
3.929
3.924
3.752
3.748
3.730
3.725
2.790
2.044 EtOAc
1.084
HO
HO
O
O
5.0
2.0
2.0
2.0
2.0
3.0
8.5 8.0 7.5 7.0 6.5 6.0 5.5 5.0 4.5 4.0 3.5 3.0 2.5 2.0 1.5 1.0 0.5
f1 (ppm)

PE-1

^{1}H NMR (400 MHz, $CDCl_3$)

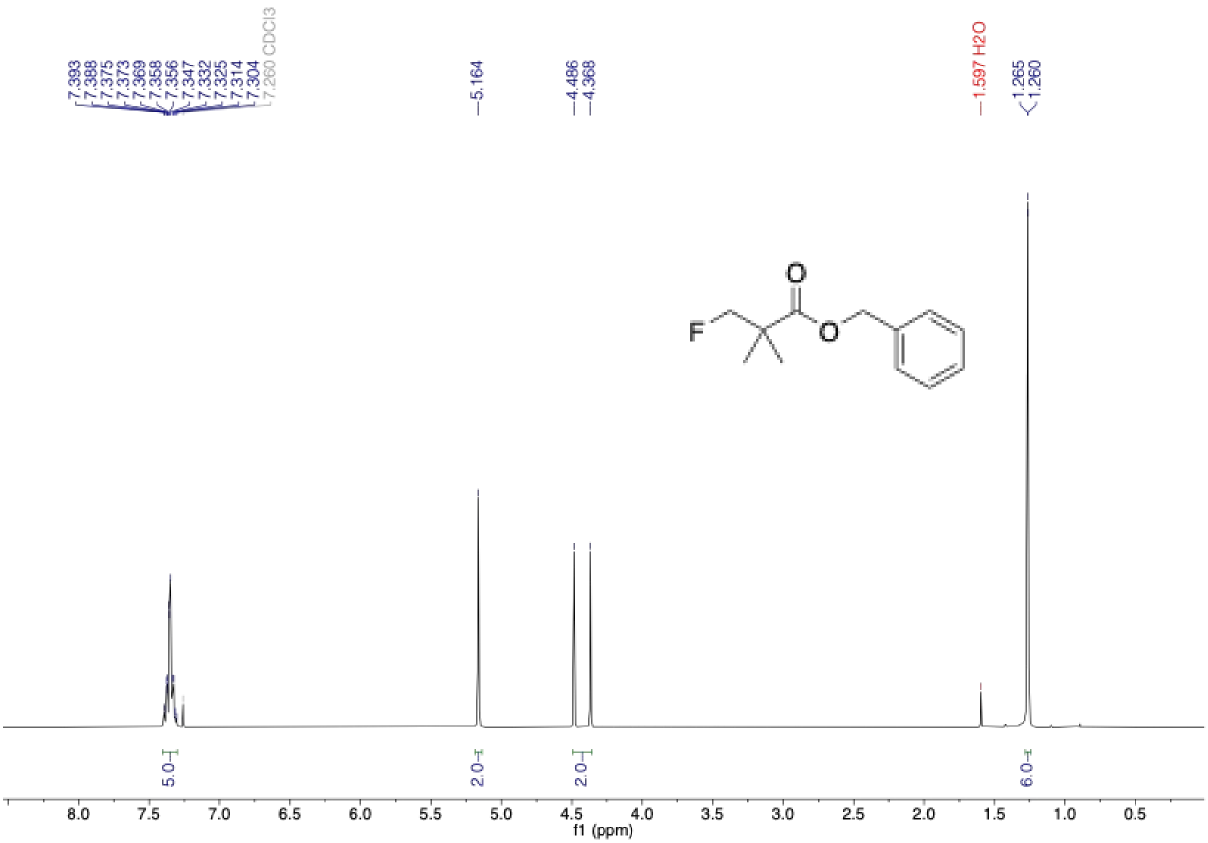

$^{19}F\{^{1}H\}$ NMR (377 MHz, $CDCl_3$)

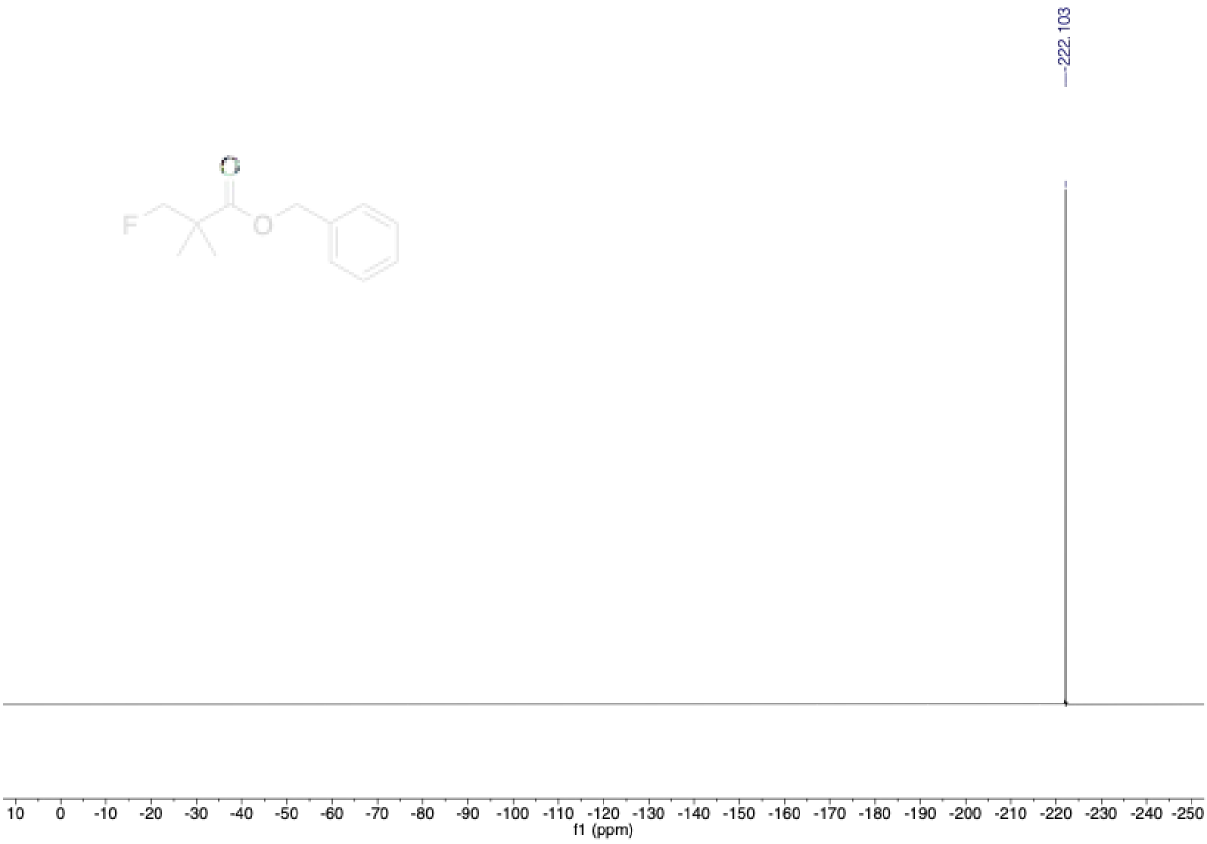

PE-2

^{1}H NMR (400 MHz, $CDCl_3$)

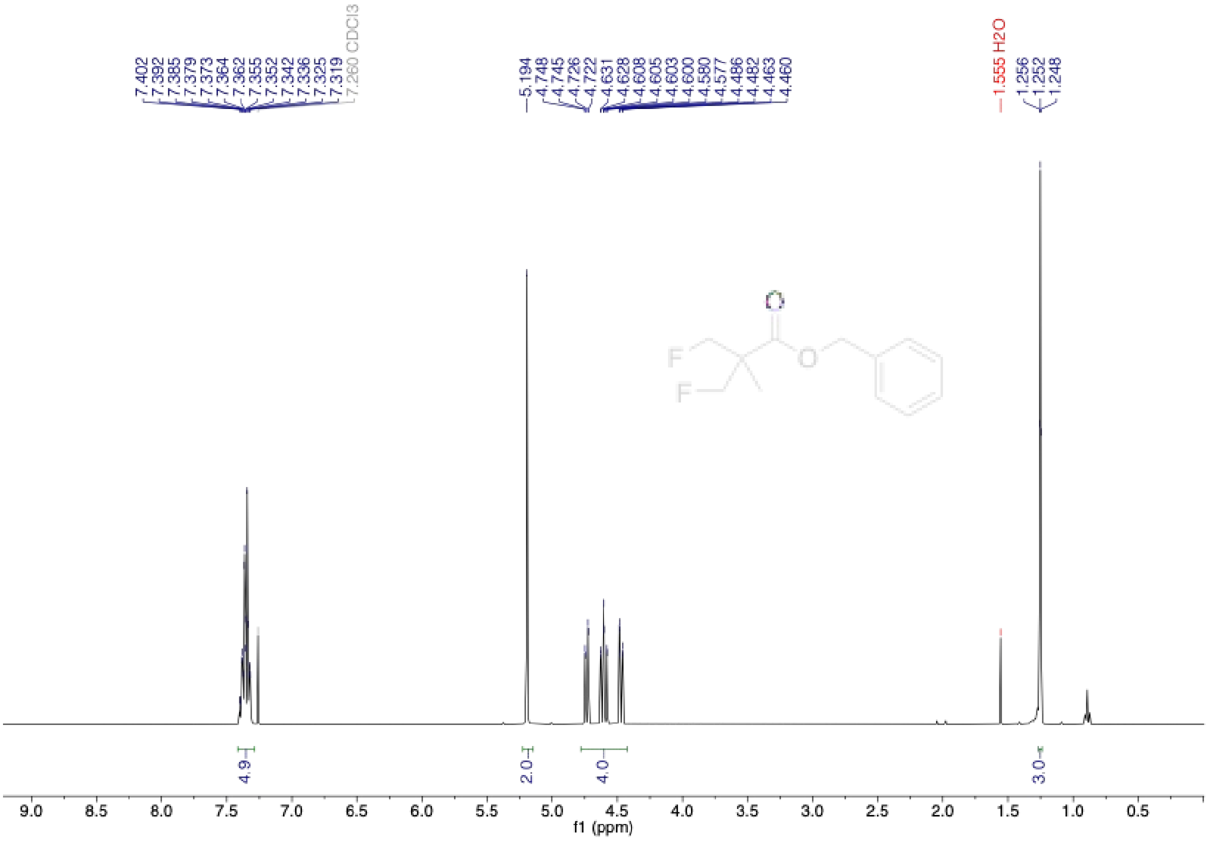

$^{19}F\{^1H\}$ NMR (377 MHz, $CDCl_3$)

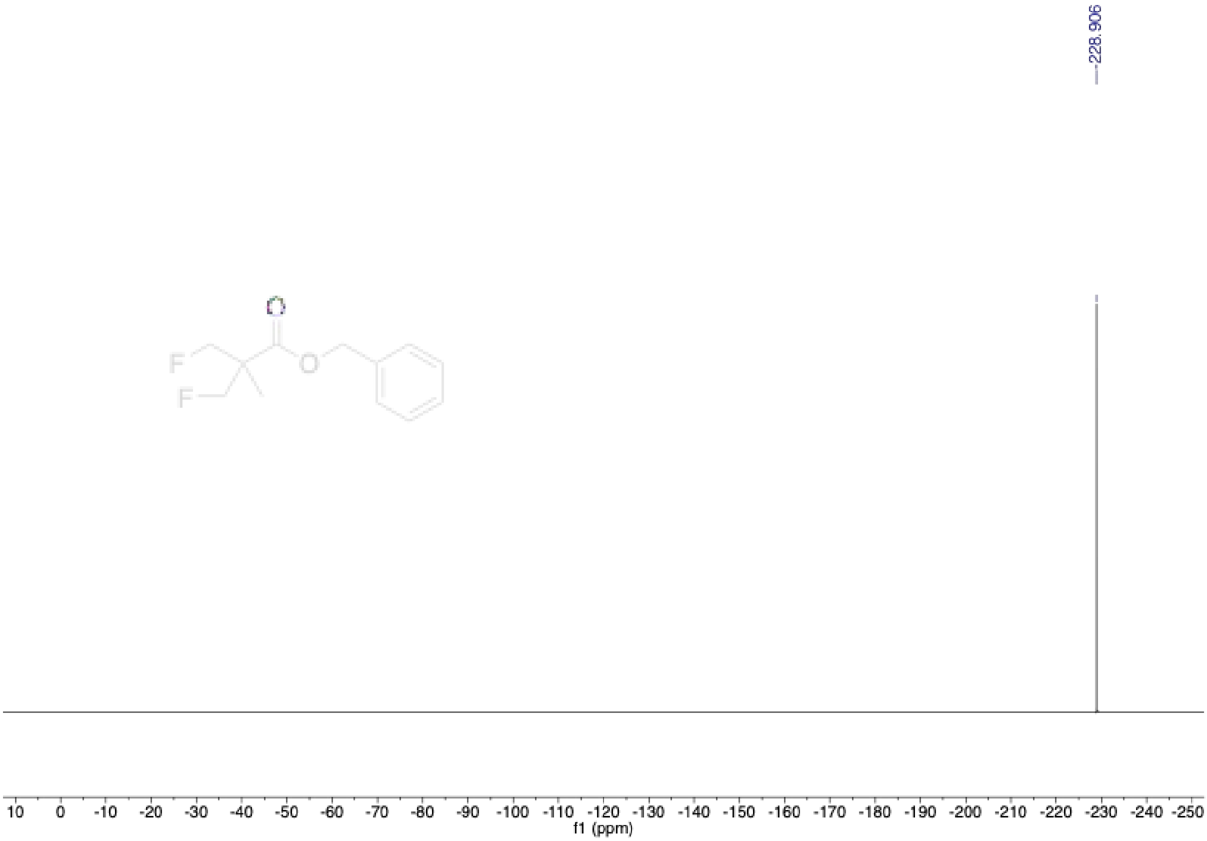

$^{13}C\{^1H\}$ NMR (126 MHz, $CDCl_3$)

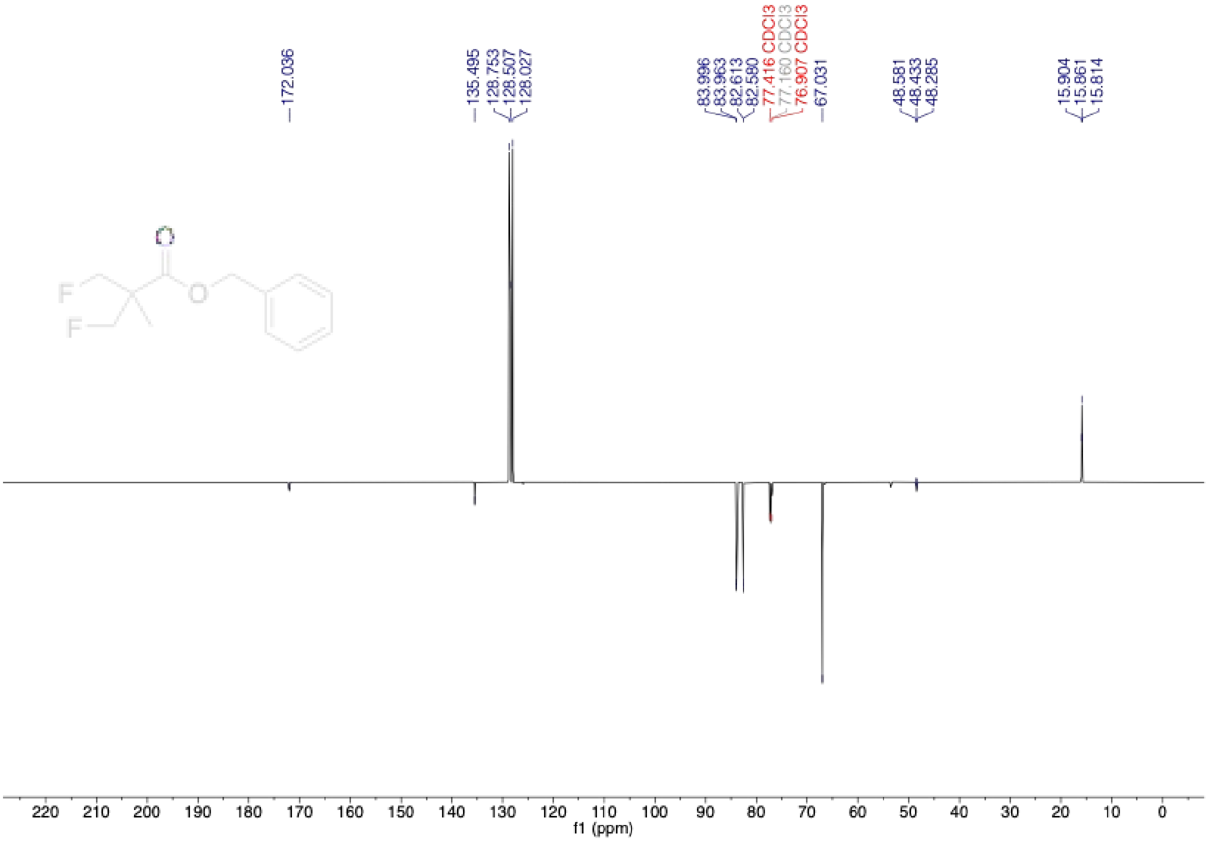

PA-1

^{1}H NMR (400 MHz, $CDCl_3$)

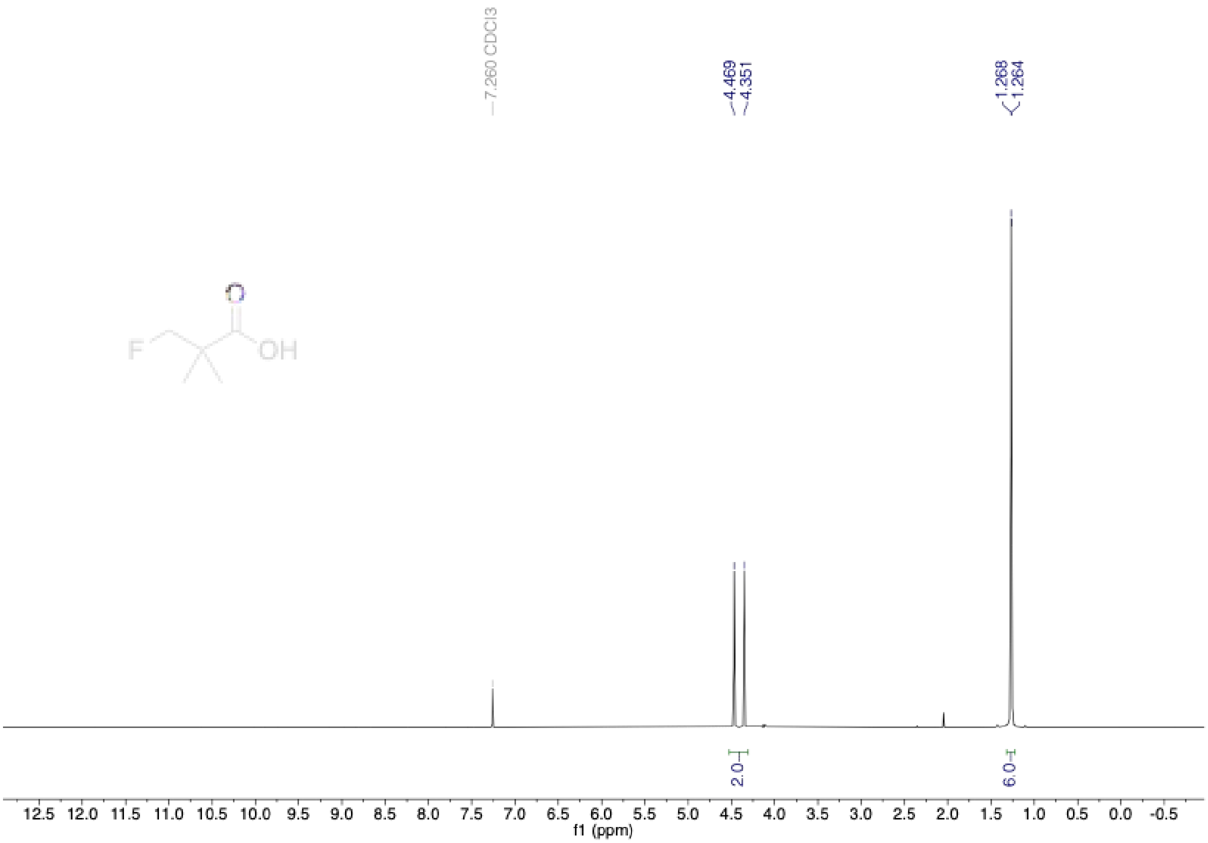

^{19}F{^{1}H} NMR (377 MHz, $CDCl_3$)

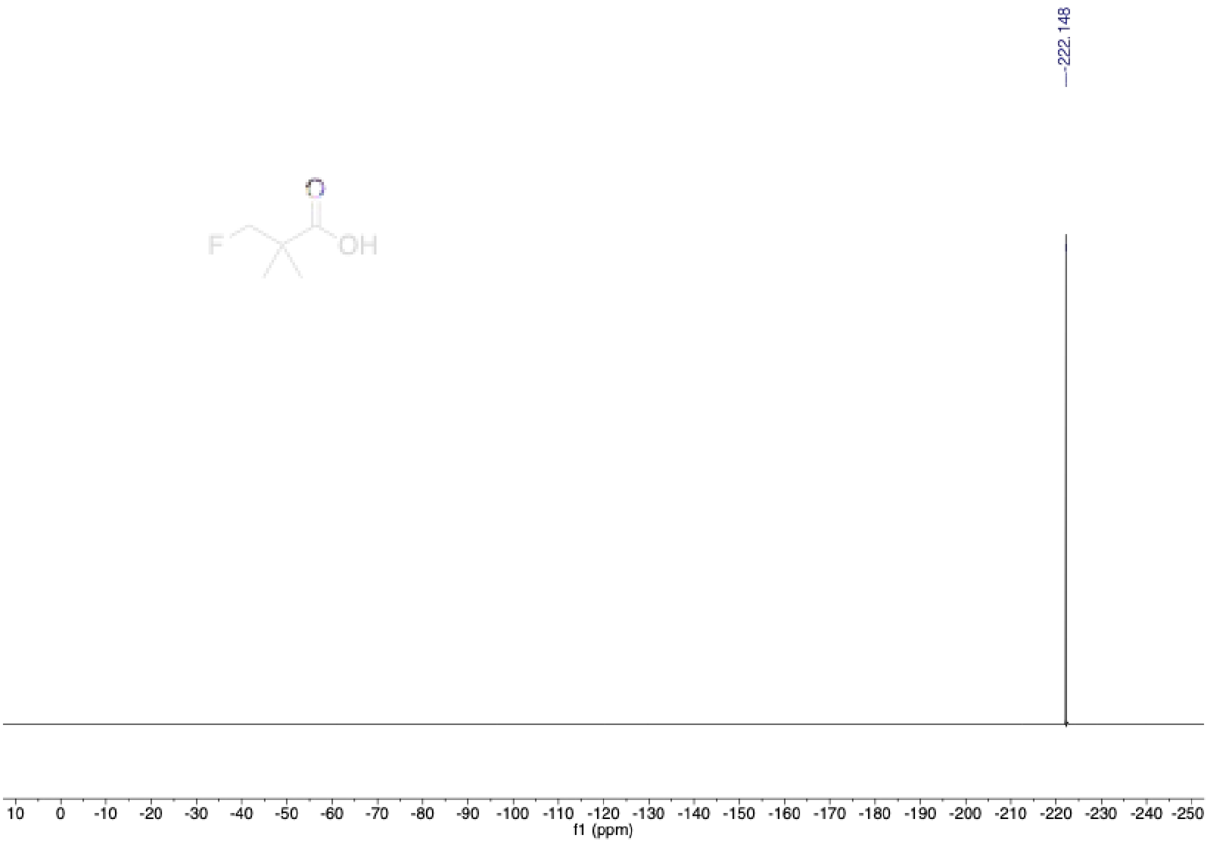

PA-2

^{1}H NMR (400 MHz, $CDCl_3$)

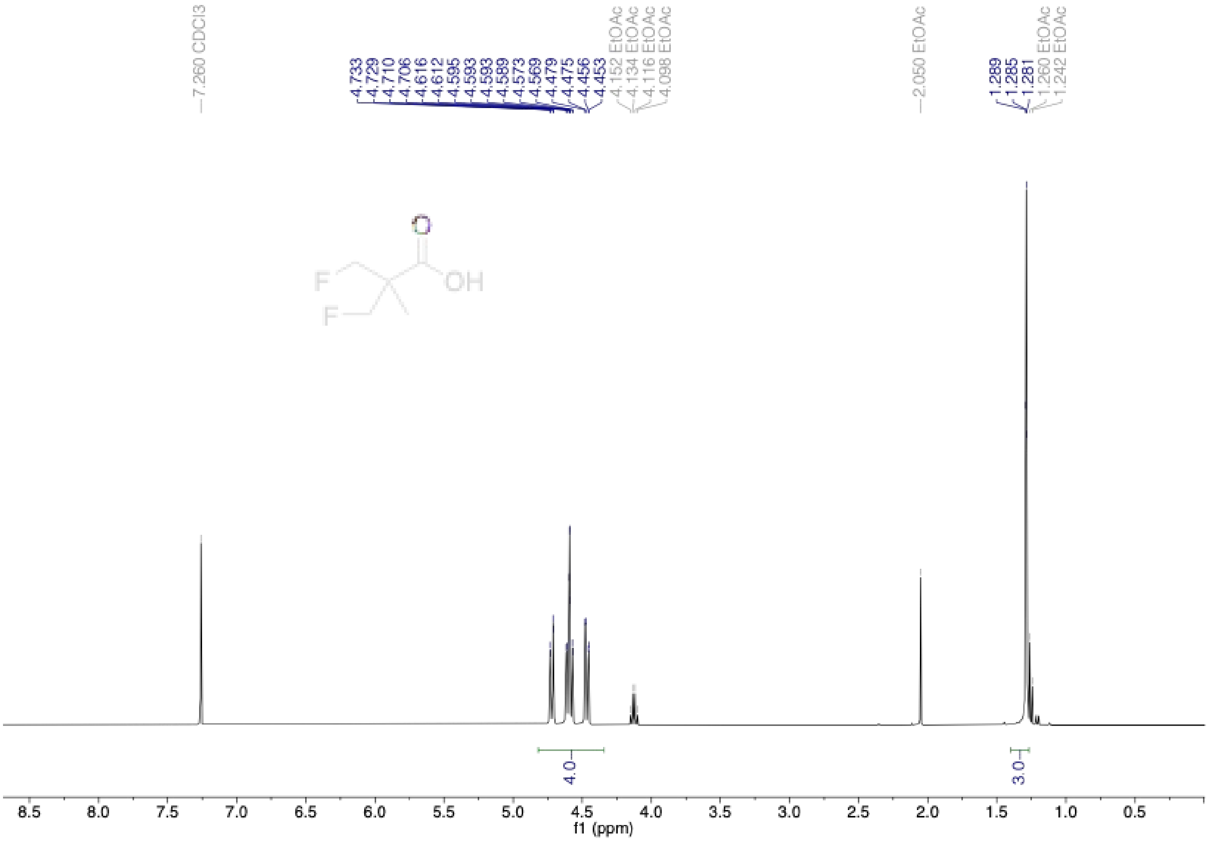

$^{19}F\{^{1}H\}$ NMR (377 MHz, $CDCl_3$)

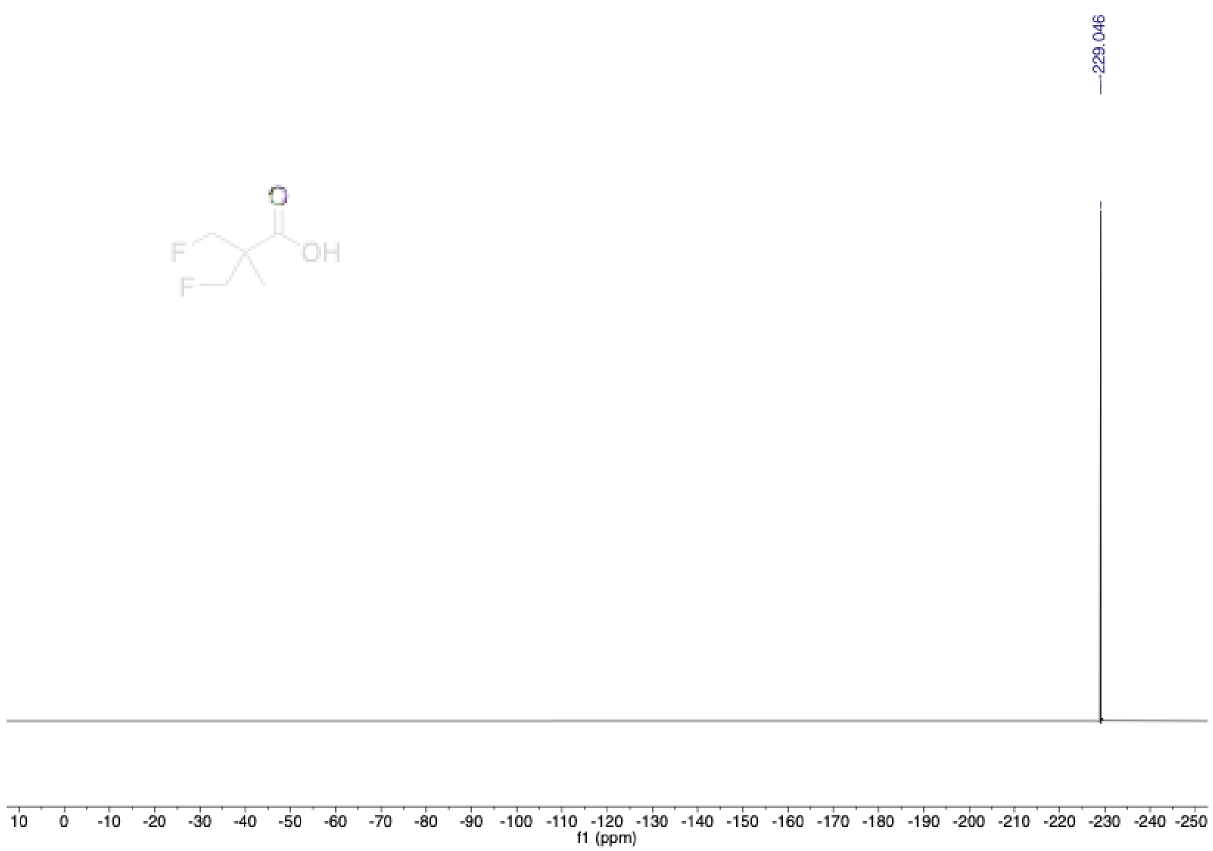

$^{13}C\{^{1}H\}$ NMR (126 MHz, $CDCl_3$)

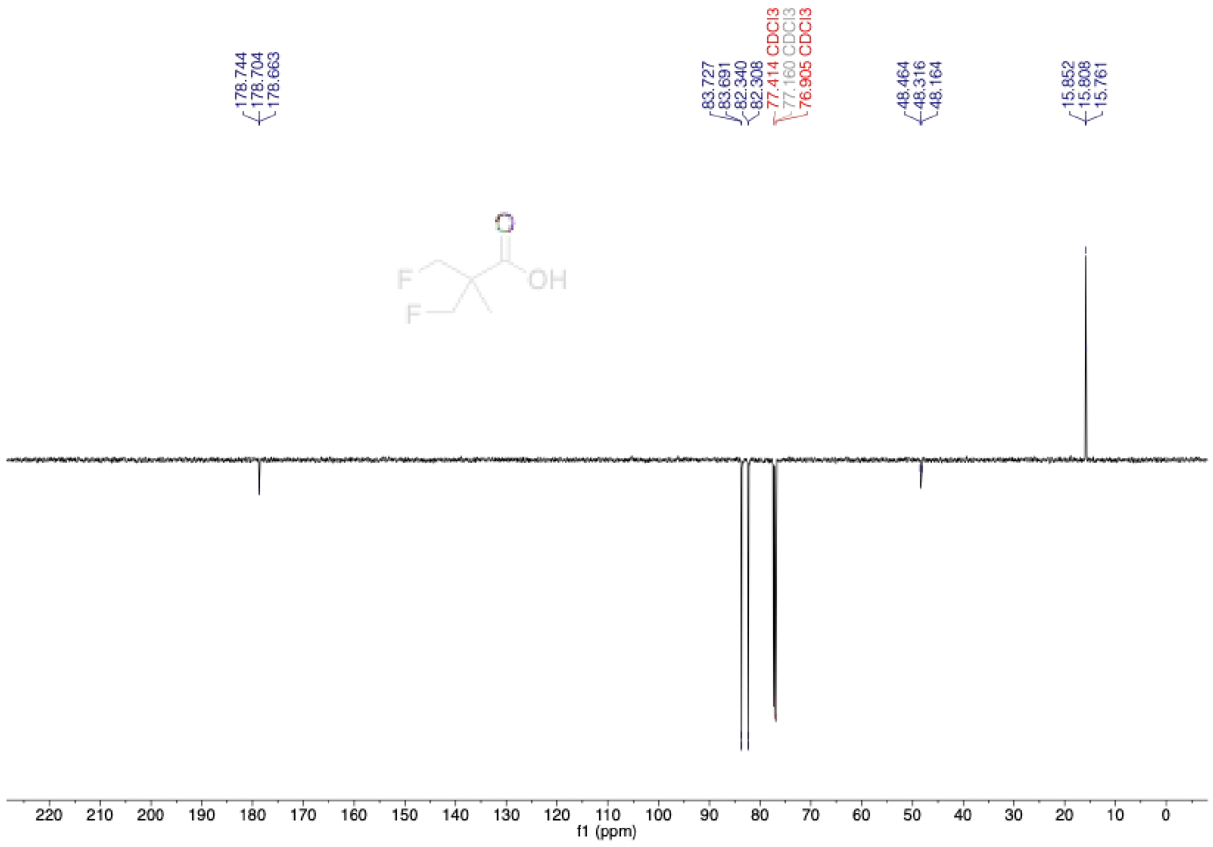

S7

^{1}H NMR (500 MHz, $CDCl_3$)

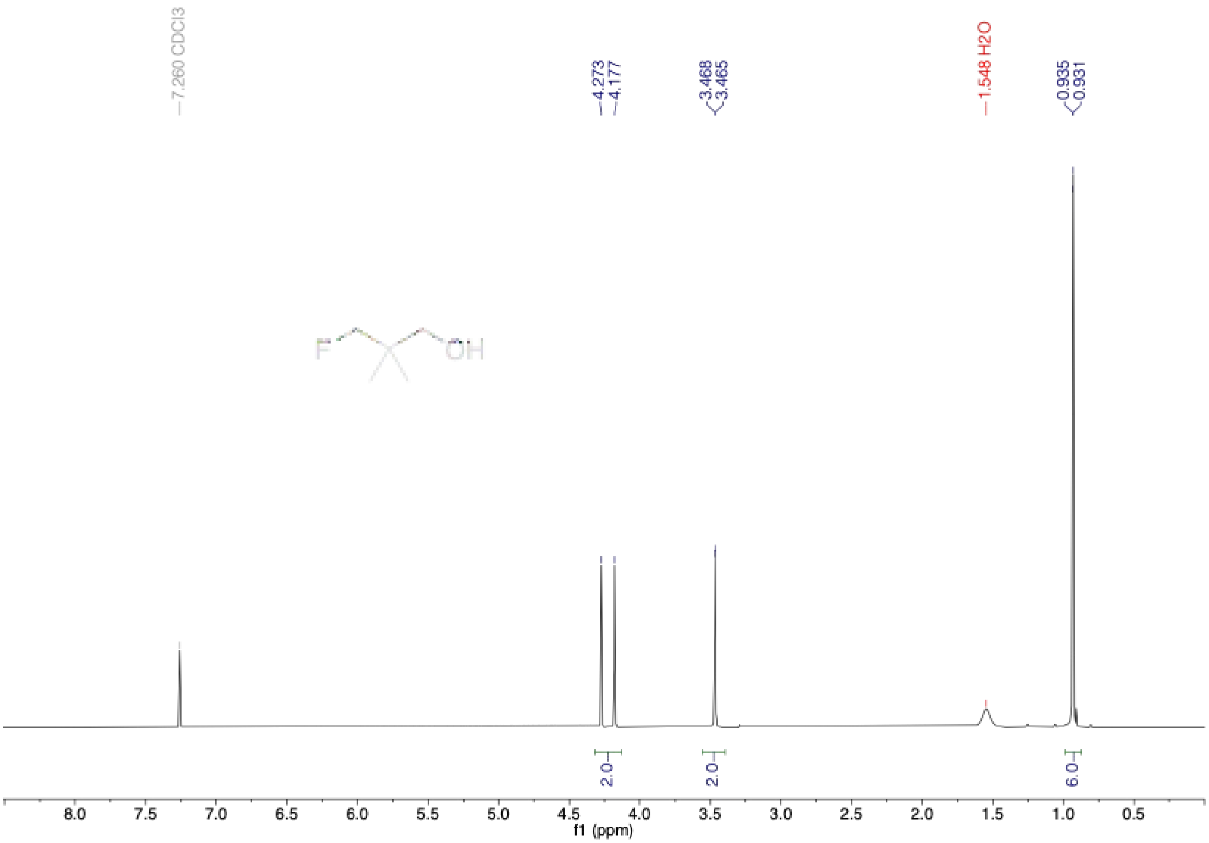

$^{19}F\{^{1}H\}$ NMR (470 MHz, $CDCl_3$)

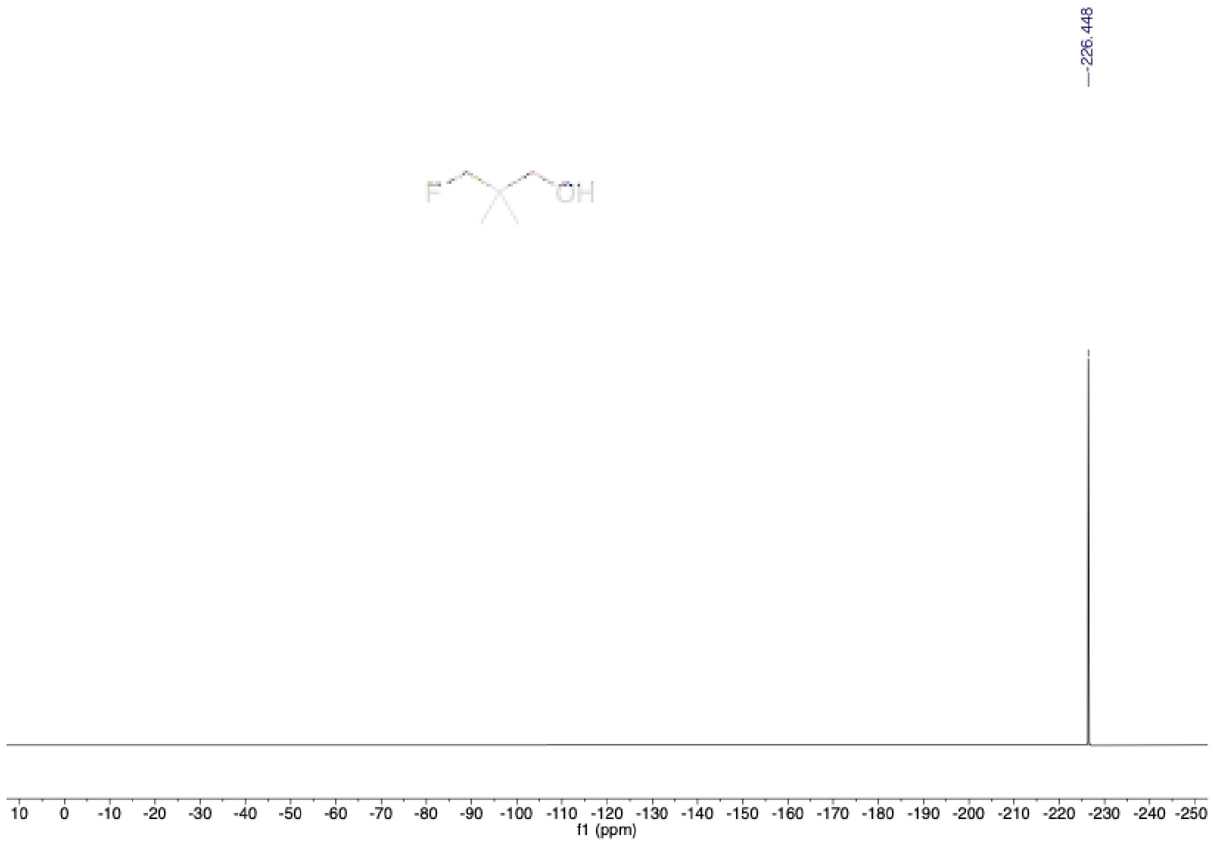

S8

^{1}H NMR (500 MHz, $CDCl_3$)

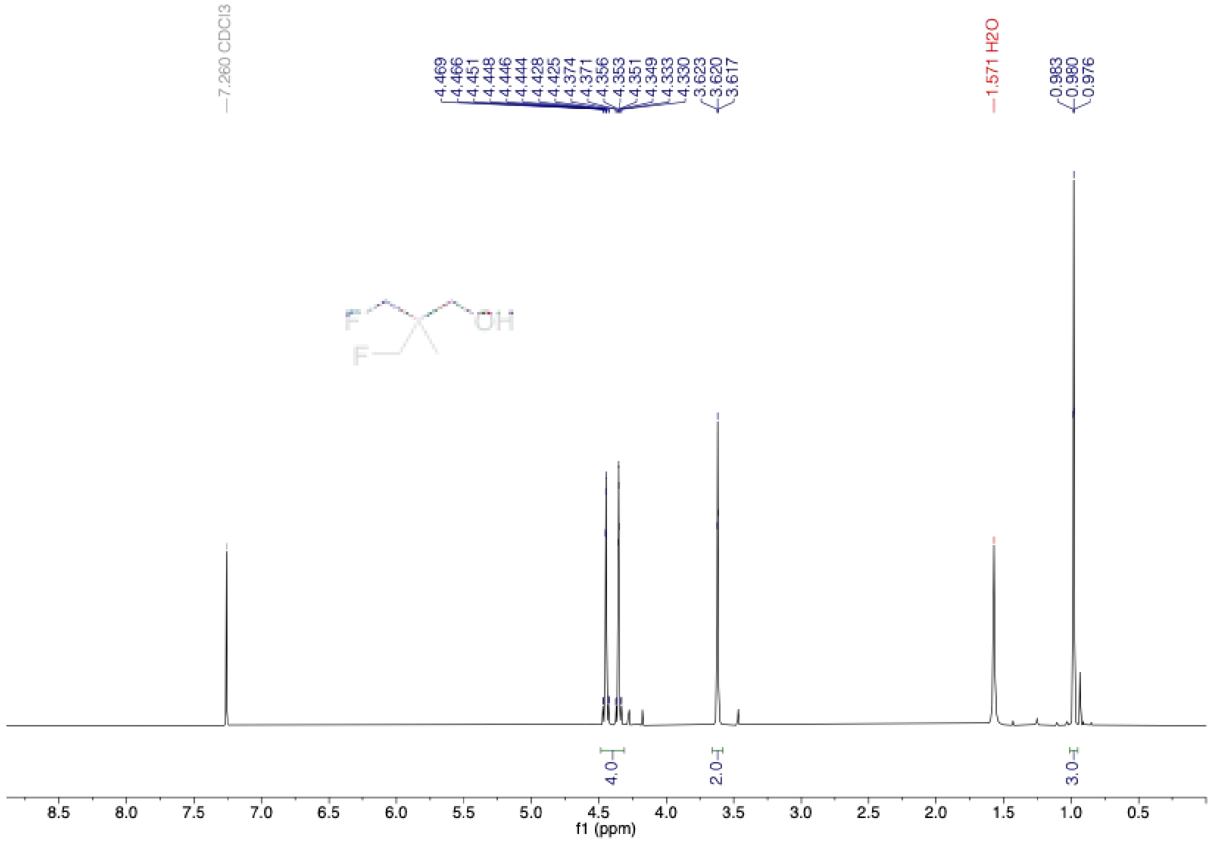

$^{19}F\{^1H\}$ NMR (470 MHz, $CDCl_3$)

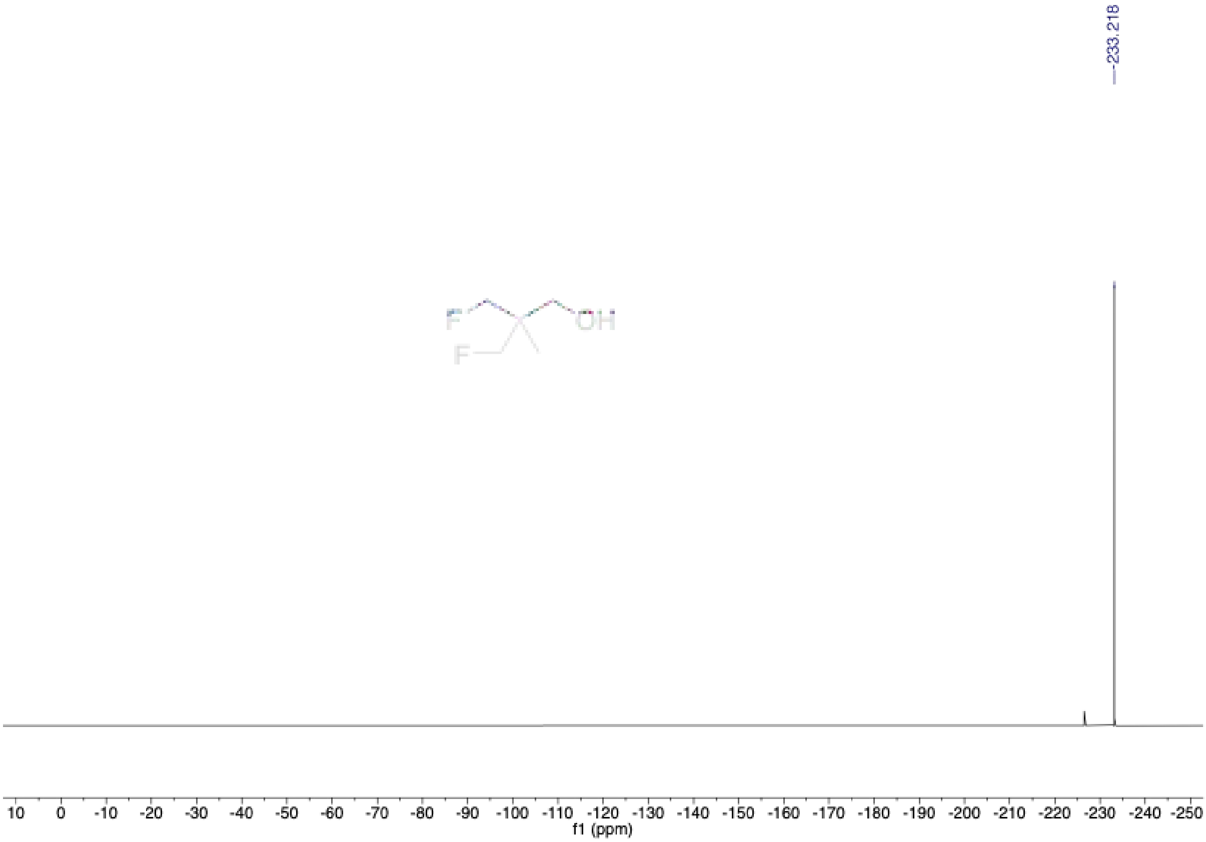

$^{13}C\{^1H\}$ NMR (126 MHz, $CDCl_3$)

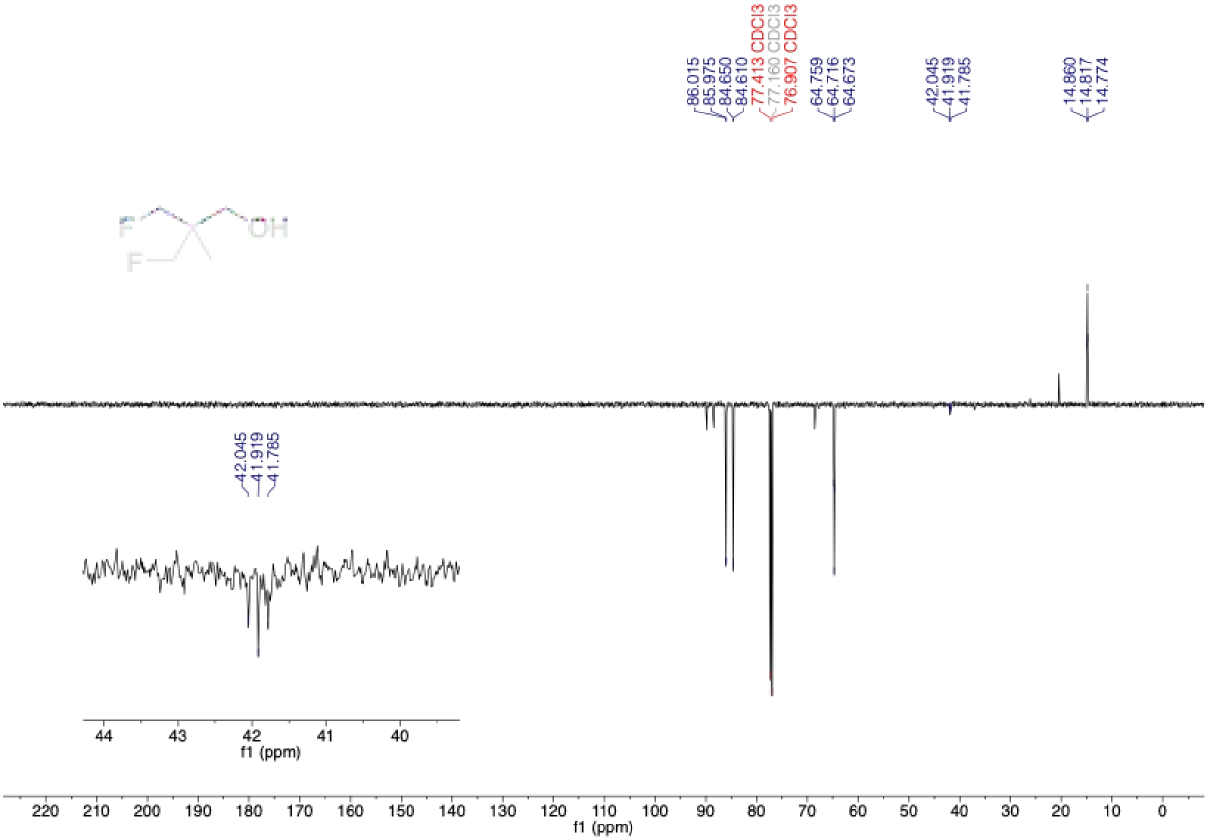

33

^{1}H NMR (400 MHz, $CDCl_3$)

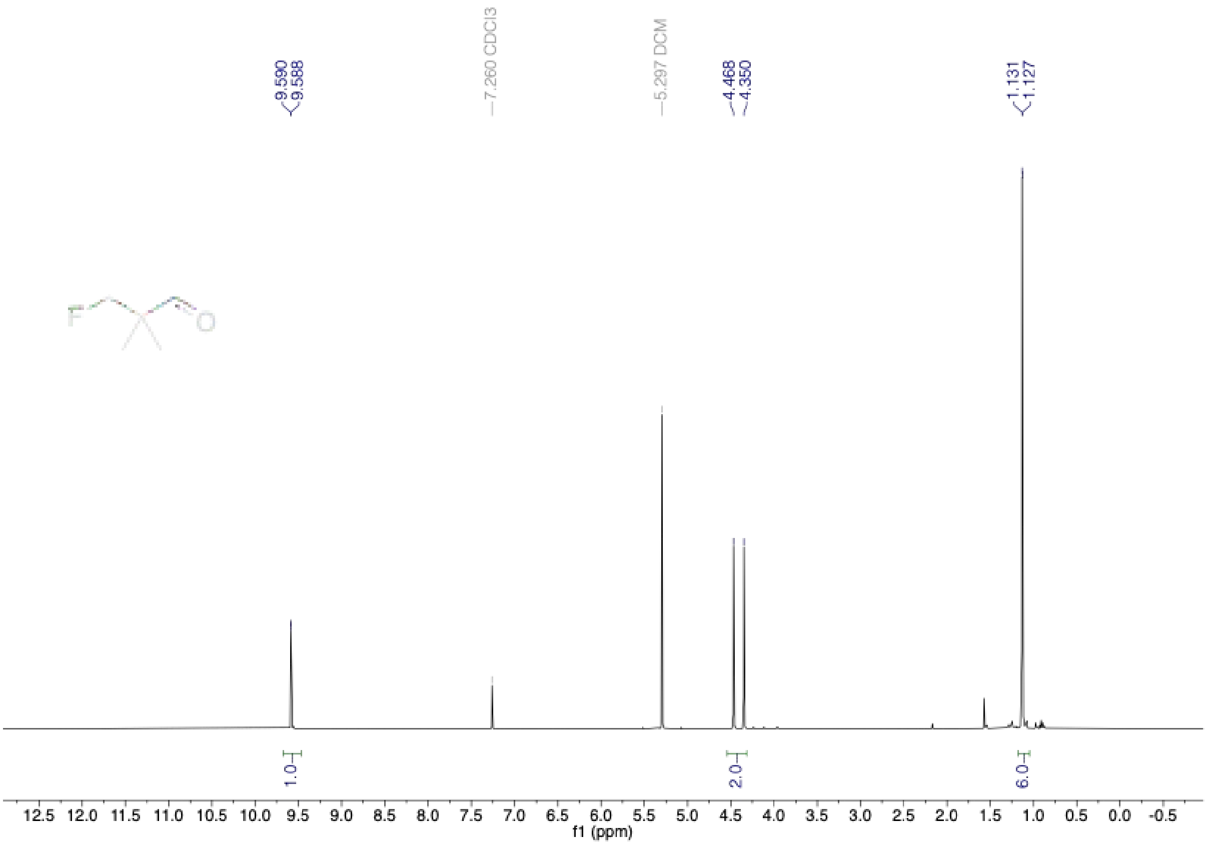

$^{19}F\{^{1}H\}$ NMR (377 MHz, $CDCl_3$)

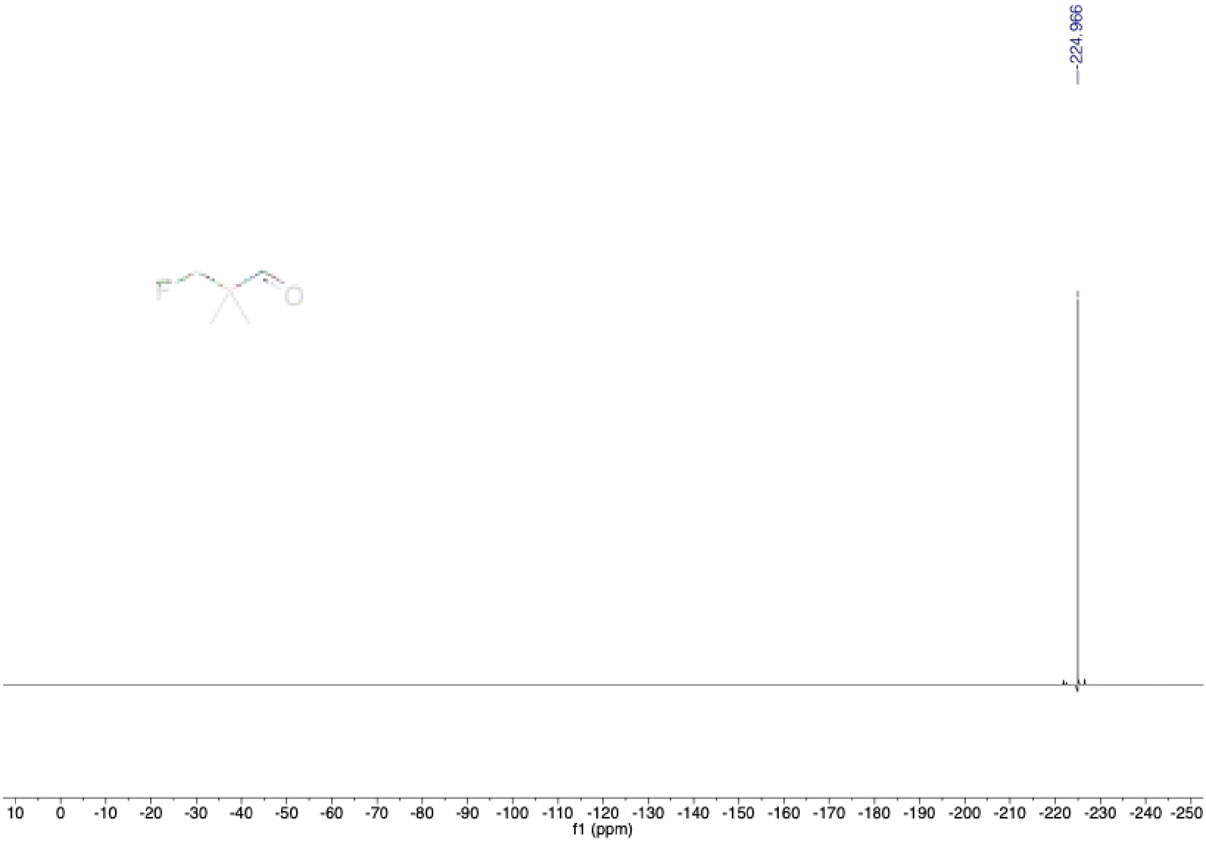

$^{13}C\{^{1}H\}$ NMR (126 MHz, $CDCl_3$)

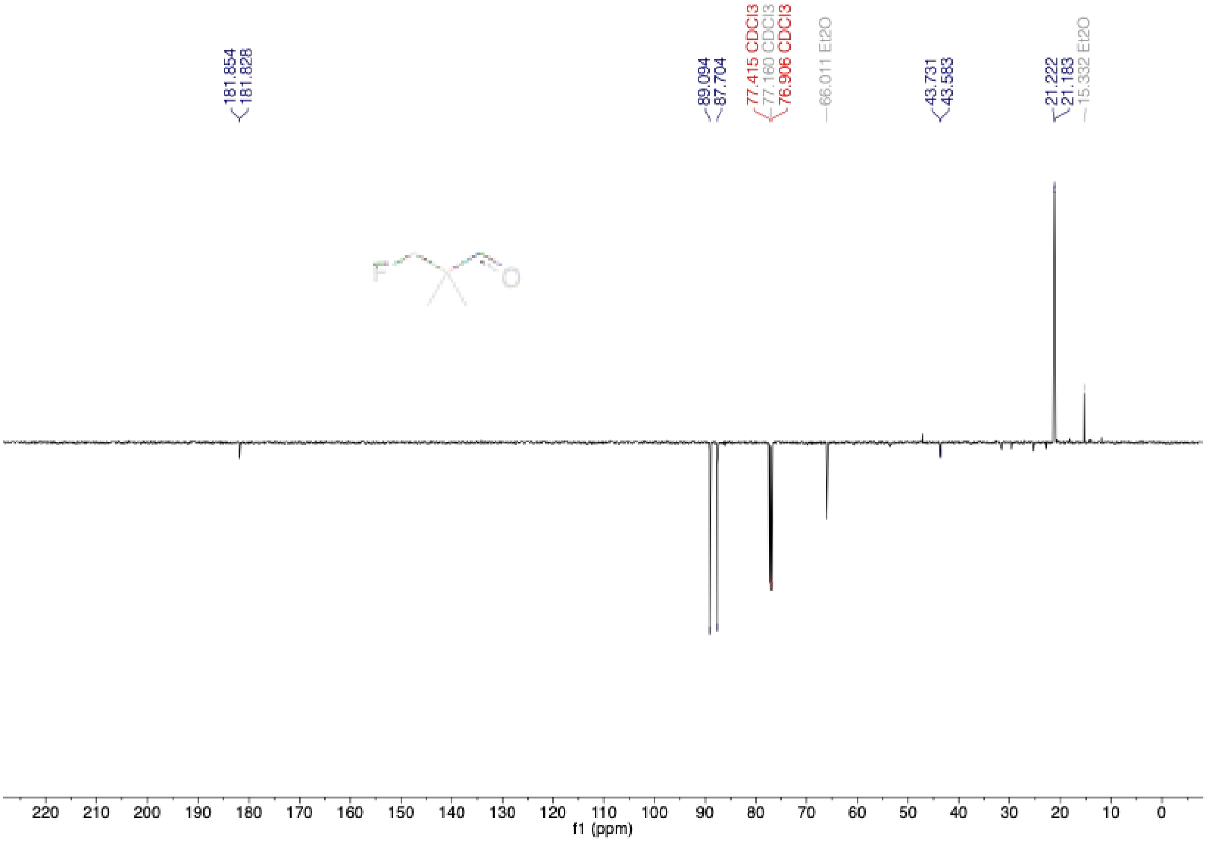

34

^{1}H NMR (500 MHz, $CDCl_3$)

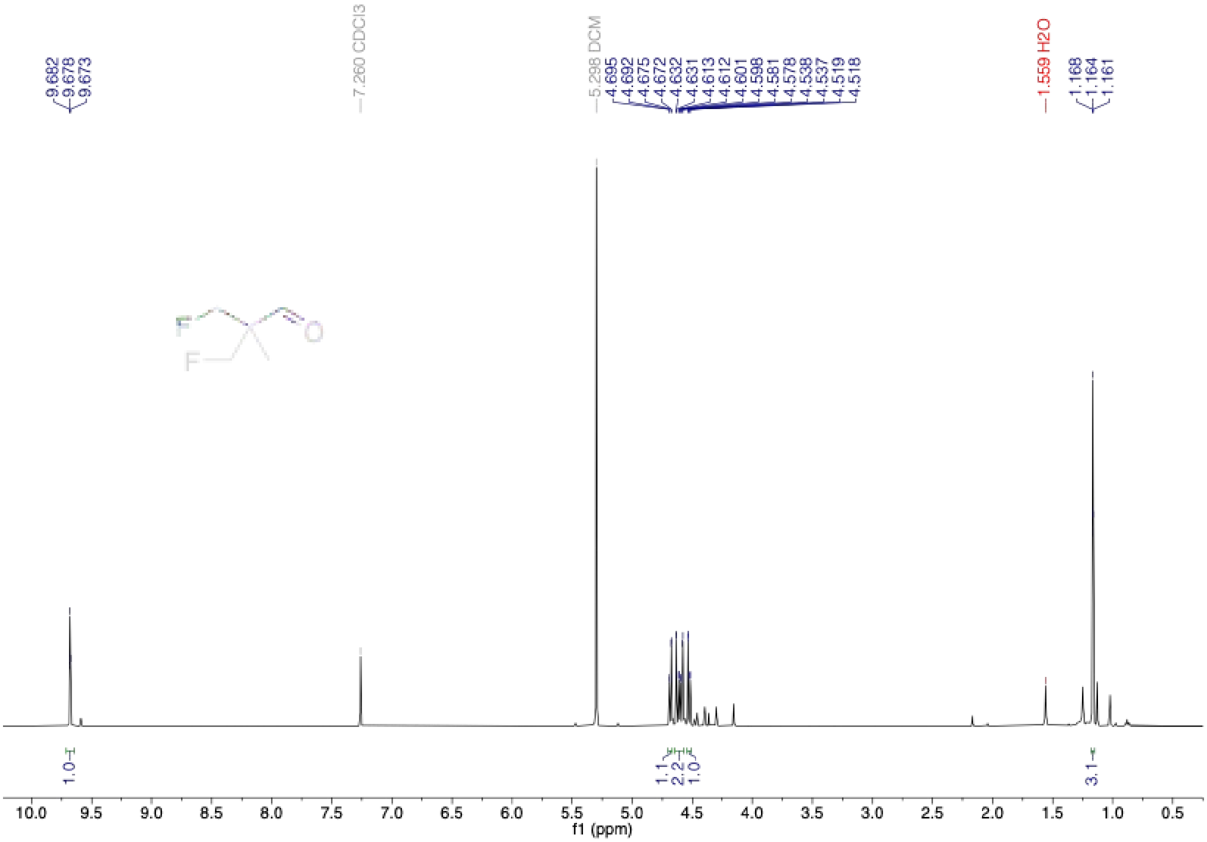

$^{19}F\{^{1}H\}$ NMR (470 MHz, $CDCl_3$)

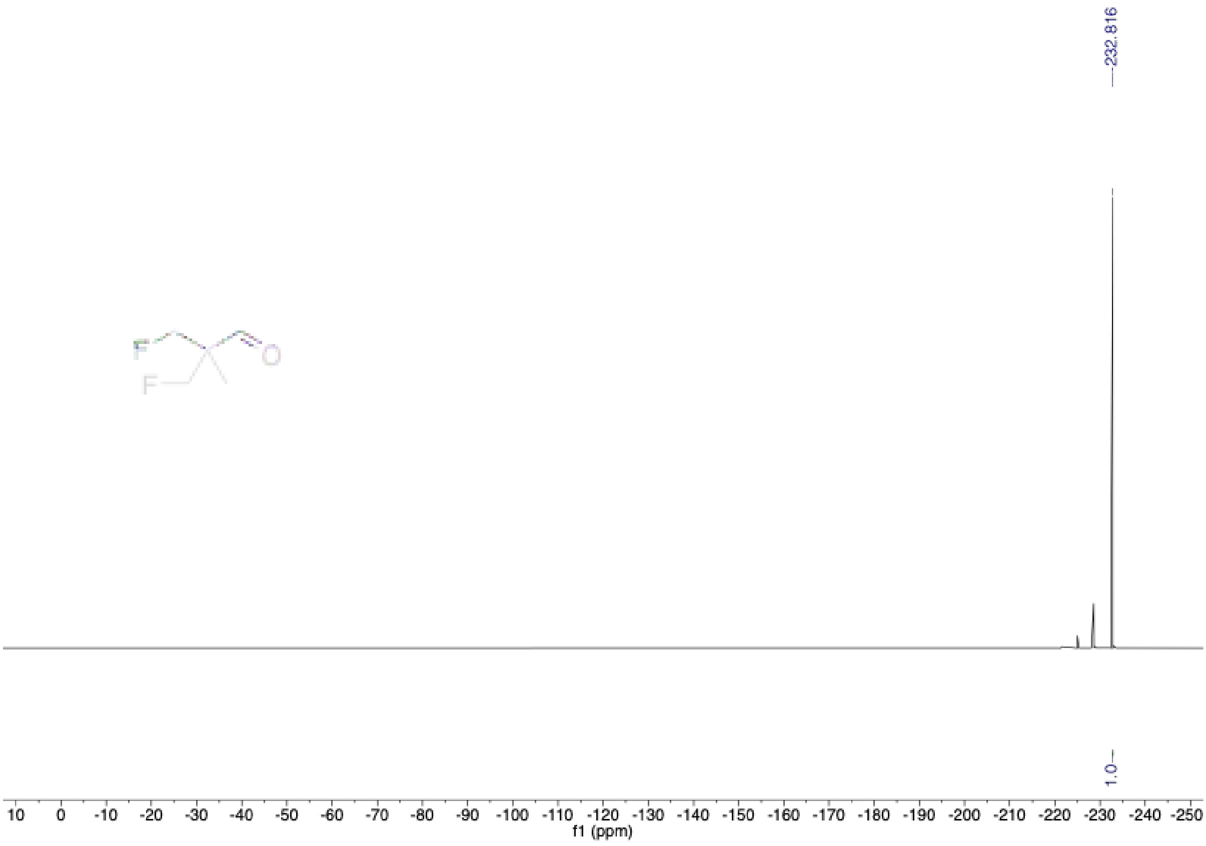

$^{13}C\{^{1}H\}$ NMR (126 MHz, $CDCl_3$)

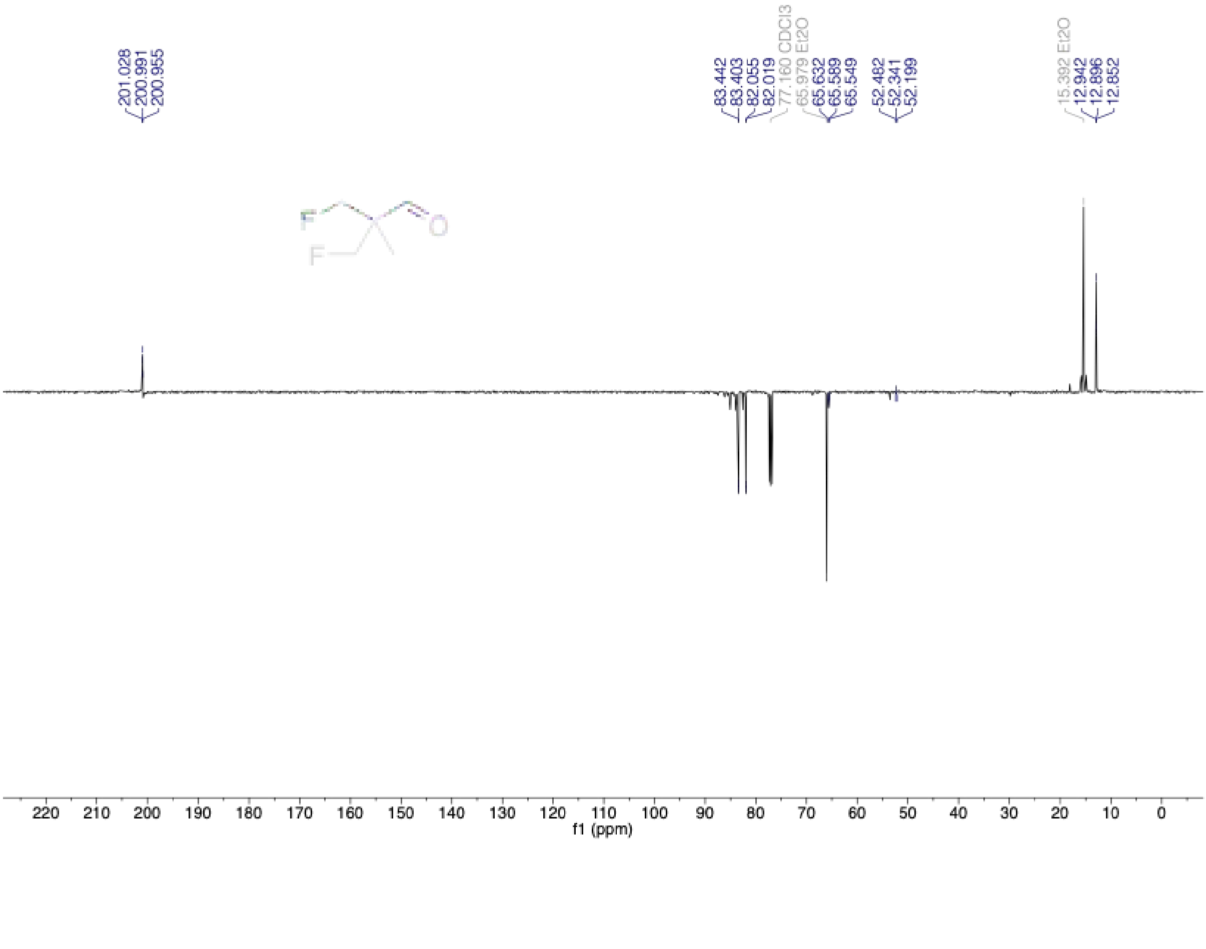

S9

^{1}H NMR (400 MHz, $CDCl_3$)

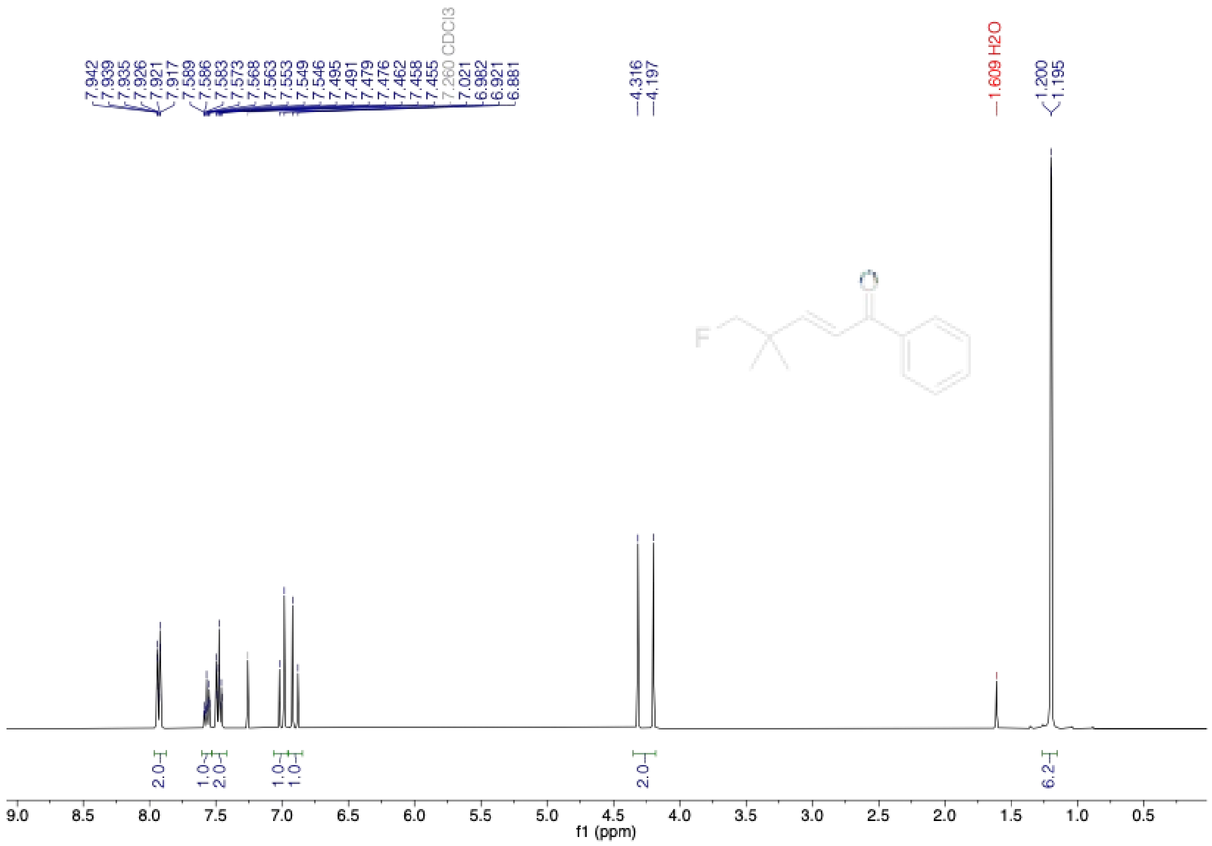

$^{19}F\{^{1}H\}$ NMR (377 MHz, $CDCl_3$)

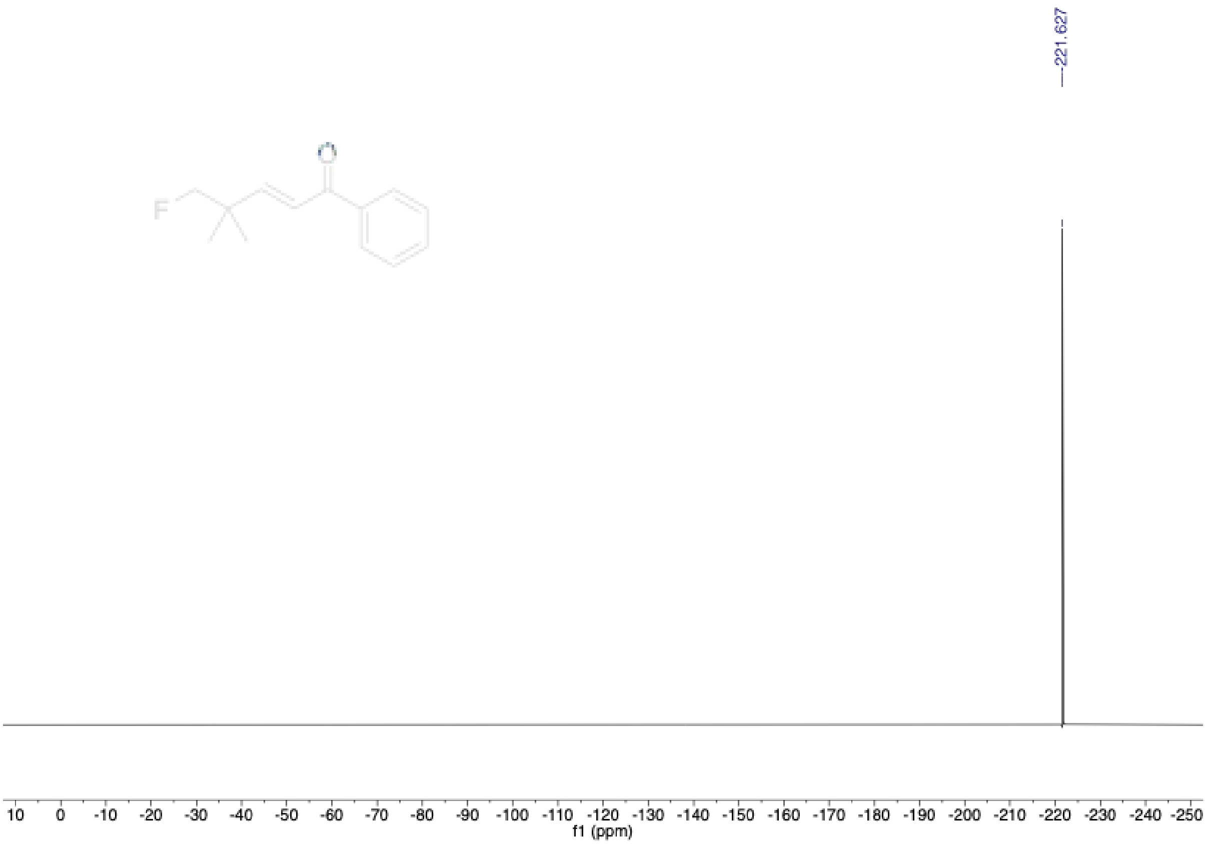

$^{13}C\{^{1}H\}$ NMR (126 MHz, $CDCl_3$)

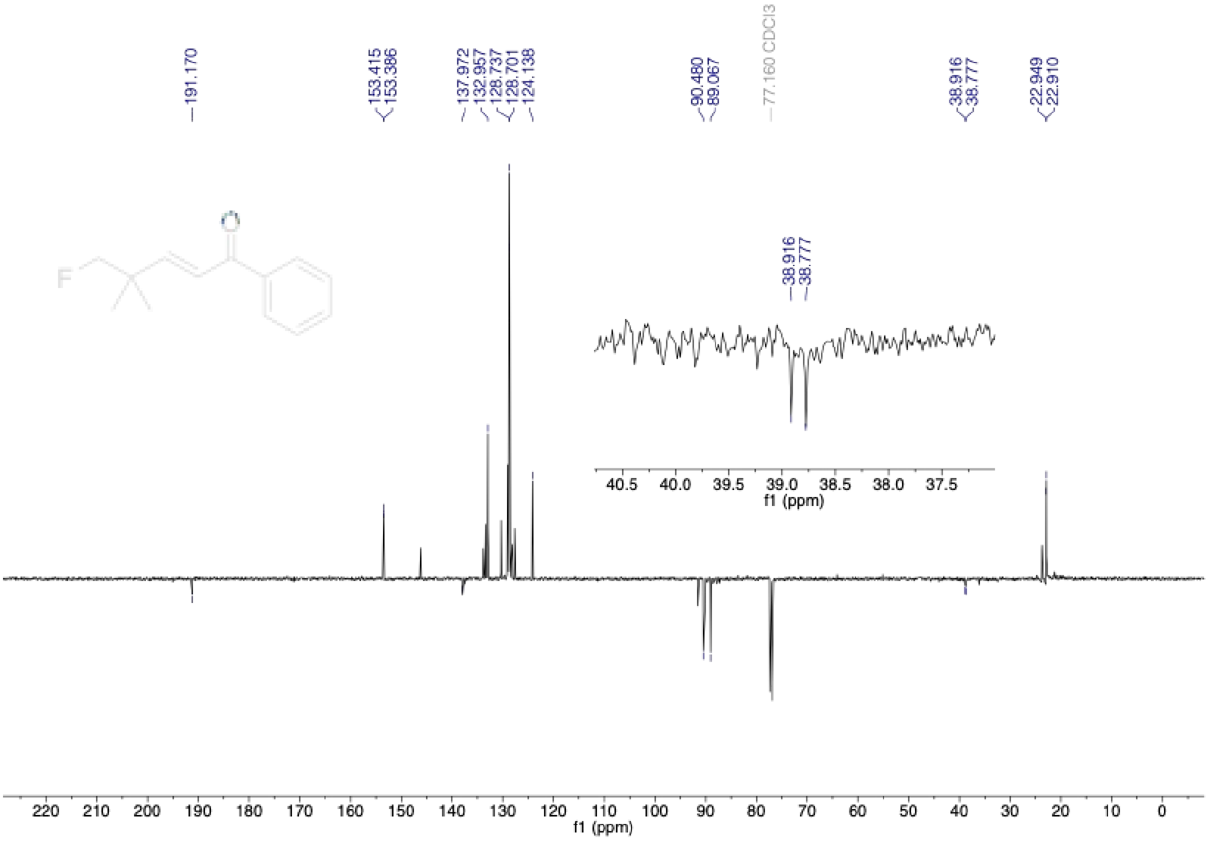

S10

^1H NMR (400 MHz, $CDCl_3$)

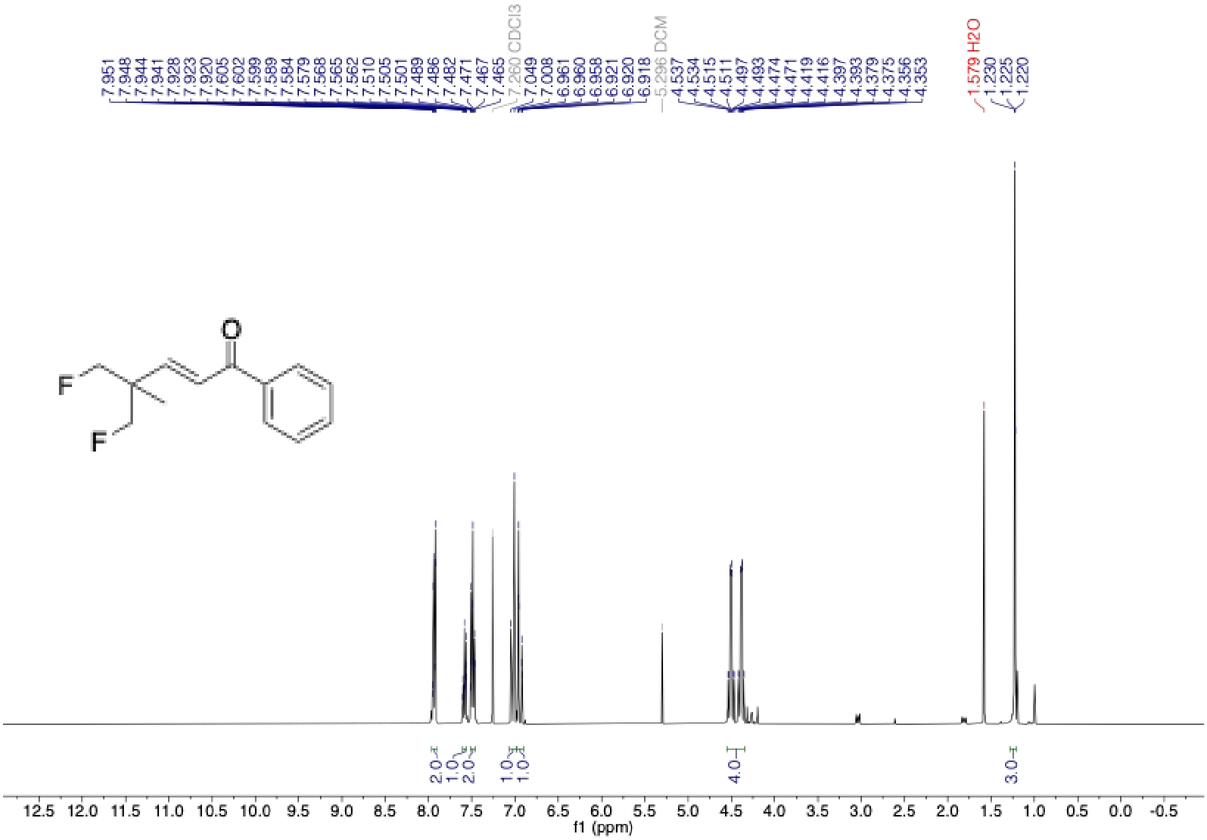

$^{19}F\{^1H\}$ NMR (377 MHz, $CDCl_3$)

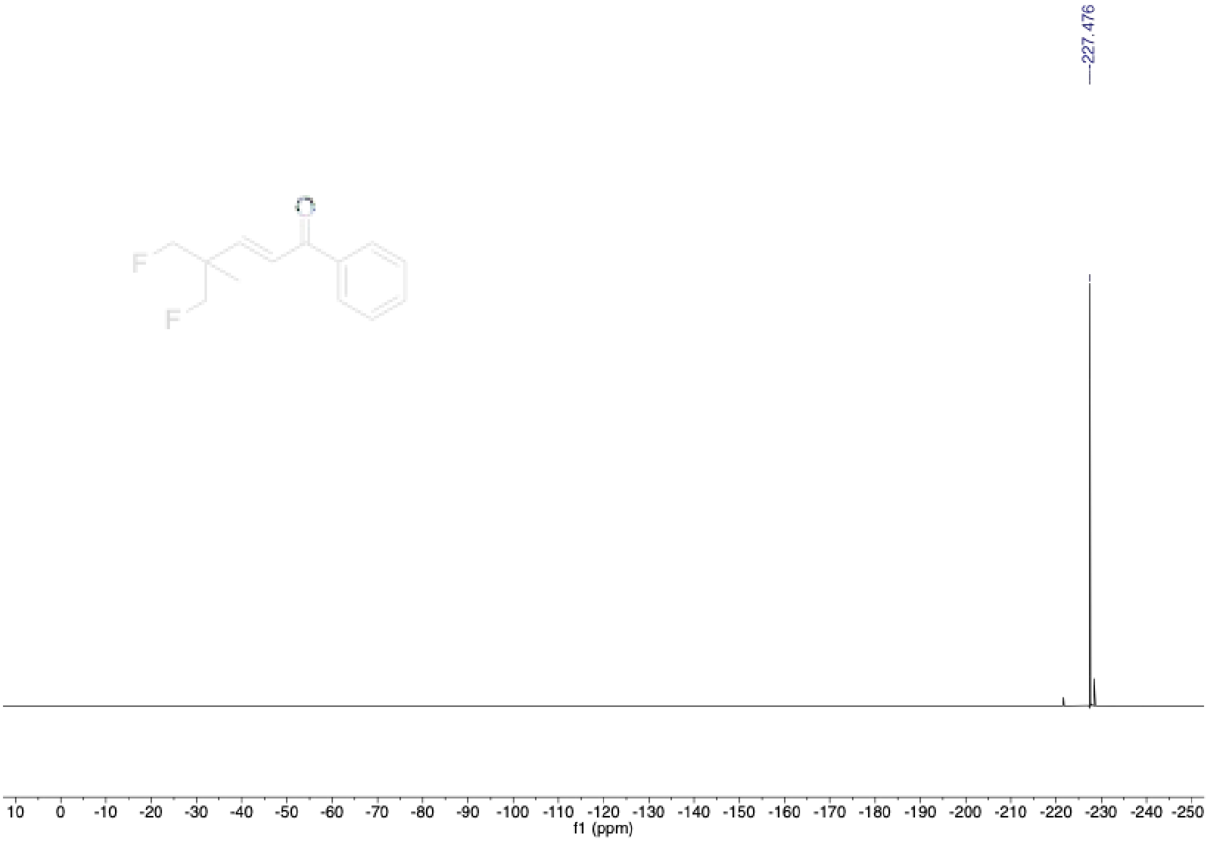

$^{13}C\{^1H\}$ NMR (101 MHz, $CDCl_3$)

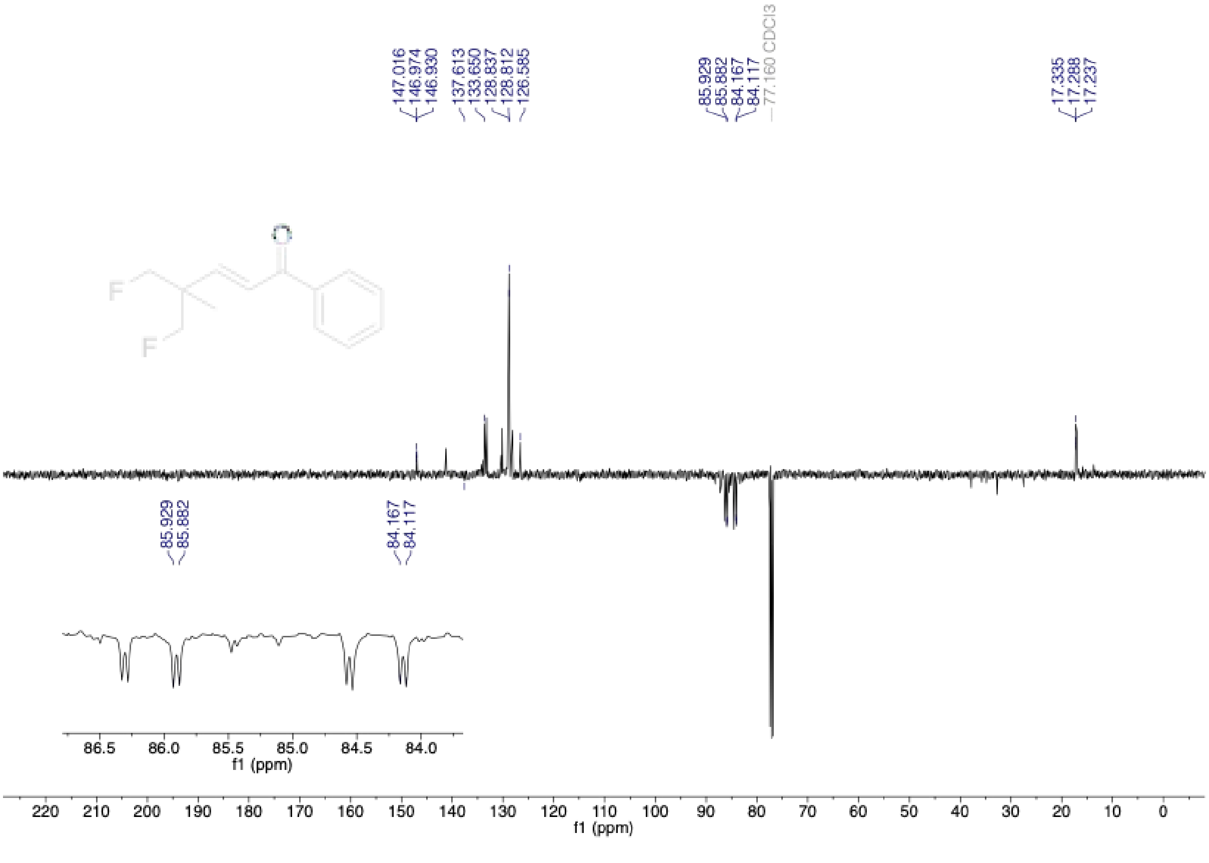

^{1}H-^{13}C HMBC ($CDCl_3$)

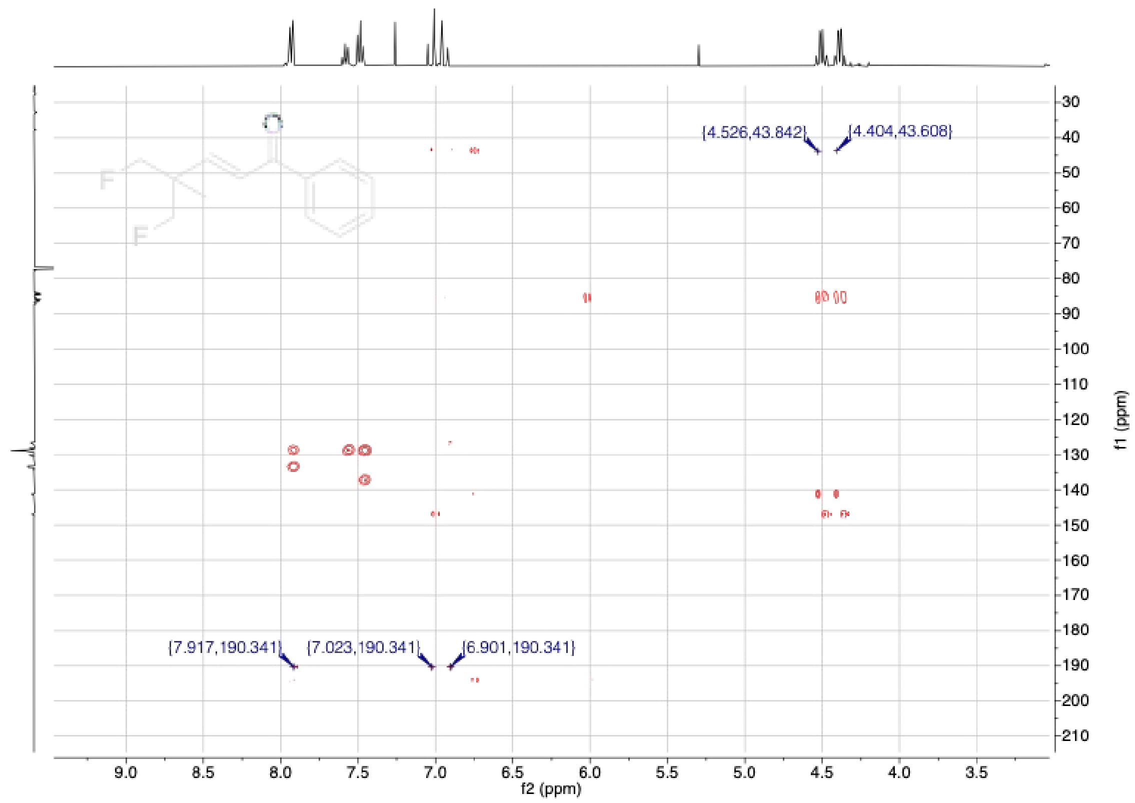

37

^{1}H NMR (500 MHz, $CDCl_3$)

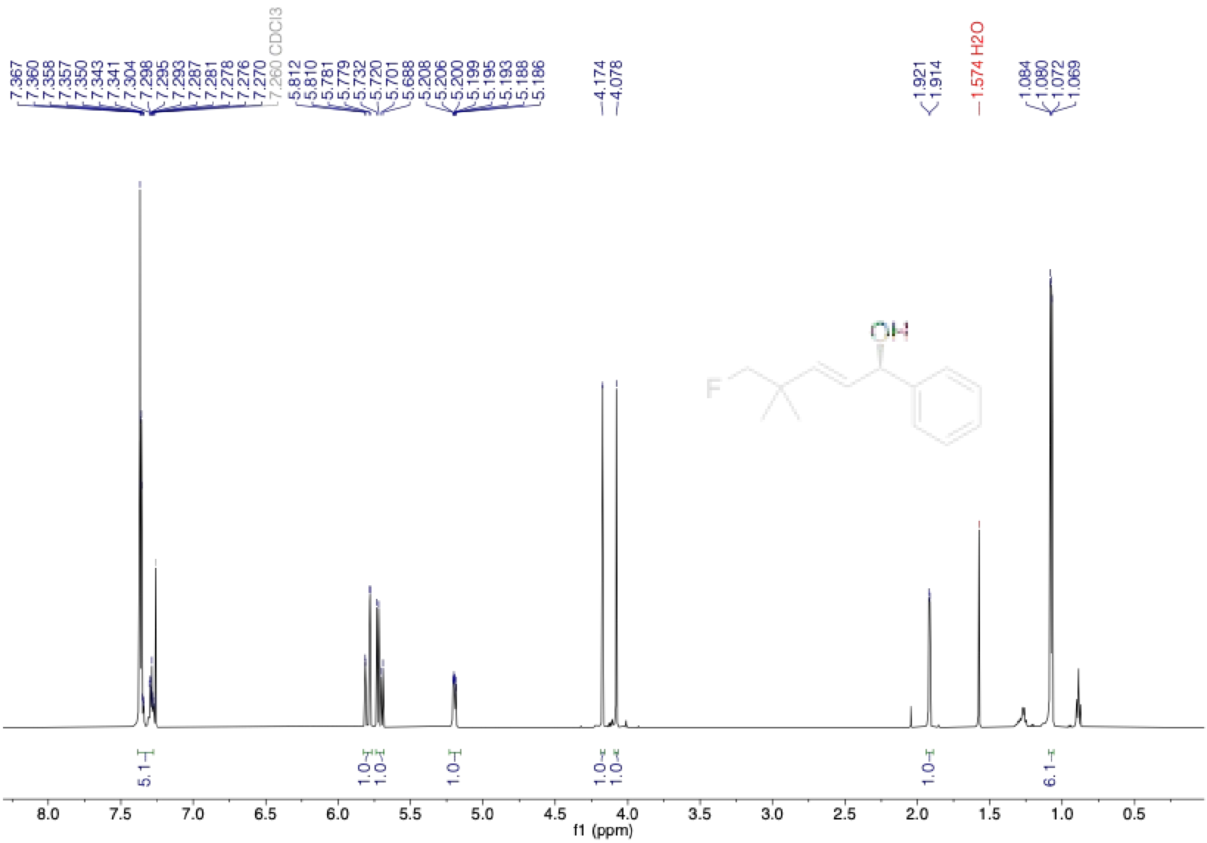

$^{19}F\{^1H\}$ NMR (470 MHz, $CDCl_3$)

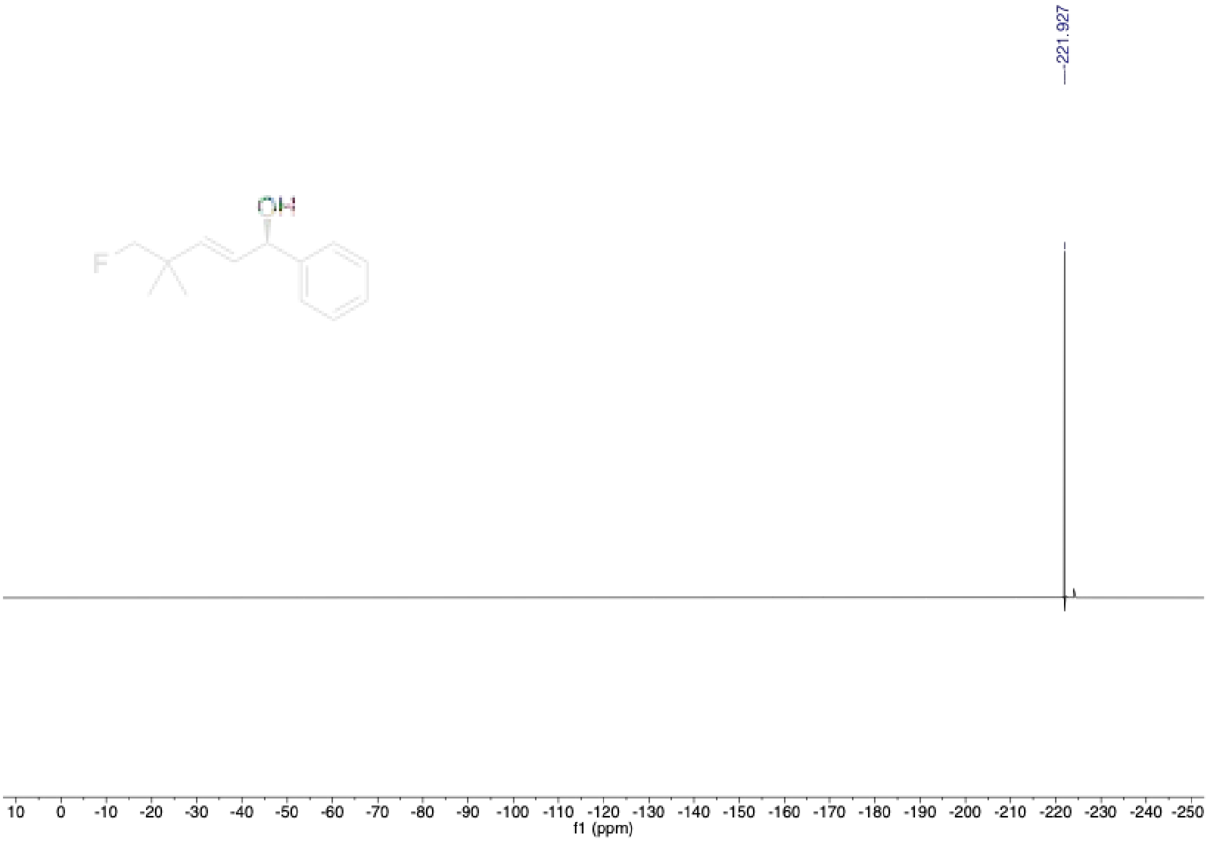

$^{13}C\{^1H\}$ NMR (126 MHz, $CDCl_3$)

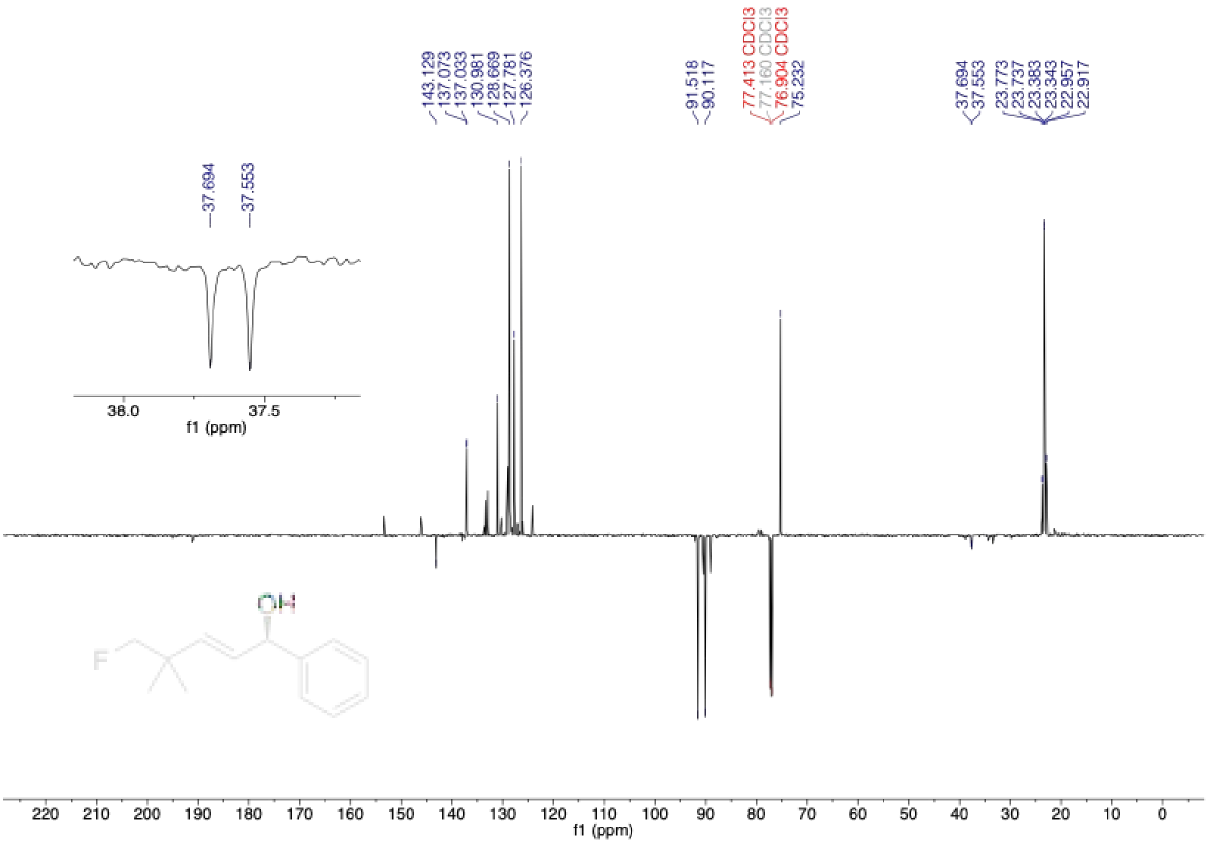
143.129
137.073
137.033
130.981
128.669
127.781
126.376
91.518
90.117
77.413 CDCl3
77.160 CDCl3
76.904 CDCl3
75.232
37.694
37.553
23.773
23.737
23.383
23.343
22.957
22.917
37.694
37.553
38.0
37.5
f1 (ppm)
OH
F
220 210 200 190 180 170 160 150 140 130 120 110 100 90 80 70 60 50 40 30 20 10 0
f1 (ppm)

38

^{1}H NMR (500 MHz, $CDCl_3$)

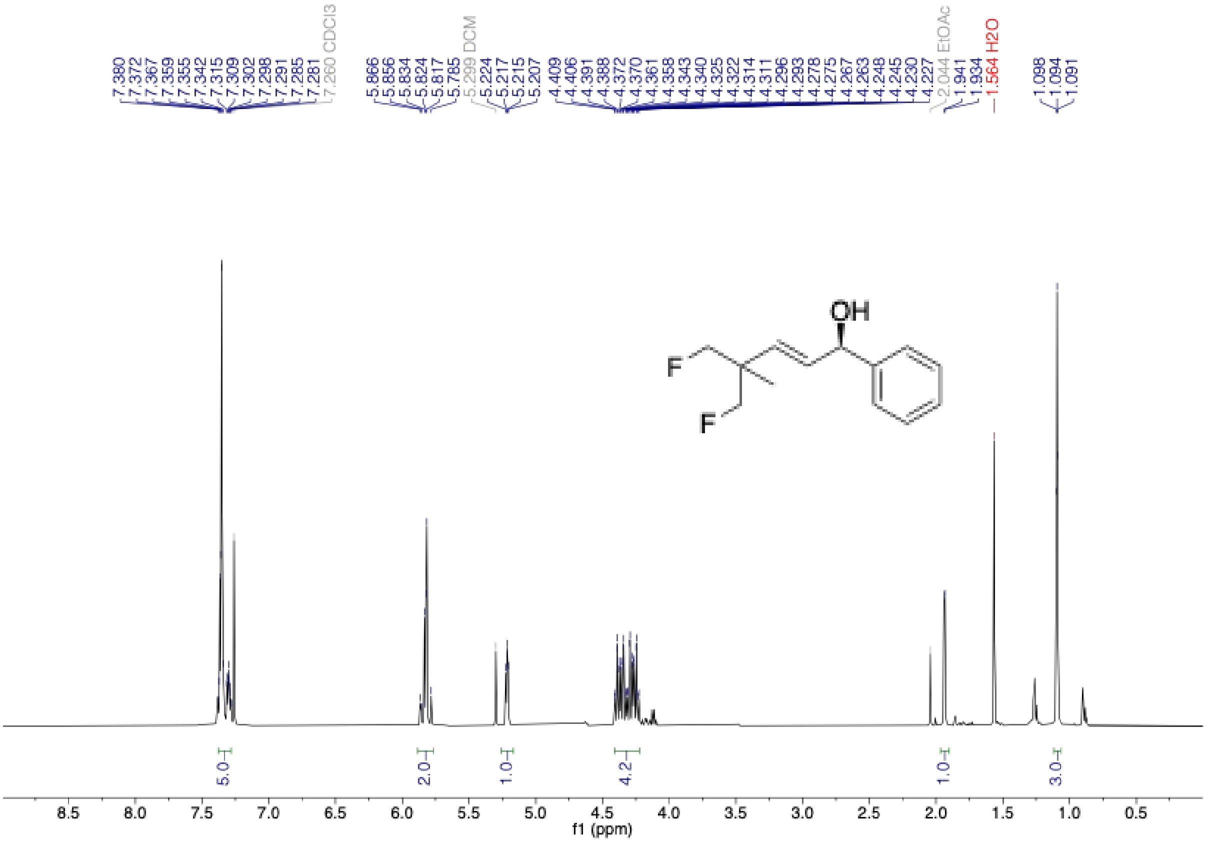

$^{19}F\{^{1}H\}$ NMR (470 MHz, $CDCl_3$)

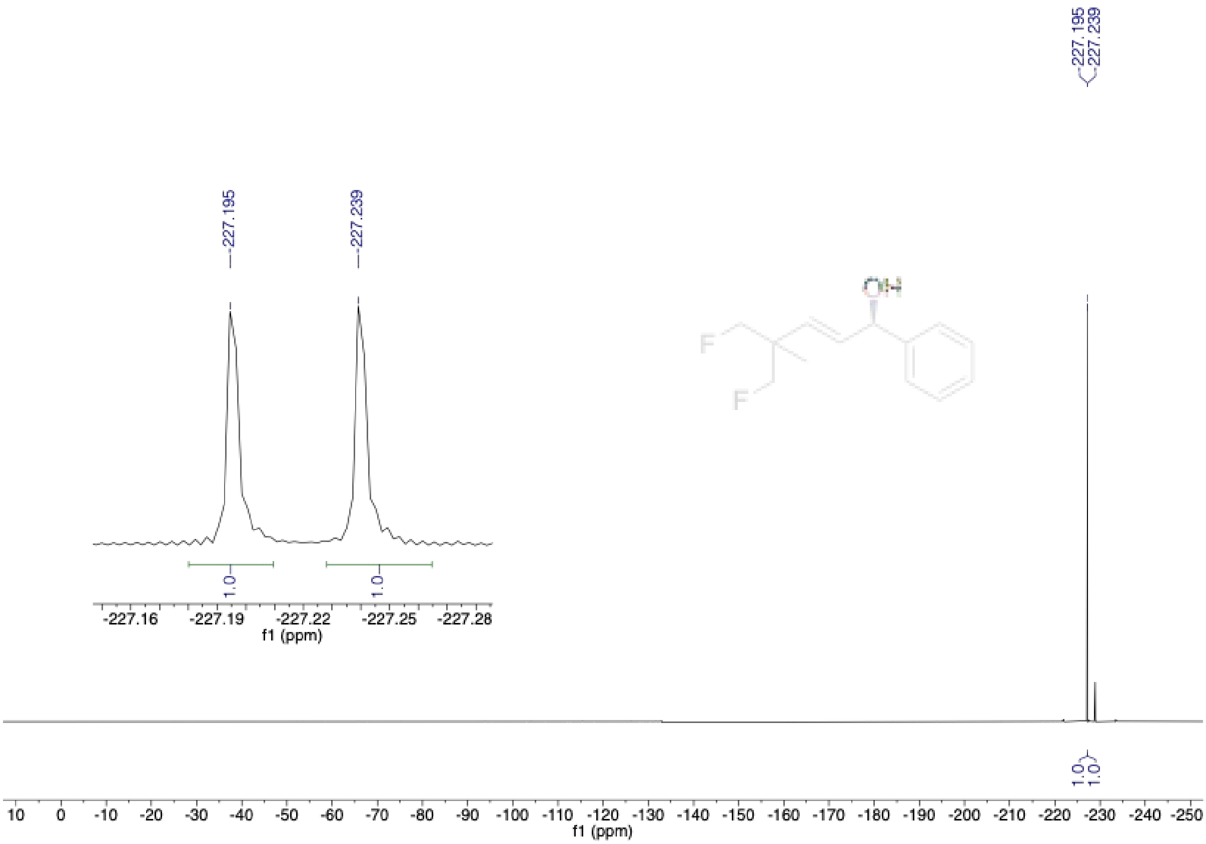

$^{13}C\{^1H\}$ NMR (126 MHz, $CDCl_3$)

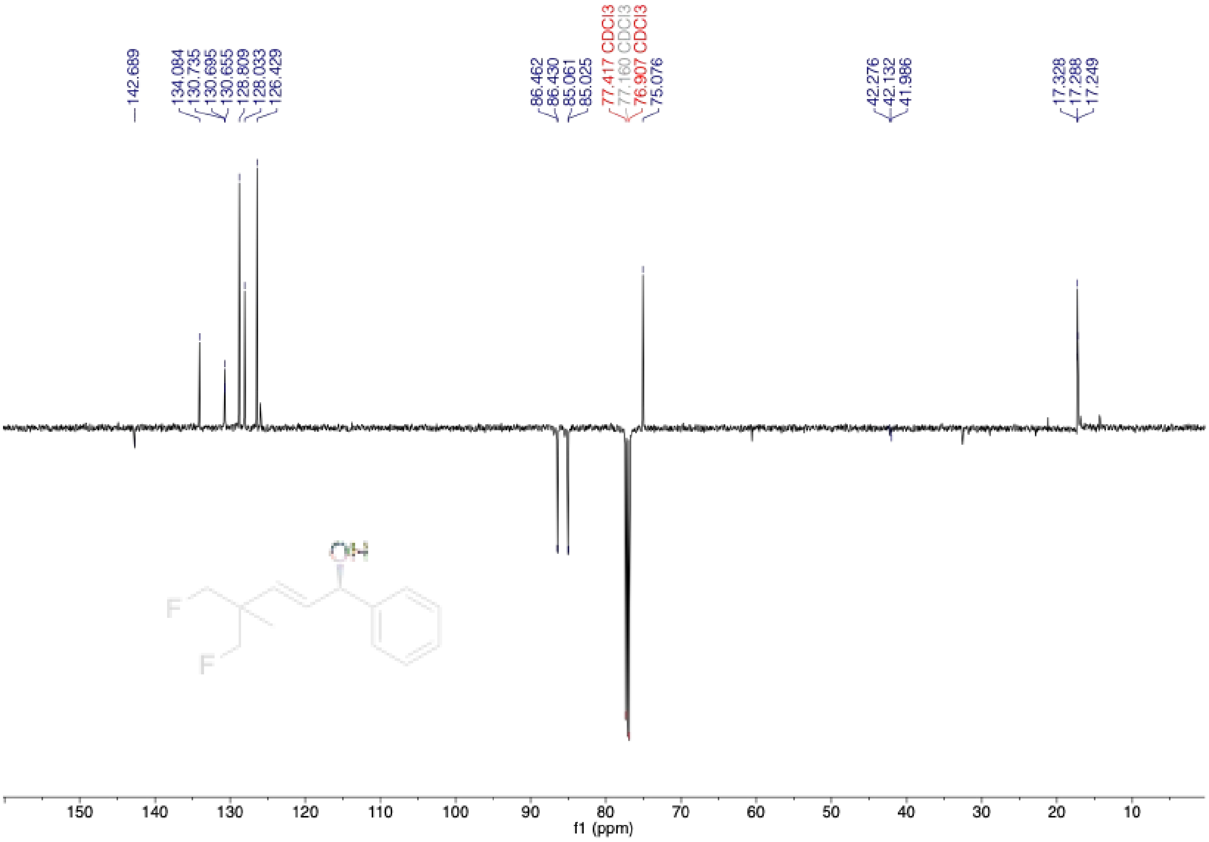

39

^{1}H NMR (500 MHz, $CDCl_3$)

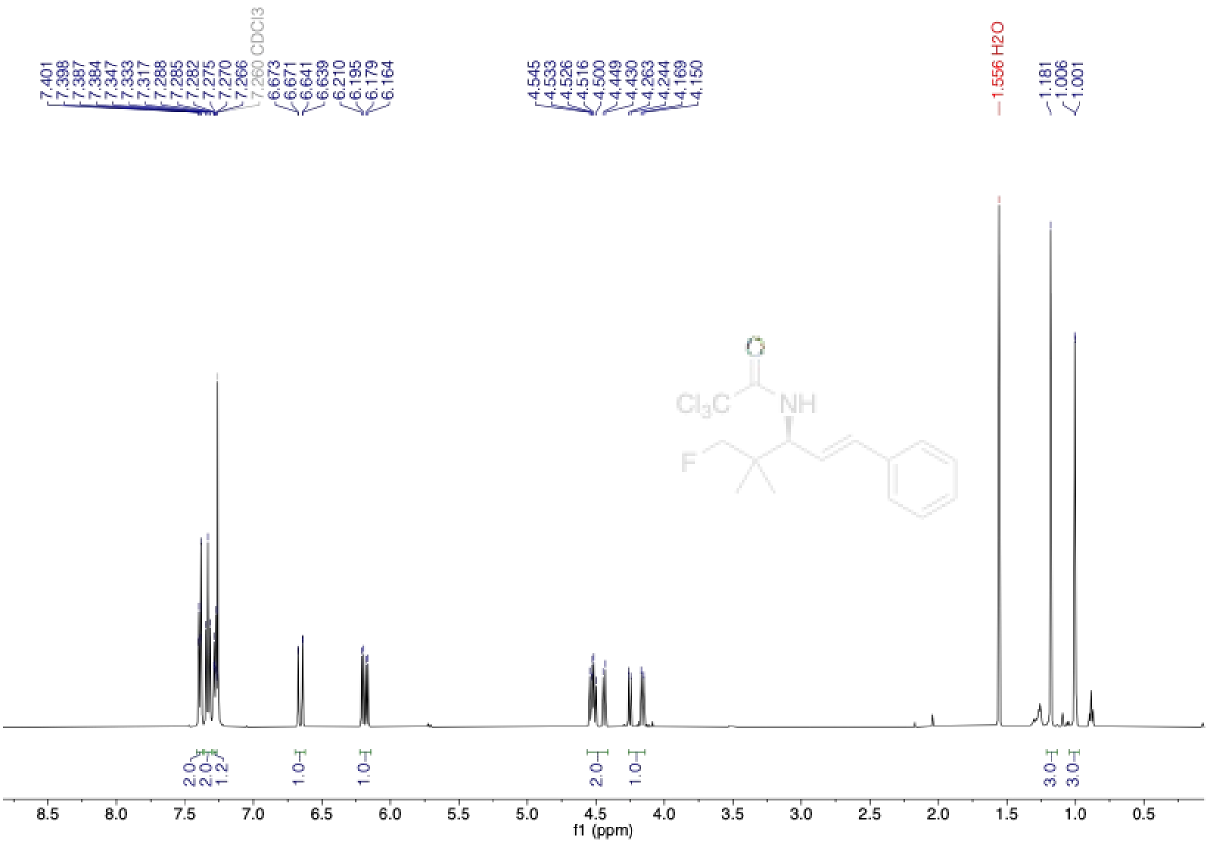

$^{19}F\{^{1}H\}$ NMR (470 MHz, $CDCl_3$)

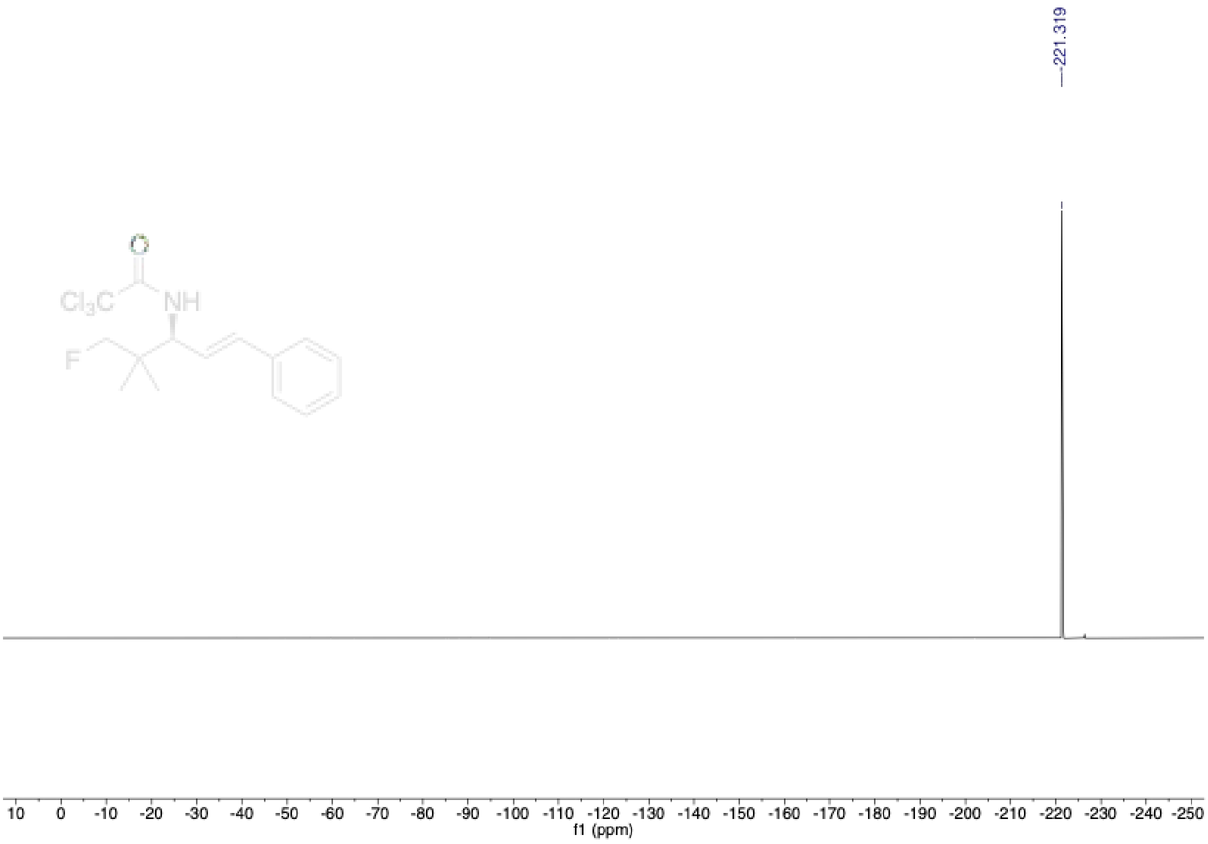

$^{13}C\{^{1}H\}$ NMR (126 MHz, $CDCl_3$)

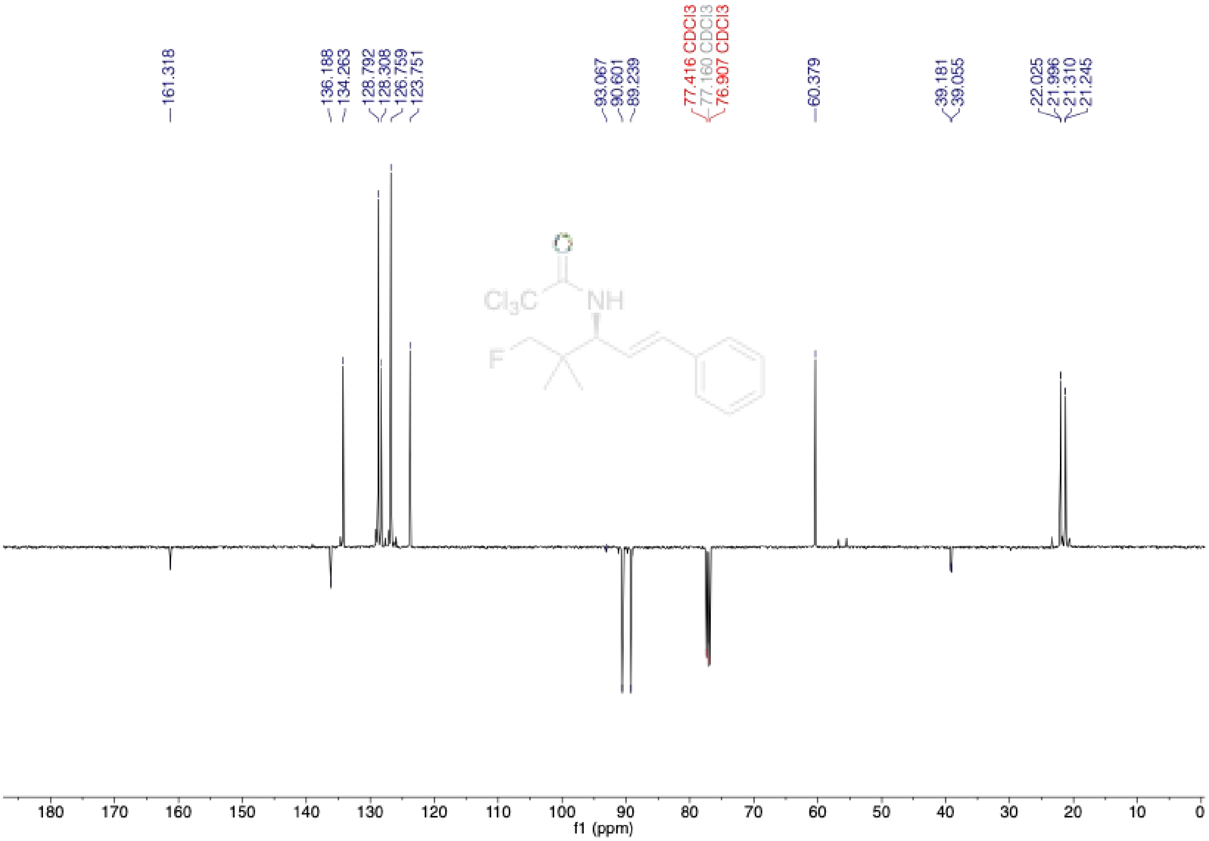

40

^{1}H NMR (500 MHz, $CDCl_3$)

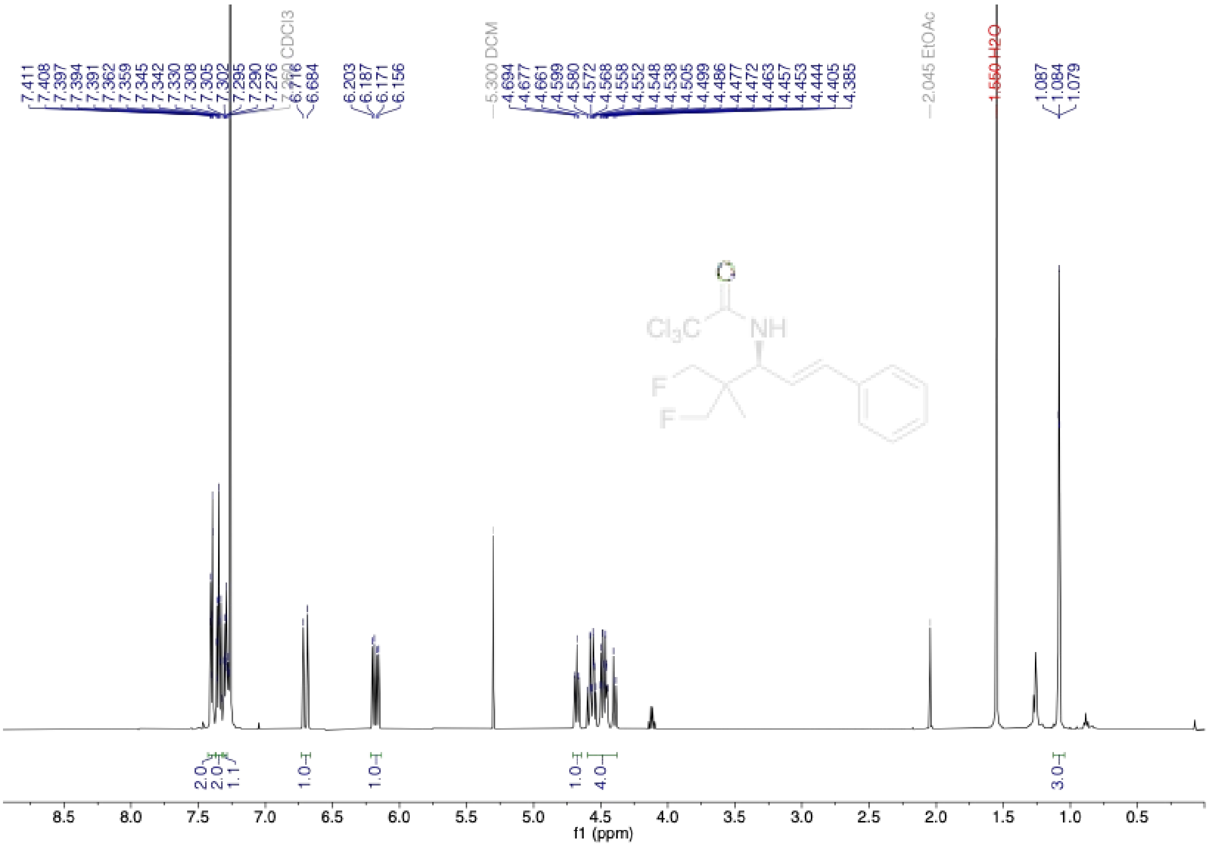

^{9}F{^{1}H} NMR (470 MHz, $CDCl_3$)

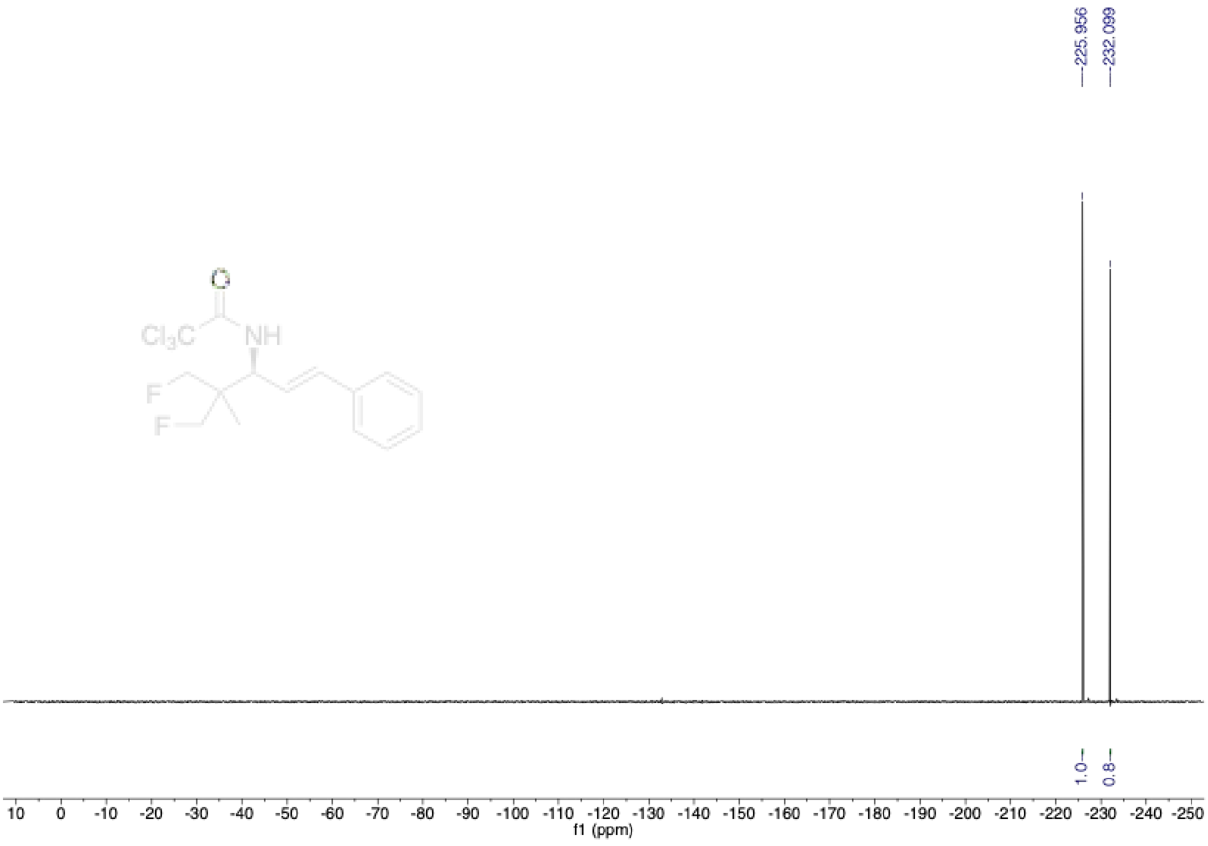

$^{13}C\{^{1}H\}$ NMR (126 MHz, $CDCl_3$)

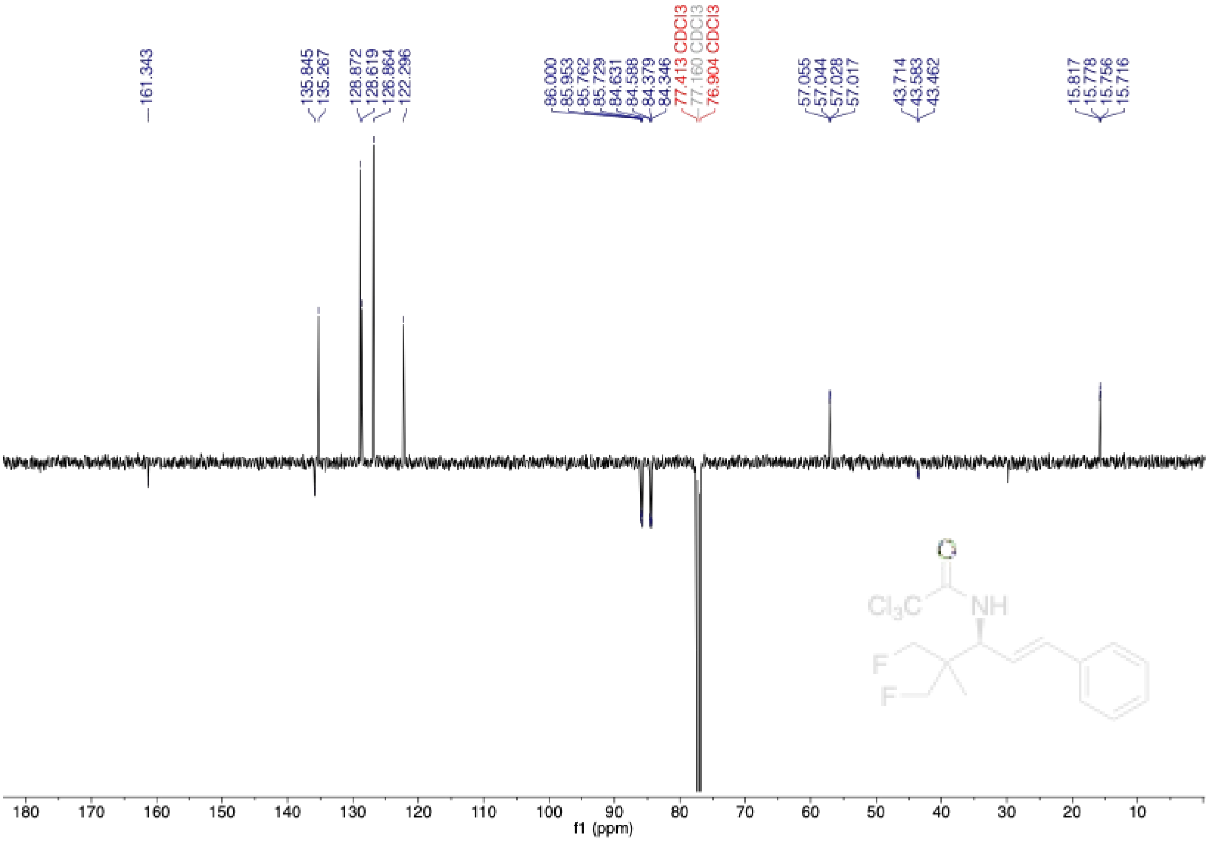

41

^{1}H NMR (500 MHz, D_2O)

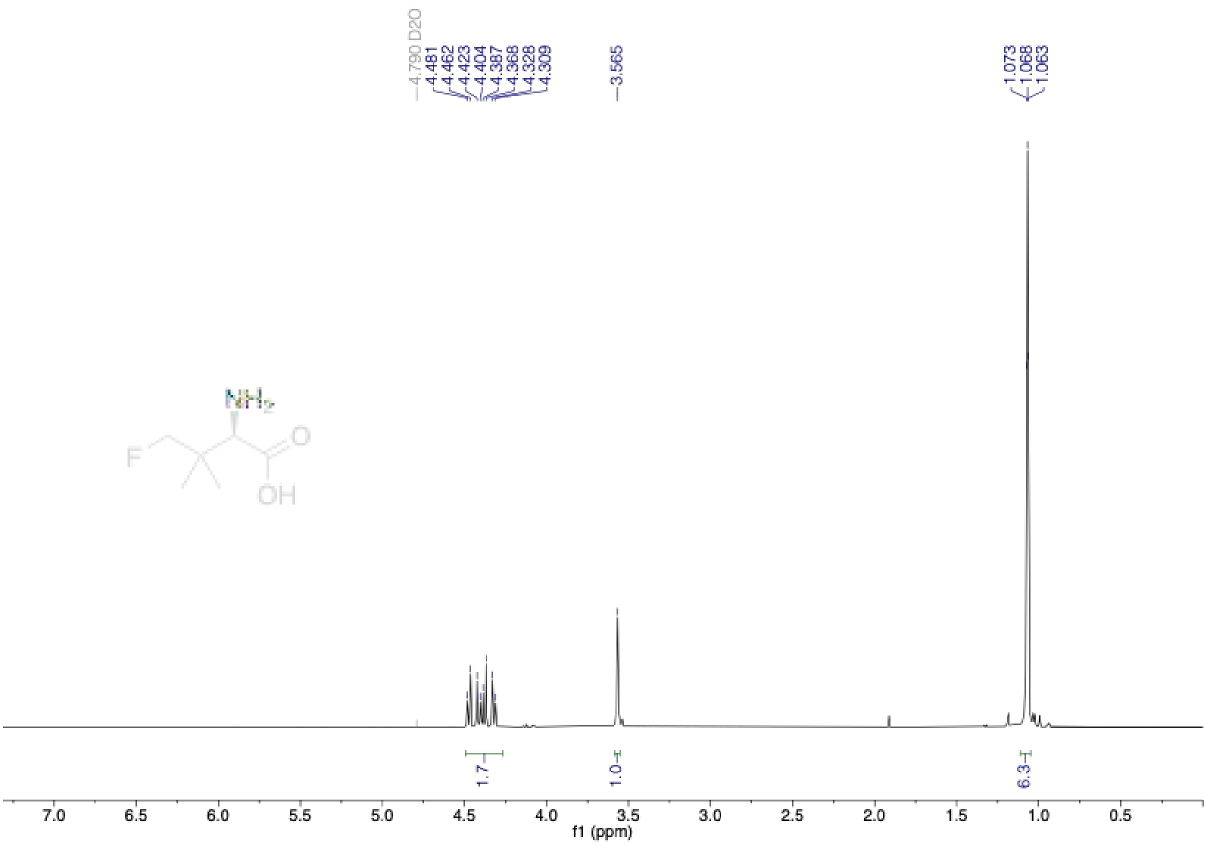

^{19}F{^{1}H} NMR (470 MHz, D_2O)

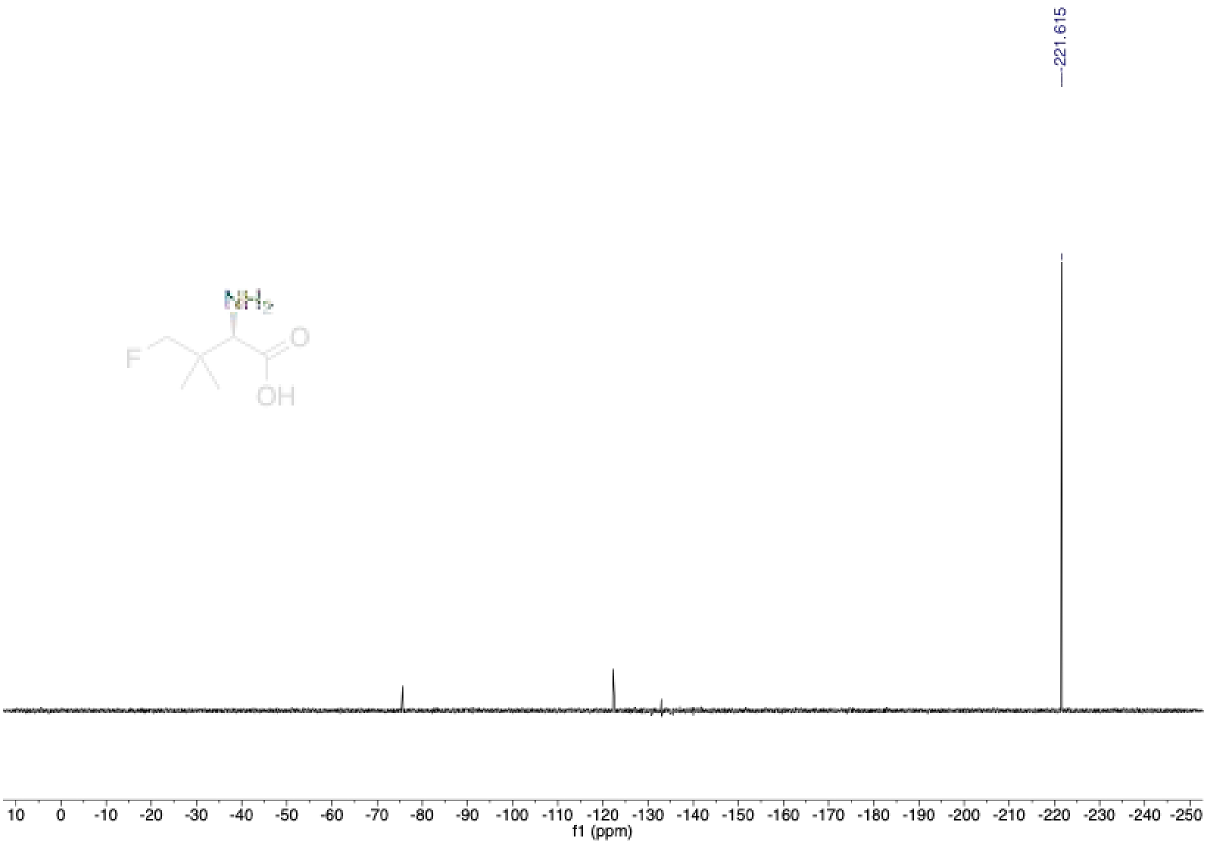

$^{13}C\{^{1}H\}$ NMR (176 MHz, D_2O)

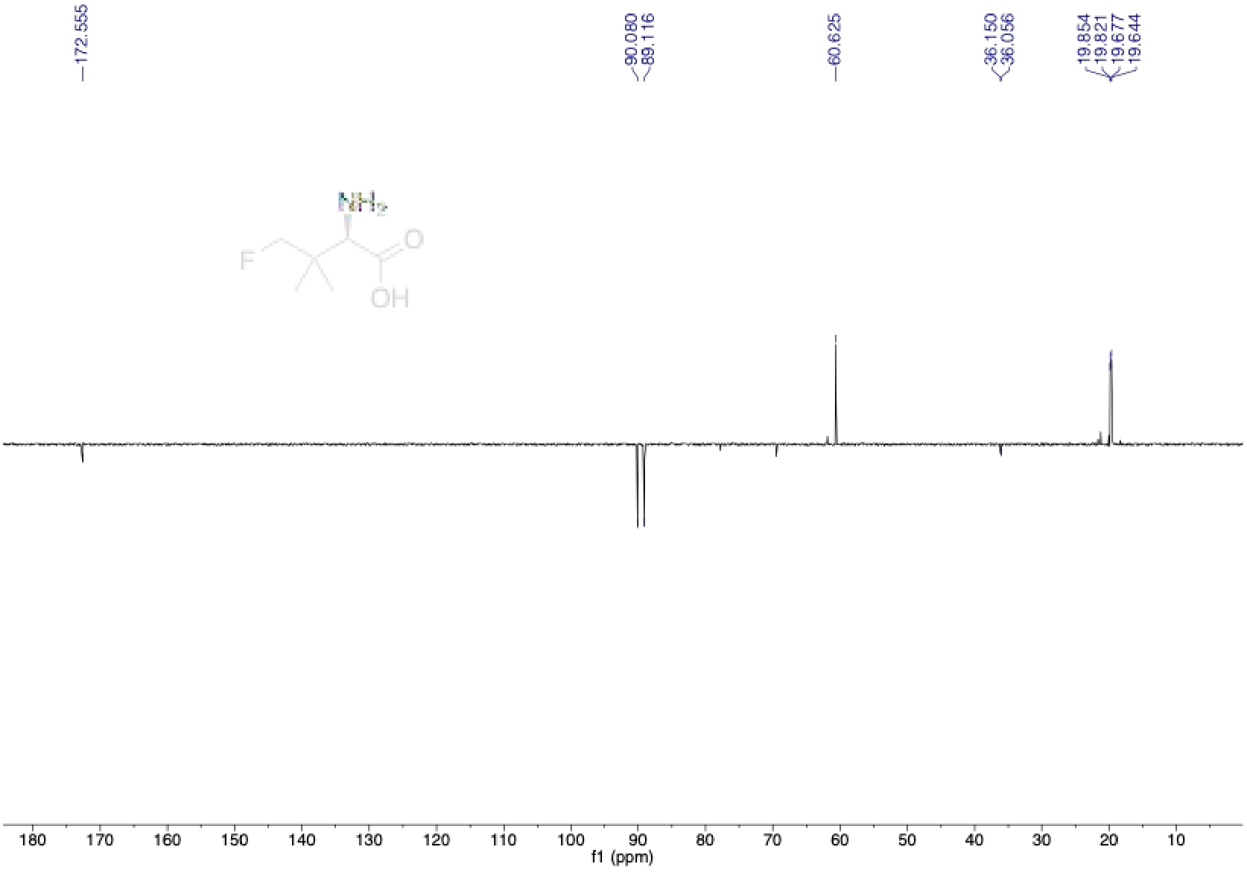

42

^{1}H NMR (500 MHz, D_2O)

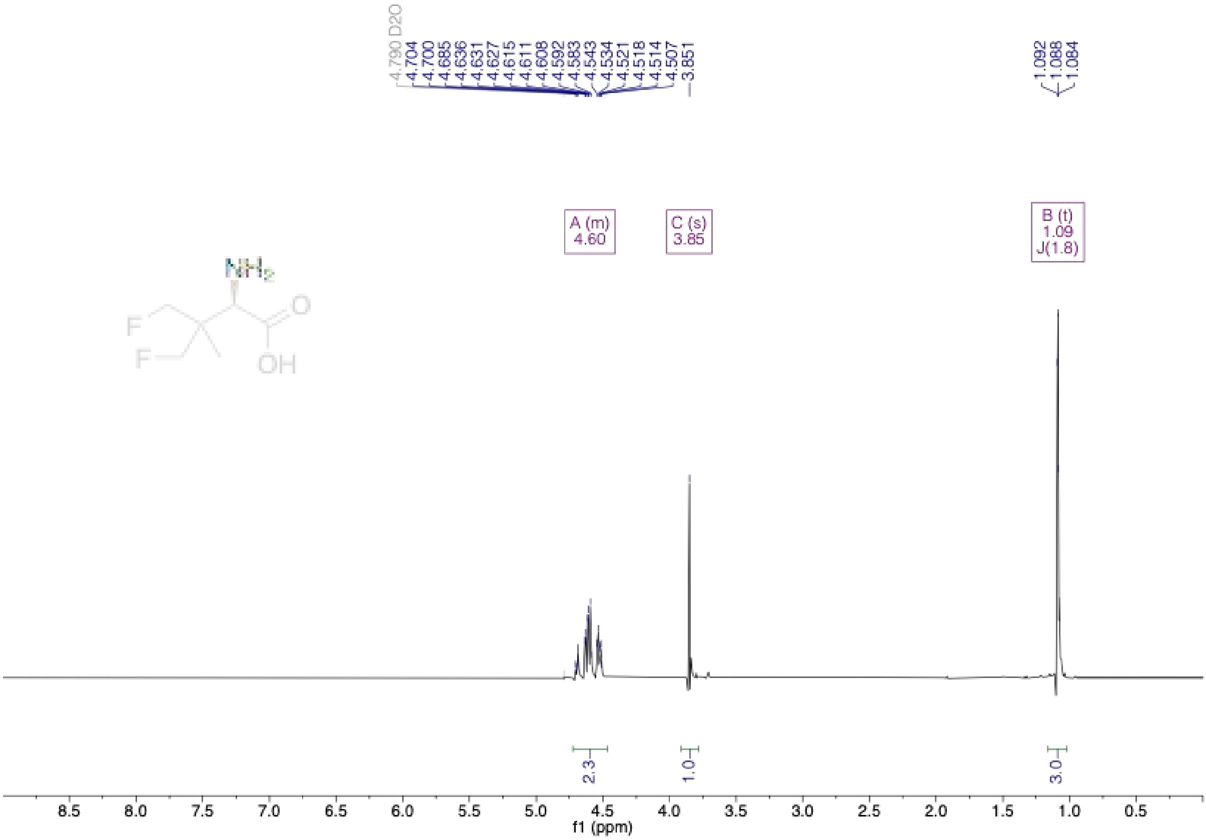

^{19}F NMR (471 MHz, D_2O)

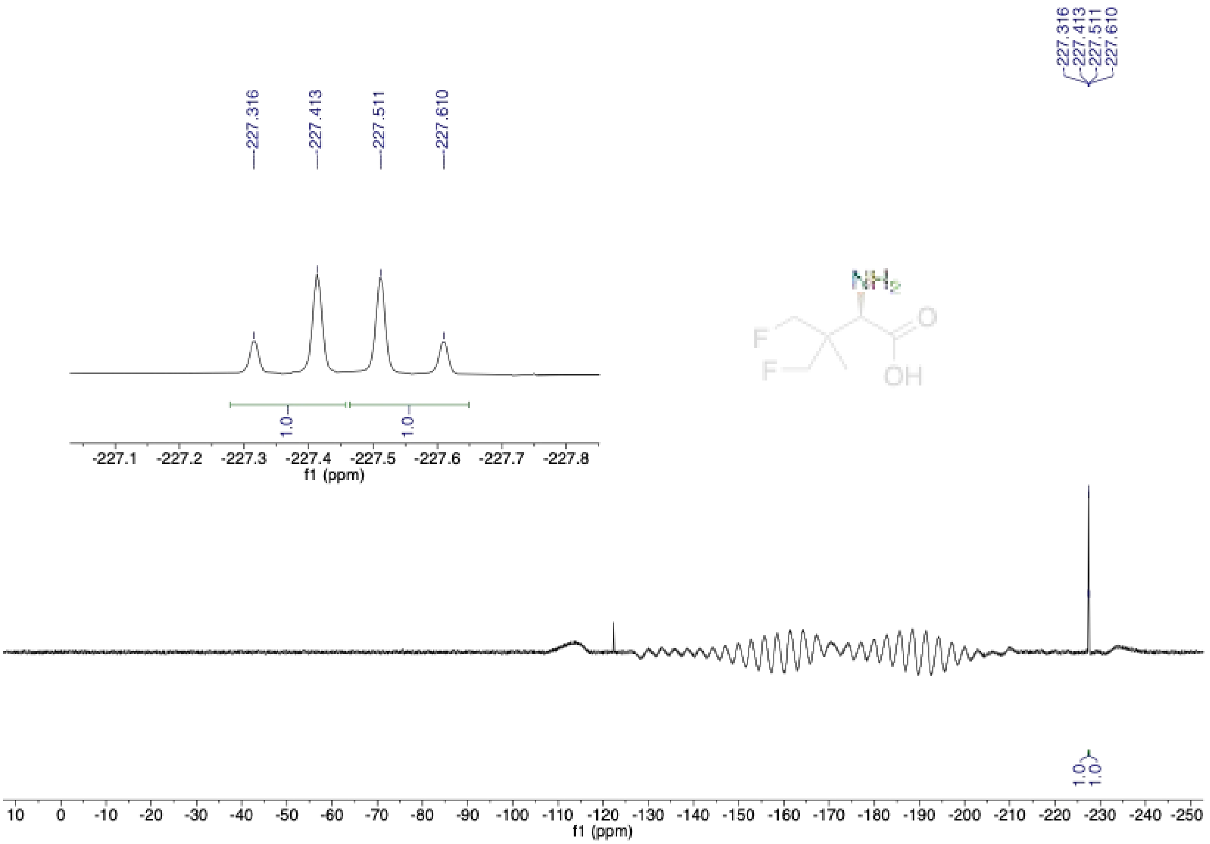

$^{13}C\{^{1}H\}$ NMR (126 MHz, D_2O)

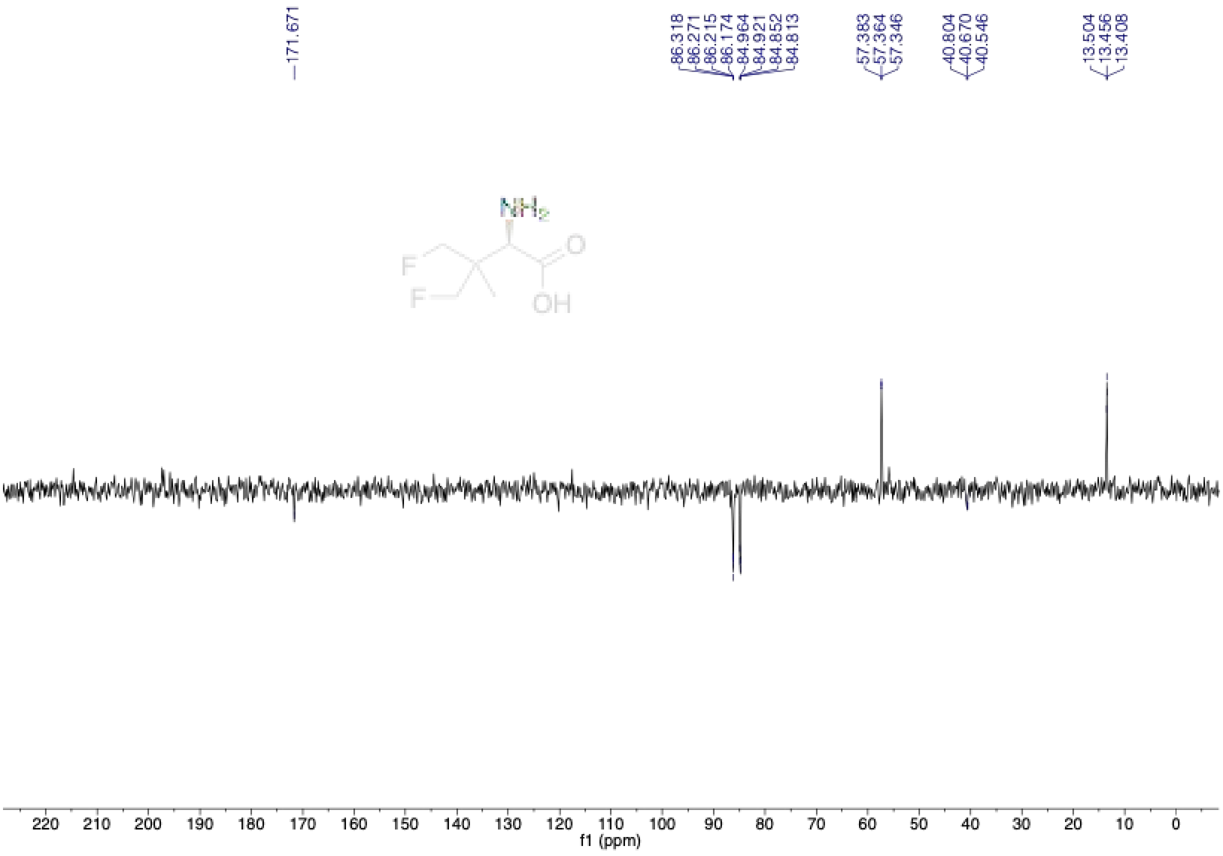

43

^{1}H NMR (500 MHz, $CDCl_3$)

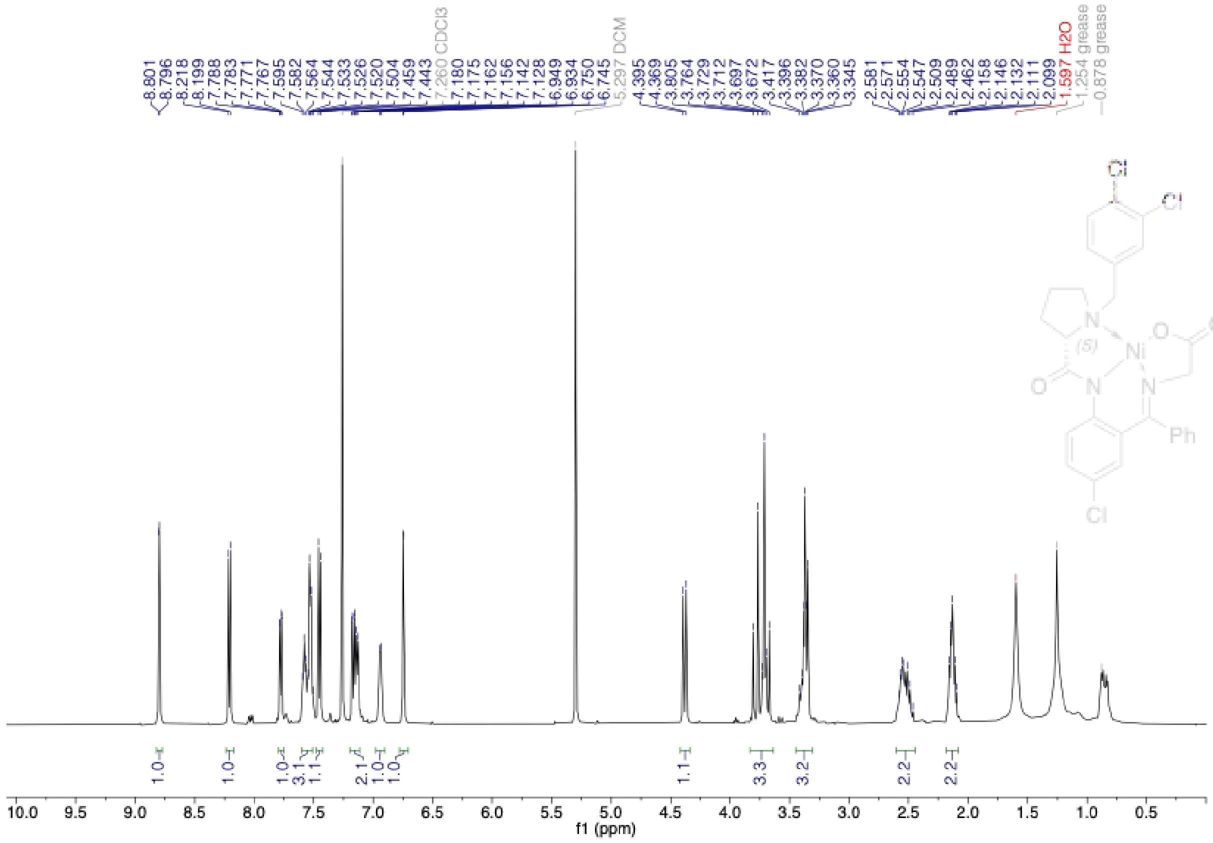

44

^{1}H NMR (500 MHz, $CDCl_3$)

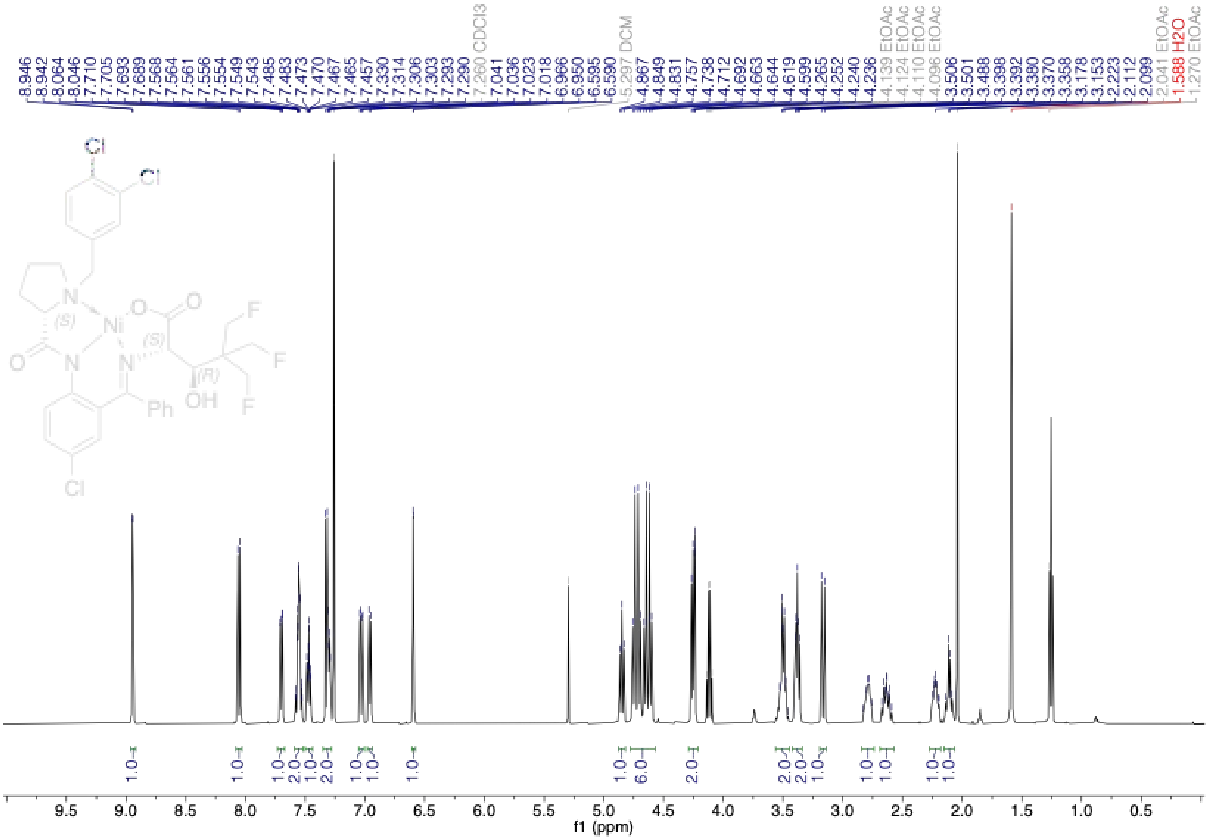

$^{19}F\{^{1}H\}$ NMR (470 MHz, $CDCl_3$)

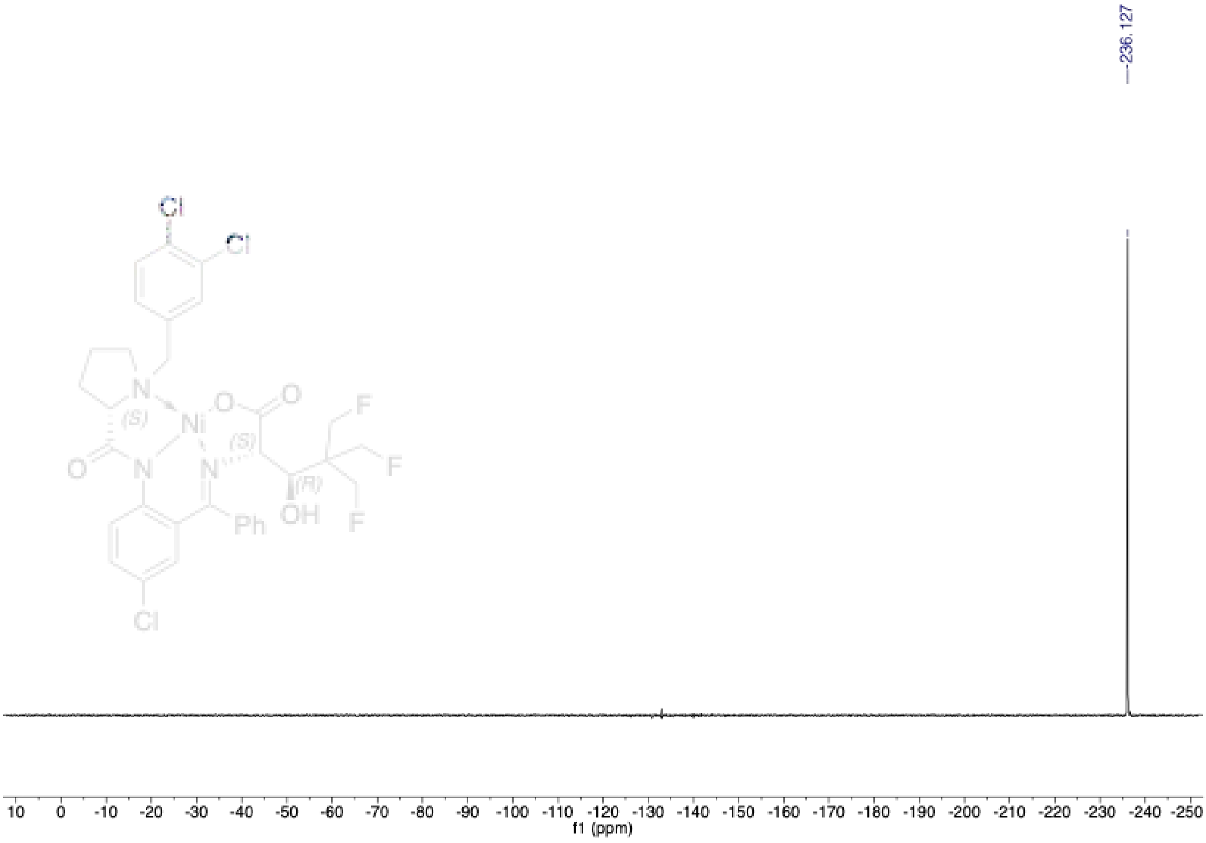

$^{13}C\{^{1}H\}$ NMR (126 MHz, $CDCl_3$)

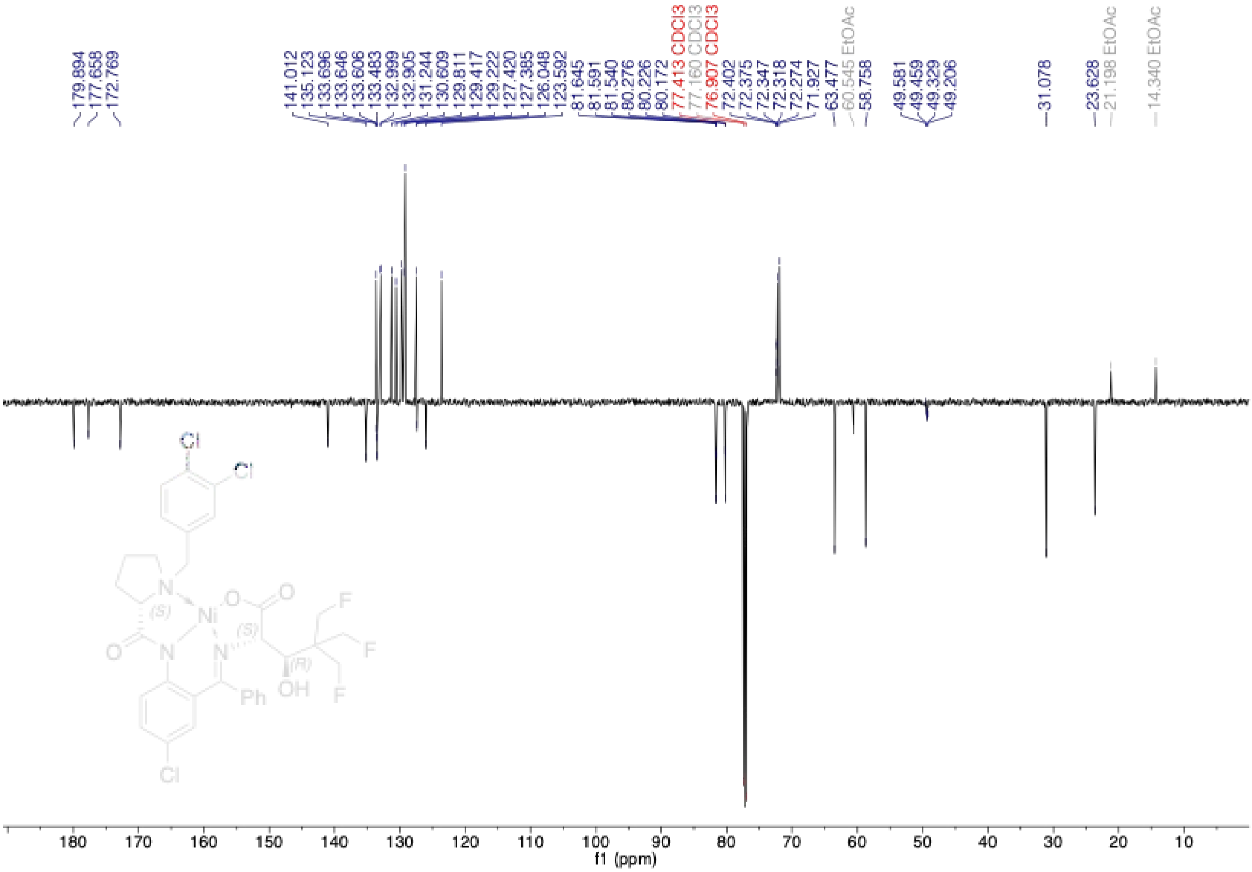

46

^{1}H NMR (500 MHz, D_2O)

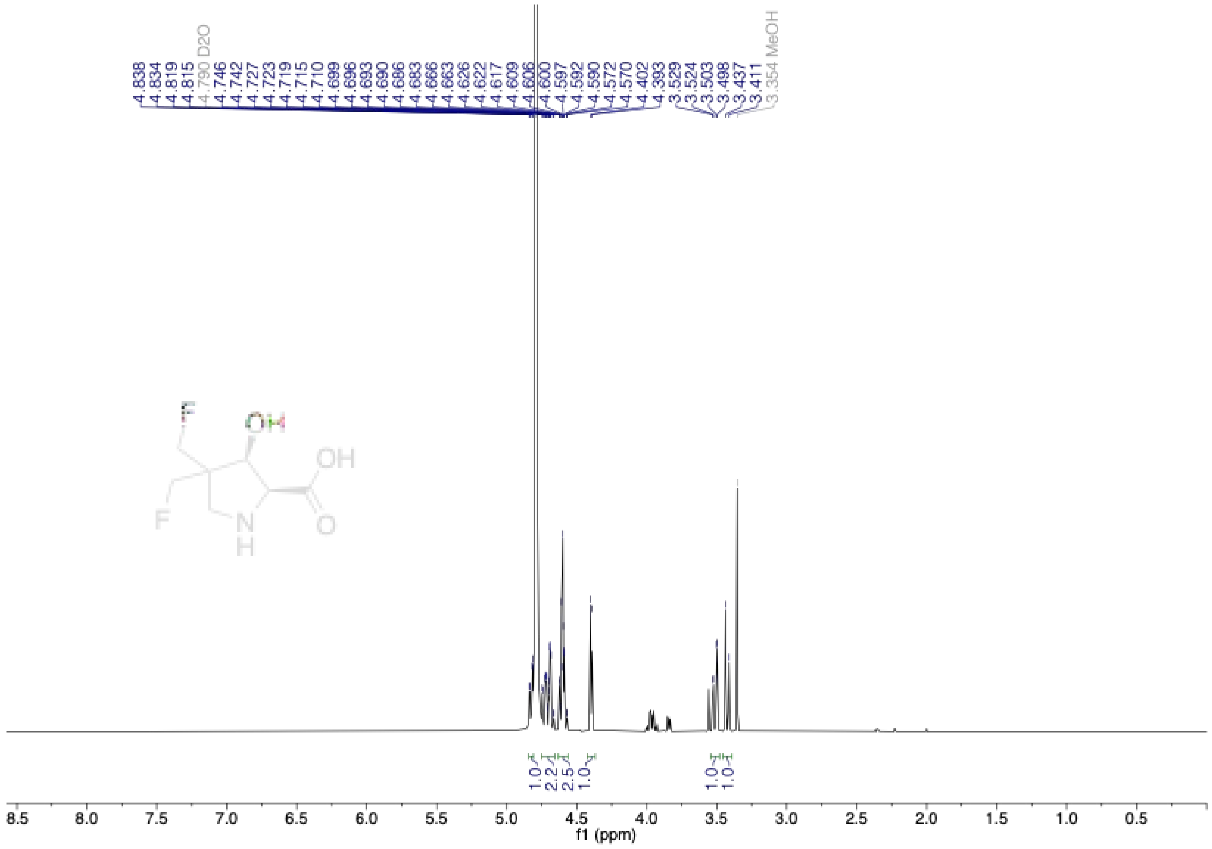

^{1}H-^{19}F HMBC NMR (D_2O)

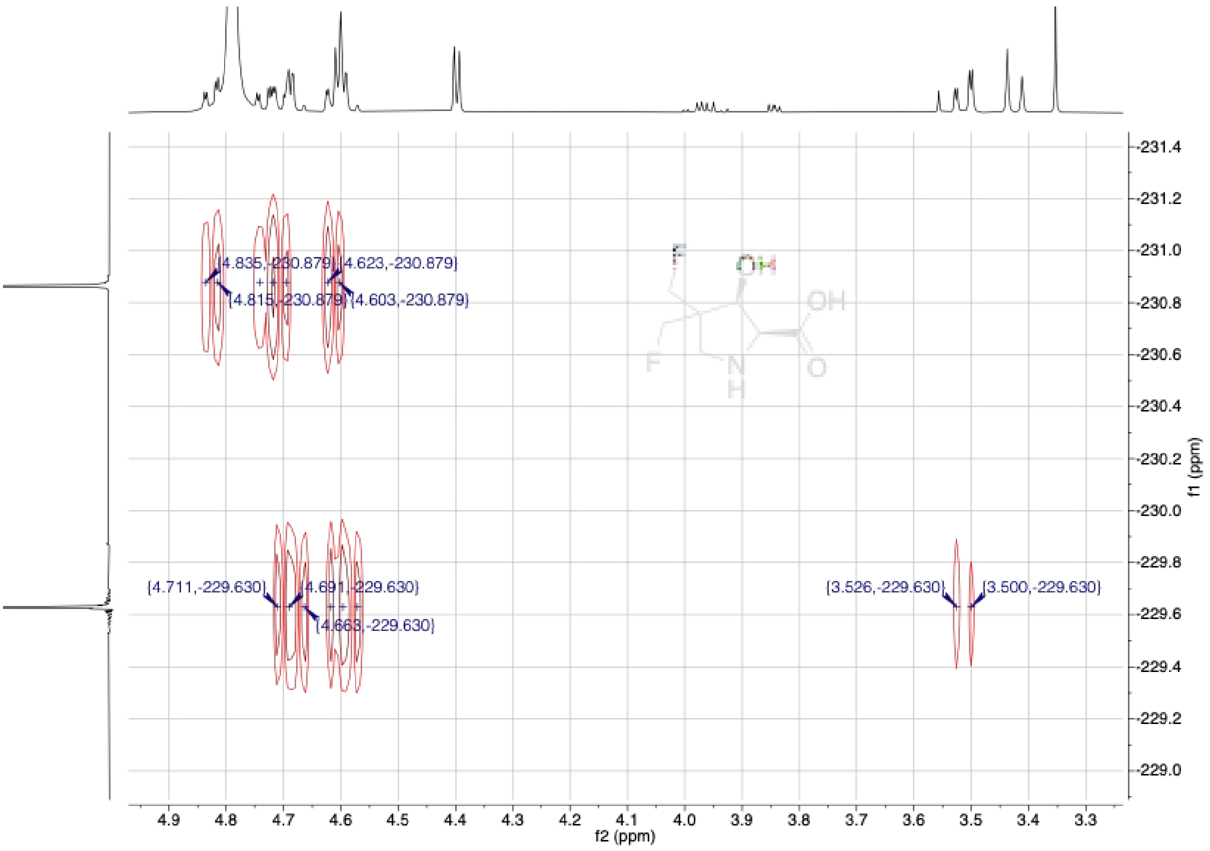

$^{19}F\{^1H\}$ NMR (470 MHz, D_2O)

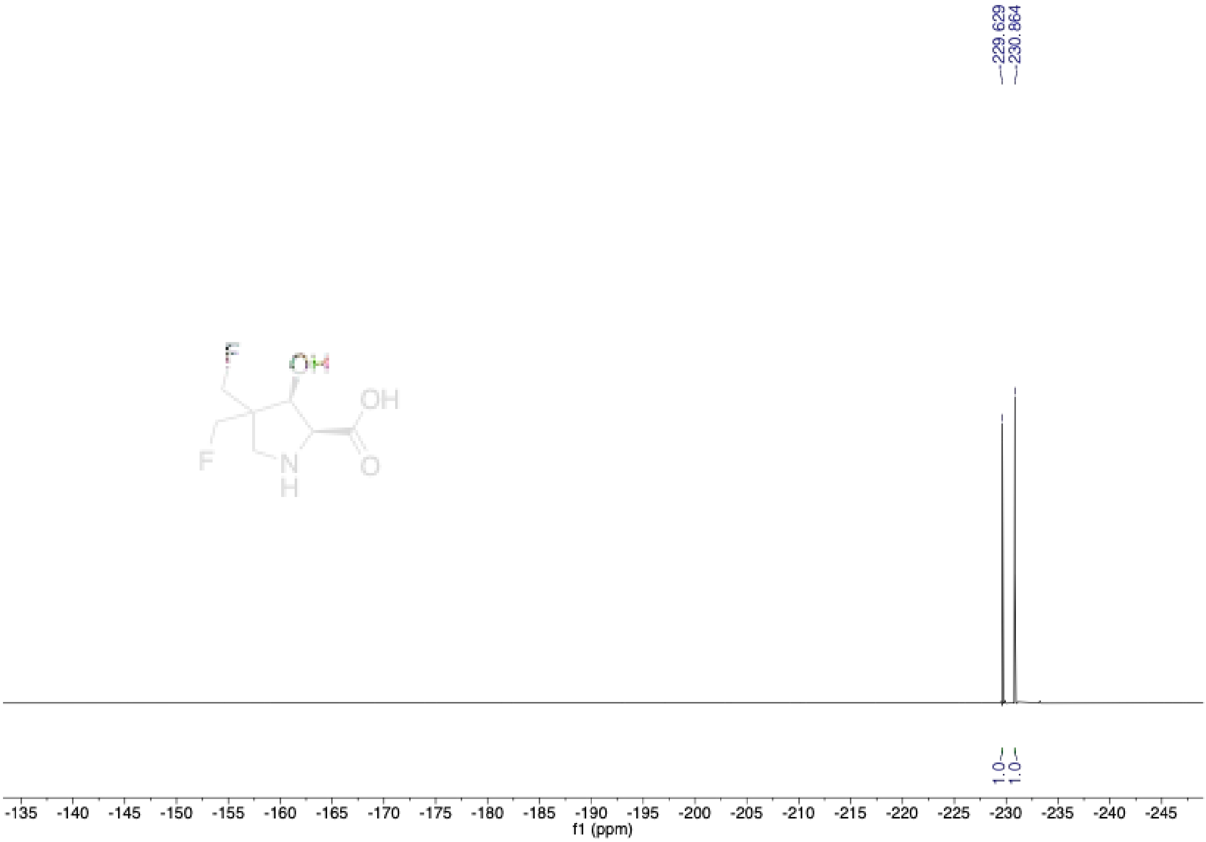

$^{13}C\{^1H\}$ NMR (176 MHz, D_2O)

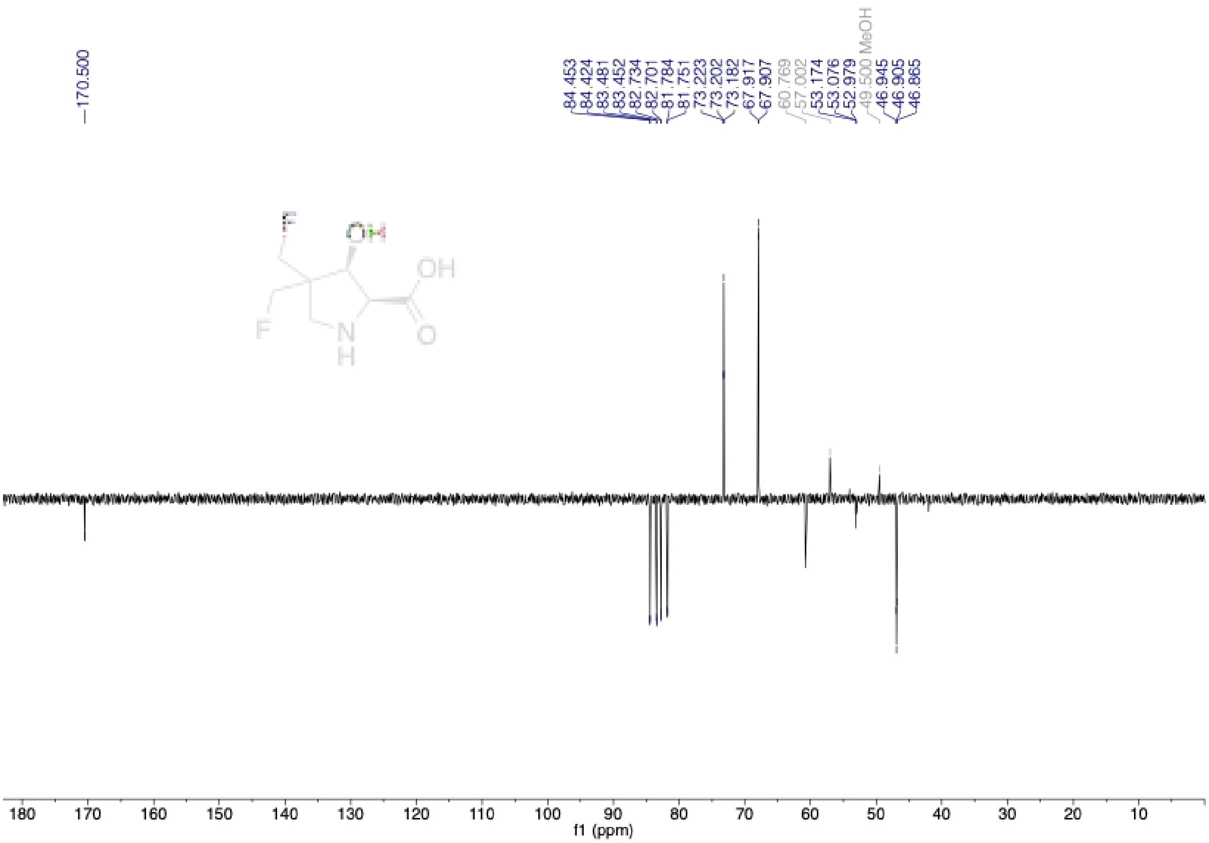

PA-3

^{1}H NMR (500 MHz, $CDCl_3$)

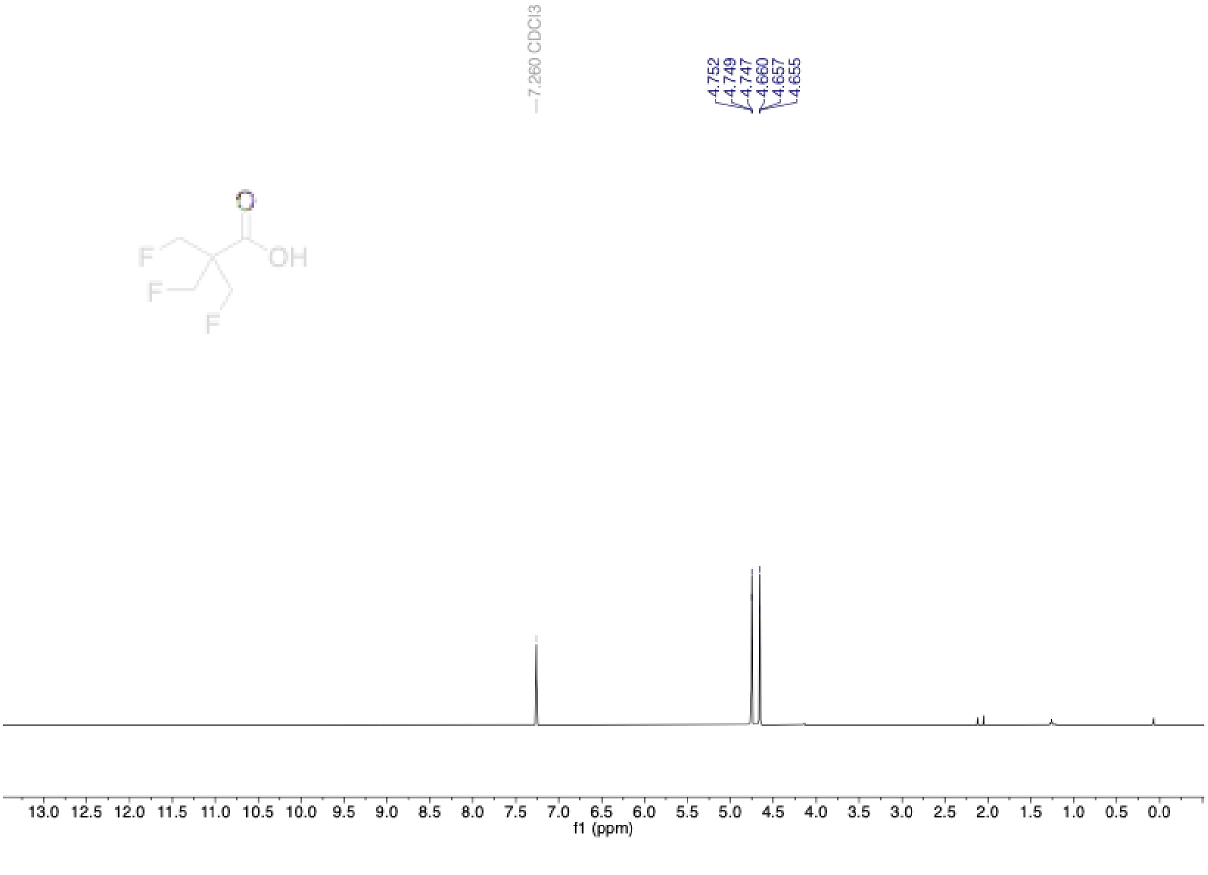

$^{19}F\{^{1}H\}$ NMR (470 MHz, $CDCl_3$)

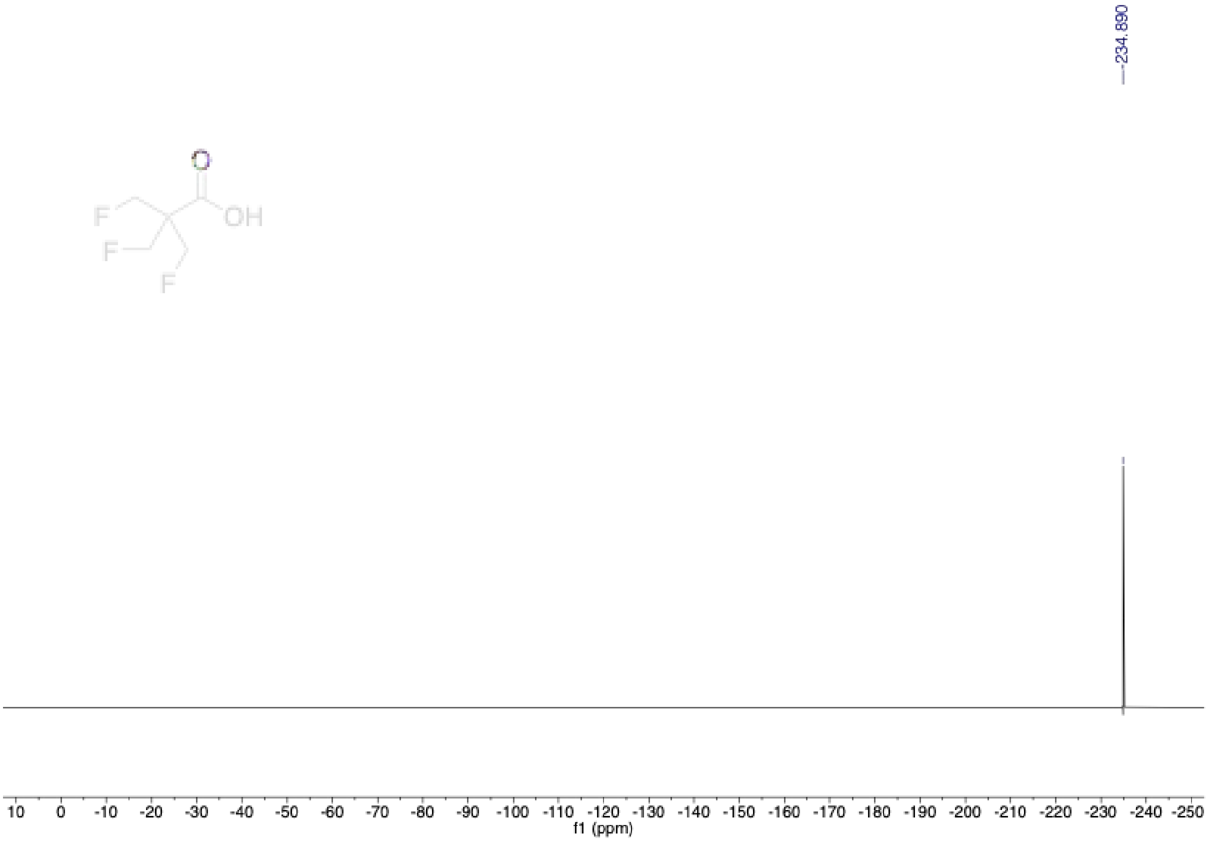

$^{13}C\{^{1}H\}$ NMR (126 MHz, $CDCl_3$)

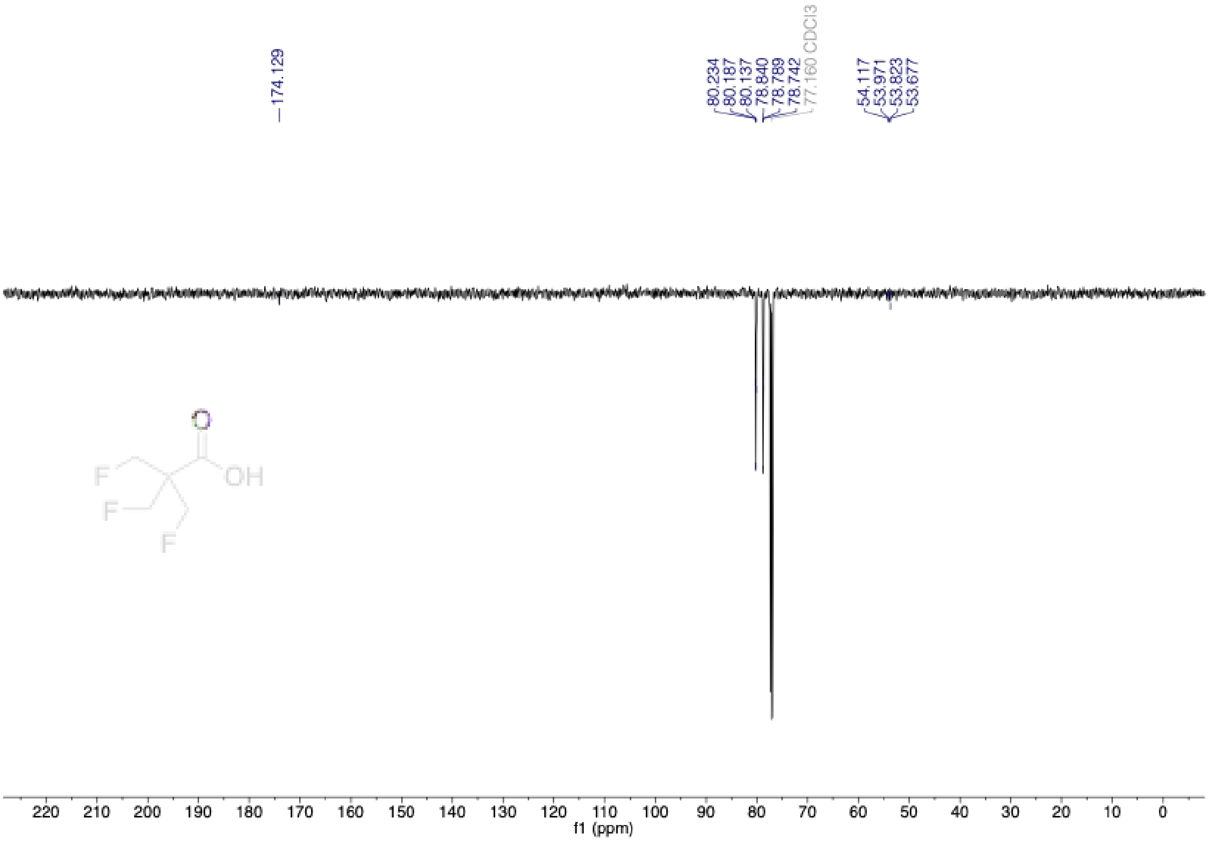

PE-3

^{1}H NMR (500 MHz, $CDCl_3$)

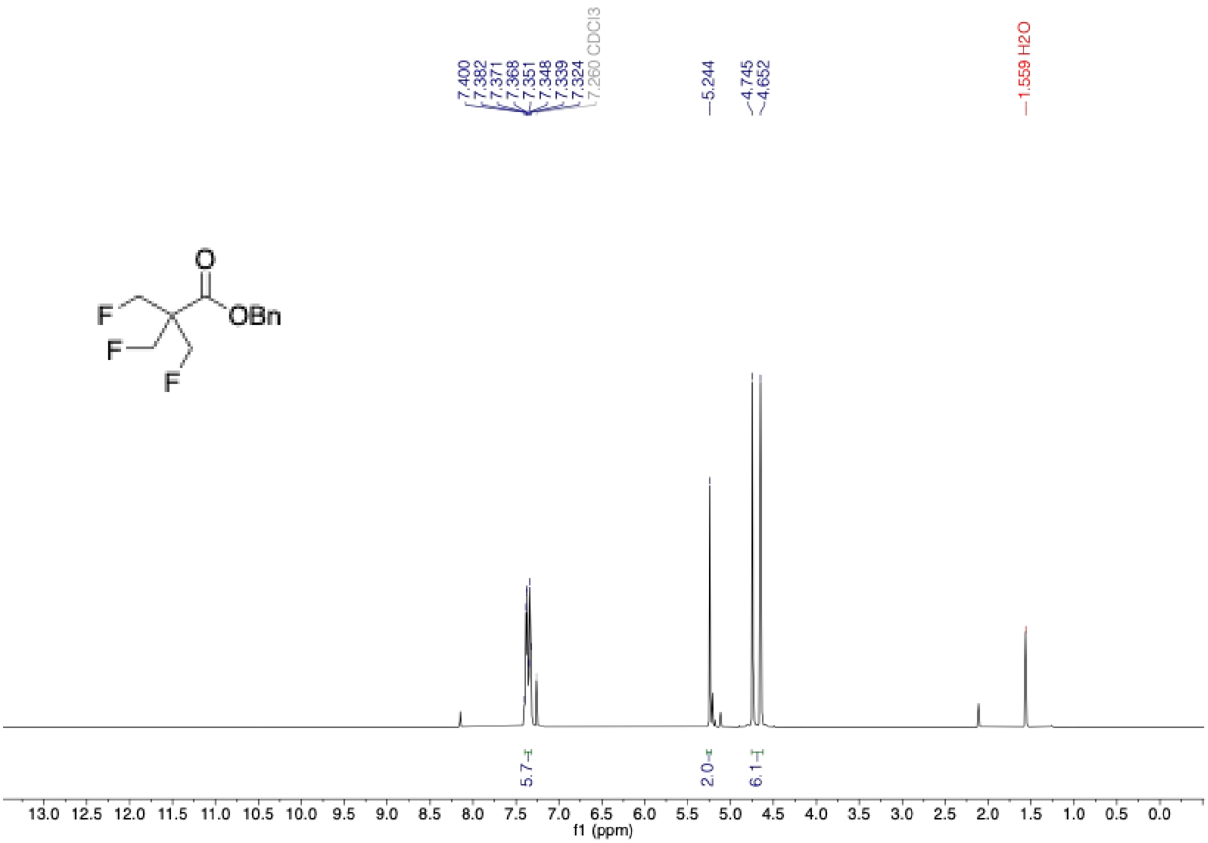

$^{19}F\{^1H\}$ NMR (470 MHz, $CDCl_3$)

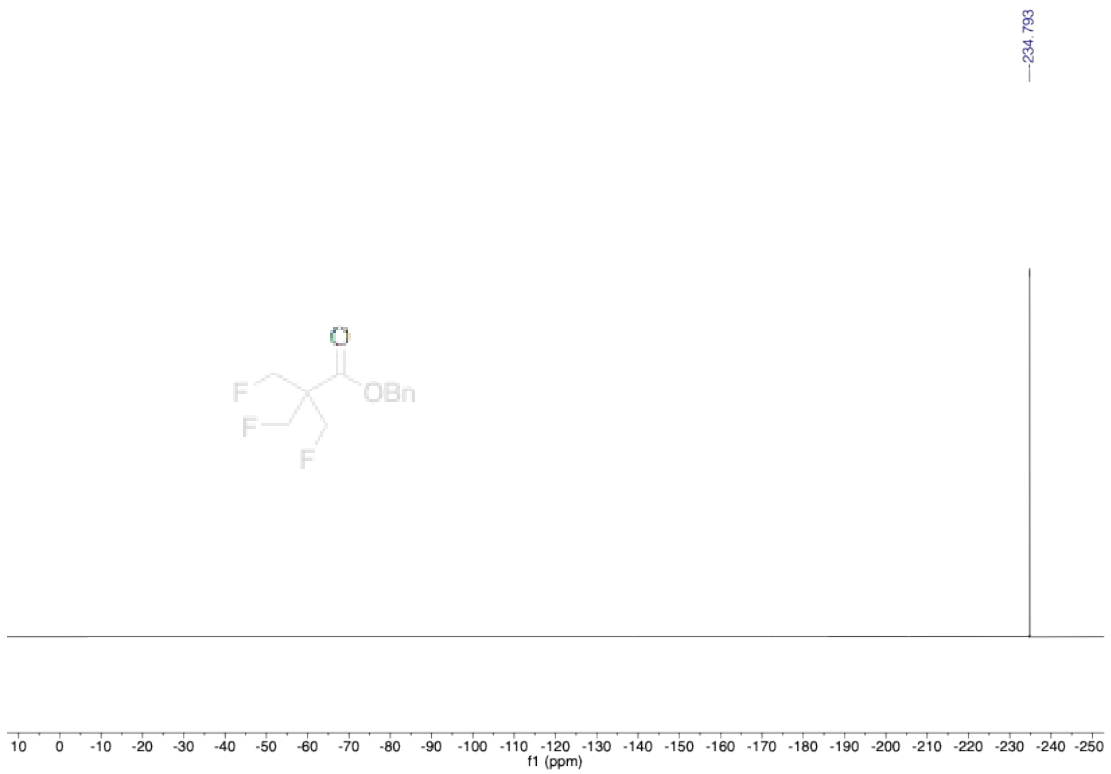

$^{13}C\{^1H\}$ NMR (101 MHz, CDCl3)

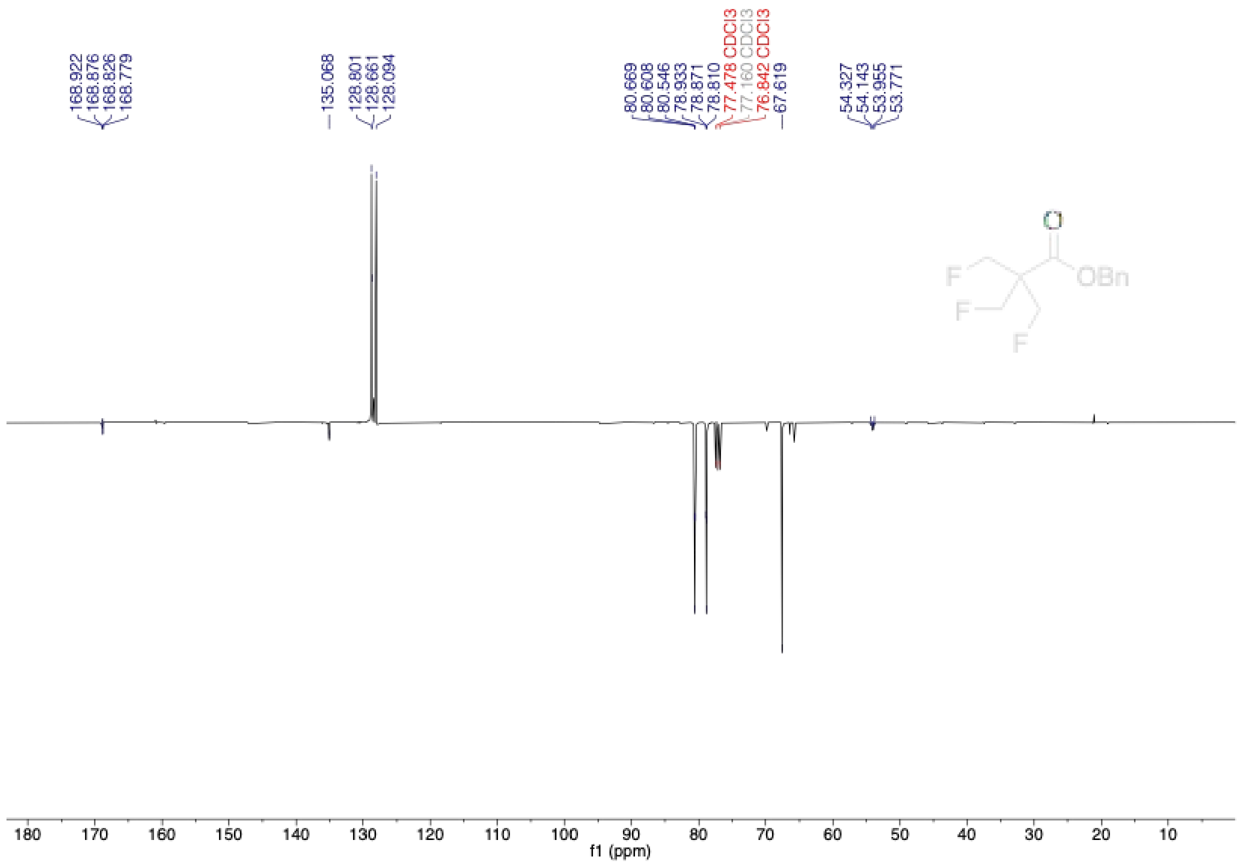

S6

^{1}H NMR (500 MHz, $CDCl_3$)

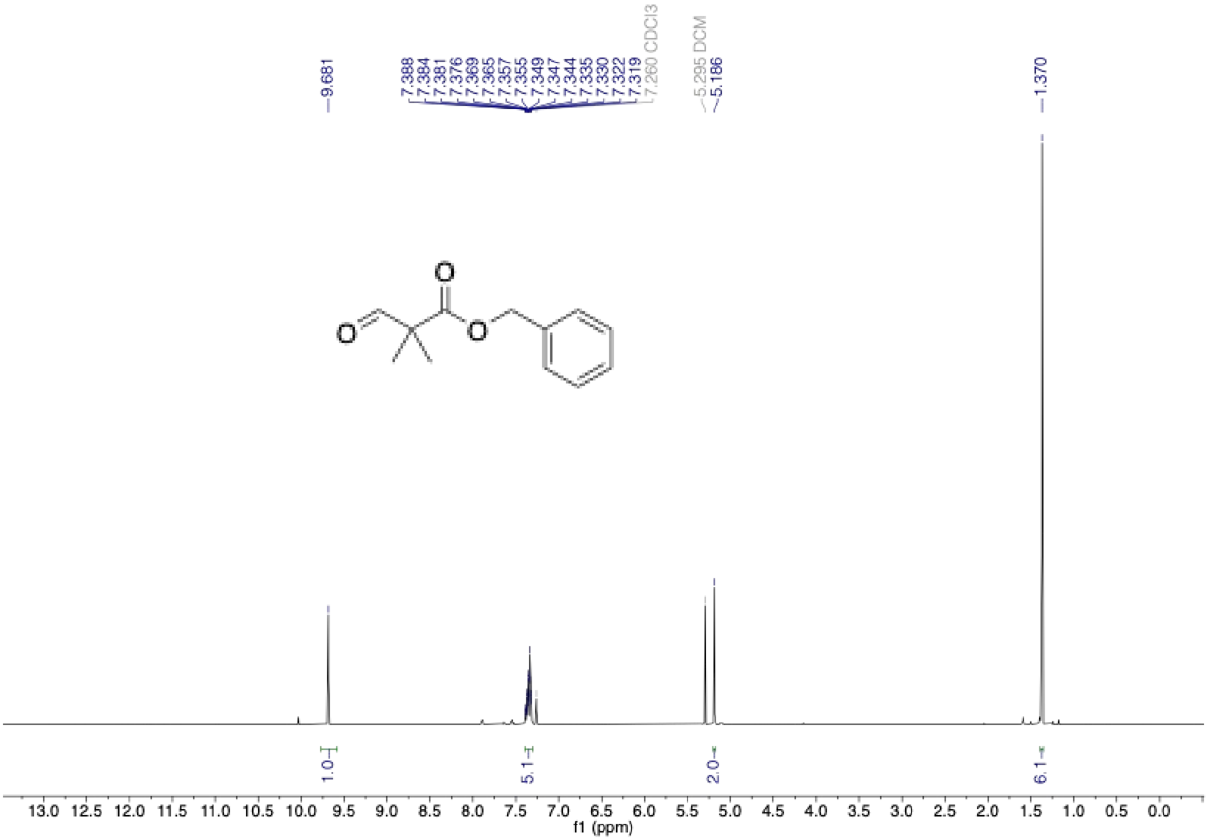

PE-4

^1H NMR (400 MHz, $CDCl_3$)

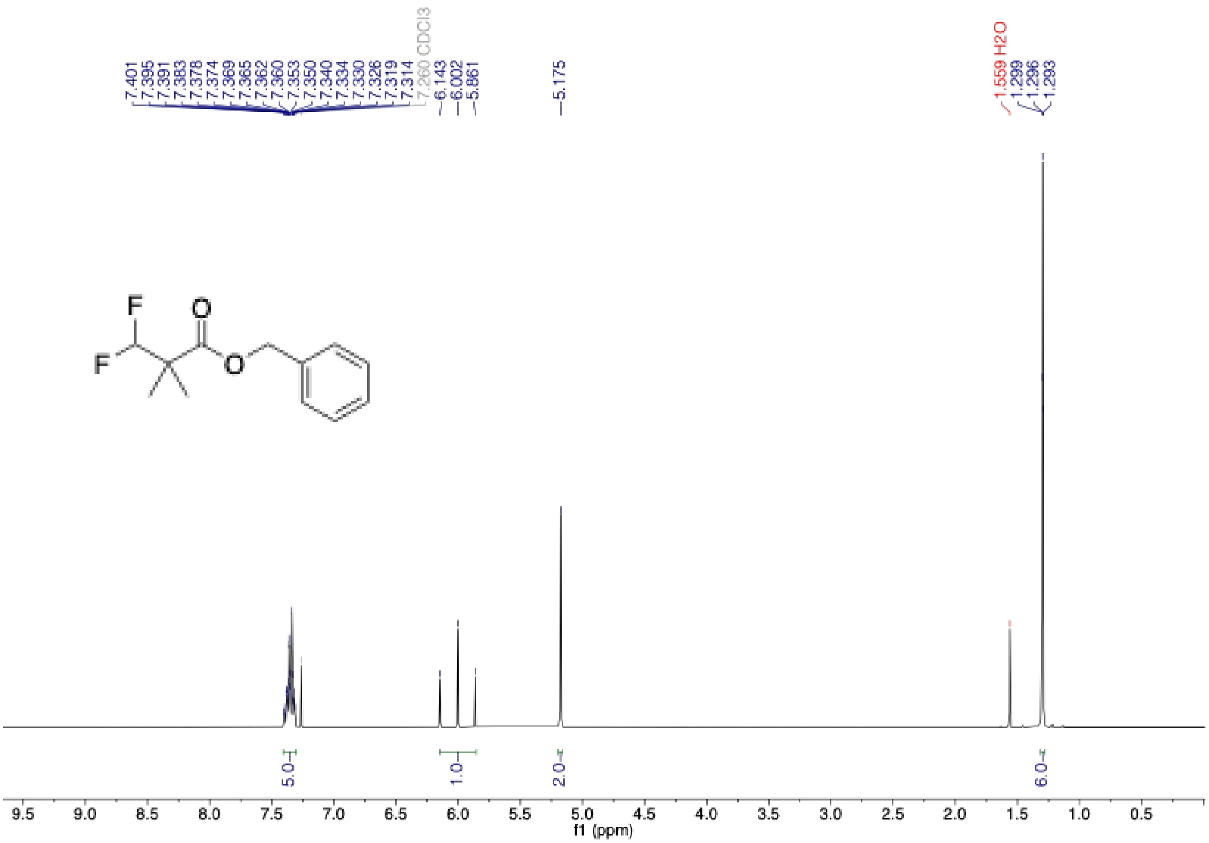

$^{19}F\{^1H\}$ NMR (377 MHz, $CDCl_3$)

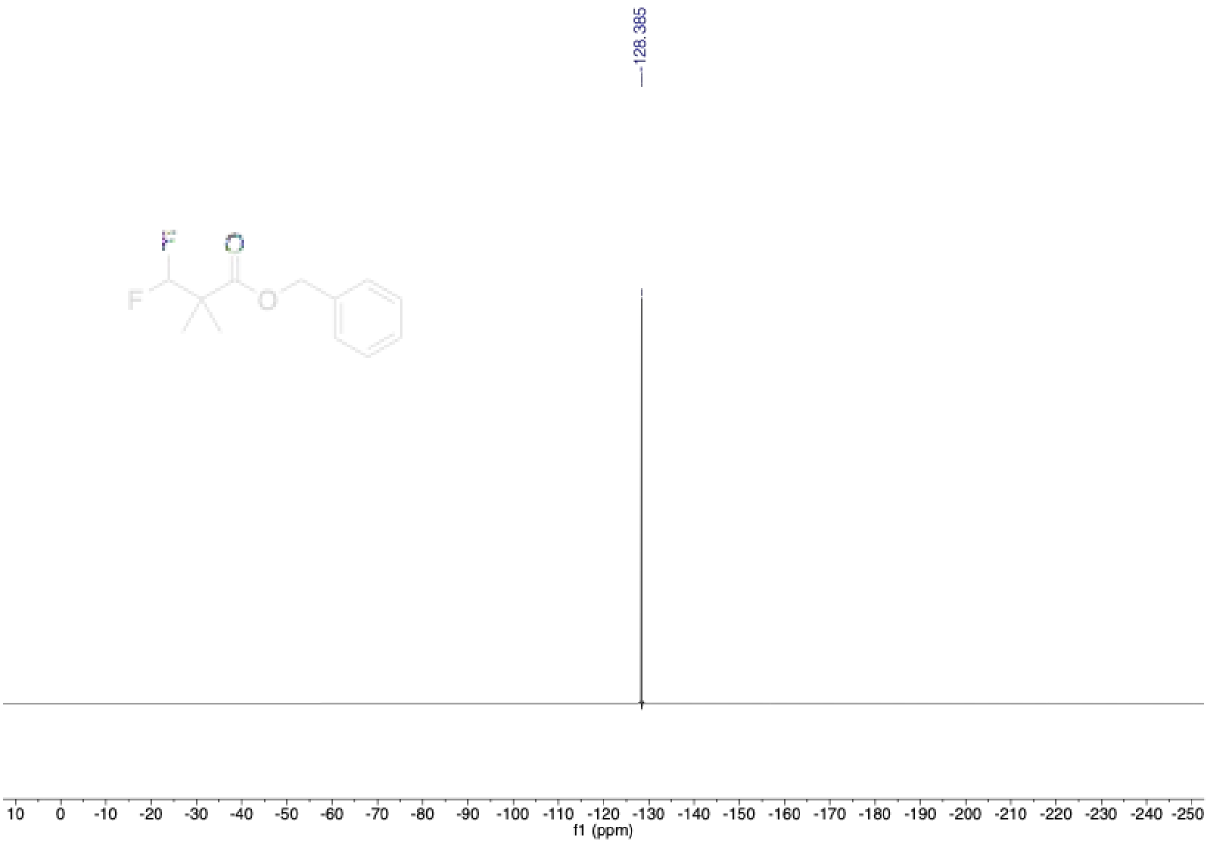

$^{13}C\{^{1}H\}$ NMR (126 MHz, $CDCl_3$)

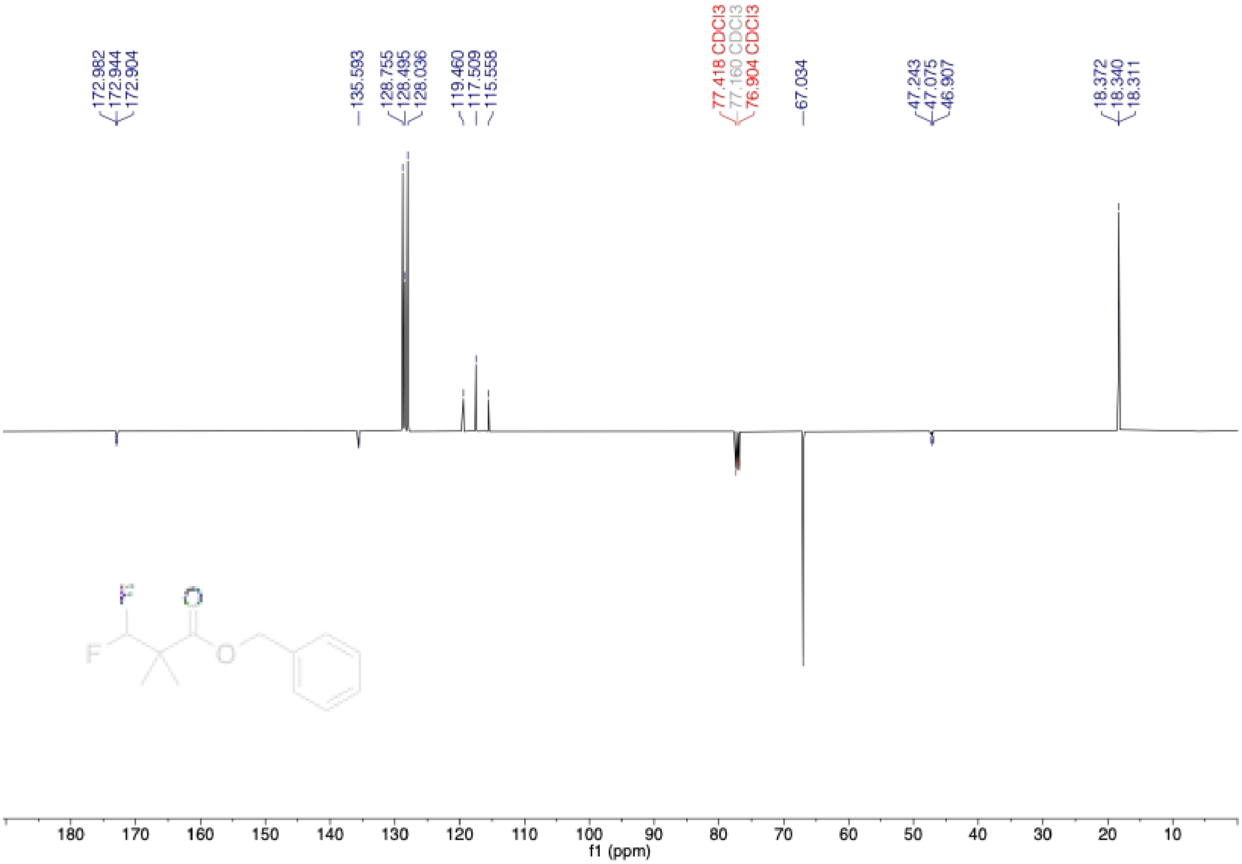

PA-4

^1H NMR (400 MHz, $CDCl_3$)

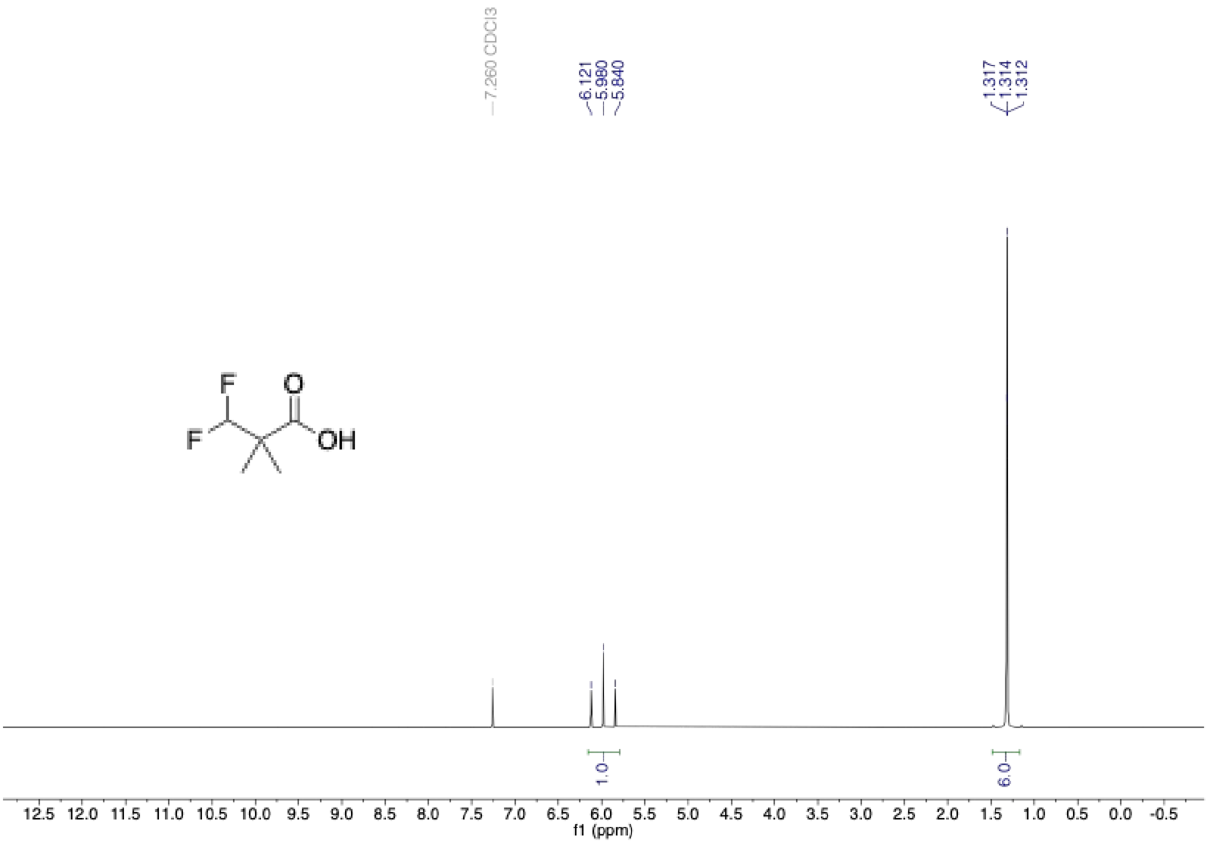

$^{19}F\{^1H\}$ NMR (377 MHz, $CDCl_3$)

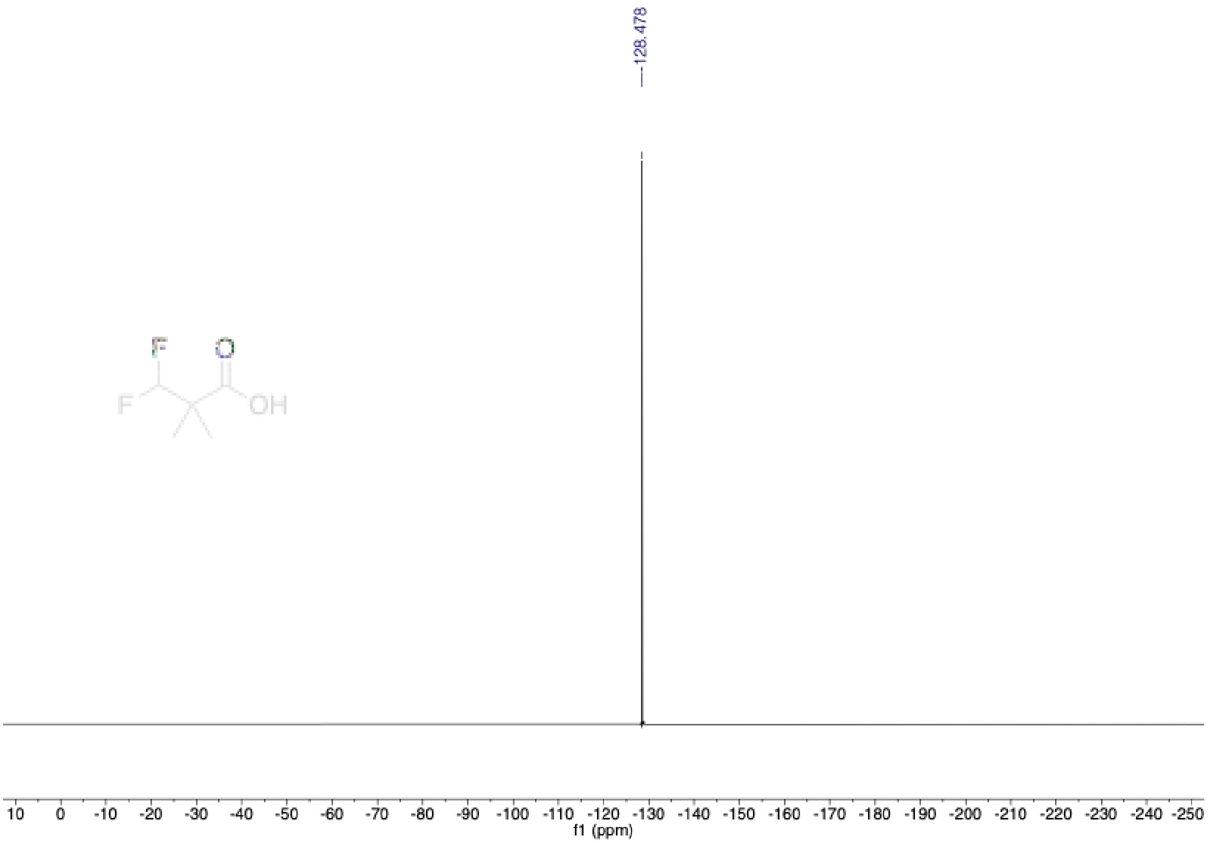

$^{13}C\{^{1}H\}$ NMR (101 MHz, $CDCl_3$)

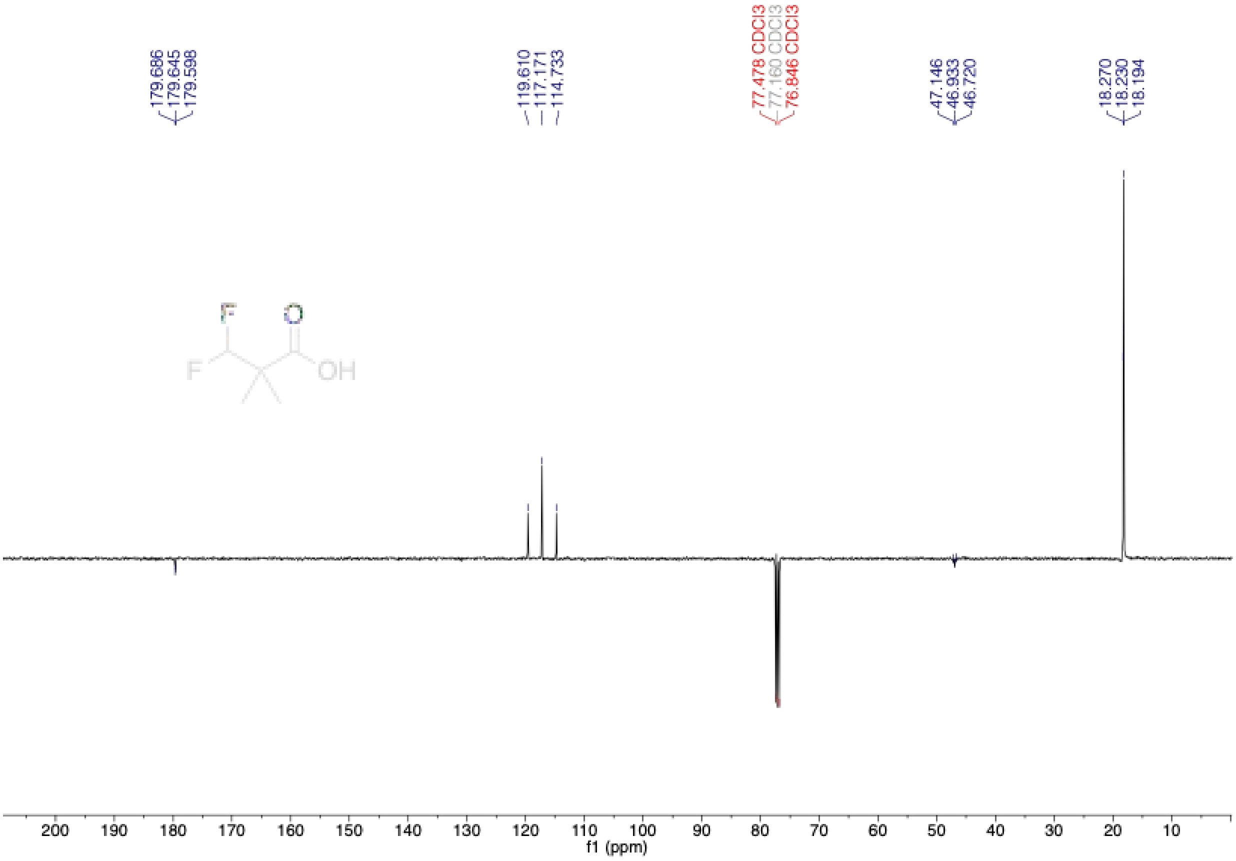

PE-5

^{1}H NMR (500 MHz, $CDCl_3$)

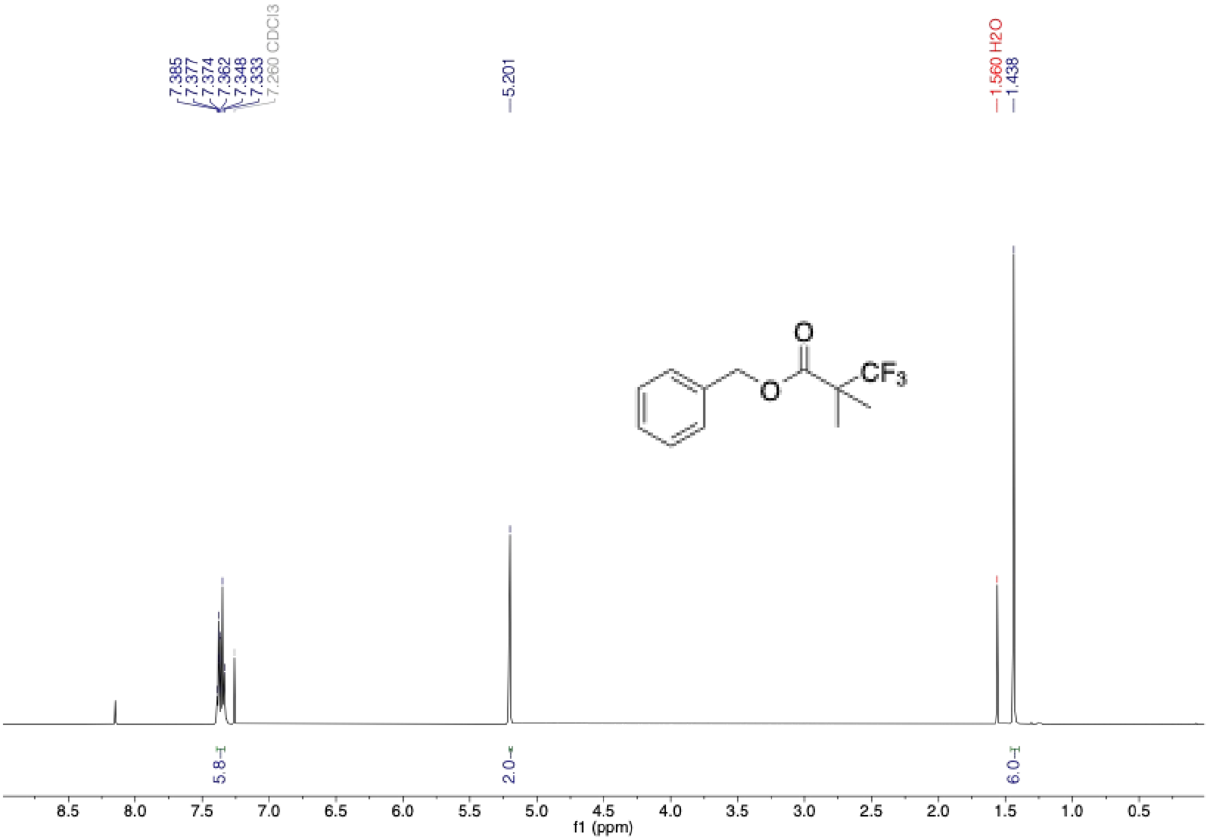

^{19}F{^{1}H} NMR (377 MHz, $CDCl_3$)

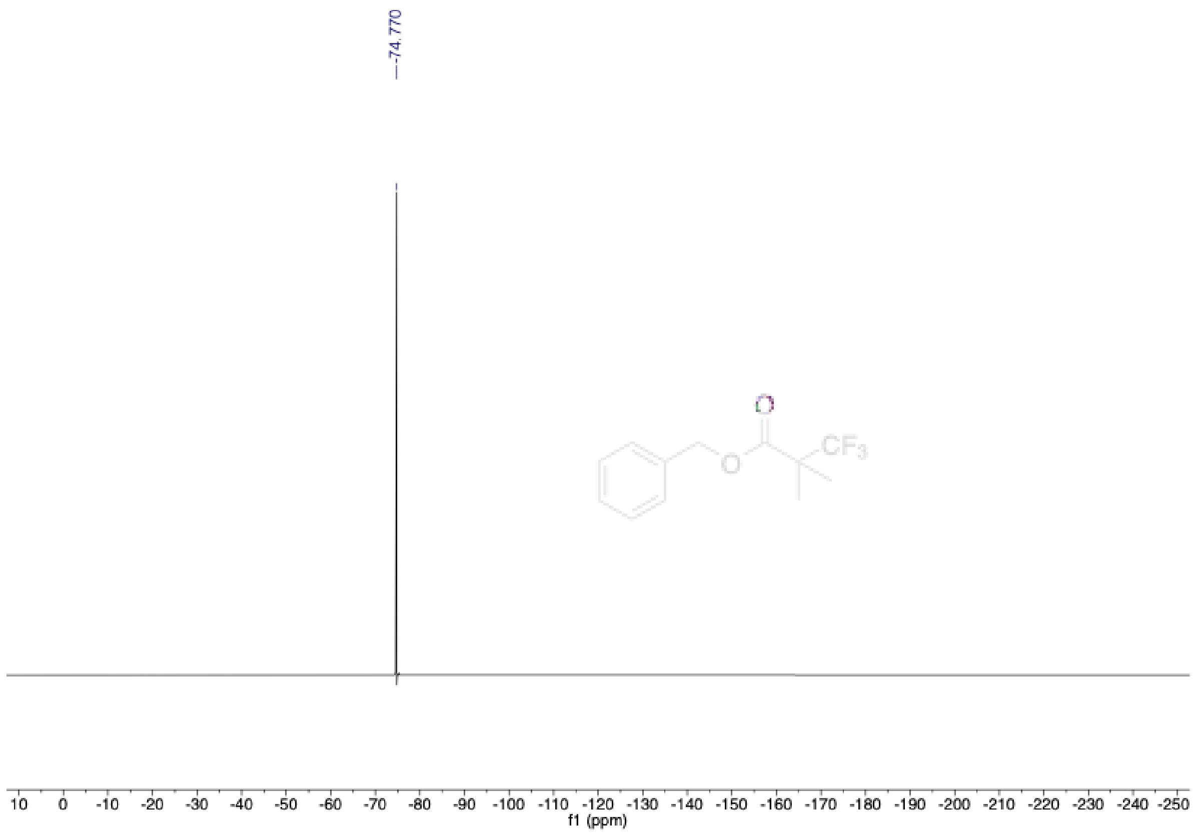

^{13}C{^{1}H} NMR (126 MHz, $CDCl_3$)

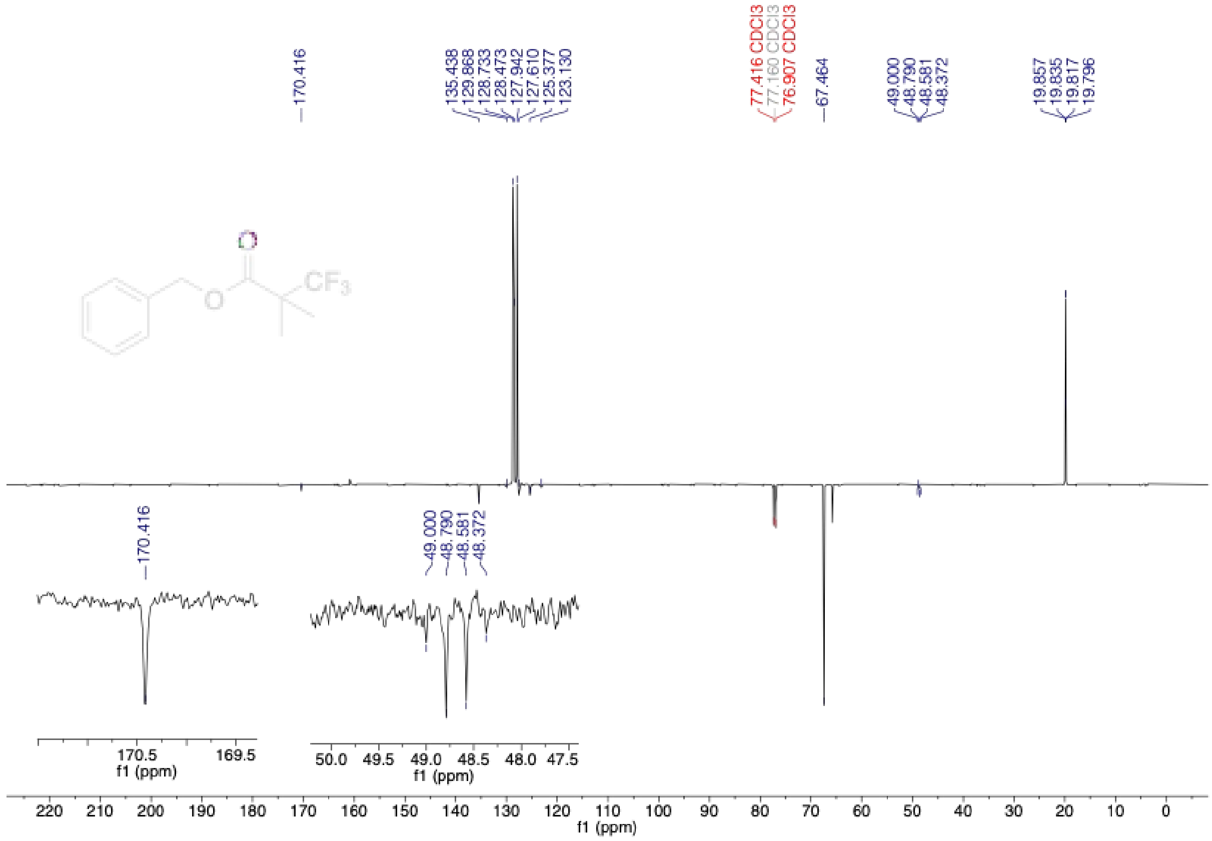

48

^{1}H NMR (500 MHz, DMSO)

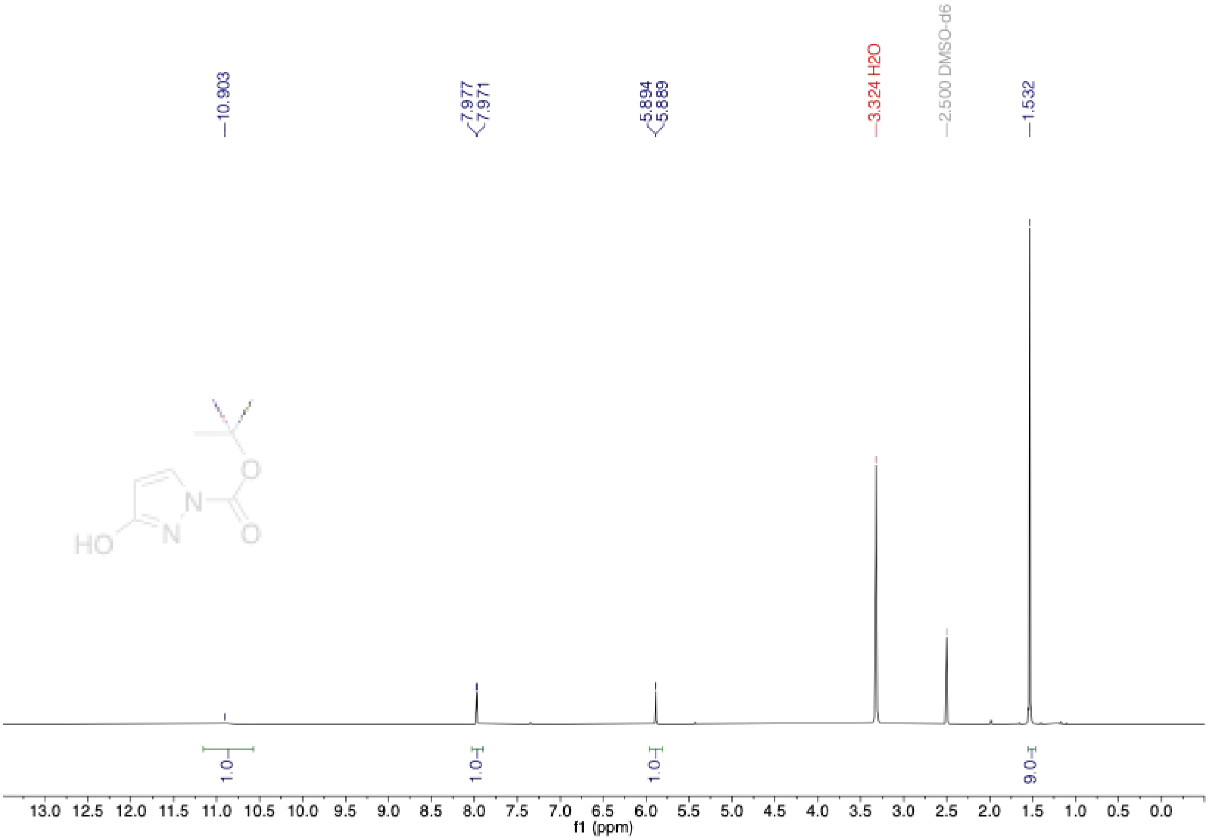

^{13}C{^{1}H} NMR (126 MHz, DMSO)

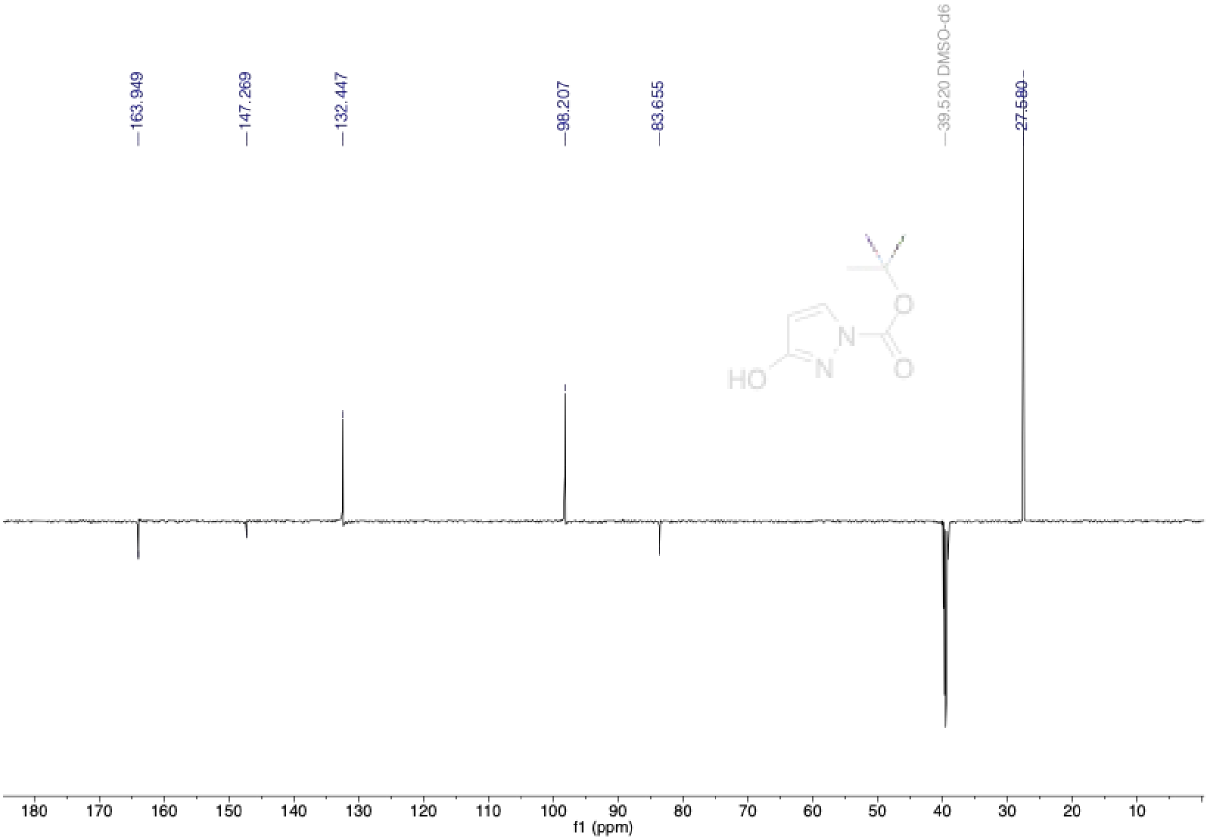

49

^{1}H NMR (400 MHz, $CDCl_3$)

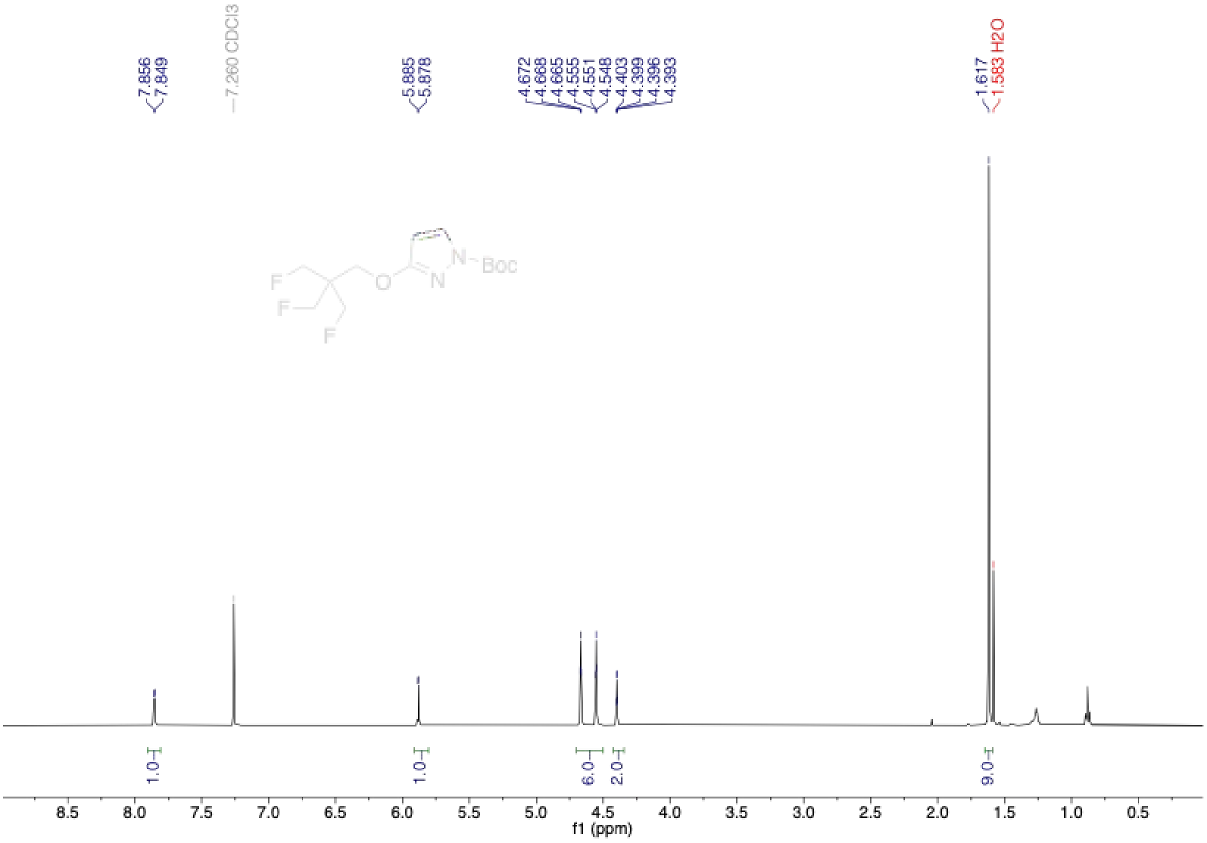

$^{19}F\{^1H\}$ NMR (377 MHz, $CDCl_3$)

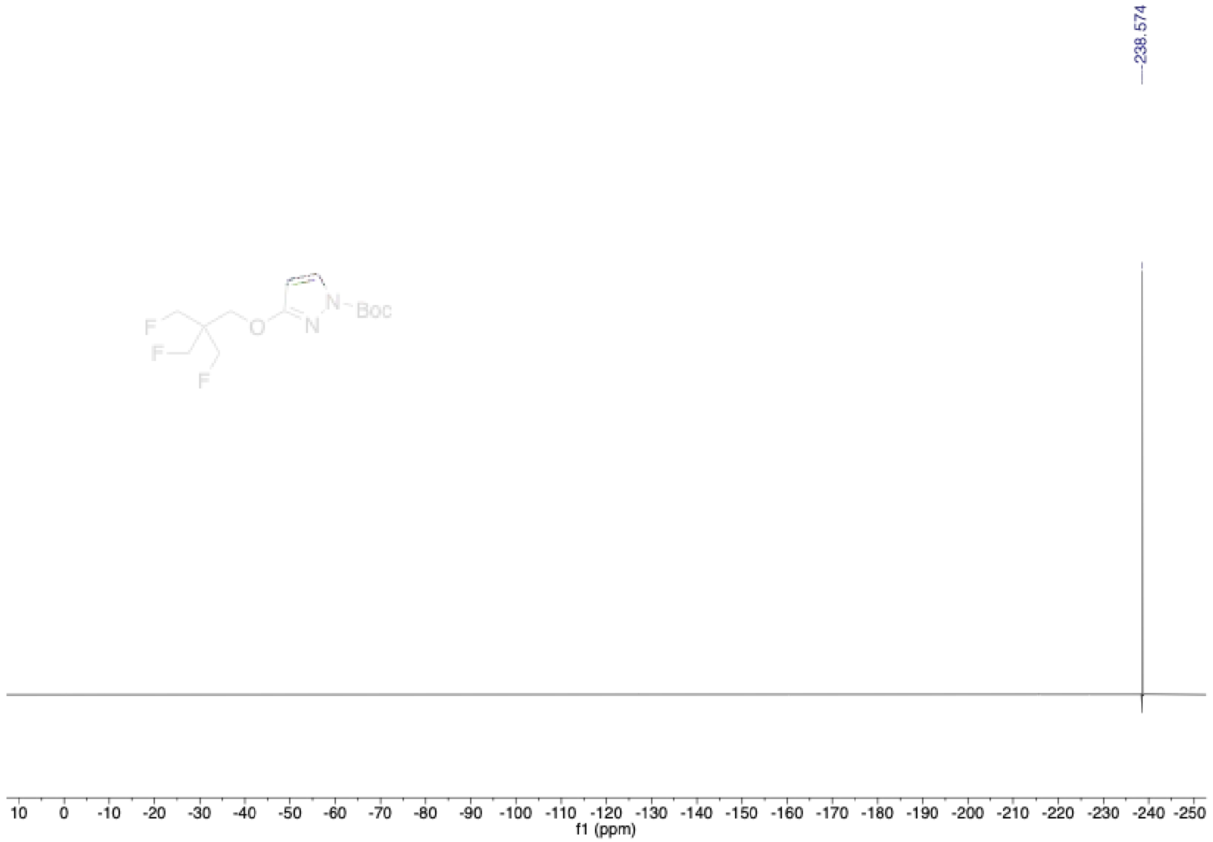

$^{13}C\{^1H\}$ NMR (101 MHz, $CDCl_3$)

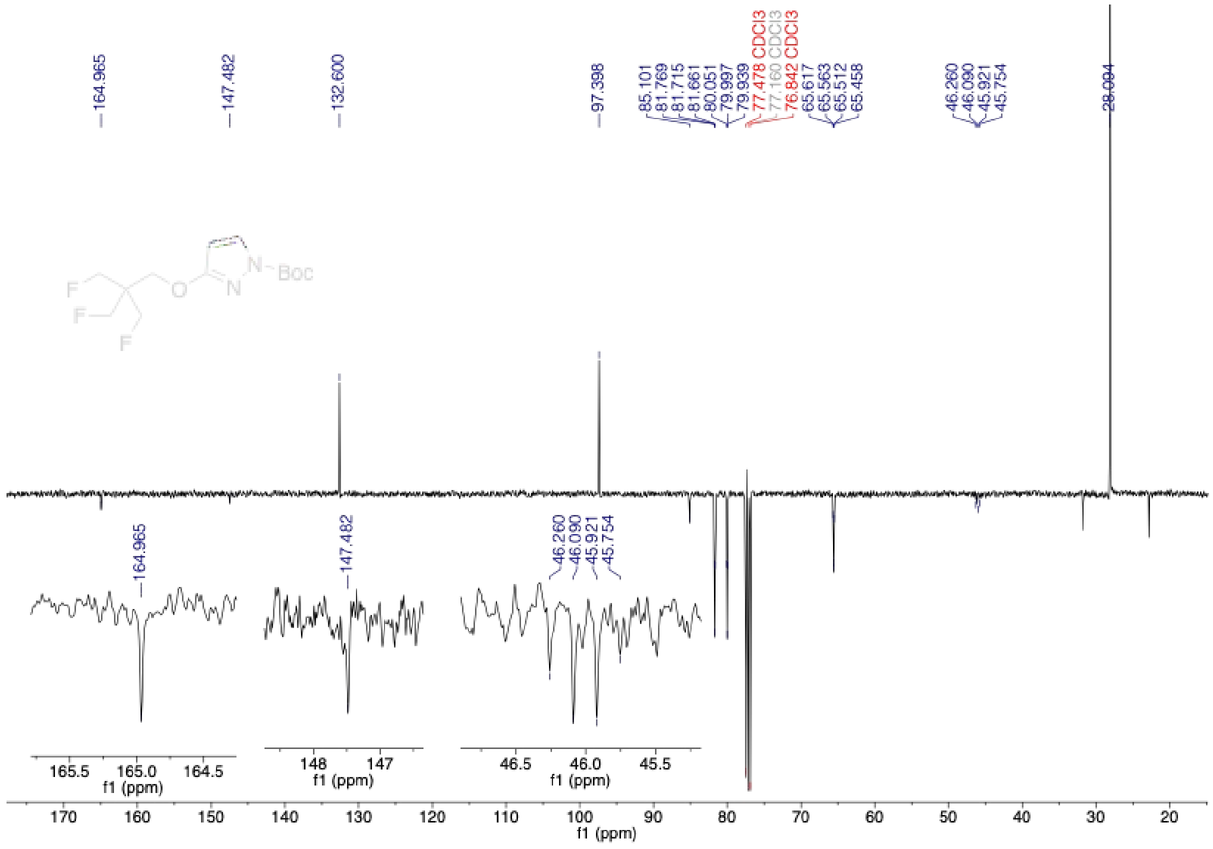

50

^{1}H NMR (400 MHz, $CDCl_3$)

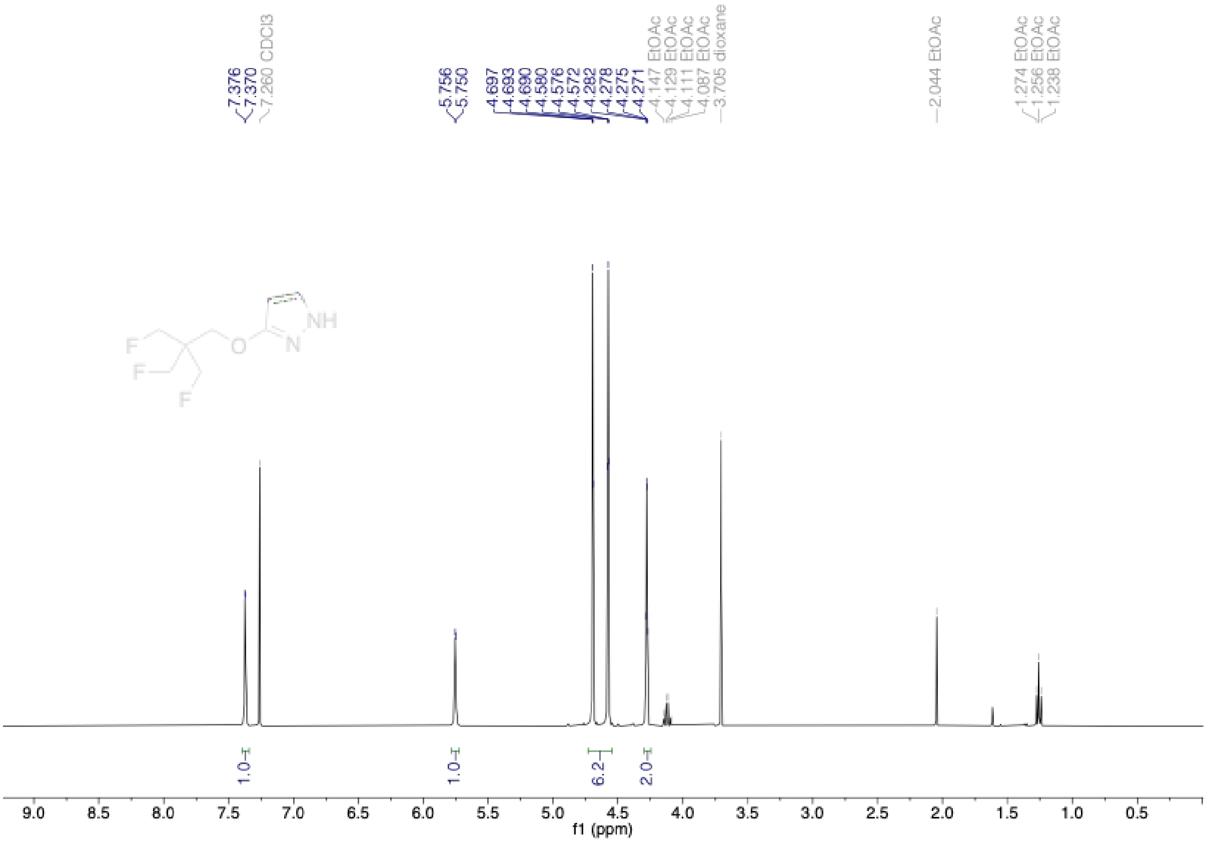

$^{19}F\{^{1}H\}$ NMR (377 MHz, $CDCl_3$)

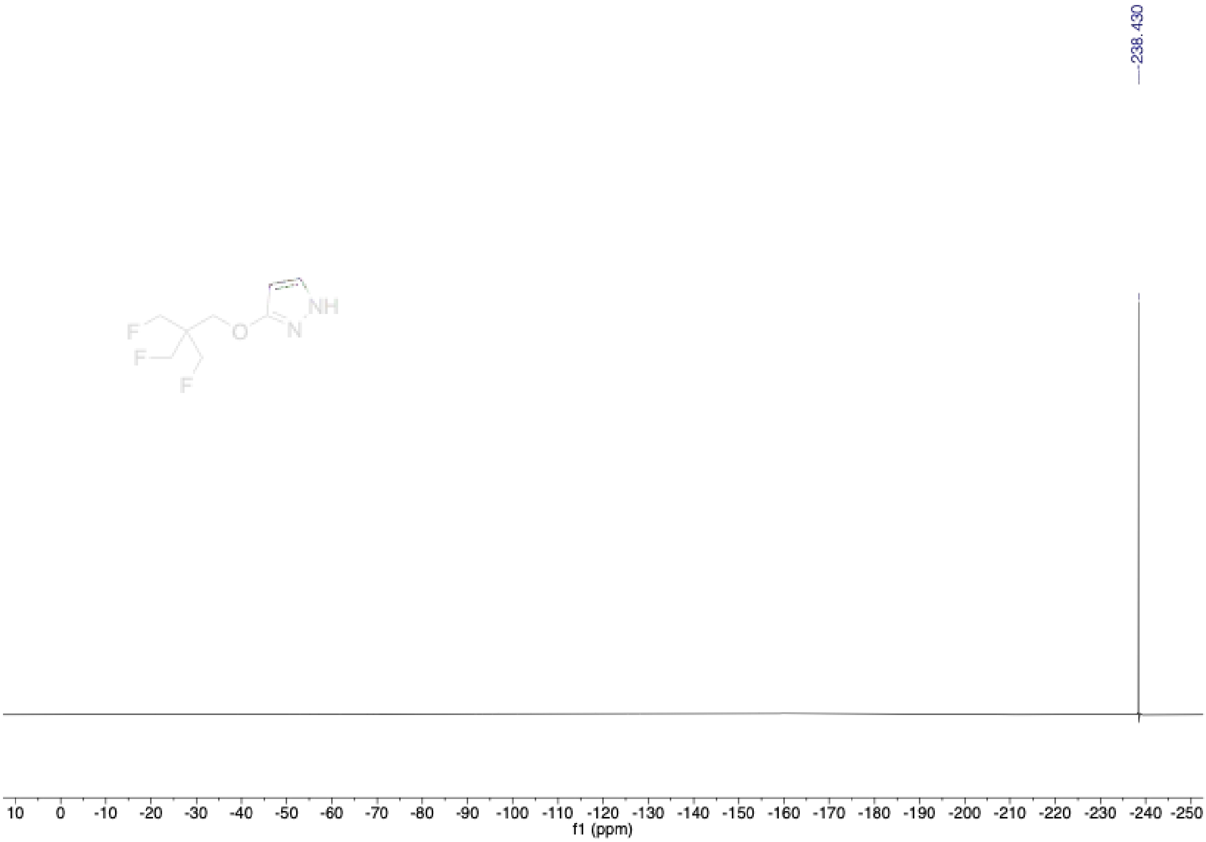

$^{13}C\{^{1}H\}$ NMR (126 MHz, $CDCl_3$)

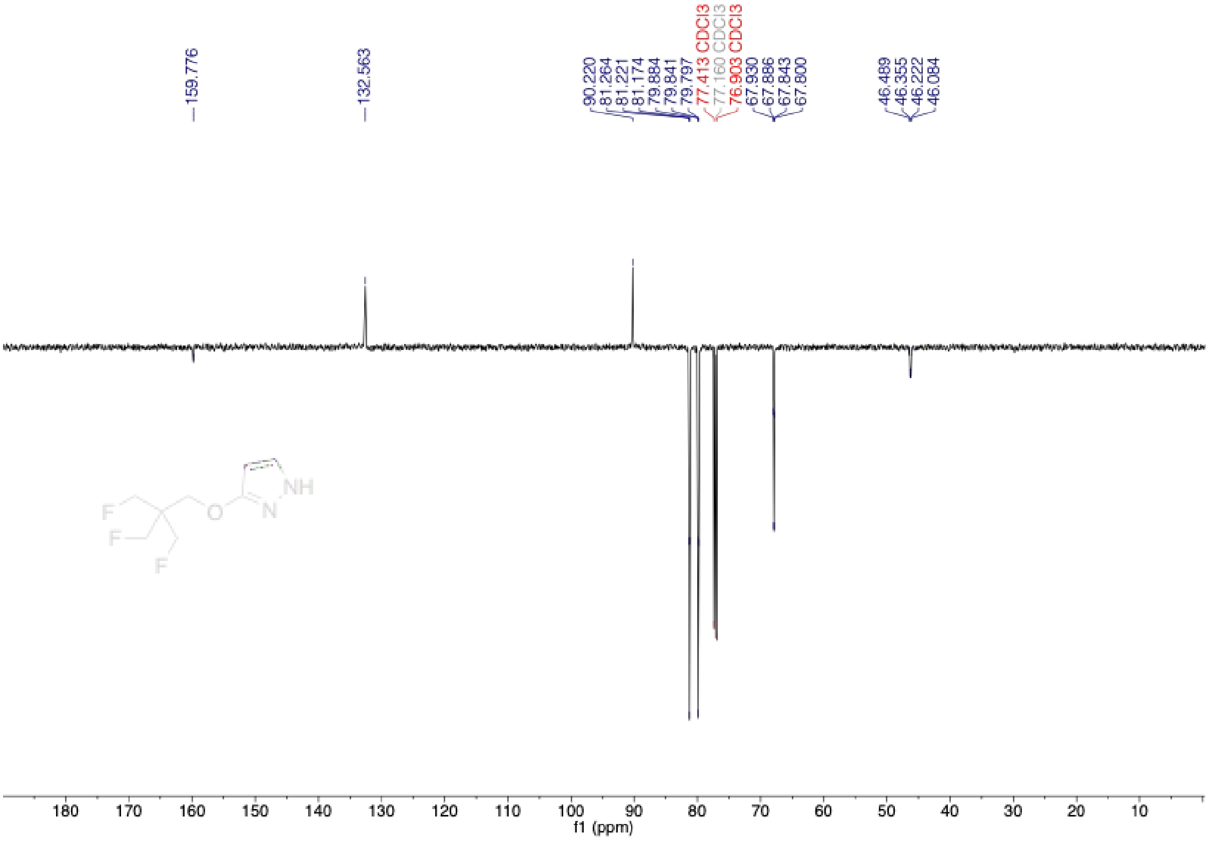

52

^{1}H NMR (400 MHz, $CDCl_3$)

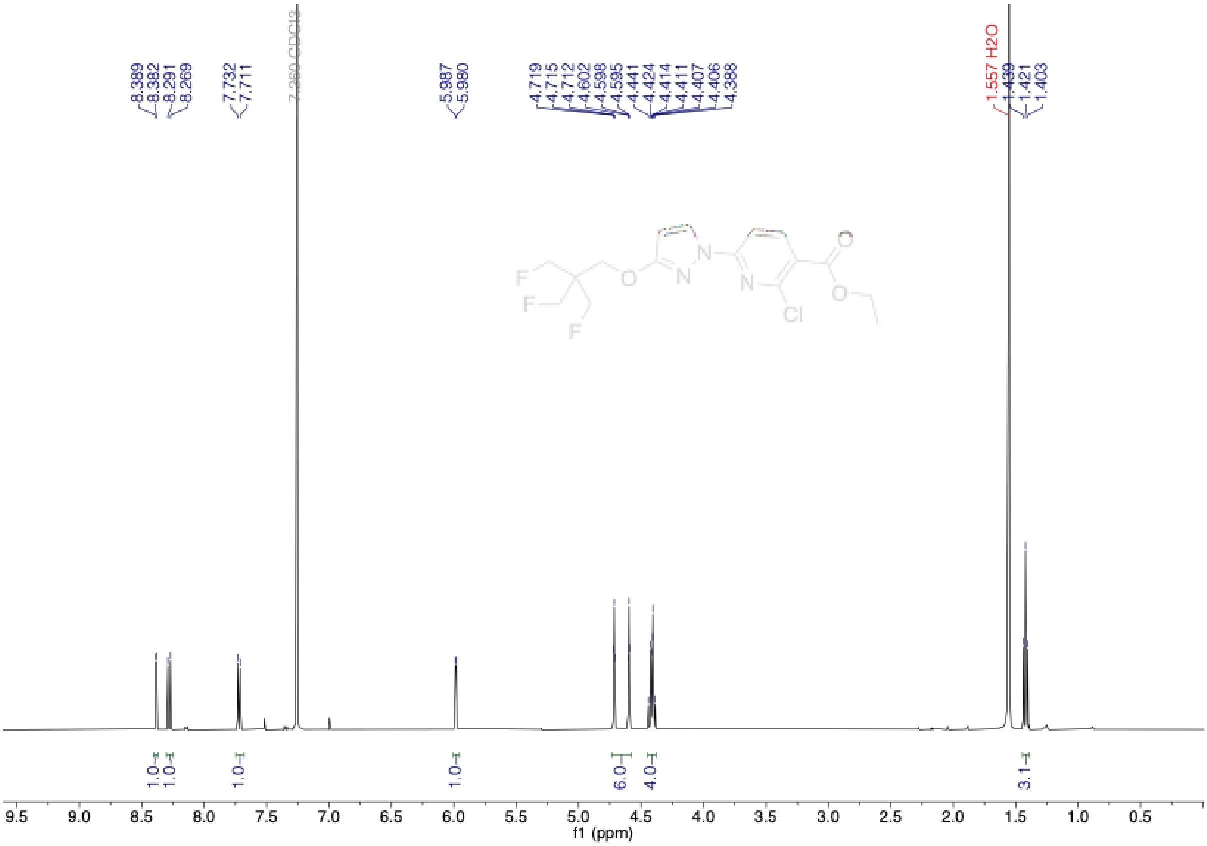

$^{19}F\{^{1}H\}$ NMR (377 MHz, $CDCl_3$)

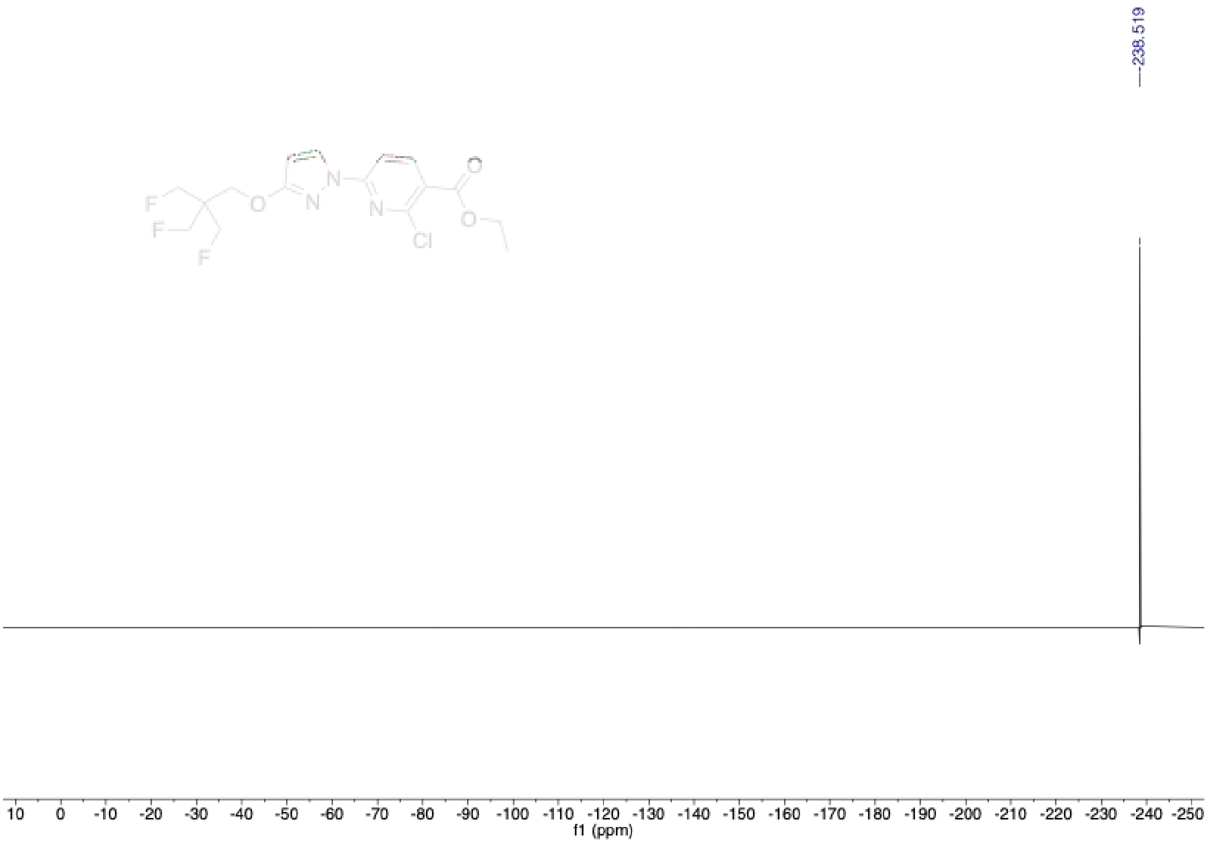

$^{13}C\{^{1}H\}$ NMR (126 MHz, $CDCl_3$)

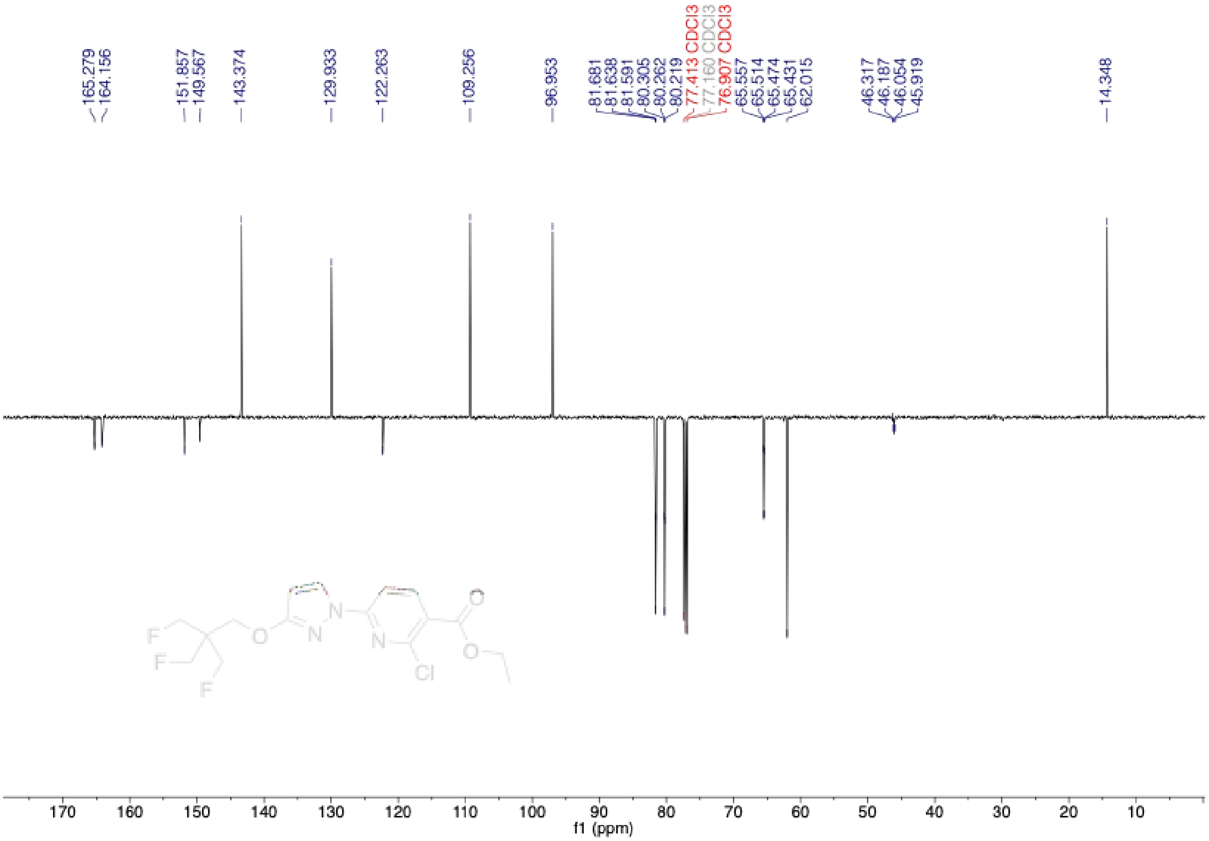

53

^{1}H NMR (500 MHz, DMSO)

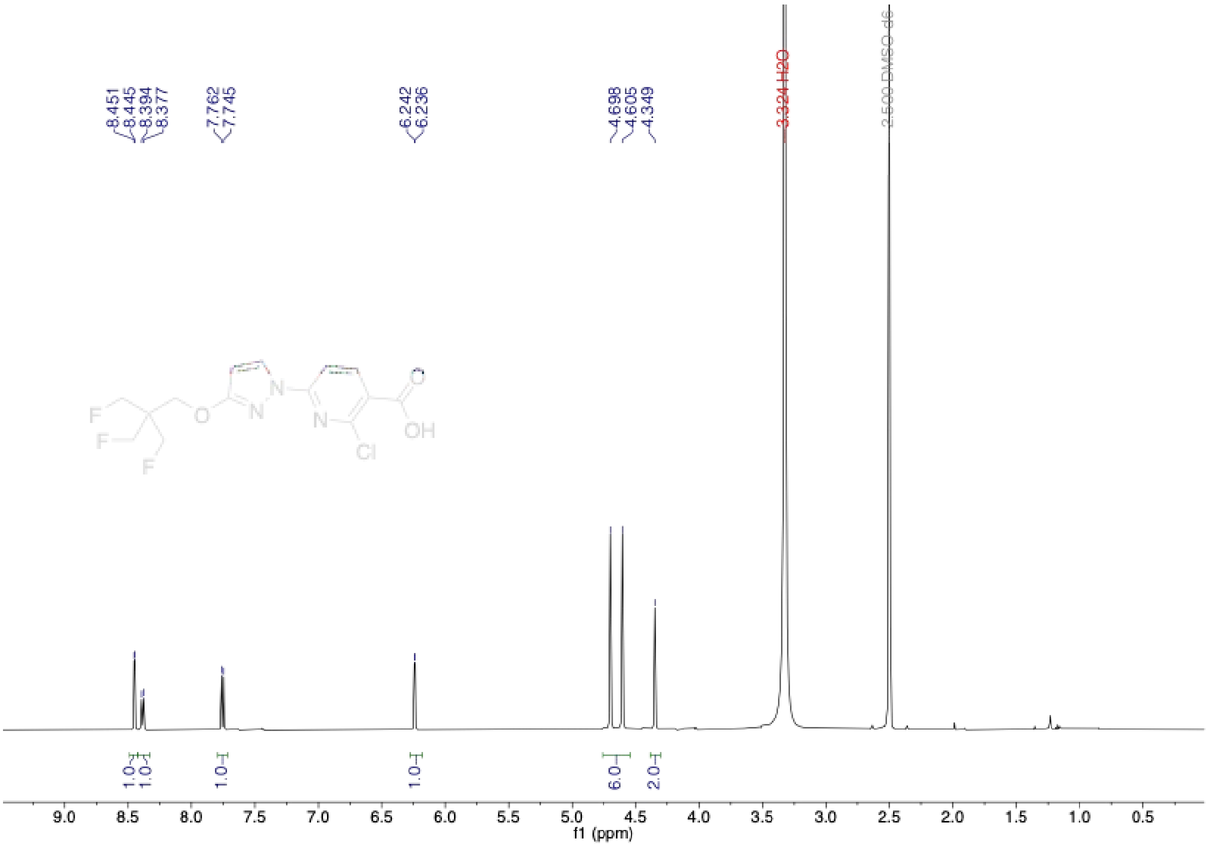

$^{19}F\{^{1}H\}$ NMR (470 MHz, DMSO)

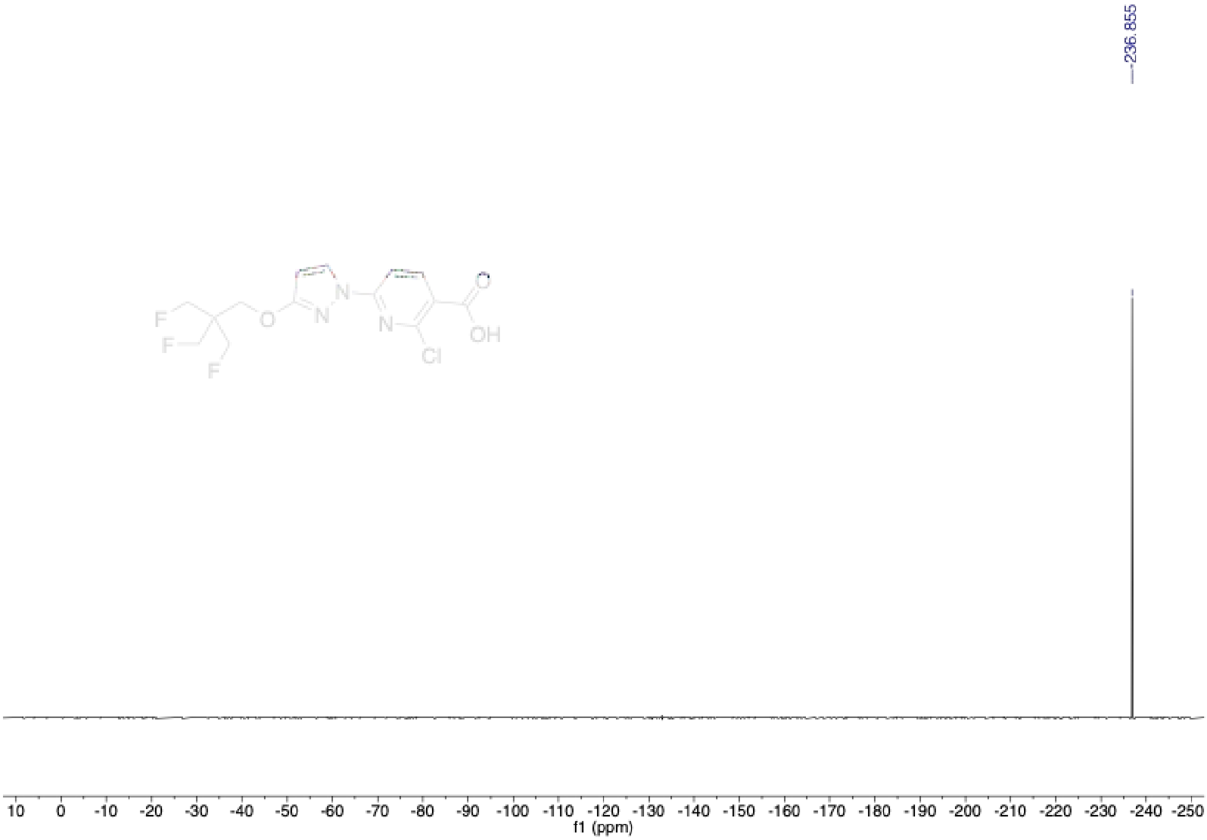

$^{13}C\{^{1}H\}$ NMR (126 MHz, DMSO)

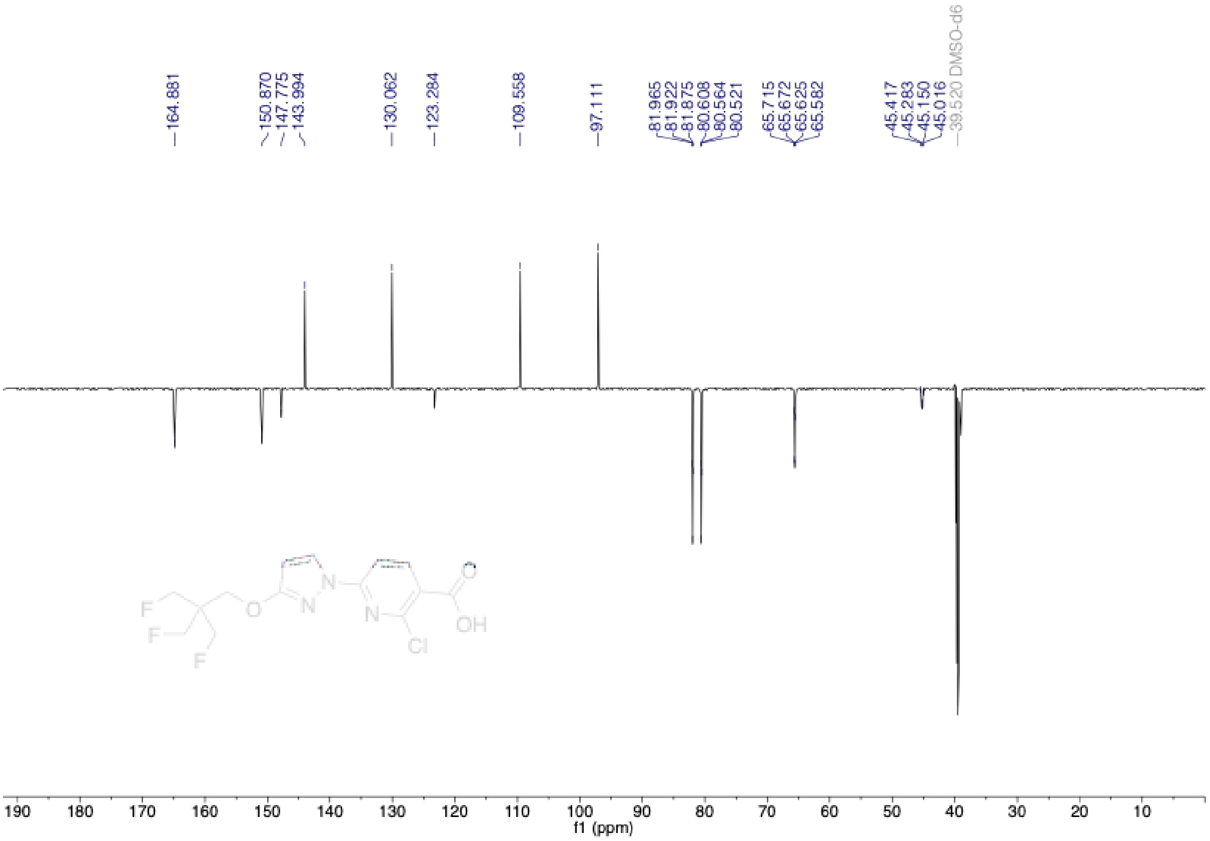

55

^{1}H NMR (500 MHz, $CDCl_3$)

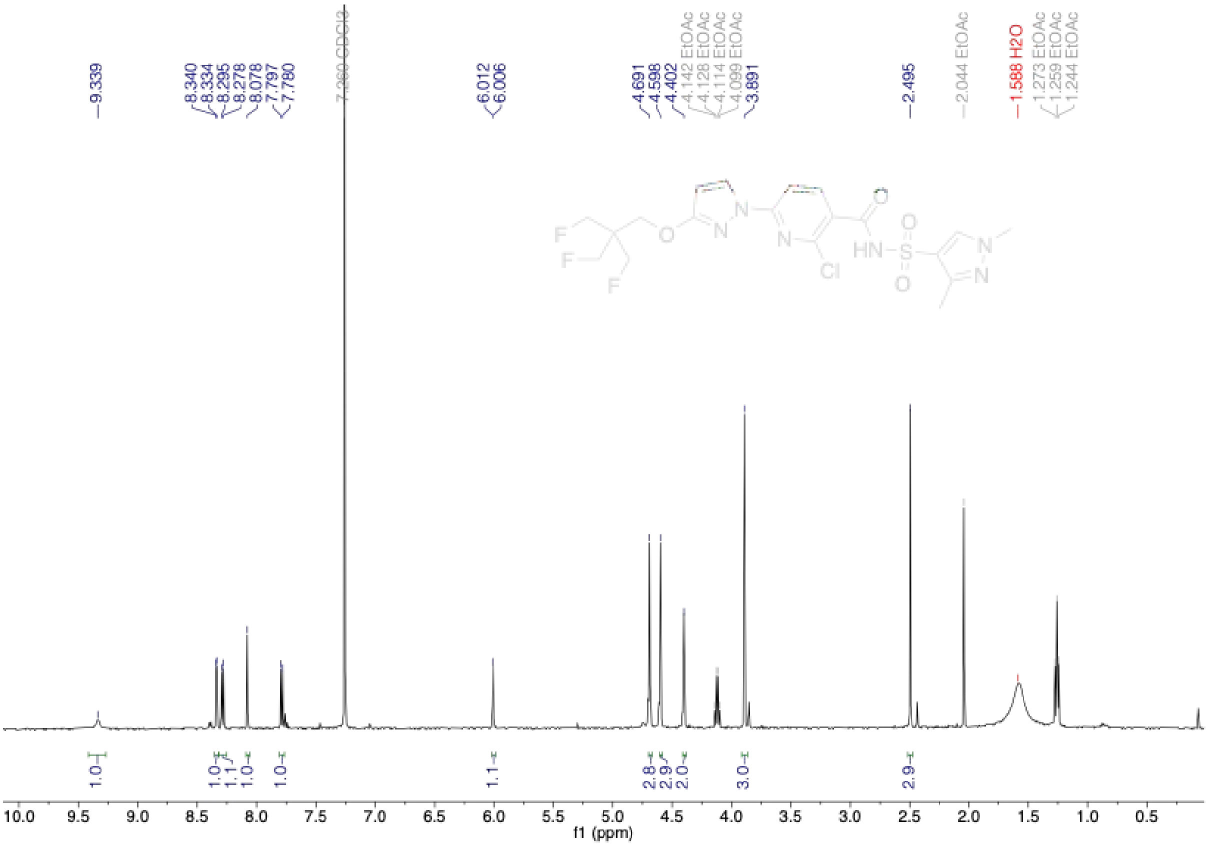

$^{19}F\{^{1}H\}$ NMR (470 MHz, $CDCl_3$)

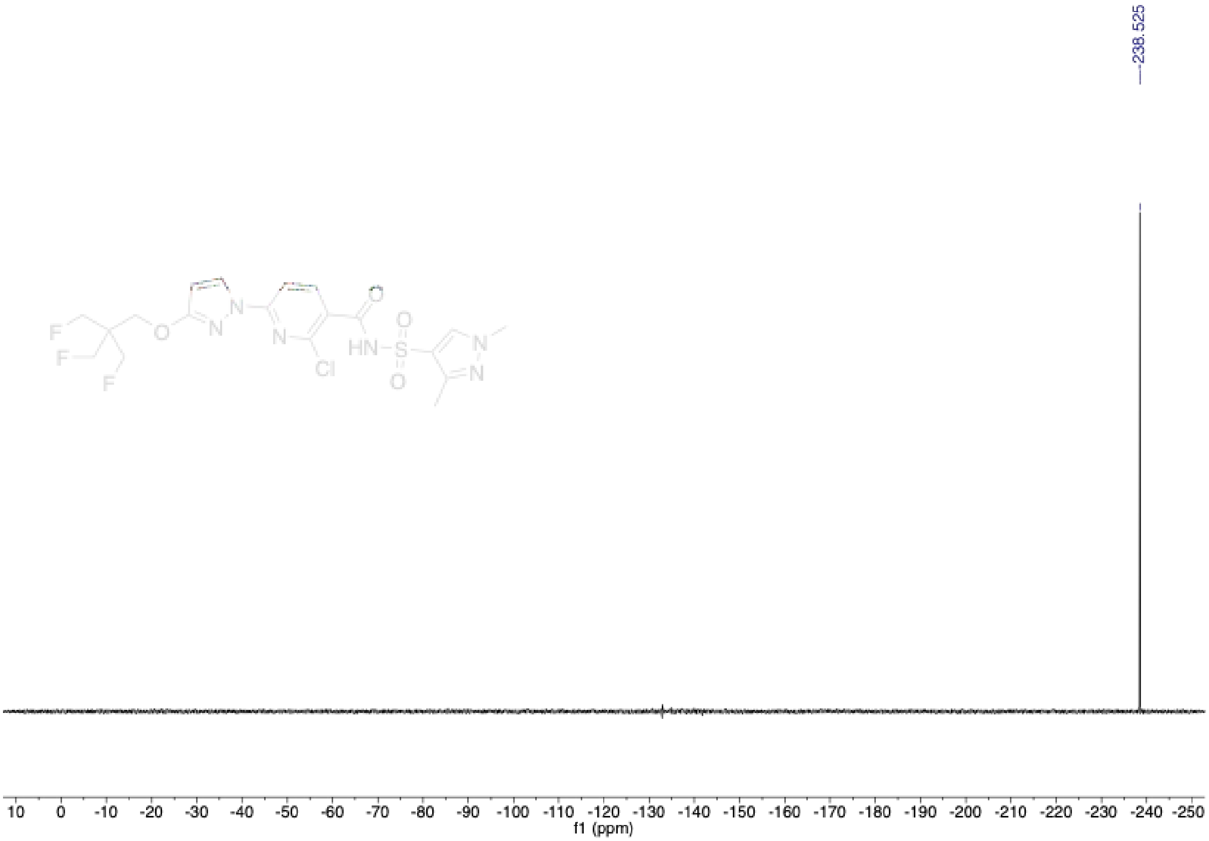

$^{13}C\{^{1}H\}$ NMR (126 MHz, $CDCl_3$)

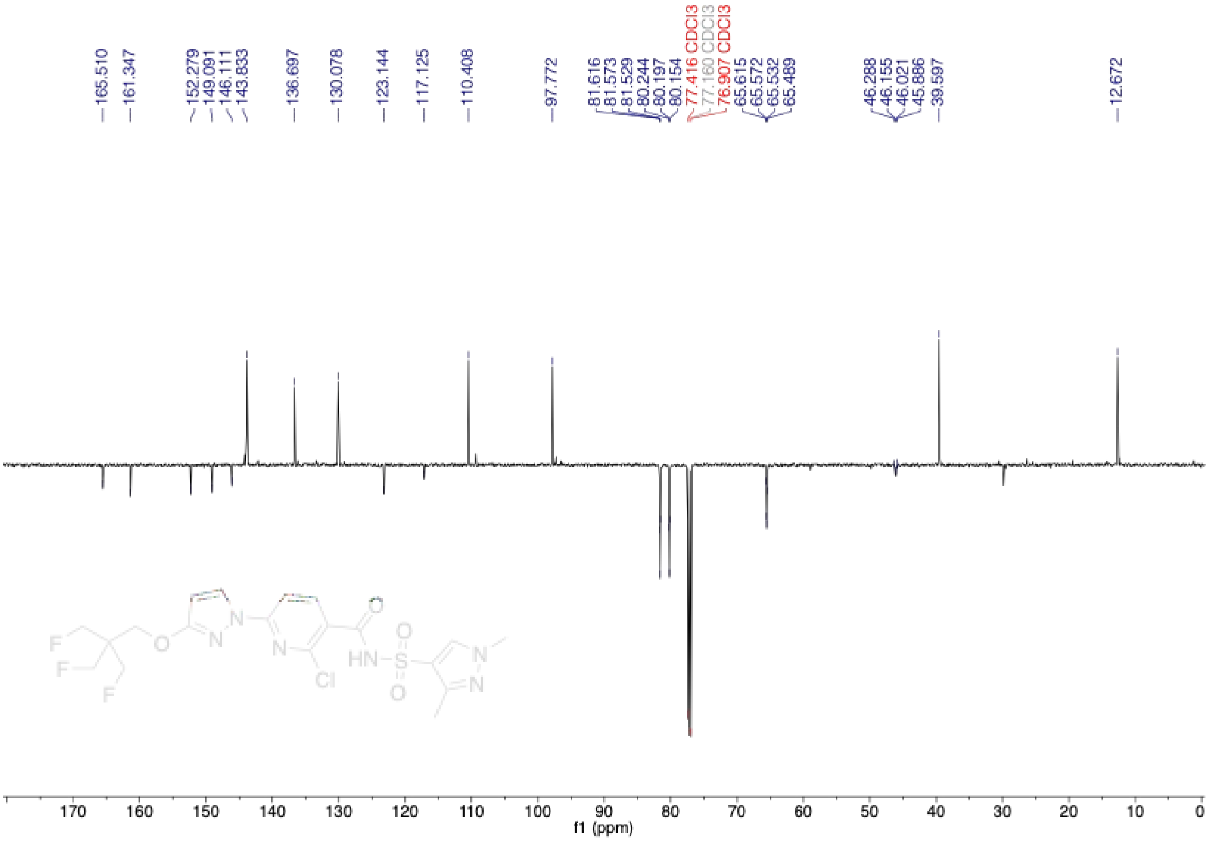

57

^{1}H NMR (400 MHz, DMSO)

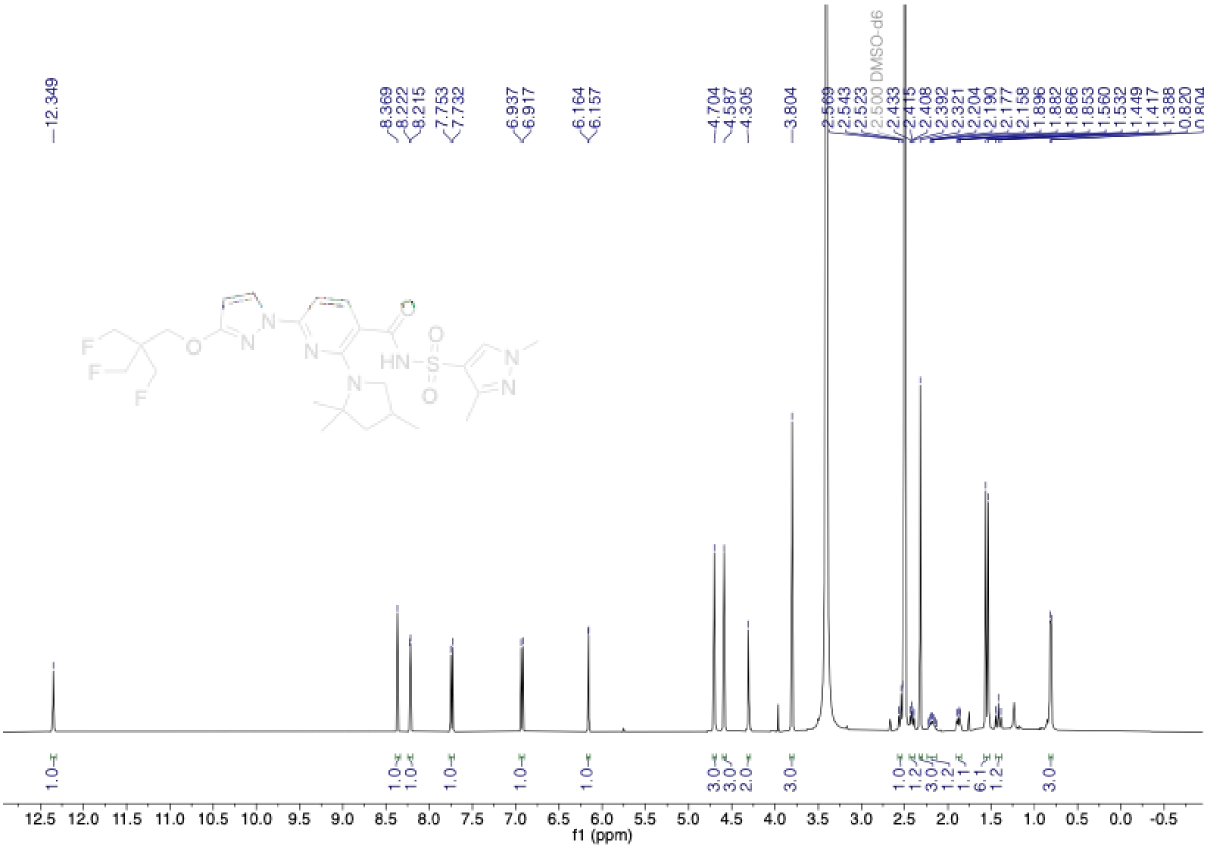

$^{19}F\{^{1}H\}$ NMR (377 MHz, DMSO)

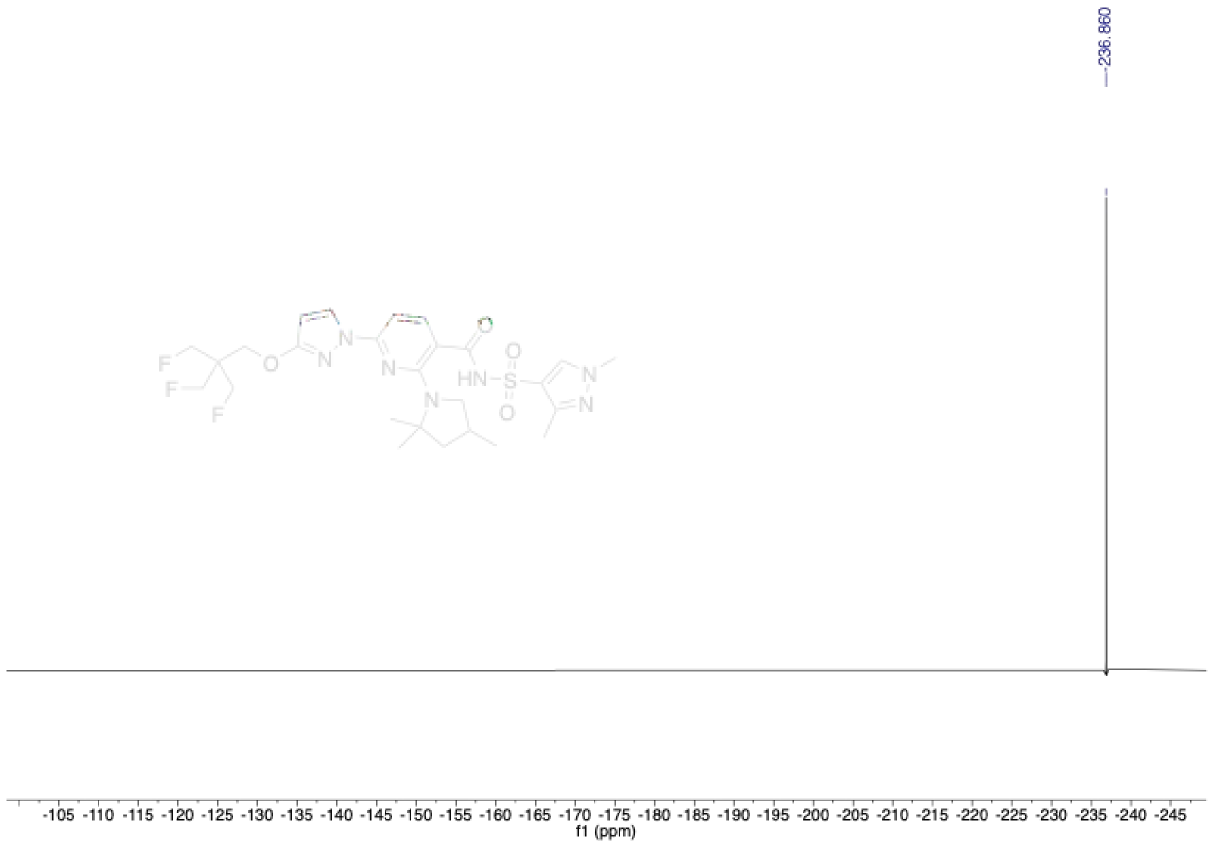

$^{13}C\{^{1}H\}$ NMR (126 MHz, DMSO)

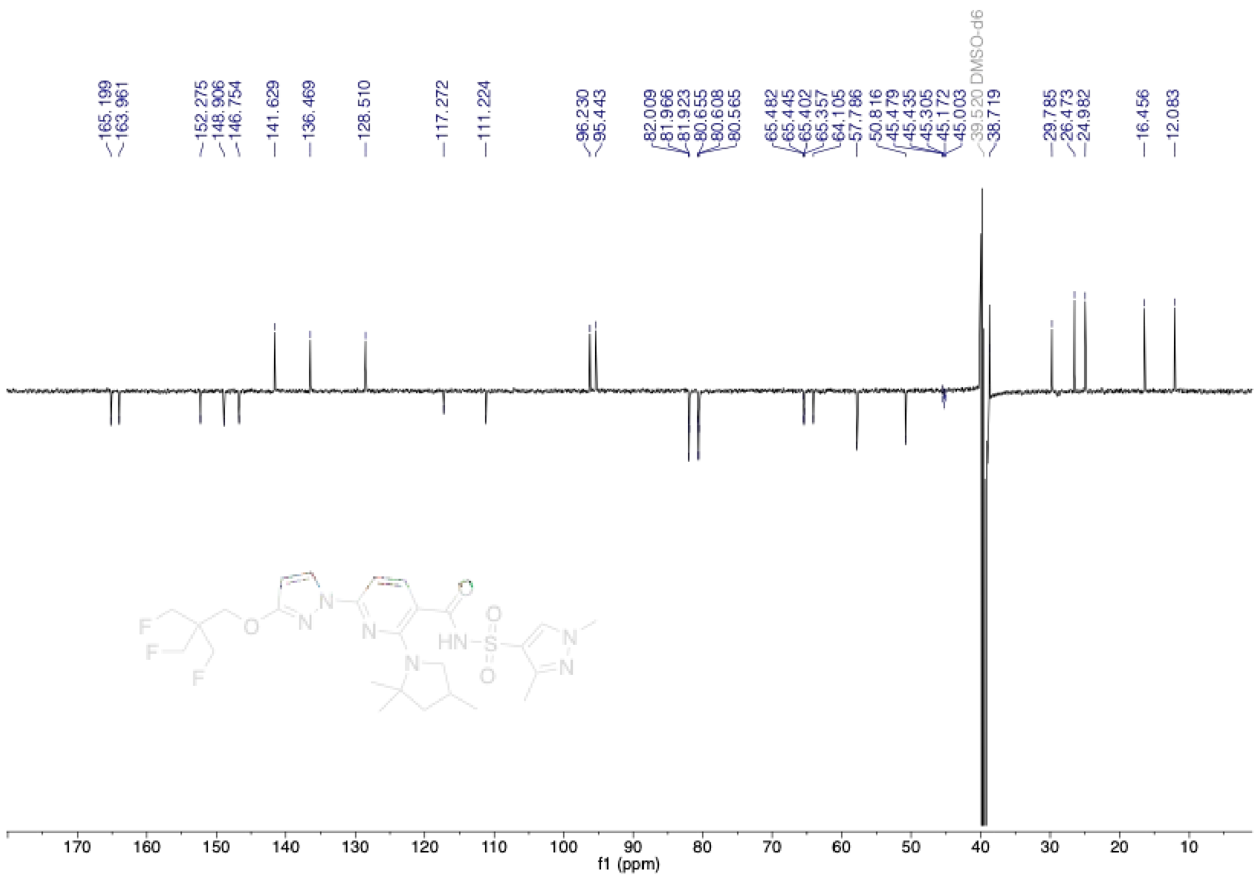

61

^1H NMR (400 MHz, $CDCl_3$)

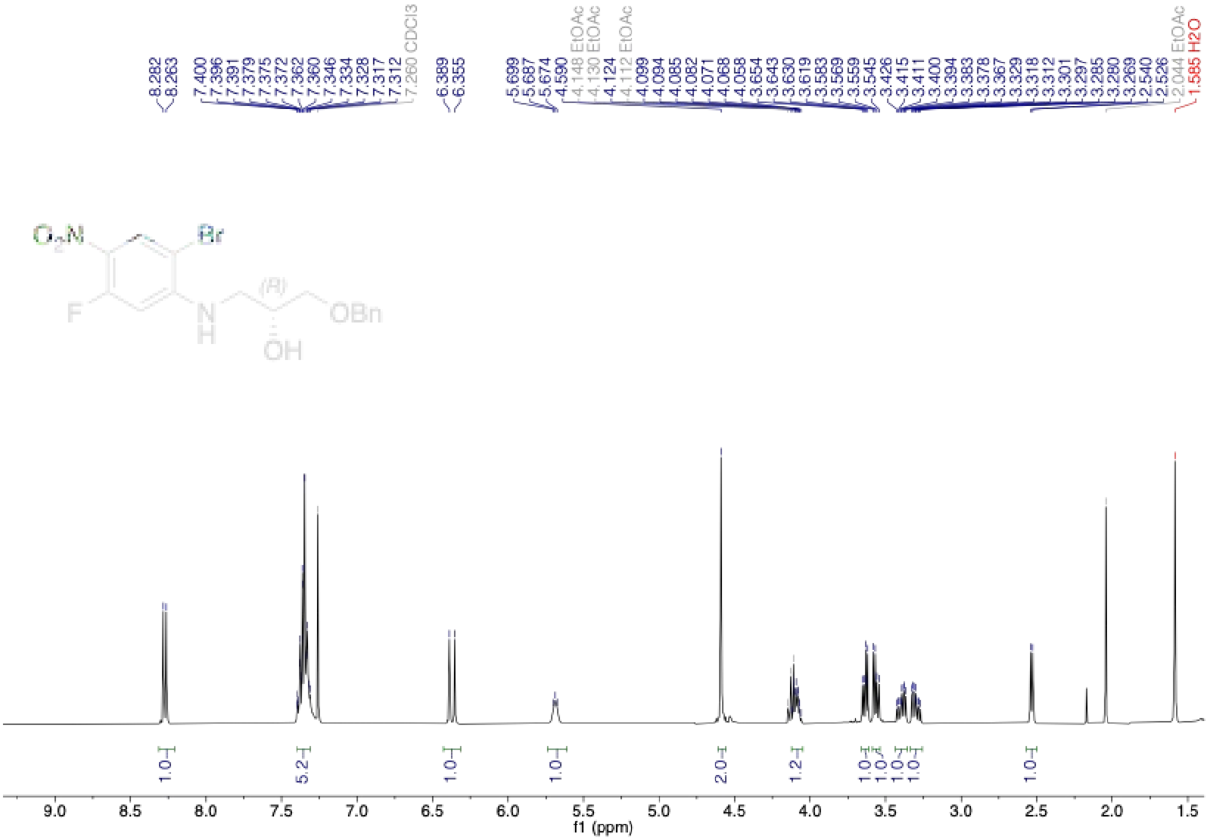

$^{19}F\{^{1}H\}$ NMR (377 MHz, $CDCl_3$)

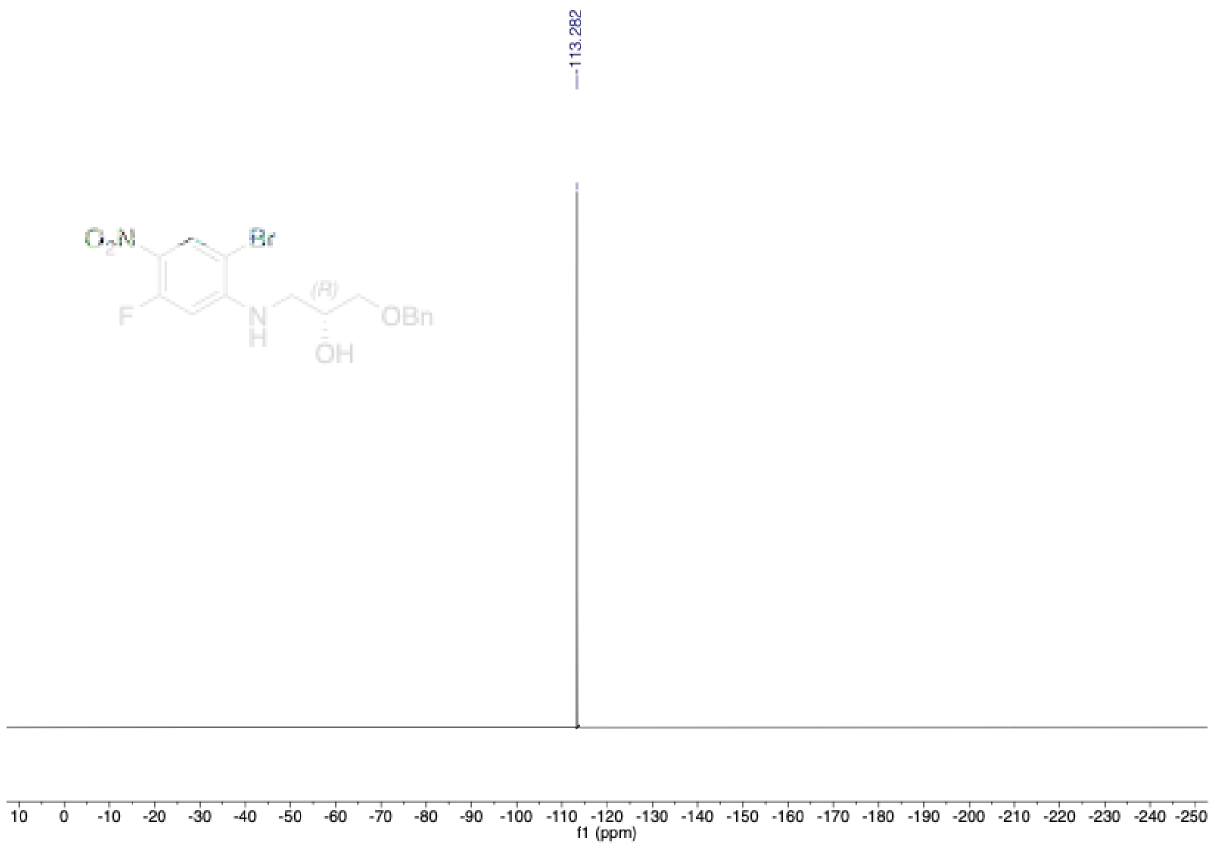

60

^{1}H NMR (400 MHz, $CDCl_3$)

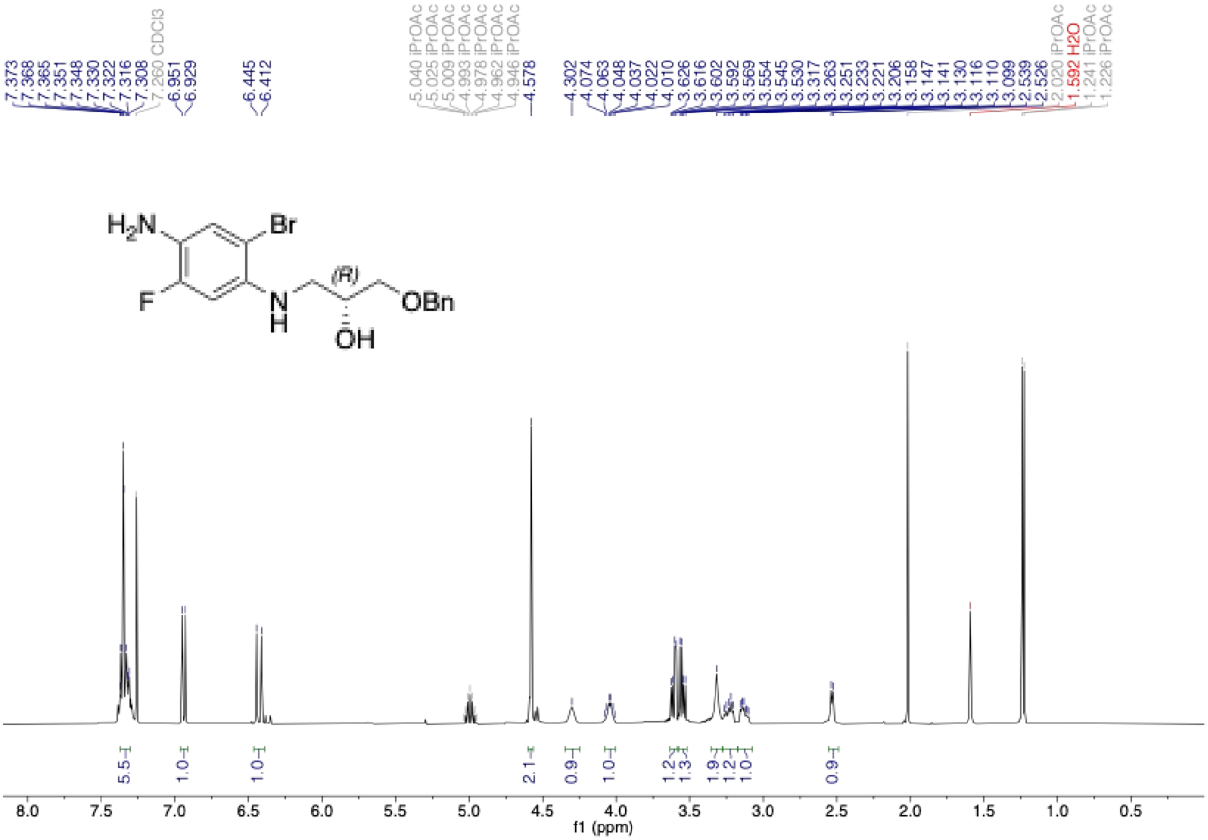

$^{19}F\{^{1}H\}$ NMR (470 MHz, $CDCl_3$)

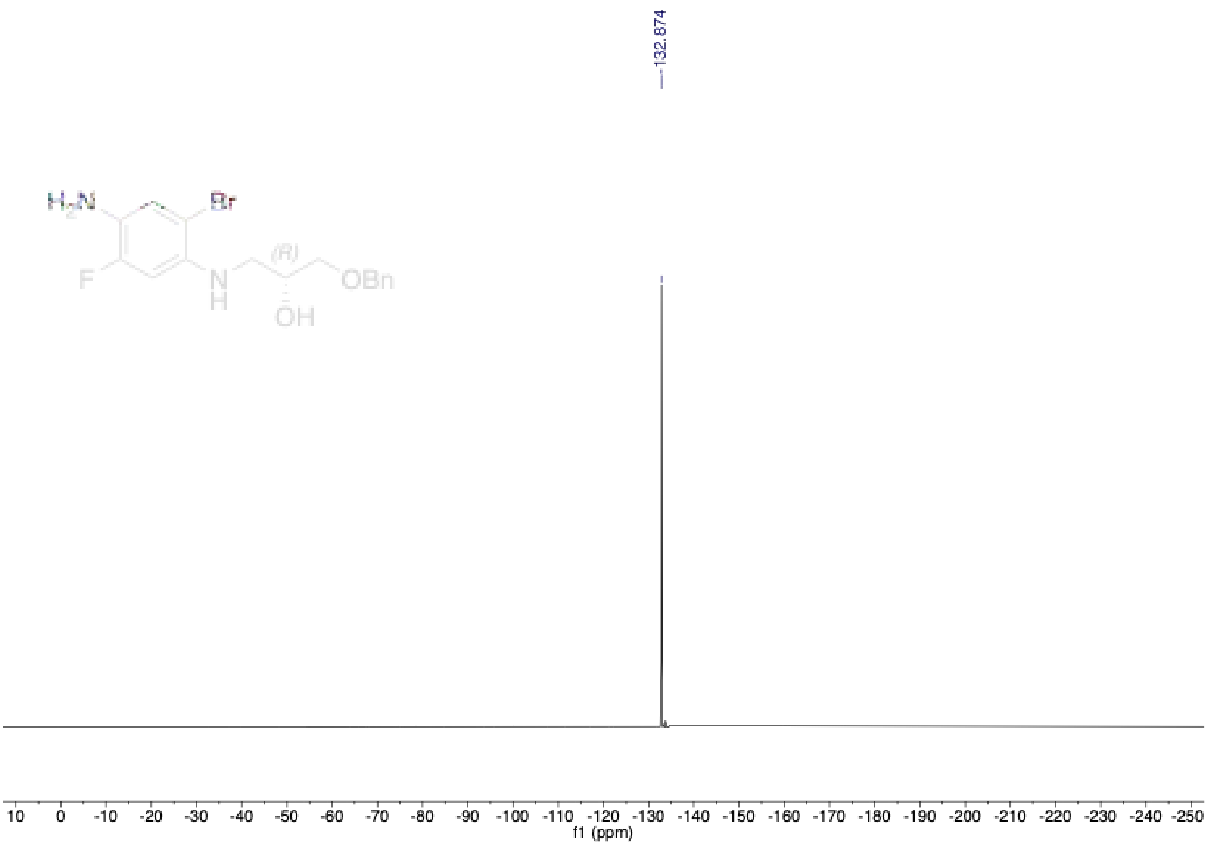

63

^{1}H NMR (400 MHz, $CDCl_3$)

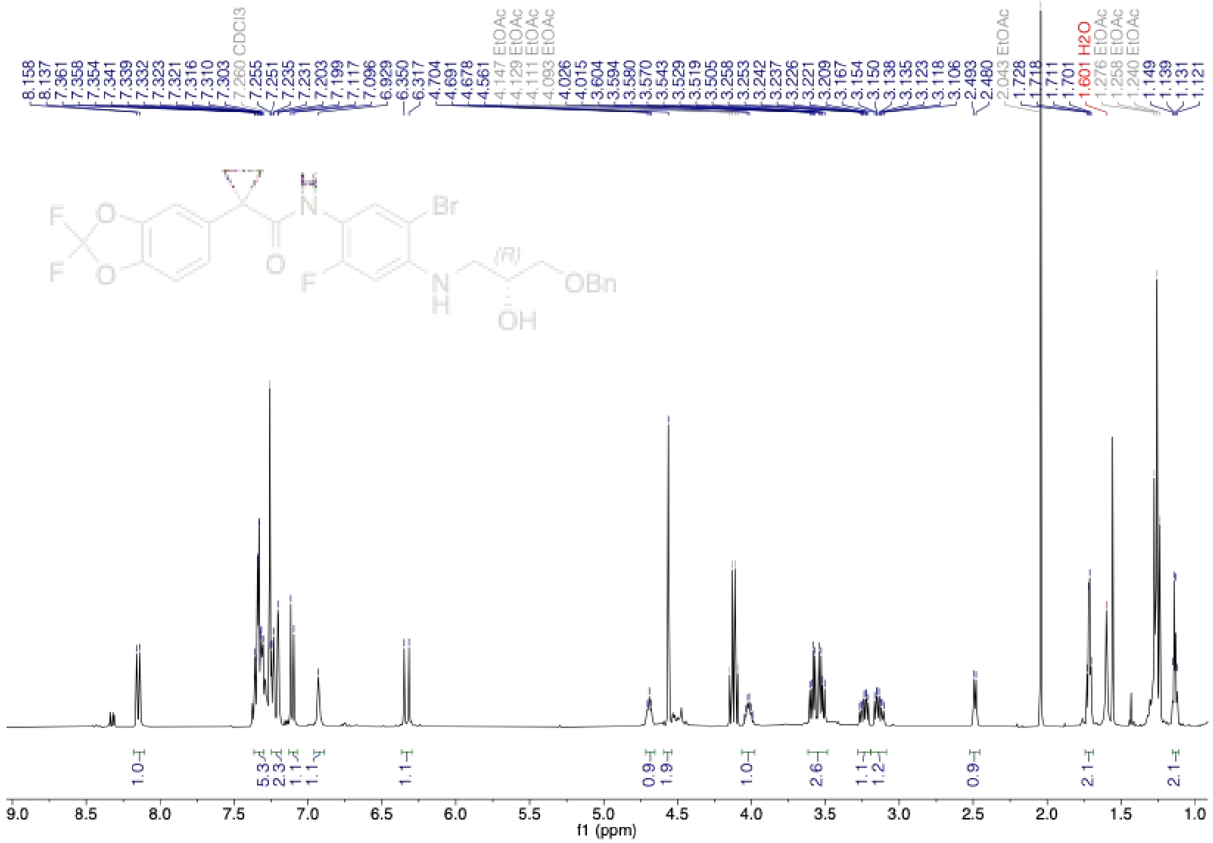

$^{19}F\{^{1}H\}$ NMR (377 MHz, $CDCl_3$)

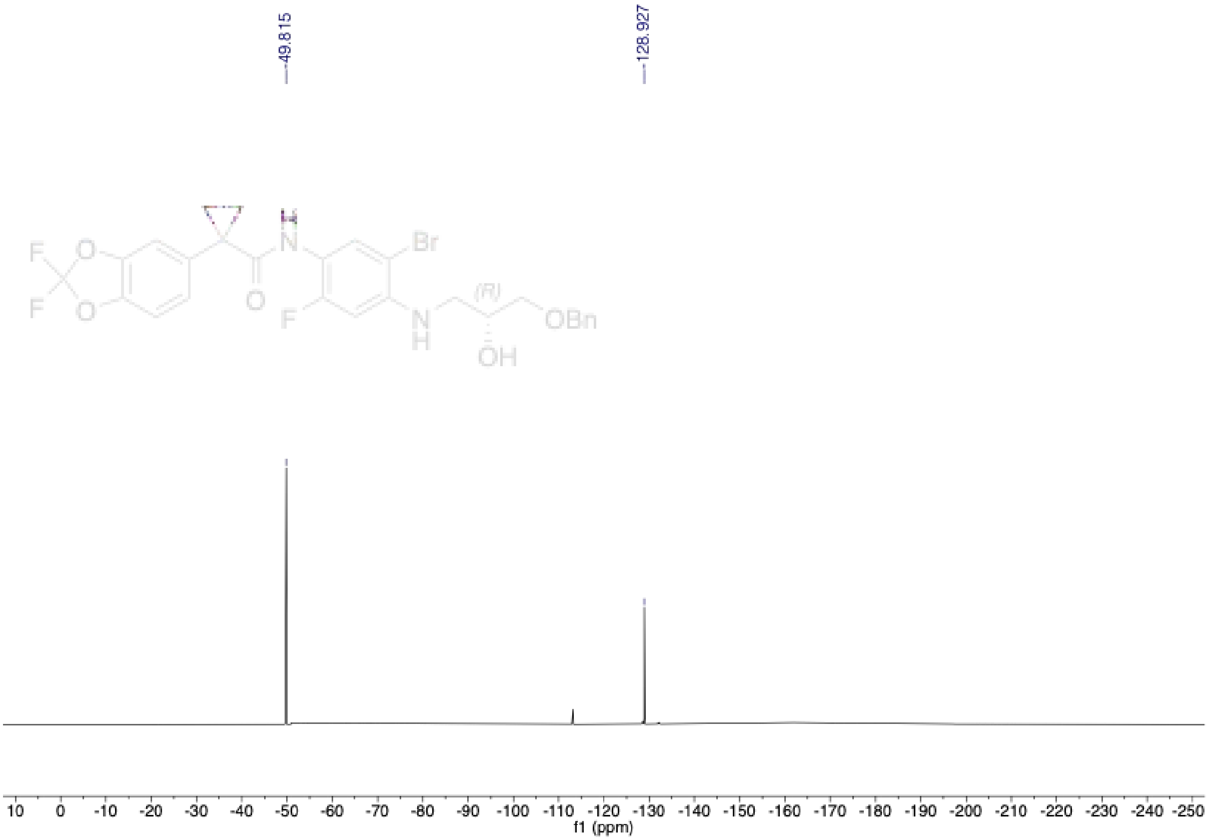

$^{13}C\{^{1}H\}$ NMR (126 MHz, $CDCl_3$)

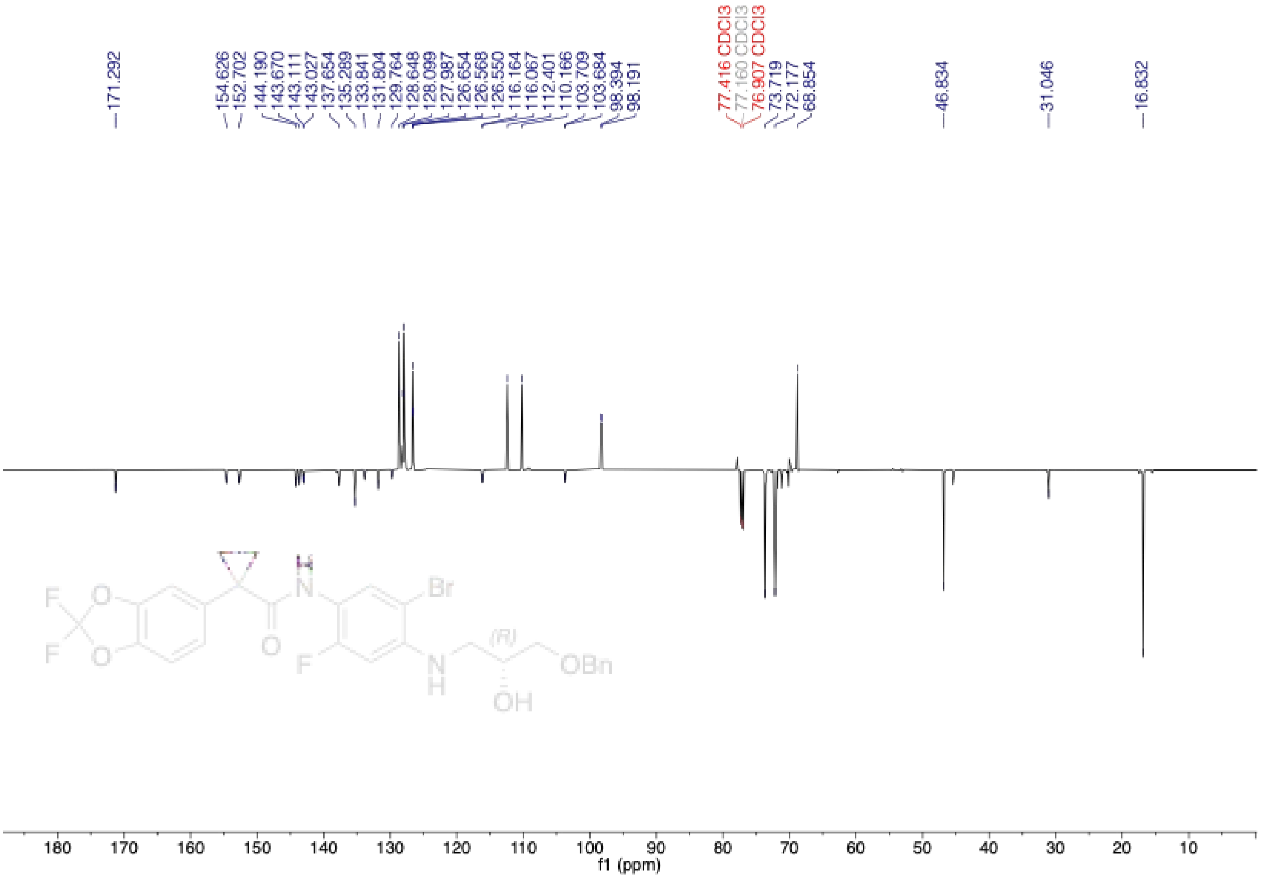

64

^{1}H NMR (500 MHz, $CDCl_3$)

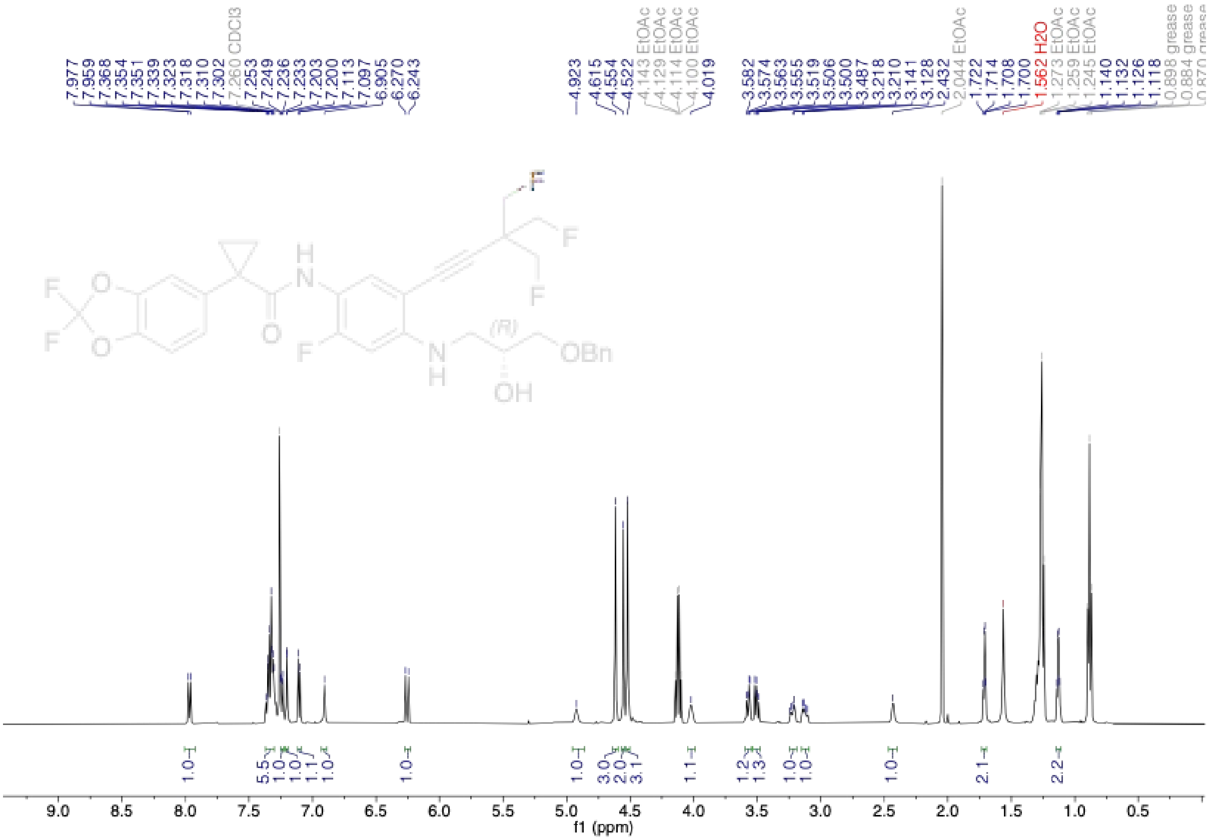

$^{19}F\{^{1}H\}$ NMR (470 MHz, $CDCl_3$)

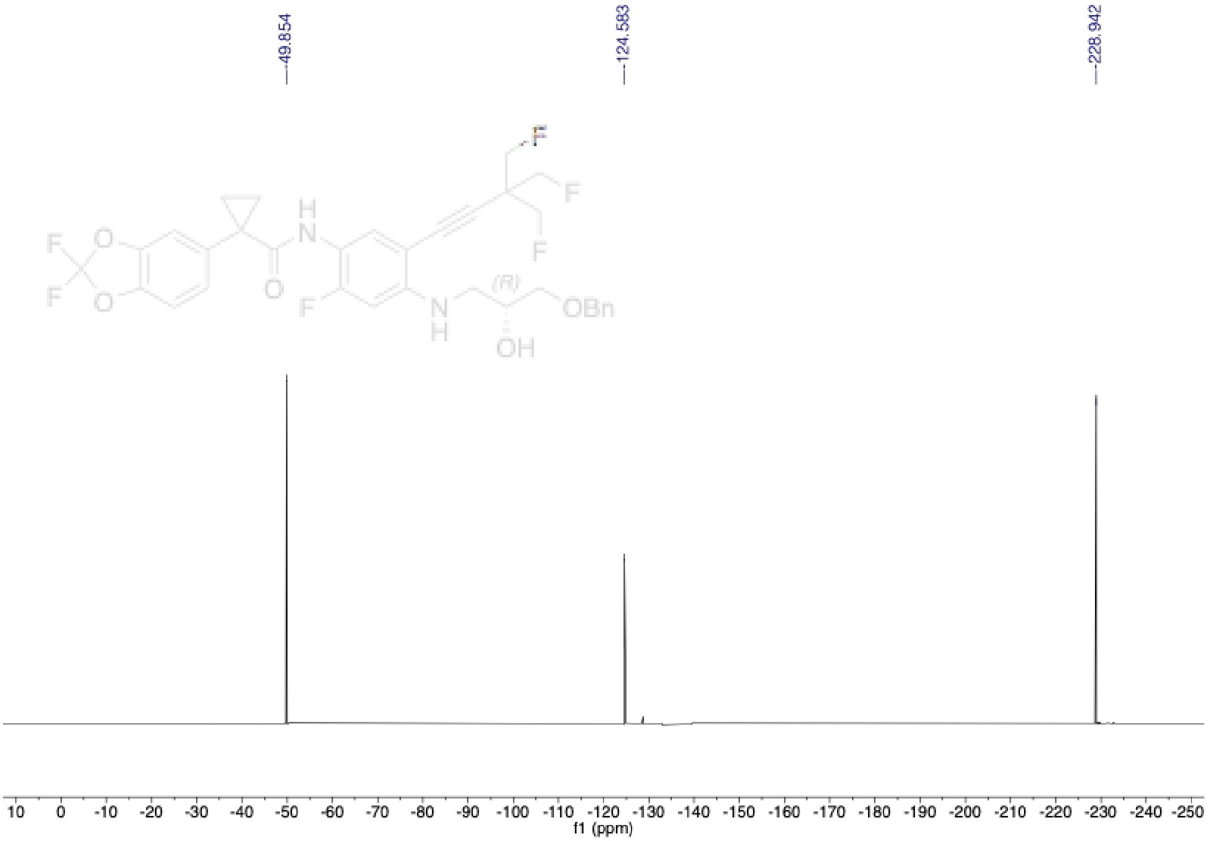

$^{13}C\{^{1}H\}$ NMR (126 MHz, $CDCl_3$)

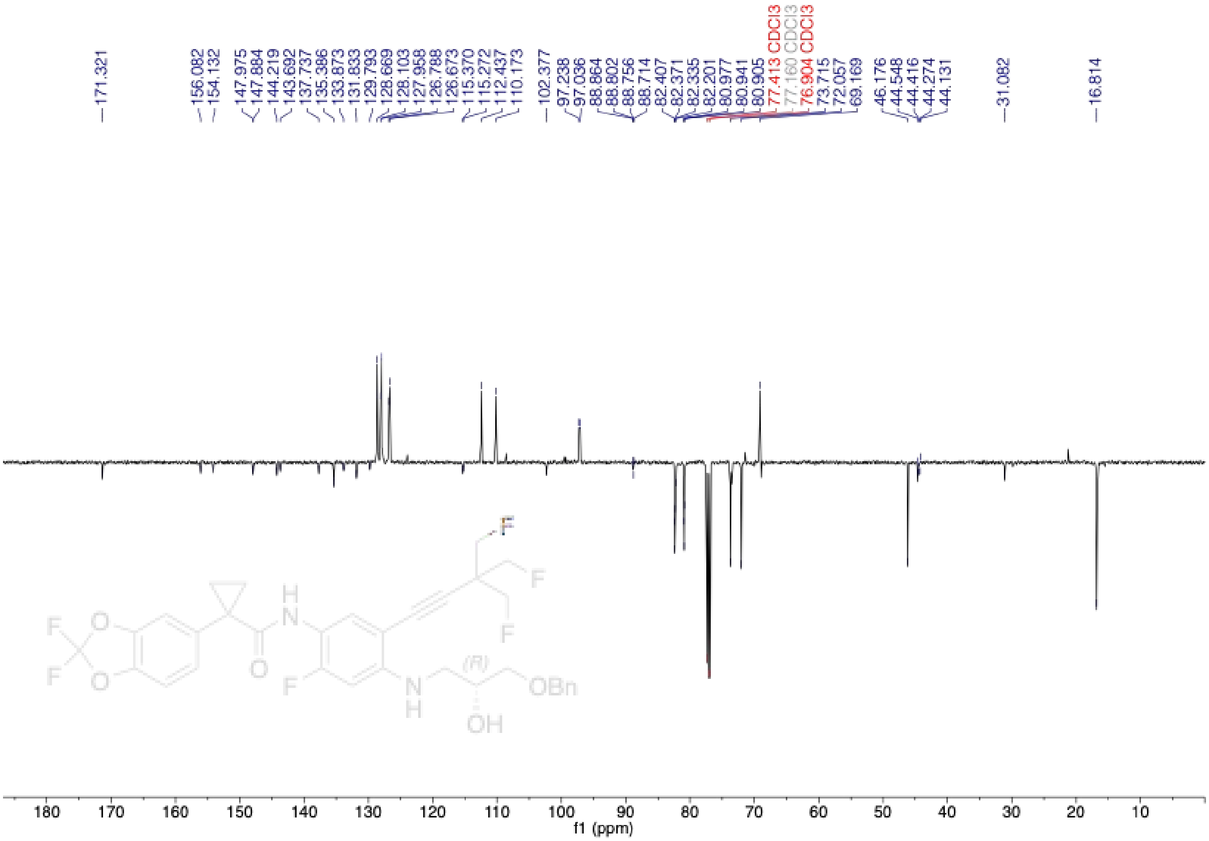

65

^1H NMR (500 MHz, $CDCl_3$)

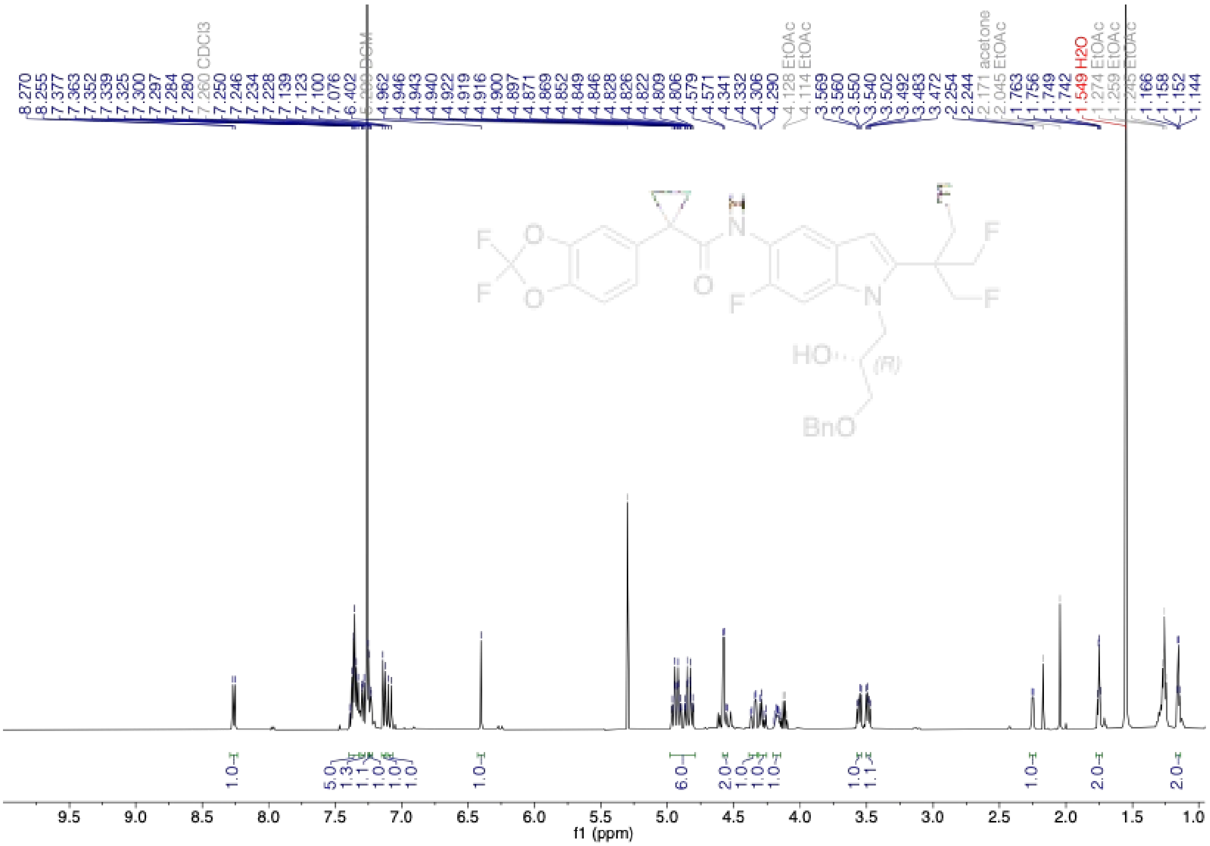

$^{19}F\{^{1}H\}$ NMR (470 MHz, $CDCl_3$)

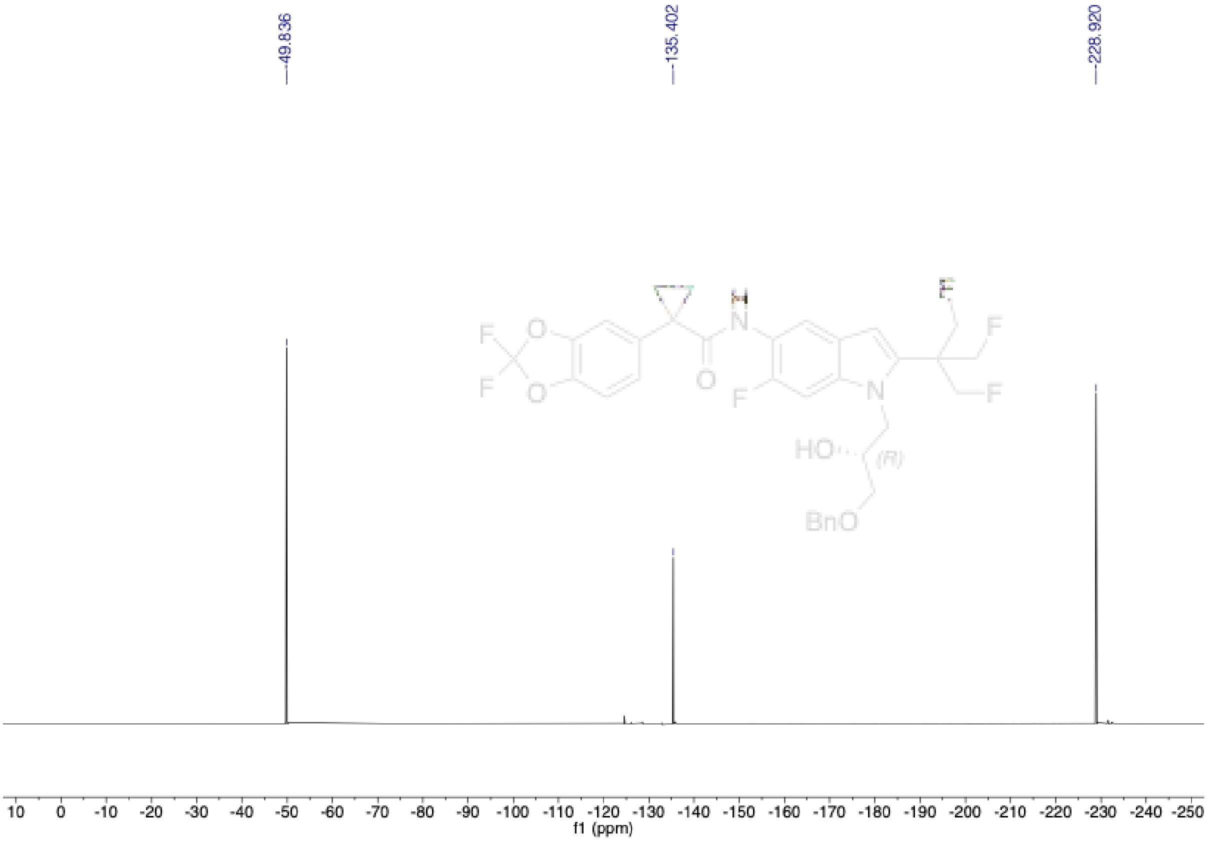

$^{13}C\{^{1}H\}$ NMR (126 MHz, $CDCl_3$)

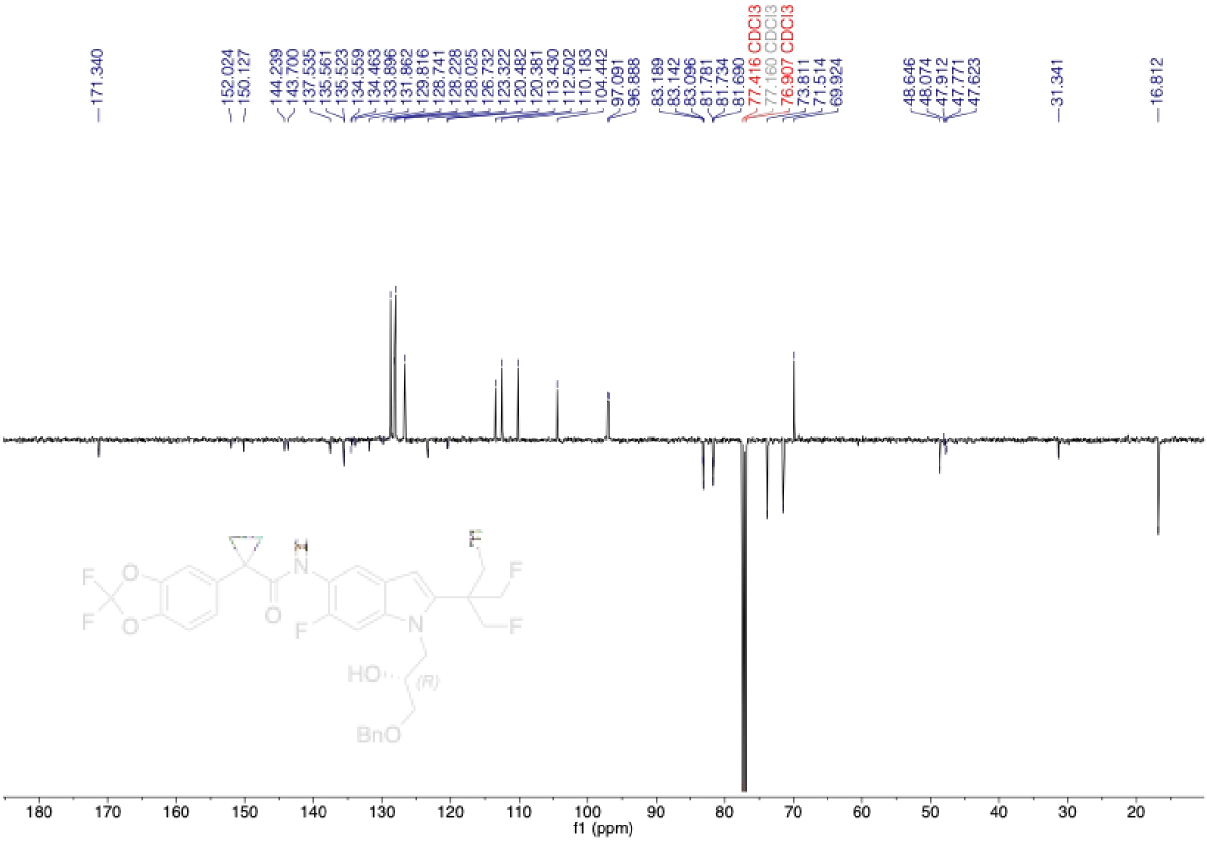

66

^{1}H NMR (500 MHz, CD_3CN)

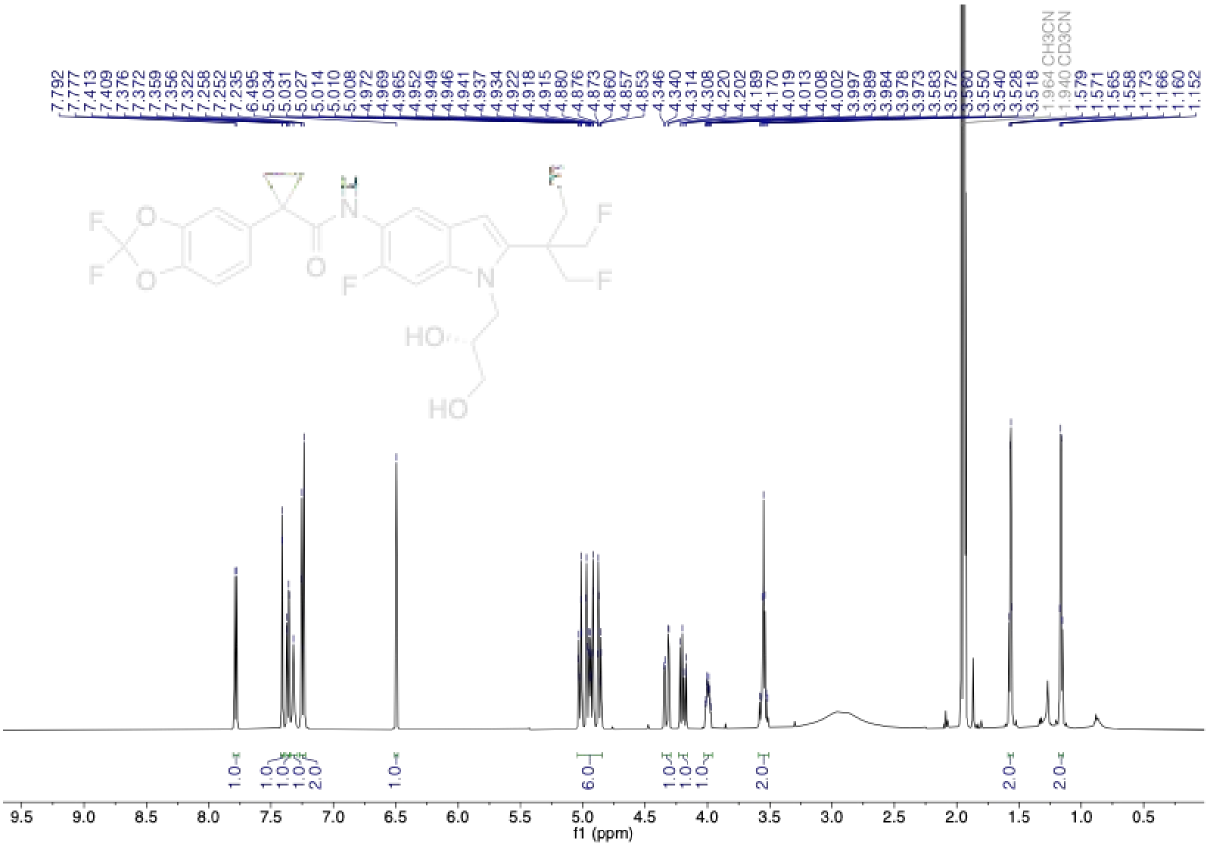

$^{19}F\{^{1}H\}$ NMR (470 MHz, CD_3CN)

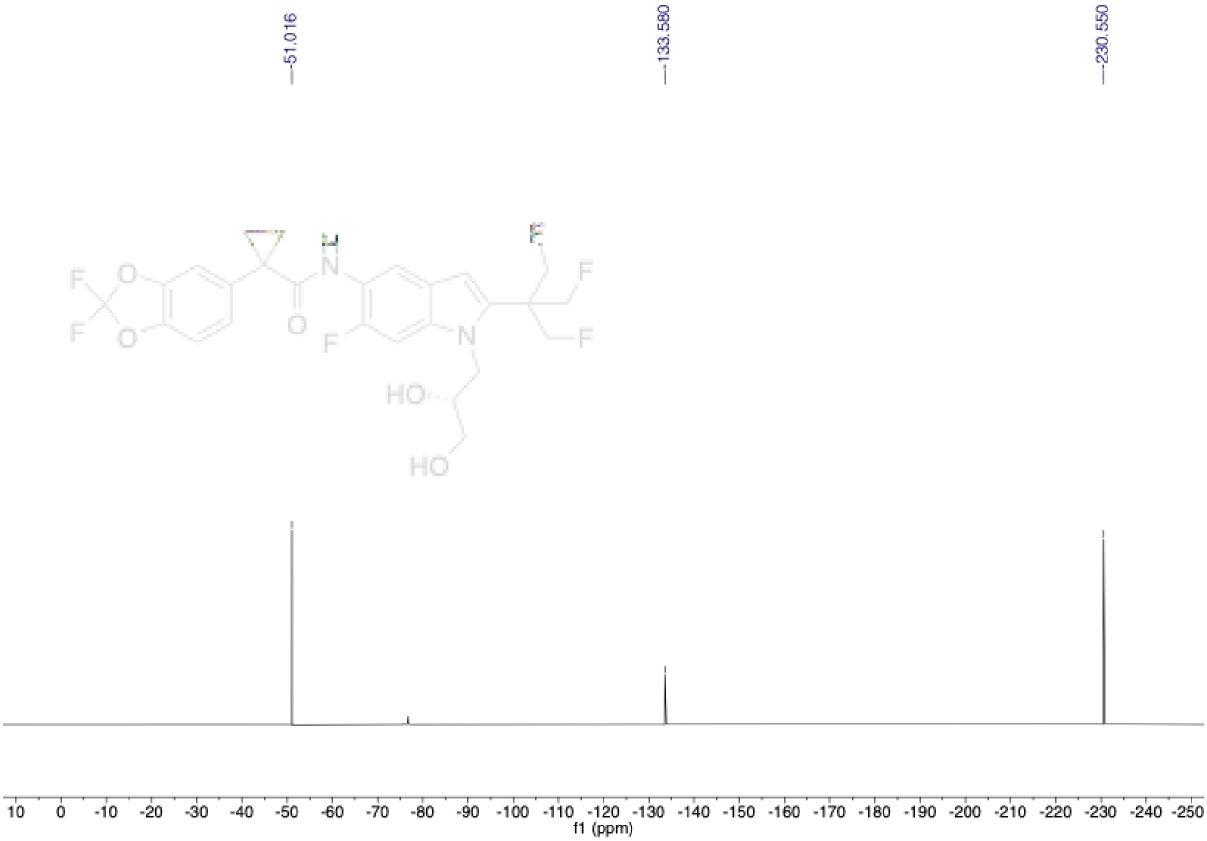

$^{13}C\{^{1}H\}$ NMR (126 MHz, CD_3CN)

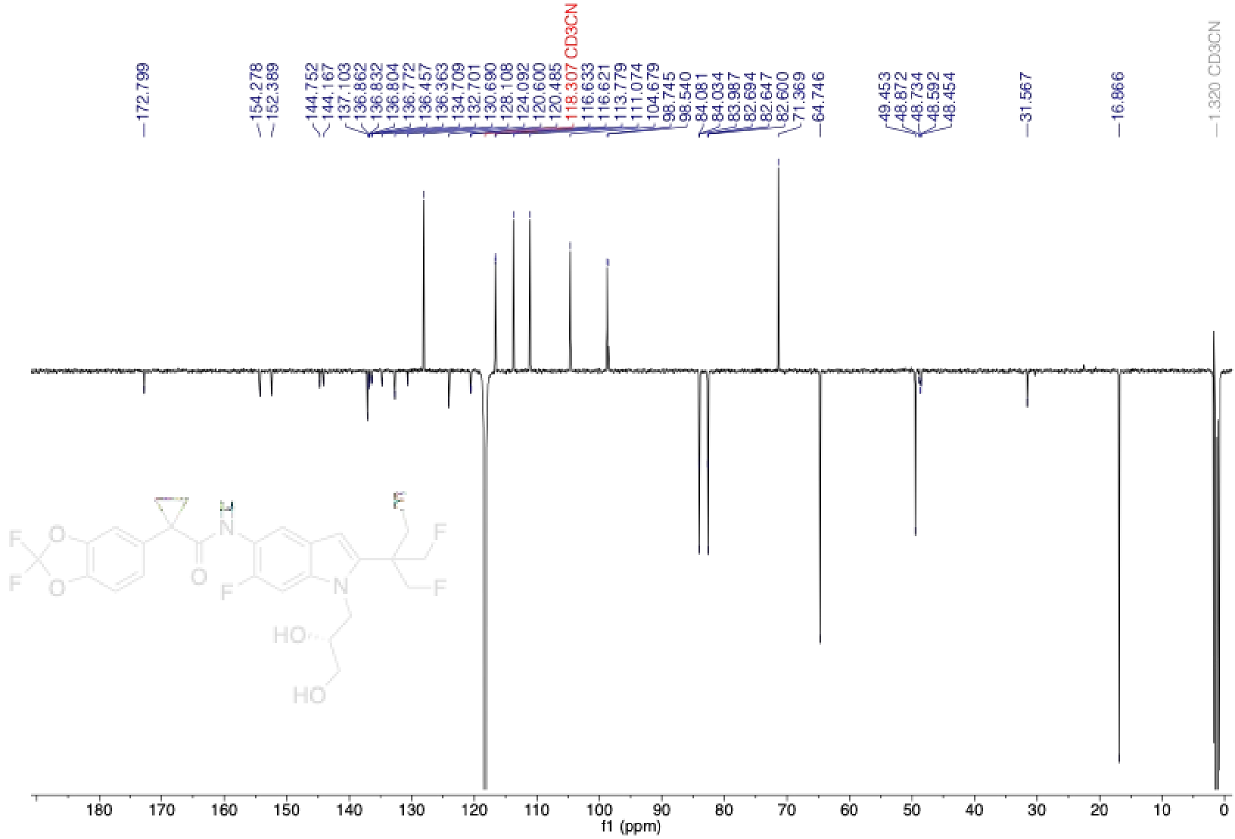

References

1 N. Arai, K. Azuma, N. Nii and T. Ohkuma, *Angew. Chem. Int. Ed.*, 2008, **47**, 7457–7460.

2 Y. K. Chen, A. E. Lurain and P. J. Walsh, *J. Am. Chem. Soc.*, 2002, **124**, 12225–12231.

3 I. A. Gorodetskaya, T.-L. Choi and R. H. Grubbs, *J. Am. Chem. Soc.*, 2007, **129**, 12672–12673.

4 O. Linnenberg, A. Kondinski, C. Stöcker and K. Yu. Monakhov, *Dalton Trans*, 2017, **46**, 15636–15640.

5 N. Roques, A. Tovar-Molle, C. Duhayon, S. Brandès, A. Spieß, C. Janiak and J. Sutter, *Chem. Eur. J.*, 2022, **28**, e202201935.

6 M. Desroses, T. Koolmeister, S. Jacques, S. Llona-Minguez, M.-C. Jacques-Cordonnier, A. Cázares-Körner, T. Helleday and M. Scobie, *Tetrahedron Lett.*, 2013, **54**, 3554–3557.

7 O. C. Finch, D. P. Furkert and M. A. Brimble, *Tetrahedron*, 2014, **70**, 590–596.

8 A. Lancelot, R. González-Pastor, R. Clavería-Gimeno, P. Romero, O. Abian, P. Martín-Duque, J. L. Serrano and T. Sierra, *J. Mater. Chem. B*, 2018, **6**, 3956–3968.

9 M. T. La, B. Jeong and H. Kim, *Bull. Korean Chem. Soc.*, 2021, **42**, 740–743.

10 B. Linclau, F. Peron, E. Bogdan, N. Wells, Z. Wang, G. Compain, C. Q. Fontenelle, N. Galland, J. Le Questel and J. Graton, *Chem. Eur. J.*, 2015, **21**, 17808–17816.

11 Y. Katayama, Y. Nishikawa, M. Inagaki, J. Park, D.-C. Oh and Y. Masuda, *Tetrahedron Lett.*, 2024, **145**, 155177.

12 Y. Nian, J. Wang, H. Moriwaki, V. A. Soloshonok and H. Liu, *Dalton Trans*, 2017, **46**, 4191–4198.

13 V. A. Soloshonok, D. V Avilov, V. P. Kukhar’, V. I. Tararov, T. F. Savel’eva, T. D. Churkina, N. S. Ikonnikov, K. A. Kochetkov, S. A. Orlova, A. P. Pysarevsky, Y. T. Struchkov, N. I. Raevsky and Y. N. Belokon’, *Tetrahedron Asymmetry*, 1995, **6**, 1741–1756.

14 D. Klyukovskyi, O. S. Liashuk, M. Redka, A. T. Bezuhla, O. P. Blahun, D. Lesyk, P. Borysko, A. V. Chernykh, Y. M. Pustovit and O. O. Grygorenko, *Chem. Eur. J.*, 2026, **32**, e70736.

15 O. C. Finch, D. P. Furkert and M. A. Brimble, *Tetrahedron*, 2014, **70**, 590–596.

16 H. J. Ross, Y. Yu, L. Atkin, M. Ghorbani, K. Mint, N. Warne, K. Kempe and D. L. Priebbenow, *J. Am. Chem. Soc.*, 2025, **147**, 24734–24746.

17 A. R. Abela, T. Alcacio, C. Anderson, P. T. Angell, M. Baek, J. J. Clemens, T. Cleveland, L. A. Ferris, P. D. J. Grootenhuis, R. S. Gross, A. Gulevich, S. S. Hadida Ruah, C. K.-J. Hsia, R. M. Hughes, P. V. Joshi, P. Kang, A. Keshavarz-Shokri, H. Khatuya, P. J. Krenitsky, J. McCartney, M. T. Miller, P. Paraselli, F. J. D. Pierre, Y. Shi, M. Shrestha, D. A. Siesel, K. Stravropoulos, A. P. Termin, J. Uy, F. F. Van Goor, T. J. Young and J. Zhou, WO/2018/107100, 2018.

18 D. T. Belmont, C. Harrison, R. M. Hughes, Y. C. Jung, E. C. Lee, B. J. Littler, P. J. Rose, D. A. Siesel and G. J. Tanoury, 9035072 B2, 2015.

19 N. Fleck, R. M. Thomas, M. Müller, S. Grimme and B. H. Lipshutz, *Green Chemistry*, 2022, **24**, 6517–6523.

20 J. Sangster, *J. Phys. Chem. Ref. Data*, 1989, **18**, 1111–1229.

21 C. Hansch, A. Leo and D. H. Hoekman, *Exploring QSAR*, American Chemical Society, 1995.

Computational Details

A development version of Autobench[22] was employed to perform a comprehensive single-point energy benchmarking workflow for compounds **28**, **29**, and **35–38**. Initially, conformational searches were carried out for each compound using the Global Optimizer Algorithm (GOAT)[23], as implemented in ORCA 6.1.0[24], in combination with the extended semiempirical tight-binding method (GFN2-xTB)[25] and implicit solvation for chloroform ($CHCl_3$) described by the Analytical Linearized Poisson–Boltzmann (ALPB) model[26]. The resulting conformers were fully optimized at the ALPB($CHCl_3$)-GFN2-xTB level and characterized as true minima by numerical frequency calculations at the same level of theory, showing no imaginary frequencies.

Conformers were compared based on heavy-atom RMSD values and relative energies, allowing the identification and removal of duplicate structures. The remaining unique conformers were subsequently reoptimized using the composite ωB97X-3c functional[27] with SMD implicit solvation[28]. Duplicate conformers were again eliminated based on RMSD and relative energy comparisons. In addition, conformers exhibiting Boltzmann populations below 0.5% were considered unlikely to contribute meaningfully to spectroscopic properties and were therefore discarded. All retained conformers were confirmed as true minima by analytical frequency calculations, with no imaginary frequencies observed.

Relative single-point energies were then computed for the resulting conformers using several combinations of DFT functionals and basis sets (Table S1). These values were benchmarked against reference relative energies obtained at the DLPNO-CCSD(T) level[29] using Dunning's cc-pVDZ, cc-pVTZ, and cc-pVQZ basis sets[30] extrapolated to the complete basis set (CBS) limit. According to the benchmarking results, the r^2SCANh/pc-2 level of theory[31,32] yielded a low mean absolute error (MAE) relative to the reference data (Table 1) and was therefore selected for all subsequent calculations.

All conformers were then reoptimized at the r^2SCANh/pc-2 level and verified as true minima by analytical frequency calculations at the same level of theory, again showing no imaginary frequencies. Duplicate conformers were identified through RMSD and relative energy analyses, and conformers with Boltzmann populations below 1% were considered negligible contributors to Boltzmann-averaged spectroscopic properties and excluded.

NMR chemical shieldings for all retained conformers were calculated at the r^2SCANh/pcSseg-2[33] level, using geometries optimized at the r^2SCANh/pc-2 level. The pcSseg-2 basis set, a

variant of pc-2 specifically designed for improved treatment of core electrons and accurate prediction of chemical shifts, was employed for these calculations. Final calculated chemical shifts were obtained by the difference between the calculated chemical shielding of a reference molecule (TMS) and the target molecule following the equation:

$$\delta_{calc} = \sigma_{TMS} - \sigma_{targetmol}, \quad \sigma_{TMS} = 31.62\ ppm \qquad \text{(Equation 1)}$$

Chemical shifts reported in the main text are the contribution of each conformer weighted by their Boltzmann population in terms of ΔG at 298.15K. Thermodynamical corrections to afford the relative Gibbs free energies were obtained from the frequency calculations at 298.15K. Natural Chemical Shielding (NCS)[34] analyses were also performed at the r^2SCANh/pcSseg-2 level using the NBO 7.0 program[35]. Chloroform solvation effect was implicitly incorporated in all DFT calculations through the SMD model. All calculations were carried out with ORCA 6.1.0.

The $CH_2F\cdots H^a$ NCHBs for the conformer exhibiting the shortest $CH_2F\cdots H^a$ contact in each alcohol and ketone derivative was further analyzed in the framework of Natural Bond Orbitals[36] and the Natural Coulomb Analysis with NPA atomic charges[37] at the SMD($CHCl_3$)-r^2SCANh/pc-2, as well as NCI[17] and QTAIM[18] analysis over the SMD($CHCl_3$)-r^2SCANh/pc-2 wave functions.

References

22. R. A. Cormanich; G. D. da Silva, *J. Chem. Inf. Model.*, 2024, **64**, 3322–3331.

23. B. de Souza, *Angew. Chem. Int. Ed.*, 2025, **64**, e202500393

24. F. Neese, *Wires Comput. Molec. Sci.*, 2022, **12**, e1606.

25. C. Bannwarth; S. Ehlert; S. Grimme, *J. Chem. Theory Comput.*, 2019, **15**, 1652–1671.

26. S. Ehlert; M. Stahn; S. Spicher; S. Grimme, *J. Chem. Theor. Comp.*, 2021, **17**, 4250-4261.

27. M. Müller; A. Hansen; S. Grimme, *J. Chem. Phys.*, 2023, **158**, 014103.

28. A. V. Marenich, C. J. Cramer, D. G. Truhlar, *J. Phys. Chem. B*, 2009, **113**, 6378-6396.

29. C. Riplinger; F. Neese, *J. Chem. Phys.*, 2013, **138**, 034106–034125.

30. T. H. Dunning, *J. Chem. Phys.*, 1989, **90**, 1007-1023.

31. M. Bursch; H. Neugebauer; S. Ehlert; S. Grimme, *J. Chem. Phys.*, 2022, **156**, 134105-134116.

32. F. Jensen, *J. Chem. Phys.*, 2001, **115**, 9113–9125.

33. F. Jensen, *J. Chem. Theory Comput.*, 2015, **11**, 132-138.

34. J. A. Bohmann; F. Weinhold; T. C. Farrar, *J. Chem. Phys.*, 1997, **107**, 1173-1184

35. E. D. Glendening, J. K. Badenhoop, A. E. Reed, J. E. Carpenter, J. A. Bohmann, C. M. Morales, P. Karafiloglou, C. R. Landis, F. Weinhold, F. NBO 7.0, Theoretical Chemistry Institute, University of Wisconsin, Madison, 2018.

36. F. Weinhold; C. R. Landis; E. D. Glendening, *Int. Rev. Phys. Chem.*, 2016, **35**, 399–440.

37. A. E. Reed; R. B. Weinstock; F. Weinhold, *J. Chem. Phys.*, 1985, **83**, 735-746.

38. (a) E. R. Johnson; S. Keinan; P. M. Sánchez; J. C. García; A. J. Cohen; W. Yang, *J. Am. Chem; Soc.*, 2010, **132**, 6498-6506. (b) R. A. Boto; F. Peccati; R. Laplaza; C. Quan; A. Carbone; J. P. Piquemal; Y. Maday; J. C. García, *J. Chem. Theot. Comput.*, 2020, **16**, 4150-4158.

39. (a) AIMAll (Version 19.10.12), Todd A. Keith, TK Gristmill Software, Overland Park KS, USA, 2019. (b) "Atoms in Molecules: A Quantum Theory", R.F.W. Bader, Oxford University Press, Oxford (1990).

Table S6. Mean absolute errors (MAEs) of all tested theoretical methods compared to the reference DLPNO-CCSD(T)/CBS calculations for compounds **28**, **29** and **35**-**38**. The method chosen for subsequent calculations is highlighted in green.

Theoretical Level	28	35	36	29	37	38	Avg MAE
SMD($CHCl_3$)-M06-2X-D3/pc-1	0.19	0.35	0.25	0.36	0.31	0.42	0.31
SMD($CHCl_3$)-M06-2X-D3/pc-2	0.06	0.07	0.07	0.17	0.19	0.14	0.12
SMD($CHCl_3$)-M06-L-D3/pc-1	0.19	0.37	0.22	0.36	0.35	0.32	0.30
SMD($CHCl_3$)-M06-L-D3/pc-2	0.18	0.19	0.15	0.22	0.17	0.16	0.18
SMD($CHCl_3$)-B3LYP-D4/pc-1	0.19	0.39	0.21	0.47	0.24	0.36	0.31
SMD($CHCl_3$)-B3LYP-D4/pc-2	0.07	0.16	0.08	0.10	0.12	0.13	0.11
SMD($CHCl_3$)-BHandHLYP-D4/pc-1	0.25	0.44	0.28	0.39	0.27	0.31	0.32
SMD($CHCl_3$)-BHandHLYP-D4/pc-2	0.07	0.09	0.08	0.10	0.12	0.11	0.10
SMD($CHCl_3$)-TPSS-D4/pc-1	0.18	0.31	0.18	0.37	0.32	0.46	0.30
SMD($CHCl_3$)-TPSS-D4/pc-2	0.12	0.14	0.12	0.16	0.13	0.20	0.15
SMD($CHCl_3$)-TPSSh-	0.18	0.29	0.17	0.41	0.29	0.39	0.29

D4/pc-1							
SMD($CHCl_3$)-TPSSh-D4/pc-2	0.10	0.12	0.10	0.15	0.12	0.16	0.13
SMD($CHCl_3$)-TPSS0-D4/pc-1	0.18	0.24	0.14	0.46	0.27	0.40	0.28
SMD($CHCl_3$)-TPSS0-D4/pc-2	0.06	0.10	0.08	0.14	0.11	0.13	0.10
SMD($CHCl_3$)-PBE-D4/pc-1	0.17	0.36	0.20	0.30	0.33	0.55	0.32
SMD($CHCl_3$)-PBE-D4/pc-2	0.08	0.12	0.08	0.11	0.13	0.18	0.12
SMD($CHCl_3$)-PBE0-D4/pc-1	0.16	0.31	0.18	0.40	0.26	0.41	0.29
SMD($CHCl_3$)-PBE0-D4/pc-2	0.05	0.09	0.06	0.09	0.09	0.12	0.08
SMD($CHCl_3$)-r^2SCAN-D4/pc-1	0.19	0.41	0.25	0.40	0.34	0.39	0.33
SMD($CHCl_3$)-r^2SCAN-D4/pc-2	0.06	0.07	0.07	0.10	0.10	0.11	0.09
SMD($CHCl_3$)-r^2SCANh-D4/pc-1	0.19	0.35	0.21	0.40	0.31	0.35	0.30
SMD($CHCl_3$)-r^2SCANh-D4/pc-2	0.06	0.07	0.08	0.09	0.09	0.09	0.08
SMD($CHCl_3$)-r^2SCAN0-D4/pc-1	0.19	0.33	0.24	0.39	0.32	0.49	0.33

SMD($CHCl_3$)-r^{2}SCAN0-D4/pc-2	0.08	0.09	0.10	0.09	0.09	0.10	0.09
SMD($CHCl_3$)-r^{2}SCAN50-D4/pc-1	0.19	0.36	0.26	0.35	0.32	0.43	0.32
SMD($CHCl_3$)-r^{2}SCAN50-D4/pc-2	0.10	0.09	0.11	0.09	0.10	0.14	0.11
SMD($CHCl_3$)-ωB97X-D4/pc-1	0.33	0.49	0.35	0.35	0.32	0.43	0.38
SMD($CHCl_3$)-ωB97X-D4/pc-2	0.13	0.10	0.10	0.09	0.09	0.10	0.10
SMD($CHCl_3$)-ωB97M-D4rev/pc-1	0.19	0.36	0.22	0.37	0.28	0.42	0.31
SMD($CHCl_3$)-ωB97M-D4rev/pc-2	0.05	0.13	0.08	0.13	0.11	0.11	0.10

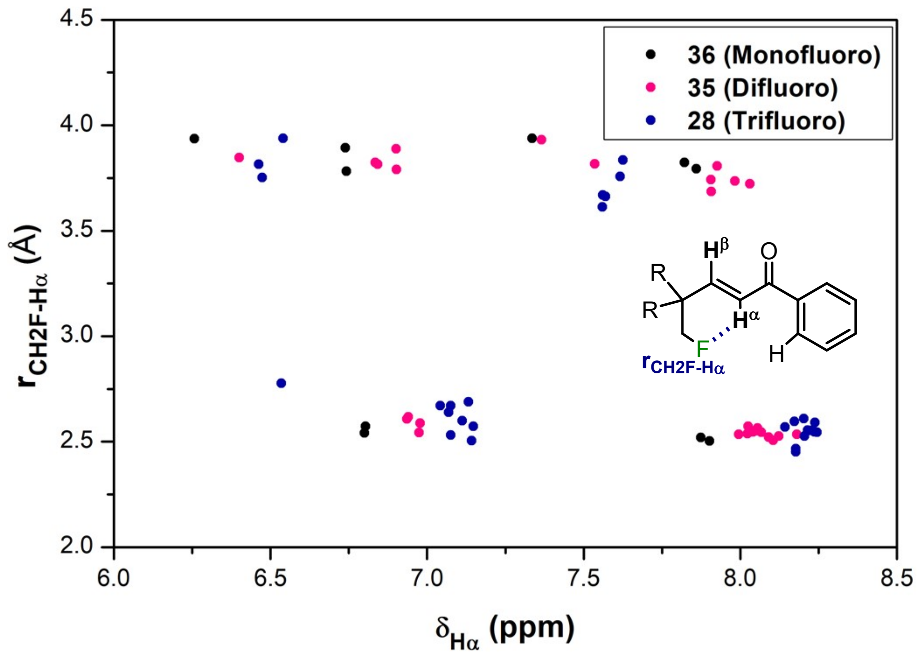

Figure S9. Plot of dH^{a} against the $CF_2H\cdots H^{a}$ distance for ketones **28**, **35** and **36**. Geometries at the SMD($CHCl_3$)-r^2SCANh-D4/pc-2 theoretical level.

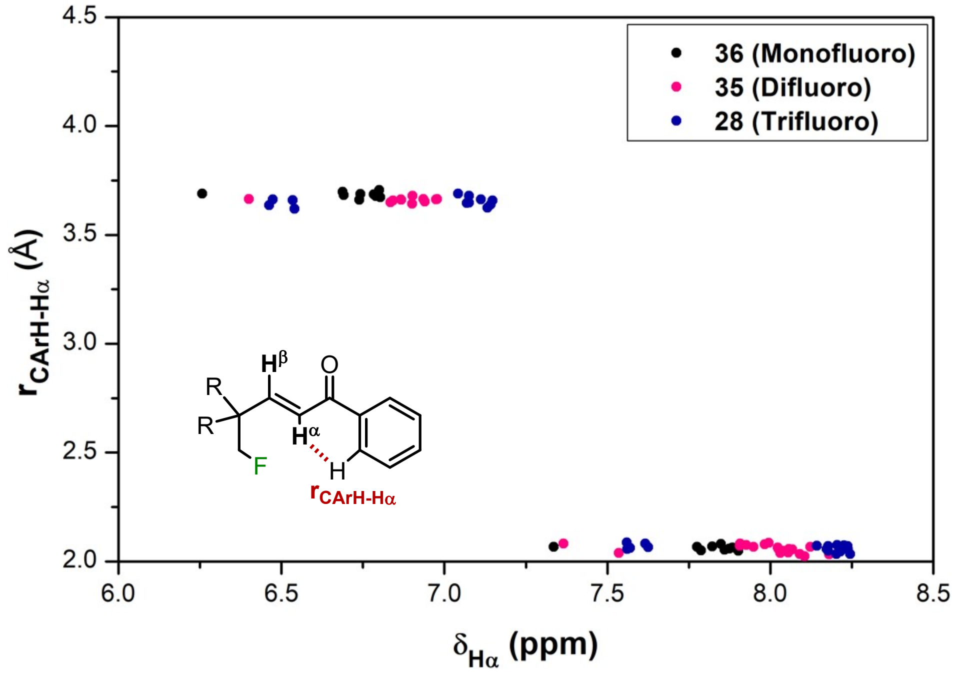

Figure S10. Plot of dH^{a} against the $C_{Ar}H\cdots H^{a}$ distance for ketones **28**, **35** and **36**. Geometries at the SMD($CHCl_3$)-r^2SCANh-D4/pc-2 theoretical level.

Table S7. Relative Gibbs free energies (ΔG, kcal mol^{-1}), Boltzmann population (Pop), lowest $CH_2F\cdots H^a$ distance (Å), H^a and H^b chemical shieldings (σH^a and σH^b, respectively, in ppm), and NCS Lewis ($\sigma_L H^a$), non-Lewis ($\sigma_{NL} H^a$) and total ($\sigma_{Total} H^a$) components of the shielding tensor of H^a, in ppm, for the monofluoro ketone derivative **28** calculated at the SMD($CHCl_3$)-r^2SCANh/pcSseg-2//r^2SCANh/pc-2 level of theory.

**Conformer**	**ΔG**	**Pop[a]**	**$CH_2F\cdots H^a$**	**σH^a**	**σH^b**	**$\sigma_L H^a$**	**$\sigma_{NL} H^a$**	**$\sigma_{Total} H^a$**
**1**	0.48	8.25%	2.52	23.75	23.77	21.56	2.19	23.75
**3**	0.00	18.65 %	3.79	23.77	23.58	14.01	9.76	23.77
**4**	0.10	15.69 %	3.82	23.80	23.54	19.51	4.30	23.80
**5**	0.45	8.77%	2.50	23.72	23.73	21.78	1.95	23.72
**6**	0.65	6.23%	4.93	23.78	23.74	14.50	9.28	23.78
**8**	0.82	4.68%	3.89	24.89	24.34	26.21	-1.32	24.89
**9**	0.96	3.66%	3.94	24.29	23.66	24.47	-0.18	24.29
**10**	0.64	6.36%	4.73	23.85	23.23	22.77	1.08	23.85
**11**	0.47	8.41%	4.73	23.84	23.21	23.15	0.69	23.84
**12**	1.41	1.73%	4.71	24.93	24.00	28.64	-3.71	24.93
**13**	1.11	2.87%	3.78	24.88	24.32	28.61	-3.73	24.88
**14**	0.57	7.10%	4.92	23.74	23.75	27.25	-3.51	23.74
**15**	1.26	2.21%	4.71	24.94	23.99	26.22	-1.29	24.94
**16**	1.48	1.54%	2.57	24.82	24.55	31.92	-7.1	24.82

**19**	1.69	1.07%	2.54	24.82	24.50	29.82	-5.00	24.82
**20**	1.53	1.42%	4.90	24.83	24.52	31.02	-6.18	24.83
**21**	1.55	1.35%	4.94	24.84	24.53	30.93	-6.09	24.84
**NCHB-forming conformers:**		19.5%	**Boltzmann-Averaged d:**	7.64[b]	7.93[b]	21.36	2.63	23.99
**no NCHB conformers:**		80.5%						

[a] *$T = 298.15K$, $K_B = 0.001987$ kcal mol^{-1} K^{-1}.*

[b] *Boltzmann-averaged d's calculated through Equation 1.*

Table S8. Relative Gibbs free energies (ΔG, kcal mol^{-1}), Boltzmann population (Pop), lowest $CH_2F\cdots H^a$ distance (Å), H^a and H^b chemical shieldings (σH^a and σH^b, respectively, in ppm), and NCS Lewis ($\sigma_L H^a$), non-Lewis ($\sigma_{NL} H^a$) and total ($\sigma_{Total} H^a$) components of the shielding tensor of H^a, in ppm, for the difluoro ketone derivative **35** calculated at the SMD($CHCl_3$)-r^2SCANh/pcSseg-2//r^2SCANh/pc-2 level of theory.

**Conformer**	**ΔG**	**Pop[a]**	**$CH_2F\cdots H^a$**	**σH^a**	**σH^b**	**$\sigma_L H^a$**	**$\sigma_{NL} H^a$**	**$\sigma_{Total} H^a$**
1	0.41	6.53%	2.55	23.57	23.76	20.96	2.60	23.57
2	0.47	5.87%	2.52	23.53	23.98	25.51	-1.97	23.53
3	0.52	5.39%	3.74	23.64	23.85	27.04	-3.40	23.64
5	0.98	2.47%	2.54	23.60	24.07	21.06	2.54	23.60
6	0.00	12.95 %	3.82	24.09	23.67	20.57	3.52	24.09
7	0.70	3.99%	3.69	23.72	23.20	27.20	-3.48	23.72
10	0.30	7.75%	3.72	23.59	23.80	19.14	4.46	23.59
11	1.07	2.13%	3.85	25.22	24.47	24.14	1.09	25.22
12	0.32	7.52%	2.55	23.56	23.76	14.59	8.96	23.56
13	0.48	5.76%	3.74	23.72	23.18	27.29	-3.57	23.72
14	1.00	2.41%	2.51	23.52	24.00	19.40	4.12	23.52
15	1.27	1.51%	3.82	24.78	23.96	25.83	-1.05	24.78
16	0.24	8.64%	4.73	23.68	23.49	27.45	-3.77	23.68
17	1.22	1.65%	2.57	23.60	23.41	22.34	1.26	23.60
18	0.82	3.26%	2.53	23.63	23.51	13.98	9.66	23.63

19	1.37	1.28%	3.89	24.72	24.60	29.52	-4.80	24.72
21	1.50	1.02%	2.54	24.65	24.79	32.49	-7.84	24.65
24	1.25	1.58%	4.71	24.76	24.29	27.23	-2.48	24.76
25	0.85	3.08%	2.56	23.57	23.39	18.82	4.75	23.57
27	0.84	3.15%	3.81	23.70	23.52	27.02	-3.32	23.70
28	0.82	3.24%	2.53	23.50	23.70	13.67	9.83	23.50
30	0.65	4.30%	2.55	23.58	24.05	15.30	8.29	23.58
33	1.20	1.70%	4.72	24.76	24.26	31.53	-6.77	24.76
34	1.36	1.30%	3.82	24.79	23.96	32.35	-7.56	24.79
**NCHB-forming conformers:**		43.2%	**Boltzmann-Averaged:**	7.82[b]	7.89[b]	22.13	1.70	23.82
**no NCHB conformers:**		56.8%						

[a] *T = 298.15K, K_B = 0.001987 kcal mol^{-1} K^{-1}.*

[b] *Boltzmann-averaged d's calculated through Equation 1.*

Table S9. Relative Gibbs free energies (Δ*G*, kcal mol^{-1}), Boltzmann population (Pop), lowest $CH_2F \cdots H^a$ distance (Å), H^a and H^b chemical shieldings (σH^a and σH^b, respectively, in ppm), and NCS Lewis ($\sigma_L H^a$), non-Lewis ($\sigma_{NL} H^a$) and total ($\sigma_{Total} H^a$) components of the shielding tensor of H^a, in ppm, for the trifluoro ketone derivative **36** calculated at the SMD($CHCl_3$)-r^2SCANh/pcSseg-2//r^2SCANh/pc-2 level of theory.

Conformer	Δ*G*	Pop[a]	$CH_2F \cdots H^a$	σH^a	σH^b	$\sigma_L H^a$	$\sigma_{NL} H^a$	$\sigma_{Total} H^a$
1	0.11	13.97 %	2.53	23.42	24.06	14.67	8.75	23.42
2	0.41	8.38%	2.55	23.39	24.04	17.94	5.44	23.39
4	0.62	5.96%	2.57	23.48	23.74	15.37	8.11	23.48
5	0.81	4.27%	3.61	24.07	23.41	20.00	4.07	24.06
6	0.41	8.40%	3.66	24.06	23.37	15.6	8.45	24.06
7	0.76	4.64%	2.60	23.45	23.37	19.83	3.62	23.45
9	1.39	1.61%	3.82	25.16	24.15	34.59	-9.43	25.16
10	0.57	6.48%	3.67	24.06	23.32	19.05	5.02	24.06
11	1.15	2.44%	2.55	23.41	23.32	14.8	8.61	23.41
13	1.42	1.54%	2.67	24.55	24.54	27.70	-3.15	24.55
14	1.56	1.21%	2.57	24.48	24.84	30.19	-5.71	24.48
15	0.00	16.86 %	3.76	24.01	23.61	19.99	4.02	24.01
16	1.66	1.03%	3.75	25.15	24.11	34.22	-9.06	25.15
17	1.57	1.19%	2.64	24.56	24.50	30.19	-5.64	24.56

18	0.62	5.89%	2.61	23.42	23.69	14.86	8.56	23.42
22	0.96	3.33%	2.47	23.45	23.73	14.52	8.93	23.45
23	0.94	3.45%	2.55	23.40	23.97	18.49	4.91	23.40
25	0.88	3.80%	2.45	23.45	23.77	14.64	8.80	23.45
26	1.41	1.55%	2.51	24.48	24.81	31.62	-7.14	24.48
27	1.33	1.79%	3.84	24.00	23.99	23.91	0.09	24.00
28	1.60	1.13%	2.55	23.38	24.31	17.44	5.94	23.38
29	1.63	1.07%	2.59	23.39	24.38	14.85	8.54	23.39
**NCHB-forming conformers:**		60.0%	**Boltzmann-Averaged d:**	7.86[b]	7.85[b]	18.80	5.00	23.79
**no NCHB conformers:**		40.0%						

[a] *$T = 298.15K$, $K_B = 0.001987$ kcal mol^{-1} K^{-1}.*

[b] *Boltzmann-averaged d's calculated through Equation 1.*

Table S10. Relative Gibbs free energies (Δ*G*, kcal mol^{-1}), Boltzmann population (Pop), lowest $CH_2F\cdots H^a$ distance (Å), H^a and H^b chemical shieldings (σH^a and σH^b, respectively, in ppm), and NCS Lewis ($\sigma_L H^a$), non-Lewis ($\sigma_{NL} H^a$) and total ($\sigma_{Total} H^a$) components of the shielding tensor of H^a, in ppm, for the monofluoro alcohol derivative **29** calculated at the SMD($CHCl_3$)-r^2SCANh/pcSseg-2//r^2SCANh/pc-2 level of theory.

**Conformer**	**Δ*G***	**Pop**[a]	**$CH_2F\cdots H^a$**	**σH^a**	**σH^b**	**$\sigma_L H^a$**	**$\sigma_{NL} H^a$**	**$\sigma_{Total} H^a$**
2	0.78	2.97%	3.77	25.81	25.15	14.95	10.86	25.81
3	0.90	2.42%	4.88	25.27	25.26	24.23	1.04	25.27
4	0.68	3.54%	3.77	25.36	25.13	28.99	-3.62	25.36
9	0.83	2.76%	2.53	25.57	25.25	28.43	-2.86	25.57
12	0.17	8.38%	4.70	25.72	24.83	17.08	8.64	25.72
14	1.16	1.56%	3.80	25.03	26.62	33.66	-8.63	25.03
15	0.95	2.25%	4.89	25.24	25.45	27.21	-1.96	25.24
17	1.09	1.77%	4.90	25.23	25.43	27.40	-2.18	25.22
20	0.50	4.78%	4.71	25.60	24.85	17.41	8.19	25.60
22	1.17	1.56%	2.54	25.80	25.31	12.12	13.68	25.80
24	1.26	1.34%	2.53	25.26	25.47	23.29	1.98	25.26
25	0.44	5.32%	2.56	25.59	25.41	21.32	4.28	25.59
27	1.12	1.69%	4.88	25.77	25.30	17.35	8.41	25.77
28	0.56	4.33%	2.55	25.61	25.40	15.80	9.82	25.61
29	1.01	2.04%	3.83	25.07	26.70	25.15	-0.08	25.07
33	1.14	1.63%	4.90	24.98	26.85	26.55	-1.57	24.98

34	0.34	6.27%	4.89	25.55	25.23	30.14	-4.59	25.55
35	0.05	10.19 %	3.76	25.62	25.03	30.25	-4.63	25.62
38	0.23	7.55%	4.89	25.54	25.40	11.94	13.60	25.54
39	1.00	2.07%	4.92	25.76	25.29	27.42	-1.66	25.76
40	0.70	3.44%	3.78	25.37	25.14	30.74	-5.37	25.37
41	0.80	2.88%	3.78	25.83	25.13	29.65	-3.82	25.82
42	1.18	1.51%	4.89	25.76	25.30	18.27	7.48	25.76
43	1.40	1.06%	4.74	25.60	24.83	27.35	-1.75	25.60
44	0.41	5.58%	3.86	25.84	25.12	27.22	-1.38	25.84
45	0.00	11.13 %	3.85	25.64	25.22	27.30	-1.66	25.64
**NCHB-forming conformers:**		15.3%	**Boltzmann-Averaged:**	6.05[b]	6.37[b]	23.76	1.82	25.57
**no NCHB conformers:**		84.7%						

[a] *T = 298.15K, K_B = 0.001987 kcal mol^{-1} K^{-1}.*

[b] *Boltzmann-averaged d's calculated through Equation 1.*

Table S11. Relative Gibbs free energies (Δ*G*, kcal mol^{-1}), Boltzmann population (Pop), lowest $CH_2F\cdots H^a$ distance (Å), H^a and H^b chemical shieldings (σH^a and σH^b, respectively, in ppm), and NCS Lewis ($\sigma_L H^a$), non-Lewis ($\sigma_{NL} H^a$) and total ($\sigma_{Total} H^a$) components of the shielding tensor of H^a, in ppm, for the difluoro alcohol derivative **37** calculated at the SMD($CHCl_3$)-r^{2}SCANh/pcSseg-2//r^{2}SCANh/pc-2 level of theory.

**Conformer**	**Δ*G***	**Pop**[a]	**$CH_2F\cdots H^a$**	**σH^a**	**σH^b**	**$\sigma_L H^a$**	**$\sigma_{NL} H^a$**	**$\sigma_{Total} H^a$**
1	0.45	5.21%	3.79	25.65	25.20	21.92	3.73	25.65
3	0.11	9.25%	4.68	25.16	24.97	26.80	-1.64	25.16
5	0.35	6.18%	2.51	24.99	25.49	21.04	3.95	24.99
6	0.88	2.52%	3.79	26.15	25.24	18.79	7.36	26.15
7	0.53	4.59%	2.47	25.01	25.46	15.17	9.85	25.01
11	1.08	1.81%	2.59	25.35	25.28	17.04	8.30	25.35
12	0.59	4.14%	3.80	25.54	25.11	17.39	8.14	25.54
13	0.75	3.12%	4.87	25.31	25.45	25.53	-0.22	25.31
15	0.00	11.15 %	2.61	25.37	25.52	19.80	5.57	25.37
24	1.27	1.30%	2.50	24.94	25.73	16.34	8.60	24.94
26	1.14	1.64%	2.56	25.57	25.32	17.53	8.05	25.57
27	0.84	2.69%	3.90	25.17	24.65	19.72	5.44	25.17
28	0.75	3.13%	3.83	25.60	25.36	23.76	1.84	25.60
30	0.75	3.13%	2.49	25.07	25.45	23.41	1.66	25.07
31	0.91	2.41%	3.83	25.71	24.69	19.15	6.56	25.71

32	0.30	6.67%	2.49	25.31	25.40	20.33	4.97	25.31
33	0.21	7.89%	3.84	25.41	25.47	23.45	1.97	25.41
34	1.28	1.29%	4.71	24.91	26.20	34.58	-9.67	24.91
35	0.71	3.39%	2.55	25.31	25.41	17.31	7.99	25.31
37	0.36	6.09%	2.56	25.35	25.45	16.06	9.29	25.35
39	0.29	6.83%	4.75	25.16	24.91	28.09	-2.93	25.16
40	1.23	1.39%	2.61	24.80	27.12	33.06	-8.26	24.80
42	0.94	2.28%	2.58	25.08	25.33	16.41	8.68	25.08
43	1.04	1.92%	4.73	24.86	26.45	34.87	-10.01	24.86
**NCHB-forming conformers:**		48.0%	**Boltzmann-Averaged d:**	6.33[b]	6.28[b]	21.73	3.57	25.30
**no NCHB conformers:**		52.0%						

[a] *T = 298.15K, K_B = 0.001987 kcal mol^{-1} K^{-1}.*

[b] *Boltzmann-averaged d's calculated through Equation 1.*

Table S12. Relative Gibbs free energies (ΔG, kcal mol^{-1}), Boltzmann population (Pop), lowest $CH_2F\cdots H^a$ distance (Å), H^a and H^b chemical shieldings (σH^a and σH^b, respectively, in ppm), and NCS Lewis ($\sigma_L H^a$), non-Lewis ($\sigma_{NL} H^a$) and total ($\sigma_{Total} H^a$) components of the shielding tensor of H^a, in ppm, for the trifluoro alcohol derivative **38** calculated at the SMD($CHCl_3$)-r^2SCANh/pcSseg-2//r^2SCANh/pc-2 level of theory.

**Conformer**	**ΔG**	**Pop[a]**	**$CH_2F\cdots H^a$**	**σH^a**	**σH^b**	**$\sigma_L H^a$**	**$\sigma_{NL} H^a$**	**$\sigma_{Total} H^a$**
1	0.56	5.58%	2.56	24.78	25.51	14.96	9.82	24.78
2	0.43	6.94%	2.53	24.83	25.50	24.01	0.82	24.83
3	0.73	4.22%	3.69	25.54	24.76	21.21	4.33	25.54
4	0.86	3.40%	2.64	24.85	25.11	16.3	8.54	24.85
5	1.11	2.21%	3.80	26.02	25.14	24.45	1.57	26.02
6	0.30	8.65%	2.53	25.16	25.66	13.33	11.84	25.16
7	1.05	2.44%	3.72	25.55	24.76	21.02	4.53	25.55
12	1.22	1.84%	3.91	25.43	25.56	18.02	7.41	25.43
14	1.28	1.68%	4.10	25.18	25.18	17.02	8.16	25.18
16	0.00	14.42 %	2.56	25.19	25.64	18.11	7.07	25.18
23	0.97	2.83%	2.62	24.89	25.31	16.72	8.18	24.89
24	0.72	4.25%	2.62	25.12	25.32	19.38	5.74	25.11
25	1.47	1.21%	2.49	24.78	25.74	24.08	0.7	24.78
26	1.31	1.58%	2.59	24.86	25.41	15.34	9.52	24.86
27	1.34	1.51%	2.61	24.90	24.84	23.71	1.19	24.9

28	0.19	10.49%	2.61	25.20	25.11	14.22	10.98	25.2
29	0.86	3.37%	2.55	24.87	25.50	19.35	5.51	24.87
31	0.60	5.25%	2.56	25.04	25.67	20.3	4.74	25.04
33	1.32	1.55%	3.79	25.21	26.67	35.08	-9.88	25.21
35	0.16	11.04%	3.80	25.87	25.19	17.53	8.33	25.87
41	0.96	2.87%	3.91	25.74	25.35	15.33	10.41	25.74
42	1.34	1.49%	2.57	24.97	25.99	23.54	1.42	24.97
45	1.49	1.17%	3.92	25.78	24.97	29.32	-3.54	25.78
**NCHB-forming conformers:**		71.0%	**Boltzmann-Averaged:**	6.40[b]	6.24[b]	18.40	6.82	25.22
**no NCHB conformers:**		29.0%						

[a] *T = 298.15K, K_B = 0.001987 kcal mol^{-1} K^{-1}.*

[b] *Boltzmann-averaged d's calculated through Equation 1.*

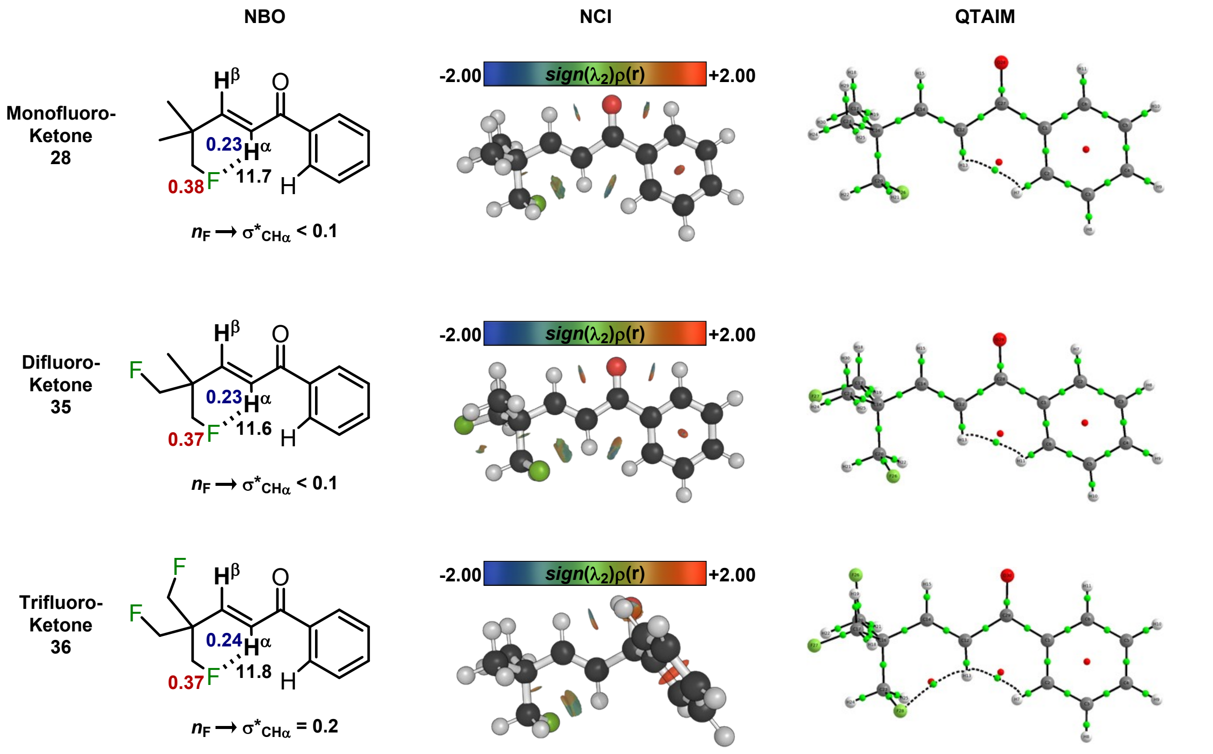

Figure S11. From left to right: NBO electrostatic (in kcal mol^{-1}, +v$_e$ in blue, -v$_e$ in red) and second order (hyperconjugative) interactions; NCI isosurfaces, using a reduced density gradient (RDG) of 0.5; QTAIM molecular graphs showing bond critical points (BPCs, green spheres) and ring critical points (RCP, red spheres), for ketone derivatives **28**, **35** and **36**.

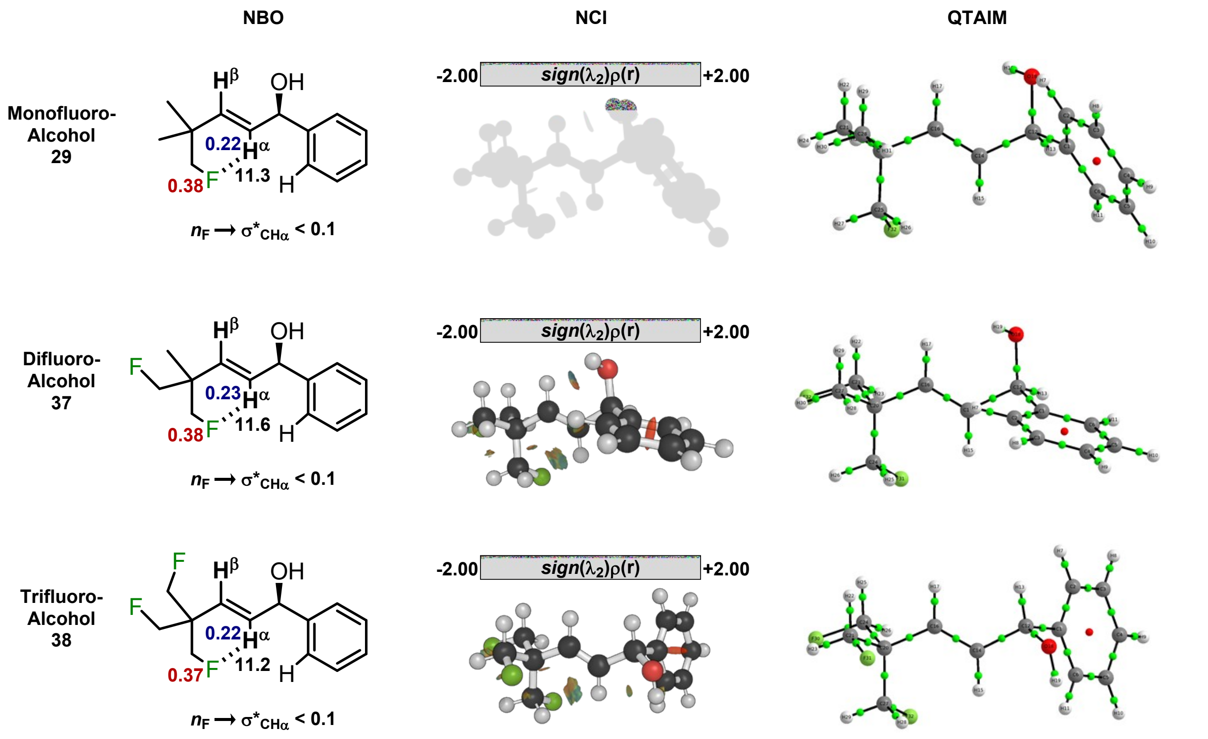

Figure S12. From left to right: NBO electrostatic (in kcal mol-1, $+v_e$ in blue, $-v_e$ in red) and second order (hyperconjugative) interactions; NCI isosurfaces, using a reduced density gradient (RDG) of 0.5; QTAIM molecular graphs showing bond critical points (BPCs, green spheres) and ring critical points (RCP, red spheres), for alcohol derivatives **29**, **37** and **38**.

Crystallography. Single crystal X-ray diffraction

X-ray diffraction data for compound **46** (Fig S13) were collected at 100 K using a Rigaku XtaLAB Synergy-R diffractometer equipped with a HyPix-Arc 100° Hybrid Photon Counting (HPC) detector, PhotonJet-R rotating anode X-ray source [Cu Kα radiation (λ = 1.54187 Å)], and confocal optics. Data were collected (using a calculated strategy) and processed (including correction for Lorentz, polarization and absorption) using CrysAlisPro.[40] The structure was solved by dual-space methods (SHELXT[41] and refined by full-matrix least-squares against F^2 (SHELXL-2025/1[42]). Non-hydrogen atoms were refined anisotropically, and carbon-bound hydrogen atoms were refined using a riding model. Hydrogen atoms bound to oxygen or nitrogen were located from the difference Fourier map and refined isotropically subject to a distance restraint. All calculations were performed using the Olex2[43] interface. CCDC 2553089 contains the supplementary crystallographic data for this paper. These data can be obtained free of charge from The Cambridge Crystallographic Data Centre via www.ccdc.cam.ac.uk/structures.

Crystal data. $C_7H_{13}F_2NO_4$, M = 213.18, monoclinic, a = 5.86046(4), b = 6.89643(6), c = 11.31648(8) Å, β = 98.5450(6) °, Vol. = 452.293(6) Å^3, T = 100 K, space group $P2_1$ (no. 4), Z = 2, 14655 reflections measured, 1795 unique (R_{int} = 0.0196), which were used in all calculations. The final R_1 [$I > 2\sigma(I)$] was 0.0223 and wR_2 (all data) was 0.0586 The Flack x parameter was -0.03(4).

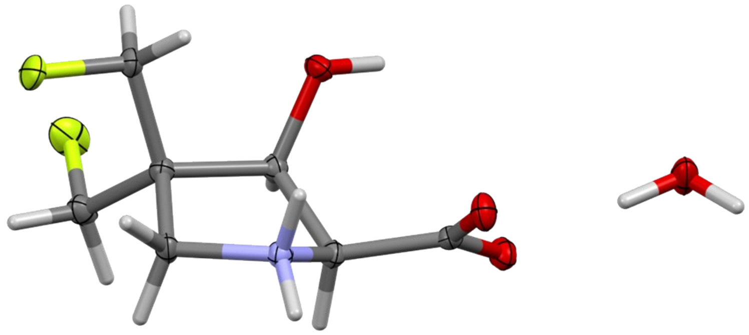

Figure S13. Thermal ellipsoid plot (50% probability ellipsoids) of the structure of **46**.

References

40 *CrysAlisPro* v1.171.44.115a. Rigaku Oxford Diffraction, Rigaku Corporation, *Tokyo, Japan*, 2025.

41. Sheldrick, G. M. SHELXT – Integrated space-group and crystal structure determination. *Acta Crystallogr., Sect. A: Found. Adv.*, 2015, **71**, 3-8. doi: 10.1107/S2053273314026370.

42. Sheldrick, G. M. Crystal structure refinement with SHELXL. *Acta Crystallogr., Sect. C: Struct. Chem.*, 2015, **71**, 3-8. Doi: 10.1107/S2053229614024218.

43. Dolomanov, O. V.; Bourhis, L. J.; Gildea, R. J.; Howard, J. A. K.; Puschmann, H. OLEX2: a complete structure solution, refinement and analysis program. *J. Appl. Crystallogr.*, 2009, **42**, 339-341. doi: 10.1107/S0021889808042726.
